# Supplementary material for: Quantifying how single dose Ad26.COV2.S vaccine efficacy depends on Spike sequence features
Source: Nat Commun. 2024 Mar 11;15:2175. doi: 10.1038/s41467-024-46536-w (PMC10928100; doi:10.1038/s41467-024-46536-w)
Supplement: Supplementary file 1 — Supplementary Information [file 41467_2024_46536_MOESM1_ESM.pdf]

Supplementary Materials for

Quantifying How Single Dose Ad26.COV2.S Vaccine Efficacy Depends on Spike Sequence Features

Craig A. Magaret, Li Li, Allan C. deCamp, Morgane Rolland, Michal Juraska, Brian D. Williamson, James Ludwig, Cindy Molitor, David Benkeser, Alex Luedtke, Brian Simpkins, Fei Heng, Yanqing Sun, Lindsay N. Carpp, Hongjun Bai, Bethany L. Dearlove, Elena E. Giorgi, Mandy Jongeneelen, Boerries Brandenburg, Matthew McCallum, John E. Bowen, David Veesler, Jerald Sadoff, Glenda E. Gray, Sanne Roels, An Vandebosch, Daniel Stieh, Mathieu Le Gars, Johan Vingerhoets, Beatriz Grinsztejn, Paul A. Goepfert, Leonardo Paiva de Sousa, Mayara Secco Torres Silva, Martin Casapia, Marcelo H. Losso, Susan J. Little, Aditya Gaur, Linda-Gail Bekker, Nigel Garrett, Carla Truyers, Ilse Van Dromme, Edith Swann, Mary A. Marovich, Dean Follmann, Kathleen M. Neuzil, Lawrence Corey, Alexander L. Greninger, Pavitra Roychoudhury, Ollivier Hyrien, and Peter B. Gilbert*

Correspondence to: pgilbert@fredhutch.org

Supplementary Note 1

Analysis of Vaccine Efficacy Against COVID-19 by SARS-CoV-2 Lineage for South Africa and for US Study Sites

In South Africa, VE appeared lower against Delta than Beta, but not significantly different (p=0.17) (**Supplementary Fig. 13a**). In the US there was no evidence of differential VE by variant (Reference vs. Alpha vs. Epsilon) (p=0.27) (**Supplementary Fig. 14a**).

Covariability Analysis of AA Positions Among the 16 Sieve Signature Sites and Among Lineages Circulating in Latin America

Supplementary Fig. 8 describes the covariability of all pairs of AA positions among the 16 positions displayed in **Fig. 2d** and **Supplementary Fig. 15**, as well as how these AA positions covary with lineages studied as indictors of a lineage (vs. not-lineage). Twelve of the 16 positions almost perfectly covaried with Lambda and with one another [Mstar[1] > 0.9]: these positions include mutations characteristic of Lambda but not present in other variants, such that the differential VE results are nearly equivalent to differential VE against Lambda (11% VE) vs. not-Lambda (56% VE). One position (249) strongly covaried with Lambda (Mstar = 0.87). Another position (501, in the RBM) had near-perfect covariability with several lineages [N501 for almost 100% of Reference and 100% of Epsilon, Lambda and Zeta sequences, and Y501Y for 100% of Alpha, Gamma and Mu sequences], but it did not have a high Mstar value because the covariability analysis studied indicators of each lineage vs. not-lineage. The remaining two positions [414 (in S1's C-terminal domain) and 778 (in S2)] did not covary strongly with any lineage or with any of the other 16 positions and hence constitute new signatures of differential VE beyond what is already captured by Lambda vs. not-Lambda.

Correlations among Deep Mutational Scanning (DMS), Protein Data Bank (PDB), and Physicochemical-Weighted Spike Sequence Features

Supplementary Fig. 23 shows the hierarchical clustering of the physicochemical-weighted Hamming distance, DMS, and PDB sequence features and **Supplementary Fig. 24** shows a correlation matrix for the physicochemical-weighted Hamming distance, DMS, and PDB sequence features ordered by the hierarchical clustering shown in **Supplementary Fig. 23**.

Spike (amino acid positions 1-1273) contains S1 (13-685), which contains the disjoint sets NTD (13-303) and RBD (319-541). The physicochemical-weighted sequence features tend to be highly correlated (e.g., Pearson correlation 0.8 for RBD and NTD, and ≥ 0.91 for the nested physicochemical-weighted sequence features). Spike, S1, and NTD form a cluster with PDB8, with correlations ranging from 0.75 to 0.99. DMS, DMS2, DMS7 and physicochemical-weighted RBD also form a cluster with correlations ranging from 0.81 to 0.91. DMS8 (an epitope cluster in the RBD) is highly correlated with PDB13 (a footprint in the N-terminal domain, NTD) (correlation 0.93). This correlation seems to be driven by co-variation in the RBD and NTD, as well as by the Mu and Lambda variants: Mu has mutations at positions 145 in the NTD and 346 in the RBD, while Lambda has a mutation at position 246 and deletions at 247-253 (all in the NTD) as well as mutations at positions 452 and 490 in the RBD (**Supplementary Table 2**). DMS6 is highly correlated with PDB4 and with PDB7 (correlations 0.95 and 0.86, respectively). Notably, the sites most influential for DMS6 are sites in the PDB4 footprint.

Limited Differential Vaccine Efficacy against COVID-19 and Against Severe-Critical COVID-19 by Virus Features for United States Study Sites

For the COVID-19 endpoint, most of the features with significant sieve effects for Latin America were not significant for the US. Here we note all significant results (q-value ≤ 0.20). Two AA positions had differential VE: 152 and 452, the latter being a neutralization-hypothesized site with FWER p = 0.0056 for the hypothesized features. VE against 452-vaccine-matched COVID-19 (452L) was 77% (70%, 82%) vs. 31% (-40%, 66%) for 452-vaccine-mismatched COVID-19 (452R). Results at site 152 were similar (**Supplementary Fig. 14**). VE significantly declined with weighted Hamming distance in Spike, RBD, NTD, and S1 (FWER p-values 0.044-0.14; q-values 0.044-0.086) (**Supplementary Figs. 17-20, Supplementary Table 12**). Of the 16 evaluable antibody escape score features in the US cohort, one feature (PDB8) had declining VE with escape score (FWER p = 0.067, q-value = 0.067) (**Supplementary Fig. 39d, Supplementary Table 16**). All features showing sieve effects for COVID-19 in the US also had sieve effects in Latin America.

For the severe–critical COVID-19 endpoint, there was evidence of differential VE in the US by some viral features. The point estimate of VE sharply decreased with weighted Hamming distance for Spike, RBD, NTD, S1 (FWER p-values 0.014-0.071), compared to Latin America with less evidence for decreasing VE (p-values 0.099-0.26, FWER p-values 0.49-0.52) (**Supplementary Figs. 45-48**, **Supplementary Table 23**). However, with only 8 non-Reference genotype severe-critical COVID-19 endpoints in the US (3 vaccine arm, 5 placebo arm) that cause the drop in VE, and with different distributions of non-Reference variants circulating in Latin America and the US, it is not possible to infer a difference of results between these geographic regions.

A similar pattern of results was seen for the US compared to Latin America for the two antibody-escape scores that qualified for analysis (DMS2, DMS6; **Supplementary Figs. 51, 52**). These findings need to be regarded as hypothesis-generating given the small number of severe-critical COVID-19 vaccine breakthrough viruses.

No Evidence of Differential Vaccine Efficacy at South Africa Study Sites

No sieve analyses showed significantly differential VE by any of the virus features studied, for either study endpoint, where results were notably flat without trends toward significance, with one possible exception the trend for VE against COVID-19 being higher against Beta than Delta as noted earlier. For the approximately 90% of sequences that were Beta or Delta, the median (range) of placebo-arm Spike distances to the vaccine-sequence was 15.1 (9.8, 23.4), compared to 7.1 (6.0-16.7) for the US and 9.5 (6.0-27.7) for Latin America, highlighting how both South Africa and the US had more limited sequence variability compared to Latin America.

Structure and Immune Evasion of the Lambda Variant Glycoprotein

The binding kinetics of RBDs to ACE2 as assessed by biolayer interferometry (BLI), surface plasmon resonance (SPR), and ELISA, as well as the effect of Lambda mutations on neutralizing monoclonal antibody binding, are provided in **Supplementary Figs. 53** and **54**, respectively.

Supplementary Discussion

The signature site 452 in the RBM was previously shown to have multiple functions, with the L452R mutation associated with cellular immune escape and increased Spike stability, viral infectivity, and viral fusogenicity, aiding viral replication.[2] Moreover, the L452R mutation (in the context of the Epsilon variant) was shown to be associated with reduced neutralization by sera of mRNA vaccine recipients.[3] However, 452R was not represented among COVID-19 endpoints in Latin America – the sieve effect was for 452L vs. 452Q (56% VE vs. 18% VE), with 452Q a characteristic mutation for the Lambda variant only. For the signature position 490, previous studies showed that the F490S mutation in the RBM was associated with a reduced neutralizing antibody response from BNT162b2 vaccine recipients.[4] Given that 490S is a characteristic residue only for Lambda, the 490/Lambda differential VE results may be interpreted as contributing to validating neutralizing antibodies as a CoP, given that the finding of abrogated neutralization by F490S translated into reduced VE. It is noteworthy that the neutralization resistance signature result for the BNT162b2 mRNA vaccine translated across vaccine platforms to a VE signature for the Ad26.COV2.S vaccine. There was not a sieve effect at neutralization-hypothesized site 484, which may be explained by the fact that the wild-type residue for this site ("E") is characteristic of both the lineage found to have the highest VE (the Reference lineage) and the lineage found to have the lowest VE (Lambda), whereas the mutant residue observed at this site ("K") is characteristic of almost all other variants. The N501Y mutation is found in several variants and has been suggested to increase transmission in an animal model as well as in human primary epithelial cells.[5]

Positions 414 (in S1's C-terminal domain) and 778 (in S2) each had an opposite result from that of typical sieve effects, with VE higher against viruses with a residue mismatch than match to the vaccine strain, with minority residues R and I respectively overrepresented in placebo arm COVID-19 endpoint sequences. A potential explanation is that the vaccine pressured the virus to be maximally fit at these positions.

Supplementary Methods

SARS-CoV-2 Sequence Data

As stated in Methods, sequences were selected for this analysis if they were obtained within 36 days following the first RNA-positive timepoint associated with the first moderate to severe-critical COVID-19 primary endpoint. This 36-day window corresponds to the Day 29 visit following a COVID-19 endpoint plus the seven-day visit window prescribed by the trial protocol.

Participants with SARS-CoV-2 viral load values and missing sequences were included in the analyses by modeling the probability of observing a sequence as a function of viral load and geographic region using logistic regression models. **Supplementary Figs. 55** and **56** show the probability of observing a sequence for the primary endpoint and the severe-critical COVID-19 endpoint, respectively, as a function of log10 viral load separately by region and subregion based on region-specific logistic regressions. The geographic region-pooled analysis included log10 viral load, indicators of the geographic regions (US, South Africa, Colombia, Latin America excluding Colombia) together with all two-way interactions of region indicators and log10 viral load. The US and South Africa analyses included log10 viral load only, and the Latin America analysis included the indicator of Colombia, log10 viral load, and their interaction.

Calculation of Physicochemically Weighted Hamming Distances

Sandberg et al.[6] characterized 87 amino acids, including the 20 biogenic amino acids, by 26 different physicochemical descriptors of experimentally determined values. They then rendered the 26 variables down to five principal components, indicated as z_1 through z_5 (referred to collectively as the *z*-scales). These five components correspond to:

z_1: hydrophobicity
z_2: steric bulk/volume
z_3: polarity
z_4: electronegativity
z_5: electophilicity

As principal components, the five *z*-scale values are orthogonal to each other in *z*-space, and their physicochemical distance between any two amino acids can be calculated as the five-dimensional orthogonal distance between the two amino acids' *z*-scale values.

We extended this method to be used as a weighting approach when calculating the Hamming distances: when calculating the distance between two sequences, if the residues at a given site are identical, they do not contribute to the total distance (per the standard Hamming distance method); if the pair of residues differ, instead of increasing the distance by 1, we increase the distance by the residues' physicochemical distance in *z*-space. These individual distances are summed for every position in the sequence string and the total distance is used in the analysis.

GenSig

We used the LANL tool GenSig to identify signature sites associated with vaccine status after phylogenetic correction that accounts for potentially spurious associations due to lineage effects.[7,8] Because ancestral reconstructions are done on the nucleotide sequences, we assembled a set of 1,159 codon-aligned nucleotide SARS-CoV-2 sequences of the S gene (constituting all nucleotide sequences available from the study participants) and ran the analyses on the pooled cohort (all regions combined), as well as on the three region-specific cohorts: Latin America (n=732), North America (n=314), and South Africa (n=113). We considered all Spike sites, with an FDR multiple testing correction of q=0.2 and considered nominal p-values less than 0.05 for sites previously found to be associated with changes in bnAb neutralization and/or binding.

AA Sequence Sieve Analysis Methods: Prospective VE sieve analysis

Some of the R packages used for the hazard ratio-based prospective VE sieve analyses are provided in the main text Methods. Note that the cited code at ref.[9] cannot reproduce the results of this work due to the fact that it would use a different seed number for random number generation. In addition, for parallel computation, the R packages doParallel[10] (version 1.0.17), foreach[11] (version 1.5.2), iterators[12] (1.0.14), and parallel (4.2.2) were used. For data pre-processing, the R packages here[13] (version 1.0.1), haven[14] (version 2.5.2), scales[15] (version 1.2.1), tidyverse[16] (version 2.0.0), plyr[17] (version 1.8.8), and dplyr[18] (version 1.1.2) were used. Additional R packages used for hazard ratio-based sieve model fitting included survival (3.5-5), MASS[19] (version 7.3-60), and nnet[19] (version 7.3-19). For creating tables and plots with results, the R packages ggpubr[20] (version 0.6.0), ggpmisc[21] (version 0.5.2), ggpol[22] (version 0.0.7), gridExtra[23] (version 2.3), gtable[24] (version 0.3.3) and ggradar[25] (GitHub version) were used.

Classification Sieve Analysis

For data pre-processing, the R packages here[13] (version 1.0.1), dplyr[18] (version 1.1.2), tidyr[26] (version 1.3.0), readr[27] (version 2.1.4), and argparse [28] (version 2.2.2) were used. For estimating functions that predict trial arm given covariates, the R packages here[13] (version 1.0.1), dplyr[18] (version 1.1.2), tidyr[26] (version 1.3.0), readr[27] (version 2.1.4), argparse[28] (version 2.2.2), SuperLearner[29,30] (version 2.0-28.1), MASS[19] (version 7.3-60), arm[31] (version 1.13-1), kernlab[32] (version 0.9-32), ranger[33] (version 0.15.1), xgboost[34] (version 1.7.5.1), glmnet[35] (version 4.1-7), earth[36] (version 5.3.2), and parallel (version 4.3.1) were used. For VIM analysis, the R packages here[13] (version 1.0.1), dplyr[18] (version 1.1.2), tidyr[26] (version 1.3.0), tibble[37] (version 3.2.1), argparse[28] (version 2.2.2), SuperLearner[29,30] (version 2.0-28.1), and vimp[38-40] (version 2.3.1) were used. For creating plots and tables with results, the R packages tidyverse[16] (version 2.0.0), cowplot[41] (version 1.1.1), vimp[38,39] (version 2.3.1), data.table[42] (version 1.14.8), optparse[43] (version 1.7.3), and knitr[44] (version 1.45) were used.

Antibody Escape Scores

Additional information on deep mutational scanning (DMS) antibody escape scores:

Identification of epitope-specific escape scores: Based on deep mutational scanning data from 228 SARS-CoV-2-elicited neutralizing antibodies (data captured April 21, 2022; https://raw.githubusercontent.com/jbloomlab/SARS2_RBD_Ab_escape_maps/651fe6fa5a7fccec2b662ddbb45b6d2c7421ae74/processed_data/escape_calculator_data.csv),[45] we used

hierarchical clustering based on Euclidean distances and complete linkage for the purpose of identifying potential antibody footprints that would be defined by a set of amino acid sites. Based on the goal of identifying putative epitope footprints consisting of approximately 15 amino acid positions to define a cluster and a relatively small number of clusters, we cut the resulting dendogram at heights that generated between K=2 and 21 non-singleton clusters from the tree. Singleton clusters were deemed to be uninformative and were dropped from further analysis. For each cluster we selected sites with an average escape of greater than 0.05 to define a putative antibody footprint. Based on these criteria we selected K=8 non-singleton clusters whose defining putative epitope footprint amino acid sites are shown in **Supplementary Table 1**.

Calculation of antibody-escape scores: Each sequence was assigned an epitope-specific antibody-escape score relative to the D614G Reference strain, based on the RBD sequence and the set of antibodies identified by each cluster from **Supplementary Table 1**. Additionally, a total escape score is computed based on all antibodies in the DMS data set. The score, defined as one minus the binding retained [see Greaney et al.[45]], is a distance from 0 to 1 where 0 is no escape and 1 is full escape. **Supplementary Table 1** lists the number of overlapping sites between the putative epitope footprint defined by each cluster and the set of the RBD sites defined in Greaney et al.[46] For the epitope region, classes 1, 2, and 3 were defined by Barnes et al.[47]

Supplementary Table 1. DMS antibody escape score-based putative epitope footprints (clusters).

Cluster	n[1]	n Sites[2]	Sites[3]	Class 1[4]	Class 2[5]	Class 3[6]	Other
1	38	14	417, 420, 421, 453, 455, 456, 460, 463, 472, 473, 475, 476, 487, 489	12	5	0	1
2	55	12	346, 348, 352, 417, 420, 455, 456, 468, 473, 475, 493, 504	7	3	1	3
3	30	10	456, 472, 473, 475, 476, 484, 485, 486, 487, 489	7	7	0	0
4	1	9	472, 478, 479, 481, 482, 483, 484, 485, 486	1	5	0	4
5	53	11	449, 452, 456, 472, 483, 484, 485, 486, 490, 493, 494	2	9	2	0
6	24	13	346, 443, 444, 445, 446, 447, 448, 449, 450, 452, 490, 494, 499	0	2	11	0
7	6	14	443, 445, 446, 447, 449, 455, 456, 484, 490, 494, 496, 498, 500, 501	2	5	9	0
8	3	15	356, 357, 444, 445, 446, 447, 448, 449, 450, 452, 490, 494, 496, 519, 525	0	2	9	4
9	18	18	335, 361, 369, 370, 372, 374, 375, 376, 378, 383, 384, 385, 408, 413, 417, 427, 441, 503	1	0	1	16
10	1	13	349, 356, 374, 377, 378, 381, 394, 410, 435, 466, 468, 507, 525	0	0	0	13

[1] Number of antibodies per cluster. Fifteen antibodies were measured separately in two different labs. Antibody C110 clustered into different clusters (2 and 6) depending on the lab that ran the assay and this antibody is counted twice.
[2] Number of AA sites with an average escape of greater than 0.05.
[3] List of AA sites.
[4] Class 1 epitope residues (from ref.[46]): 403 + 405 + 406 + 417 + 420 + 421 + 453 + 455-460 + 473-476 + 486 + 487 + 489 + 504.
[5] Class 2 epitope residues (from ref.[46]): 455 + 456 + 472 + 483-487 + 489 + 490 + 491 + 492 + 493 + 494.
[6] Class 3 epitope residues (from ref.[46]): 345 + 346 + 437-452 + 496 + 498-501.
Note that five residues overlap between the Class 1 and Class 2 epitopes: 455 + 456 + 486 + 487 + 489.

Singleton clusters 4 and 10 were excluded from the analysis. See Section 2.1.4 of the SAP for more details on how the clusters were defined.

Protein Data Bank (PDB) antibody escape scores:

In brief, a weight of 1.0 was attributed to the average interaction across all epitope sites. Neighboring residue pairs were identified by Delaunay tetrahedralization of side-chain centers of residues (Ca was counted as a side chain atom, pairs further than 8.5 Angstrom were excluded). Antibody epitope footprints were compared for all pairs of antibodies across the 274 anti-Spike antibodies (see Section 2.1.5 of the SAP for further details), with footprint similarities ranging from 0 (no overlap of the binding sites) to 1 (identical binding sites). The similarity was then transformed into a distance (1 – similarity) and hierarchical clustering was used to define clusters of AA sites based on the distance matrix (distance cutoff of 0.8), identifying 14 clusters, i.e. clustering the antibodies based on the similarity of their epitope footprint. Each cluster included at least 3 antibodies.

For each of the 14 clusters, **Supplementary Table 2** provides its location within the Spike protein, the number of antibodies in the cluster, the representative antibody, the representative PDB complex, and the number of AA sites in the cluster. **Supplementary Table 3** provides the epitope footprint of each cluster and the weight of the interaction.

A representative antibody was identified for each cluster (**Supplementary Table 2**), and the epitope distance between a virus sequence and the vaccine insert sequence was calculated as a weighted mean of the distance between all epitope sites (see Section 2.1.5 of the SAP for details).

Supplementary Table 2. Protein Data Bank (PDB) epitope footprints for antibodies representative of the 14 clusters.

Cluster	Location in Spike	n Ab[1]	Representative Antibody	Representative PDB complex	Link to representative PDB complex	n Sites[2]
1	RBD	61	P22A-1D1	7chs (E:HL)	https://www.rcsb.org/structure/7CHS	36
2	RBD	39	S2E12	7r6x (R:CD)	https://www.rcsb.org/structure/7R6X	19
3	RBD	12	Beta-40	7ps7 (R:HL)	https://www.rcsb.org/structure/7PS7	16
4	RBD	18	A19-61.1	7tbf (A:DE)	https://www.rcsb.org/structure/7TBF	20
5	RBD	12	BD-812	7ezv (A:HL)	https://www.rcsb.org/structure/7EZV	18
6	RBD	9	A5-10	7f7e (E:CL)	https://www.rcsb.org/structure/7F7E	18
7	RBD	10	35B5	7e9p (B:HL)	https://www.rcsb.org/structure/7E9P	29
8	RBD	44	MW01	7djz (C:AB)	https://www.rcsb.org/structure/7DJZ	29
9	RBD	11	C022	7rku (A:GI)	https://www.rcsb.org/structure/7RKU	29
10	RBD	15	3D11	7m7b (A:HL)	https://www.rcsb.org/structure/7M7B	20
11	RBD	4	FD20	7cyv (B:H)	https://www.rcsb.org/structure/7CYV	20
12	RBD	4	47D11	7akd (B:HL)	https://www.rcsb.org/structure/7AKD	23
13	NTD	16	2-51	7l2c (B:CD)	https://www.rcsb.org/structure/7L2C	17
14	NTD	4	2490	7dzy (B:HL)	https://www.rcsb.org/structure/7DZY	26

[1] Number of antibodies in the designated cluster.
[2] Number of AA sites in the designated cluster.

Supplementary Table 3. Amino acid sites and weights for each site in each of the 14 clusters.

Cluster	(Site:Weight)
1	403:1.04, 405:0.16, 406:0.16, 409:0.16, 415:1.21, 416:0.92, 417:2.10, 420:0.91, 421:1.79, 453:0.62
1	455:1.79, 456:1.24, 457:0.29, 458:1.66, 459:0.32, 460:0.91, 473:1.34, 474:0.32, 475:2.24, 476:1.08
1	477:0.46, 486:1.69, 487:1.81, 489:1.50, 493:1.93, 494:0.16, 495:0.30, 496:0.76, 497:0.16, 498:0.78
1	500:0.78, 501:1.36, 502:1.04, 503:0.30, 504:0.16, 505:2.57
2	417:0.41, 455:1.23, 456:0.55, 458:0.14, 473:0.79, 475:1.50, 476:1.12, 477:1.12, 478:1.12, 479:0.14
2	480:0.14, 483:0.14, 484:0.60, 485:1.45, 486:3.38, 487:1.67, 488:0.33, 489:1.86, 493:1.31
3	403:0.14, 444:0.14, 445:2.76, 446:1.46, 447:0.14, 449:1.31, 493:0.73, 494:0.14, 496:0.44, 498:1.16
3	499:1.16, 500:2.91, 501:1.89, 502:0.58, 504:0.14, 505:0.87
4	345:0.50, 346:1.59, 440:0.43, 441:0.72, 442:0.17, 443:0.60, 444:2.96, 445:2.52, 446:1.10, 447:0.72
4	448:1.15, 449:3.34, 450:0.83, 452:0.33, 490:0.33, 492:0.38, 493:0.72, 494:1.10, 496:0.33, 499:0.17
5	345:0.61, 346:2.04, 439:0.61, 440:1.78, 441:1.05, 442:0.33, 443:0.33, 444:2.37, 445:2.43, 446:0.63
5	447:0.77, 448:0.33, 449:0.33, 450:0.47, 498:0.61, 499:0.91, 500:2.25, 502:0.16
6	343:0.93, 345:1.97, 346:0.53, 439:0.92, 440:2.11, 441:2.24, 442:0.92, 443:1.45, 444:0.94, 445:1.47
6	446:0.14, 448:0.67, 450:0.27, 451:0.39, 498:0.14, 499:1.33, 500:1.18, 509:0.39
7	339:0.18, 340:1.71, 341:0.37, 344:0.84, 345:1.79, 346:4.37, 347:0.66, 348:1.89, 349:0.55, 351:0.95
7	352:0.66, 354:1.31, 356:0.95, 399:0.18, 449:2.18, 450:1.97, 451:0.37, 452:0.18, 466:1.31, 468:1.42
7	469:0.37, 470:1.89, 472:0.18, 481:0.29, 482:0.37, 483:0.37, 484:0.37, 490:1.13, 492:0.18
8	351:0.36, 403:0.19, 417:0.72, 444:0.38, 445:0.38, 446:0.73, 447:0.19, 449:2.52, 450:0.54, 452:1.46
8	453:0.72, 455:0.74, 456:0.36, 470:0.55, 472:0.19, 475:0.19, 481:0.19, 482:0.38, 483:1.28, 484:3.27
8	485:2.20, 486:3.06, 487:0.91, 488:0.19, 489:1.63, 490:2.01, 492:0.74, 493:1.81, 494:1.09
9	369:1.03, 370:0.17, 371:0.52, 372:0.17, 374:0.17, 377:1.03, 378:1.72, 379:1.72, 380:1.38, 381:1.22
9	382:0.69, 383:1.55, 384:1.04, 385:0.52, 405:0.17, 408:2.41, 409:0.17, 411:0.35, 412:1.04, 413:1.91
9	414:1.74, 415:1.39, 416:0.52, 417:0.17, 427:2.76, 428:1.72, 429:1.03, 430:0.52, 460:0.17
10	366:0.17, 369:1.18, 370:1.89, 371:0.68, 372:1.53, 374:0.68, 375:1.53, 376:1.20, 377:1.20, 378:2.22
10	379:0.85, 380:0.17, 381:0.17, 383:1.53, 384:1.36, 385:1.71, 407:0.17, 408:1.21, 411:0.17, 414:0.34
11	353:0.39, 354:0.81, 355:2.40, 356:0.39, 357:0.98, 396:0.39, 426:0.19, 427:0.39, 428:1.19, 459:0.77
11	461:0.40, 462:3.40, 463:1.38, 464:0.20, 465:2.79, 466:2.61, 468:0.19, 516:0.39, 518:0.39, 519:0.39
12	332:0.83, 333:0.18, 334:0.91, 335:2.44, 336:1.52, 337:1.89, 338:1.48, 339:3.01, 340:1.89, 342:0.69
12	343:2.69, 362:0.18, 363:0.36, 364:1.20, 365:0.18, 367:0.55, 368:0.83, 370:0.18, 371:0.18, 373:0.18
12	374:0.18, 527:0.36, 529:1.06
13	144:0.64, 145:1.33, 146:0.39, 147:2.36, 148:0.66, 150:1.01, 152:0.60, 246:1.45, 247:0.34, 248:2.11
13	249:1.22, 250:1.56, 251:2.02, 252:0.66, 253:0.39, 254:0.14, 257:0.14
14	66:0.19, 68:0.19, 69:0.39, 70:0.78, 71:0.39, 72:0.78, 73:0.19, 75:0.19, 98:0.19, 180:0.19, 181:4.72
14	182:4.92, 183:0.97, 184:1.36, 185:0.97, 186:0.19, 211:4.53, 212:0.39, 213:1.16, 214:1.16, 215:1.16
14	218:0.19, 261:0.19, 262:0.19, 263:0.19, 264:0.19

For each antibody footprint set, vaccine efficacy was assessed by the quantitative sequence feature variable computed in each of the two ways described above (DMS antibody escape score and PDB antibody escape score).

Variant-Neutralization Sensitivity Score Assigned to Variants

For each variant that caused a COVID-19 primary endpoint, a variant-neutralization resistance score is assigned to the variant/lineage based on 50% neutralization titers of Day 71 ENSEMBLE vaccine recipient sera to a panel of variants, analyzed in a Janssen-internal reference strain (D614G) pseudotyped lentivirus neutralization assay (psVNA). Eight samples were selected based on high D614G psVNA titers, which were then run in internal psVNAs for different variants including Alpha, Beta, Delta, Gamma and Zeta (**Supplementary Table 4**). Two technical replicates were performed for each serum sample, i.e. heat-inactivated serum samples were two-fold serial diluted in duplicates over 10 columns.

For each variant, the score is defined as log10 fold-change of the geometric mean ID50 titer to the variant vs. to the reference strain for per-protocol baseline seronegative vaccine recipients in a phase one trial of the Ad26.COV2.S vaccine. Variants causing COVID-19 endpoints that were not included in the psVNA measurements were assigned the score of a scored variant that was shown in other studies to have similar neutralization sensitivity. Further details are in Section 2.1.7 of the SAP.

No data in the neutralization resistance scoring experiment were available for Epsilon, Iota, Lambda, or Mu, which were assumed to have the same neutralization resistance level as Delta, Alpha, Delta, and Beta, respectively, due to sequence similarity between these lineages and support from the COV2001 study that measured neutralization levels to all of these variants.[48]

Supplementary Table 4. Neutralization activity of serum antibodies elicited by Ad26.COV2.S against SARS-CoV-2 Spike pseudotyped virus variants.

	Reference (B.1)	Alpha (B.1.1.7)	Beta (B.1.351)	Delta (B.1.617)	Delta (B.1.617.2)	Gamma (P.1)	Zeta (P.2)
GMT	246	266	68	162	154	72	115
Fold-Change Over B.1	N/A	0.9	3.6	1.5	1.6	3.4	2.2
95% LCL Fold-Change	N/A	0.8	2.1	1.0	1.2	2.1	1.2
95% UCL Fold-Change	N/A	1.0	6.2	2.4	2.2	5.7	3.8
p-value*	N/A	0.118	0.001	0.060	0.007	0.001	0.016

GMT, geometric mean titer.
* The 2-sided p-value tests whether the GM fold-change differs between the variant and the Reference virus B.1. Statistical comparison of the fold differences relative to B.1 were performed in SAS 9.4 using a paired-sample T-test at a 5% significance level. No adjustment for multiple comparisons was done.
The Epsilon, Iota, Lambda, and Mu variants were assigned the same fold-change values as Delta, Alpha, Delta, and Beta, respectively, where for Delta the value of 1.55 is used (average of the value for Delta B.1.6.17 and Delta B.1.617.2).

Spike Sequence Feature Classes and Notation

Three related classes of sequence features weigh mutations in the Spike protein relative to the vaccine-insert protein. To simplify naming conventions and figure labeling, each sequence feature class uses a specific notation. The sequence feature classes and notations are as below:

1) The physicochemical-weighted Hamming distances to the vaccine-insert sequence in Spike and its sub-domains.
- The physicochemical-weighted Hamming distances are labelled Spike, RBD, NTD, S1 and S2.

2) Receptor binding domain (RBD) antibody escape scores based on the Deep Mutational Scanning (DMS) assay,[45] referred to as DMS scores.
- The DMS sequence features are for the whole-RBD region (labeled DMS) and 8 antibody clusters (named DMS1 through DMS9 excluding DMS4; see Section 2.1.4 of the SAP for details on clustering and naming).

3) Protein Data Bank (PDB) scores defined by putative antibody footprint site sets based on Spike and human antibody complexes available in the PDB.
- The PDB scores are numbered 1 to 14 with the first 12 footprints in RBD and the footprints 13 and 14 in NTD.

Supplementary Table 5. All variants used in this sieve analysis, listed by geographic region, along with sequence counts and characteristic mutations that define each variant in Spike relative to the Wuhan-Hu-1 index virus and vaccine-insert reference strain Spike that define each variant. This table was generated in Python (version 3.10.2).

Region	WHO Label	Number Observed[1]		Characteristic Variant Mutations[2]
		Vaccine	Placebo	
Latin America	Reference	72	196	D614G
Latin America	Alpha	4	10	H69del, V70del, Y144del, N501Y, A570D, D614G, P681<br>H, T716I, S982A, D1118H
Latin America	Epsilon	0	2	S13I, W152C, L452R, D614G
Latin America	Gamma	73	111	L18F, T20N, P26S, D138Y, R190S, K417T, E484K, N501Y, D614G, H655Y, T1027I, V1176F
Latin America	Lambda	43	45	G75V, T76I, R246N,247del, Y248del, L249del, T250del, P251del, G252del, D253del, L452Q, F490S, D614G, T859N
Latin America	Mu	38	57	T95I, ins143T, Y144S, Y145N, R346K, E484K, N501Y, D614G, P681H, D950N
Latin America	Zeta	33	92	E484K, D614G, V1176F
United States				
United States	Reference	52	221	D614G
United States	Alpha	4	16	H69del, V70del, Y144del, N501Y, A570D, D614G, P681H, T716I, S982A, D1118H
United States	Epsilon	8	15	S13I, W152C, L452R, D614G
United States	Gamma	1	0	L18F, T20N, P26S, D138Y, R190S, K417T, E484K, N501Y, D614G, H655Y, T1027I, V1176F
United States	Iota	0	4	L5F, T95I, D253G, E484K, D614G, A701V

United States	Zeta	1	1	E484K, D614G, V1176F
South Africa	Alpha	1	2	H69del, V70del, Y144del, N501Y, A570D, D614G, P681H, T716I, S982A, D1118H
South Africa	Reference	1	4	D614G
South Africa	Beta	36	59	D80A, D215G, L242del, A243del, L244del, K417N, E484K, N501Y, D614G, A701V
South Africa	Delta	11	10	T19R, T95I, G142D, E156del, F157del, R158G, L452R, T478K, D614G, P681R, D950N
South Africa	Lambda	0	1	G75V, T76I, R246N, S247del, Y248del, L249del, T250del, P251del, G252del, D253del, L452Q, F490S, D614G, T859N

[1] From the next generation sequencing data, a single consensus sequence was assembled for each participant. "Number Observed" corresponds to the number of participants with a consensus sequence corresponding to the designated variant.

[2] "Characteristic mutations" are defined as those present in canonical variant sequences.[49] There may be some heterogeneity across different sequences of the same variant due to background mutations, but all sequences categorized as a given variant, with only rare exceptions, harbor all the characteristic mutations.

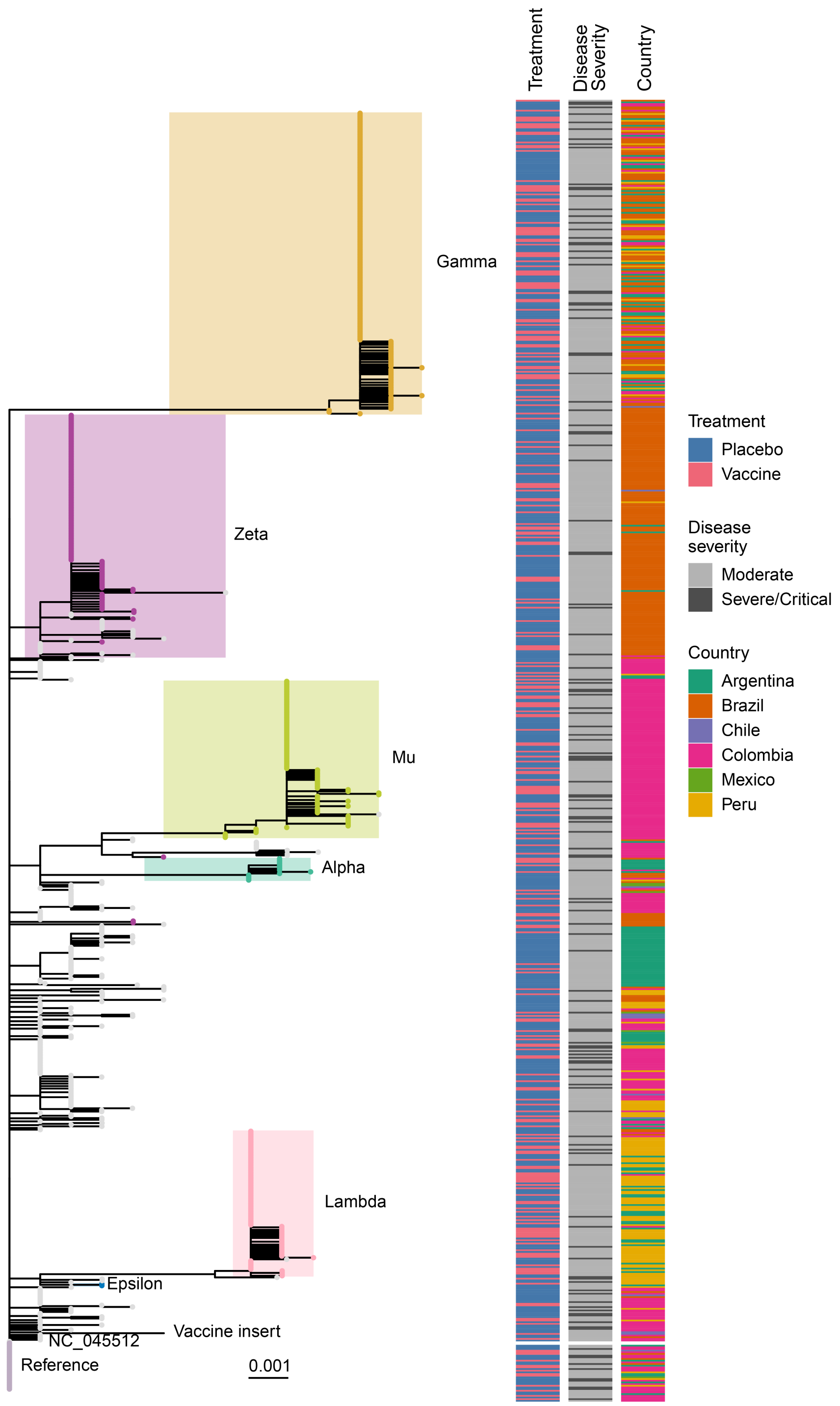

Supplementary Fig. 1
Phylogenetic tree based on the amino acid sequences from Latin America for the Spike protein. The lineages identified as WHO-labeled variants of concern/interest are indicated by the colored boxes, and the right-side colored bands (from left to right) indicate: (1) treatment assignment (vaccine vs. placebo); (2) disease severity (moderate vs. severe/critically ill); and (3) country. The tree is rooted to the Reference group and includes the reference sequence (NC_045512, https://www.ncbi.nlm.nih.gov/nuccore/1798174254) and the Ad26.COV2.S vaccine insert.

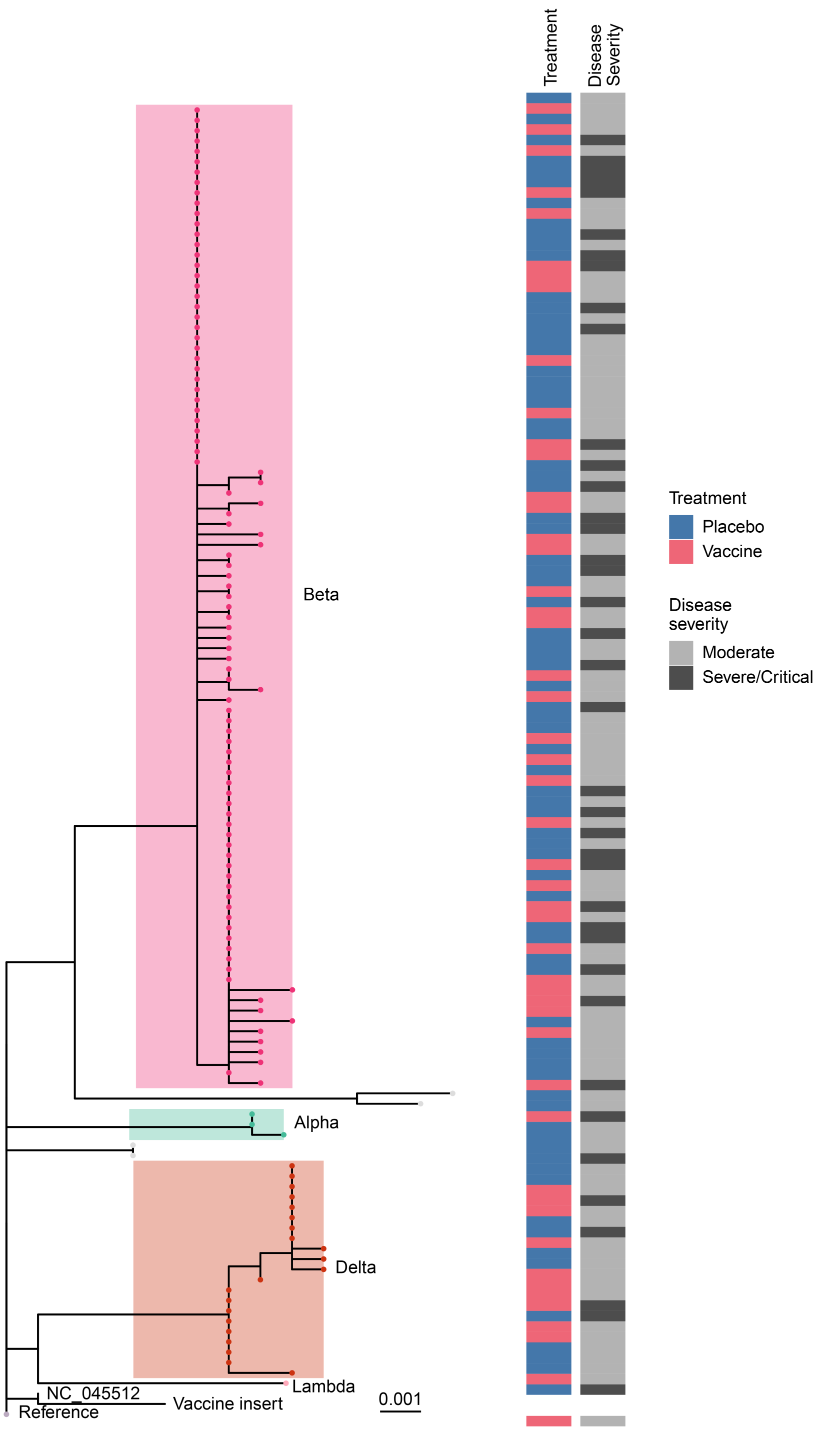

Supplementary Fig. 2
Phylogenetic tree based on the amino acid sequences from South Africa for the Spike protein. The lineages identified as WHO-labeled variants of concern/interest are indicated by the colored boxes, and the right-side colored bands (from left to right) indicate: (1) treatment assignment (vaccine vs. placebo) and (2) disease severity (moderate vs. severe/critically ill). The tree is rooted to the Reference group and includes the reference sequence (NC_045512, https://www.ncbi.nlm.nih.gov/nuccore/1798174254) and the Ad26.COV2.S vaccine insert.

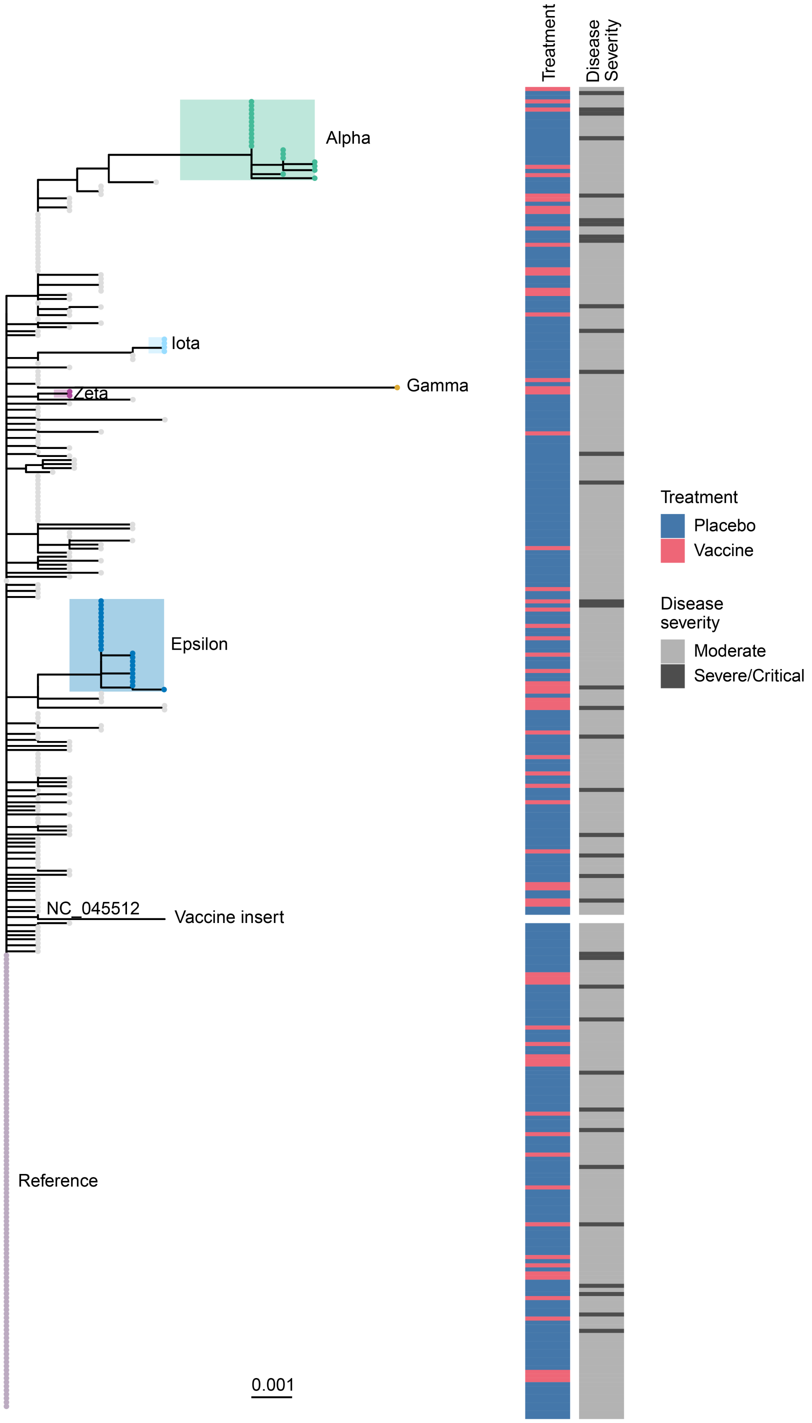

Supplementary Fig. 3

Phylogenetic tree based on the amino acid sequences from the US for the Spike protein. The lineages identified as WHO-labeled variants of concern/interest are indicated by the colored boxes, and the right-side colored bands (from left to right) indicate: (1) treatment assignment (vaccine vs. placebo) and (2) disease severity (moderate vs. severe/critically ill). The tree is rooted to the Reference group and includes the reference sequence (NC_045512, https://www.ncbi.nlm.nih.gov/nuccore/1798174254) and the Ad26.COV2.S vaccine insert.

Supplementary Table 6. Distribution of SARS-CoV-2 Spike amino acid sequences from participants who experienced the COVID-19 primary endpoint in the ENSEMBLE trial across countries for the vaccine and placebo groups, shown by lineage of the SARS-CoV-2 strain that caused the COVID-19 endpoint.

Treatment Arm	Lineage	Argentina	Brazil	Chile	Colombia	Mexico	Peru	Latin America Subtotal	South Africa	US	Overall Total
Ad26.COV2.S	Ref.*	15	12	3	33	2	7	72	1	52	125
	Alpha	4	0	0	0	0	0	4	1	4	9
	Beta	0	0	0	0	0	0	0	36	0	36
	Gamma	15	40	0	10	0	8	73	0	1	74
	Delta	0	0	0	0	0	0	0	11	0	11
	Zeta	1	31	1	0	0	0	33	0	1	34
	Iota	0	0	0	0	0	0	0	0	0	0
	Epsilon	0	0	0	0	0	0	0	0	8	8
	Lambda	8	0	0	1	0	34	43	0	0	43
	Mu	0	0	0	38	0	0	38	0	0	38
Ad26.COV2.S Subtotal		43	83	4	82	2	49	263	49	66	378
Placebo	Ref.*	42	38	6	83	5	22	196	4	221	421
	Alpha	3	4	0	2	0	1	10	2	16	28
	Beta	0	0	0	0	0	0	0	59	0	59
	Gamma	27	47	1	18	0	18	111	0	0	111
	Delta	0	0	0	0	0	0	0	10	0	10
	Zeta	2	89	0	0	0	1	92	0	1	93
	Iota	0	0	0	0	0	0	0	0	4	4
	Epsilon	0	0	1	1	0	0	2	0	15	17
	Lambda	14	0	0	1	0	30	45	1	0	46
	Mu	0	0	0	57	0	0	57	0	0	57
Placebo Subtotal		88	178	8	162	5	72	513	76	257	846
**Total**		**131**	**261**	**12**	**244**	**7**	**121**	**776**	**125**	**323**	**1224**

*Ref. = Reference.

Supplementary Table 7. Demographics of participants in the US in the sieve analysis cohort.

	Vaccine (N=93)	Placebo (N=323)	Total (N=416)
**Age**			
<60	66 (71.0%)	239 (74.0%)	305 (73.3%)
≥60	27 (29.0%)	84 (26.0%)	111 (26.7%)
Mean (Range)	47.5 (19, 77)	49.1 (19, 81)	48.8 (19, 81)
**Sex**			
Female	40 (43.0%)	150 (46.4%)	190 (45.7%)
Male	53 (57.0%)	173 (53.6%)	226 (54.3%)
**BMI**			
Underweight BMI < 18.5	-	4 (1.2%)	4 (1.0%)
Normal 18.5 ≤ BMI < 25	28 (30.1%)	84 (26.0%)	112 (26.9%)
Obese BMI ≥ 30	28 (30.1%)	91 (28.2%)	119 (28.6%)
Overweight 25 ≤ BMI < 30	37 (39.8%)	143 (44.3%)	180 (43.3%)
-	-	1 (0.3%)	1 (0.2%)
**Ethnicity**			
Hispanic Or Latino	16 (17.2%)	52 (16.1%)	68 (16.3%)
Not Hispanic Or Latino	73 (78.5%)	258 (79.9%)	331 (79.6%)
Unknown	4 (4.3%)	13 (4.0%)	17 (4.1%)
**Race**			
American Indian Or Alaska Native	2 (2.2%)	3 (0.9%)	5 (1.2%)
Asian	5 (5.4%)	14 (4.3%)	19 (4.6%)
Black Or African American	7 (7.5%)	29 (9.0%)	36 (8.7%)
Native Hawaiian Or Other Pacific Islander	-	1 (0.3%)	1 (0.2%)
White	76 (81.7%)	259 (80.2%)	335 (80.5%)
Multiple	-	3 (0.9%)	3 (0.7%)
Other	3 (3.2%)	14 (4.3%)	17 (4.1%)
**Country**			
US	93 (100.0%)	323 (100.0%)	416 (100.0%)
**Risk for Severe COVID-19***			
At-risk	38 (40.9%)	128 (39.6%)	166 (39.9%)
Not at-risk	55 (59.1%)	195 (60.4%)	250 (60.1%)
**Age, Risk for Severe COVID-19**			
Age < 60 At-risk	21 (22.6%)	84 (26.0%)	105 (25.2%)
Age < 60 Not at-risk	45 (48.4%)	155 (48.0%)	200 (48.1%)
Age ≥ 60 At-risk	17 (18.3%)	44 (13.6%)	61 (14.7%)
Age ≥ 60 Not at-risk	10 (10.8%)	40 (12.4%)	50 (12.0%)
**HIV Status**			
Negative	92 (98.9%)	323 (100.0%)	415 (99.8%)
Living with HIV	1 (1.1%)	-	1 (0.2%)

* "At-risk" is defined as having one or more comorbidities [listed in ref.[50]] associated with elevated risk of severe COVID-19.

Supplementary Table 8. Demographics of participants in South Africa in the sieve analysis cohort.

	Vaccine (N=62)	Placebo (N=110)	Total (N=172)
**Age**			
<60	54 (87.1%)	85 (77.3%)	139 (80.8%)
≥60	8 (12.9%)	25 (22.7%)	33 (19.2%)
Mean (Range)	44.7 (19, 68)	46.2 (19, 78)	45.6 (19, 78)
**Sex**			
Female	38 (61.3%)	66 (60.0%)	104 (60.5%)
Male	24 (38.7%)	44 (40.0%)	68 (39.5%)
**BMI**			
Underweight BMI < 18.5	1 (1.6%)	3 (2.7%)	4 (2.3%)
Normal 18.5 ≤ BMI < 25	15 (24.2%)	27 (24.5%)	42 (24.4%)
Obese BMI ≥ 30	28 (45.2%)	51 (46.4%)	79 (45.9%)
Overweight 25 ≤ BMI < 30	18 (29.0%)	29 (26.4%)	47 (27.3%)
**Ethnicity**			
Hispanic Or Latino	1 (1.6%)	1 (0.9%)	2 (1.2%)
Not Hispanic Or Latino	58 (93.5%)	105 (95.5%)	163 (94.8%)
Unknown	3 (4.8%)	4 (3.6%)	7 (4.1%)
**Race**			
Asian	2 (3.2%)	1 (0.9%)	3 (1.7%)
Black Or African American	43 (69.4%)	88 (80.0%)	131 (76.2%)
White	15 (24.2%)	16 (14.5%)	31 (18.0%)
Multiple	1 (1.6%)	3 (2.7%)	4 (2.3%)
Other	1 (1.6%)	2 (1.8%)	3 (1.7%)
**Country**			
South Africa	62 (100.0%)	110 (100.0%)	172 (100.0%)
**Risk for Severe COVID-19***			
At-risk	37 (59.7%)	63 (57.3%)	100 (58.1%)
Not at-risk	25 (40.3%)	47 (42.7%)	72 (41.9%)
**Age, Risk for Severe COVID-19**			
Age < 60 At-risk	32 (51.6%)	43 (39.1%)	75 (43.6%)
Age < 60 Not at-risk	22 (35.5%)	42 (38.2%)	64 (37.2%)
Age ≥ 60 At-risk	5 (8.1%)	20 (18.2%)	25 (14.5%)
Age ≥ 60 Not at-risk	3 (4.8%)	5 (4.5%)	8 (4.7%)
**HIV Status**			
Negative	55 (88.7%)	105 (95.5%)	160 (93.0%)
Living with HIV	7 (11.3%)	5 (4.5%)	12 (7.0%)

* "At-risk" is defined as having one or more comorbidities [listed in ref.[49]] associated with elevated risk of severe COVID-19.

Supplementary Table 9. Demographics of participants across all regions pooled in the sieve analysis cohort.

	Vaccine (N=484)	Placebo (N=1067)	Total (N=1551)
**Age**			
<60	381 (78.7%)	847 (79.4%)	1228 (79.2%)
≥60	103 (21.3%)	220 (20.6%)	323 (20.8%)
Mean (Range)	44.3 (18, 83)	46.0 (18, 83)	45.5 (18, 83)
**Sex**			
Female	217 (44.8%)	486 (45.5%)	703 (45.3%)
Male	267 (55.2%)	581 (54.5%)	848 (54.7%)
**BMI**			
Underweight BMI < 18.5	6 (1.2%)	10 (0.9%)	16 (1.0%)
Normal 18.5 ≤ BMI < 25	146 (30.2%)	305 (28.6%)	451 (29.1%)
Obese BMI ≥ 30	128 (26.4%)	296 (27.7%)	424 (27.3%)
Overweight 25 ≤ BMI < 30	204 (42.1%)	454 (42.5%)	658 (42.4%)
-	-	2 (0.2%)	2 (0.1%)
**Ethnicity**			
Hispanic Or Latino	336 (69.4%)	664 (62.2%)	1000 (64.5%)
Not Hispanic Or Latino	137 (28.3%)	382 (35.8%)	519 (33.5%)
Unknown	11 (2.3%)	21 (2.0%)	32 (2.1%)
**Race**			
American Indian Or Alaska Native	103 (21.3%)	190 (17.8%)	293 (18.9%)
Asian	12 (2.5%)	17 (1.6%)	29 (1.9%)
Black Or African American	62 (12.8%)	139 (13.0%)	201 (13.0%)
Native Hawaiian Or Other Pacific Islander	-	1 (0.1%)	1 (0.1%)
White	237 (49.0%)	574 (53.8%)	811 (52.3%)
Multiple	51 (10.5%)	110 (10.3%)	161 (10.4%)
Other	19 (3.9%)	36 (3.4%)	55 (3.5%)
**Country**			
Argentina	45 (9.3%)	102 (9.6%)	147 (9.5%)
Brazil	109 (22.5%)	212 (19.9%)	321 (20.7%)
Chile	6 (1.2%)	9 (0.8%)	15 (1.0%)
Colombia	105 (21.7%)	209 (19.6%)	314 (20.2%)
Mexico	3 (0.6%)	9 (0.8%)	12 (0.8%)
Peru	61 (12.6%)	93 (8.7%)	154 (9.9%)
South Africa	62 (12.8%)	110 (10.3%)	172 (11.1%)
US	93 (19.2%)	323 (30.3%)	416 (26.8%)
**Risk for Severe COVID-19***			
At-risk	189 (39.0%)	402 (37.7%)	591 (38.1%)
Not at-risk	295 (61.0%)	665 (62.3%)	960 (61.9%)
**Age, Risk for Severe COVID-19**			
Age < 60 At-risk	128 (26.4%)	279 (26.1%)	407 (26.2%)
Age < 60 Not at-risk	253 (52.3%)	568 (53.2%)	821 (52.9%)
Age ≥ 60 At-risk	61 (12.6%)	123 (11.5%)	184 (11.9%)
Age ≥ 60 Not at-risk	42 (8.7%)	97 (9.1%)	139 (9.0%)
**HIV Status**			
Negative	471 (97.3%)	1051 (98.5%)	1522 (98.1%)
Living with HIV	13 (2.7%)	16 (1.5%)	29 (1.9%)

* "At-risk" is defined as having one or more comorbidities [listed in ref.[49]] associated with elevated risk of severe COVID-19.

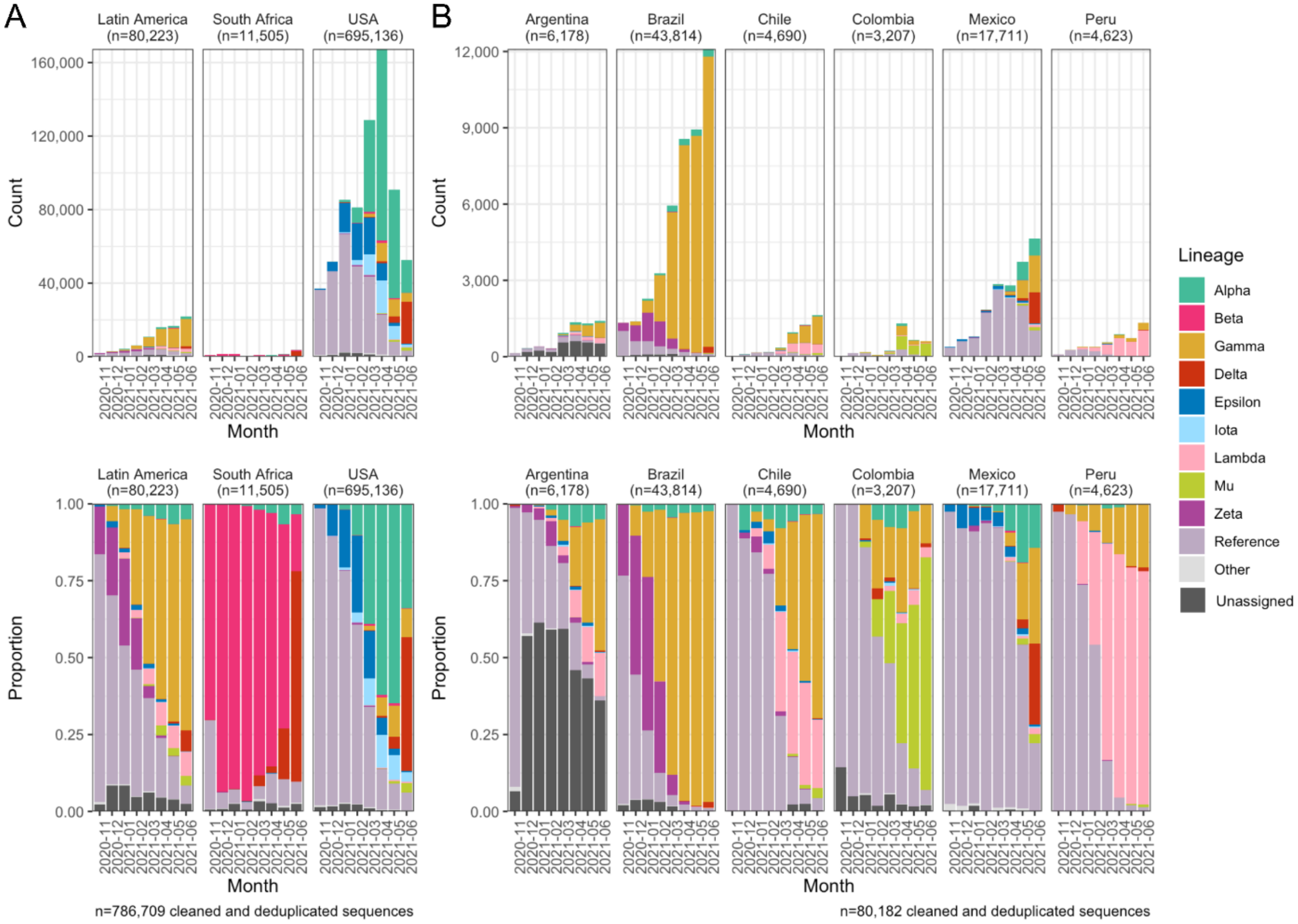

Supplementary Fig. 4
Distribution of SARS-CoV-2 variants based on data available on GISAID. All sequences sampled from countries where SARS-CoV-2 infecting viruses were sequenced during the ENSEMBLE study period were downloaded as of 11th August 2021. Sequences were de-duplicated by sequence name, and variant sequences with dates prior to emergence (i.e., having dates that were likely mis-entered) were removed. A full list of sequences and their attributions is accessible via https://doi.org/10.55876/gis8.230108me. (A) Distribution of variants in Latin America (n=80,223), South Africa (n=11,505) and the US (n=695,136). (B) Distribution of variants in six Latin America countries: Argentina (n=6,178), Brazil (n=43,814), Chile (n=4,690), Colombia (n=3,207), Mexico (n=17,711) and Peru (n=4,623).

A) Argentina

Variant	No. of Cases (V vs. P) (Incidence per 100 PYRs)	VE (%) (95% CI)
Reference	15 (2.7) vs. 42 (7.8)	68.4 (43.6, 82.3)
Gamma	15 (2.7) vs. 27 (5)	54.8 (15.8, 75.7)
Lambda	8 (1.4) vs. 14 (2.6)	52.3 (−15.1, 80.2)

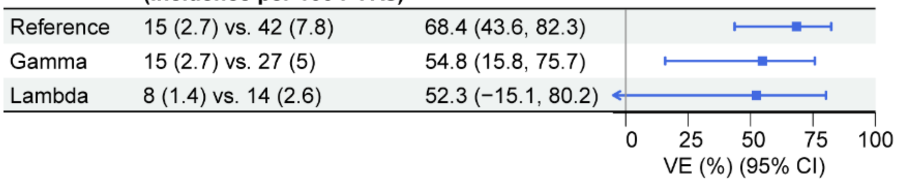

B) Brazil

Variant	No. of Cases (V vs. P) (Incidence per 100 PYRs)	VE (%) (95% CI)
Reference	12 (1.2) vs. 38 (4)	65.8 (35.6, 81.9)
Gamma	40 (4.1) vs. 47 (4.9)	14.1 (−26.8, 41.8)
Zeta	31 (3.2) vs. 89 (9.3)	65.6 (49.2, 76.7)

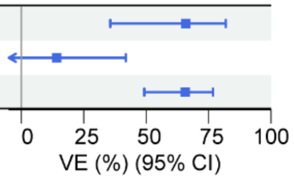

C) Chile and Peru

Variant	No. of Cases (V vs. P) (Incidence per 100 PYRs)	VE (%) (95% CI)
Reference	10 (2.3) vs. 28 (6.8)	63.2 (26.3, 81.7)
Gamma	8 (1.9) vs. 19 (4.6)	65.1 (15.9, 85.5)
Lambda	34 (8) vs. 30 (7.2)	−2.7 (−64.4, 35.9)

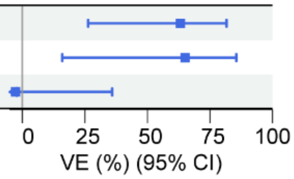

D) Colombia

Variant	No. of Cases (V vs. P) (Incidence per 100 PYRs)	VE (%) (95% CI)
Reference	33 (4.2) vs. 83 (10.9)	65.4 (49.4, 76.3)
Gamma	10 (1.3) vs. 18 (2.4)	43.6 (−21.0, 73.7)
Mu	38 (4.8) vs. 57 (7.5)	39.2 (9.5, 59.2)

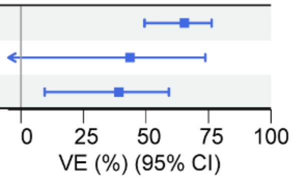

Supplementary Fig. 5
For (A) Argentina, (B) Brazil, (C) Chile and Peru, and (D) Colombia, vaccine efficacy (VE) estimates against the primary COVID-19 endpoint caused by SARS-CoV-2 lineages (lineage "X"). Point estimates are shown with a filled square and horizontal bars extend through the 95% confidence interval (CI). To avoid posthoc revision of the SAP, p values were not calculated for the country-specific analyses.

A) Argentina

Variant	No. of Cases (V vs. P) (Incidence per 100 PYRs)	VE (%) (95% CI)
Not Gamma	28 (5) vs. 61 (11.4)	60.0 (38.2, 74.1)
Not Gamma	28 (5) vs. 61 (11.4)	60.0 (38.2, 74.1)
Not Lambda	35 (6.3) vs. 74 (13.8)	59.5 (40.3, 72.5)

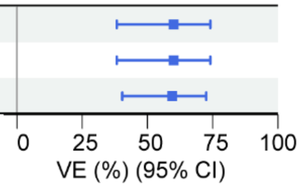

B) Brazil

Variant	No. of Cases (V vs. P) (Incidence per 100 PYRs)	VE (%) (95% CI)
Not Gamma	43 (4.4) vs. 131 (13.7)	66.5 (53.7, 75.8)
Not Gamma	43 (4.4) vs. 131 (13.7)	66.5 (53.7, 75.8)
Not Zeta	52 (5.3) vs. 89 (9.3)	38.1 (14.7, 55.1)

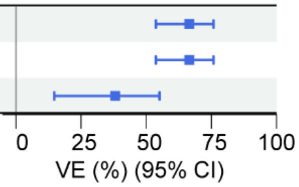

C) Chile and Peru

Variant	No. of Cases (V vs. P) (Incidence per 100 PYRs)	VE (%) (95% CI)
Not Gamma	45 (10.6) vs. 61 (14.7)	31.5 (1.6, 52.4)
Not Gamma	45 (10.6) vs. 61 (14.7)	31.5 (1.6, 52.4)
Not Lambda	19 (4.5) vs. 50 (12.1)	64.8 (41.2, 78.9)

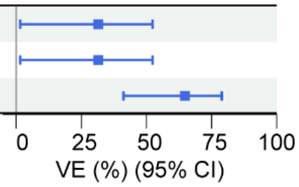

D) Colombia

Variant	No. of Cases (V vs. P) (Incidence per 100 PYRs)	VE (%) (95% CI)
Not Gamma	72 (9.2) vs. 144 (19)	55.6 (42.1, 65.9)
Not Gamma	72 (9.2) vs. 144 (19)	55.6 (42.1, 65.9)
Not Mu	44 (5.6) vs. 105 (13.8)	62.0 (47.1, 72.7)

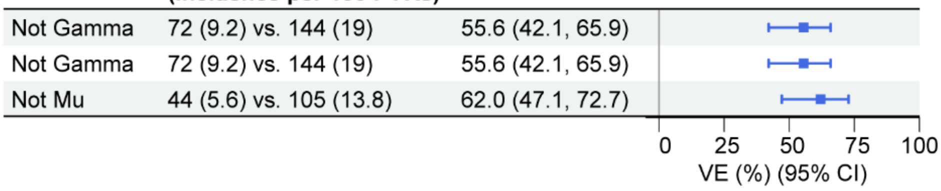

Supplementary Fig. 6
For (A) Argentina, (B) Brazil, (C) Chile and Peru, and (D) Colombia, vaccine efficacy (VE) estimates against the primary COVID-19 endpoint caused by all other lineages combined ("Not X"). Point estimates are shown with a filled square and horizontal bars extend through the 95% confidence interval (CI). To avoid posthoc revision of the SAP, p values were not calculated for the country-specific analyses.

A) Argentina

Comparison	Differential VE (95% CI)
Reference vs. Gamma	1.43 (0.61, 3.36)
Reference vs. Lambda	1.51 (0.52, 4.35)
Gamma vs. Lambda	1.06 (0.35, 3.16)
Reference vs. Not Reference	1.59 (0.76, 3.33)
Gamma vs. Not Gamma	0.88 (0.41, 1.92)
Lambda vs. Not Lambda	0.85 (0.32, 2.25)

B) Brazil

Comparison	Differential VE (95% CI)
Reference vs. Gamma	2.51 (1.19, 5.29)
Reference vs. Zeta	1.01 (0.47, 2.14)
Gamma vs. Zeta	0.40 (0.23, 0.70)
Reference vs. Not Reference	1.51 (0.75, 3.01)
Gamma vs. Not Gamma	0.39 (0.23, 0.65)
Zeta vs. Not Zeta	1.80 (1.08, 3.00)

C) Chile and Peru

Comparison	Differential VE (95% CI)
Reference vs. Gamma	0.95 (0.30, 3.01)
Reference vs. Lambda	2.79 (1.19, 6.56)
Gamma vs. Lambda	2.95 (1.06, 8.17)
Reference vs. Not Reference	1.98 (0.87, 4.47)
Gamma vs. Not Gamma	1.95 (0.73, 5.20)
Lambda vs. Not Lambda	0.34 (0.16, 0.69)

D) Colombia

Comparison	Differential VE (95% CI)
Reference vs. Gamma	1.63 (0.69, 3.83)
Reference vs. Mu	1.76 (1.01, 3.05)
Gamma vs. Mu	1.08 (0.44, 2.62)
Reference vs. Not Reference	1.68 (1.01, 2.80)
Gamma vs. Not Gamma	0.79 (0.35, 1.80)
Mu vs. Not Mu	0.62 (0.37, 1.06)

Supplementary Fig. 7

For (A) Argentina, (B) Brazil, (C) Chile and Peru, and (D) Colombia, differential vaccine efficacy (VE) estimates against the primary COVID-19 endpoint across pairs of lineages or across a lineage (“X”) vs. all other lineages (“Not X”). To avoid posthoc revision of the SAP, p values were not calculated for the country-specific analyses. Differential VE for genotype 1 vs. genotype 2, with VE(genotype 1) >= VE(genotype 2), is calculated as DVE = [1-VE(genotype 2)]/[1-VE(genotype 1)], with interpretation that vaccine protection is DVE-fold better against genotype 1 than against genotype 2.

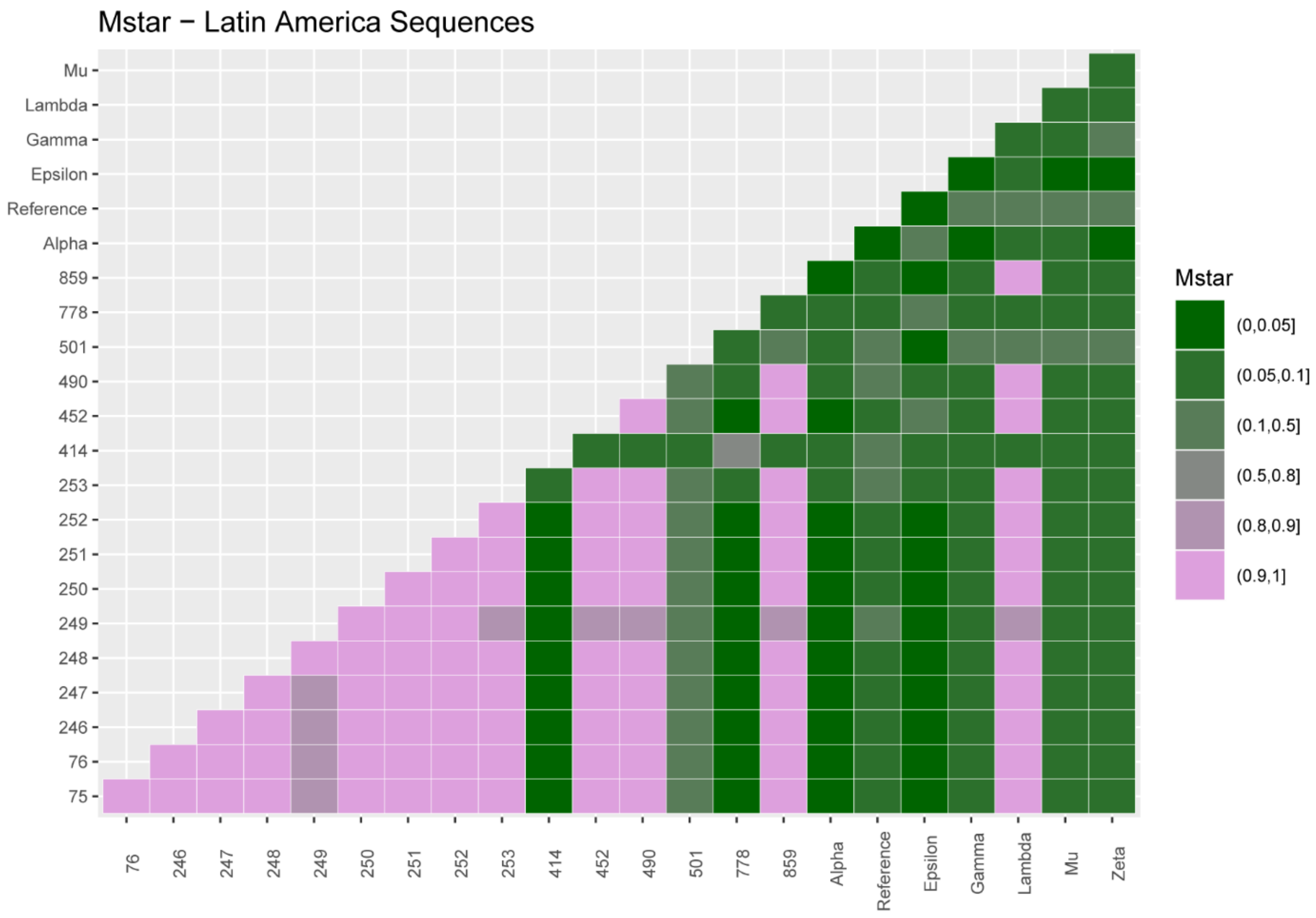

Supplementary Fig. 8

Covariability of all pairs of AA positions among the 16 sieve signature sites shown in **Fig. 2e** and with the indicators of each major lineage (vs. all other lineages) circulating in Latin America. The Mstar statistic quantifying covariability[1] takes value 0 for zero covariability and 1 for perfect covariability; pink cells have Mstar > 0.9 indicating very high covariability. Site 501 is unique, in that it nearly perfectly covaries across the set of lineages but is not detected as highly covarying with any single lineage in this heatmap because the analysis calculated covariability using dichotomous variables of lineage vs. all other lineages combined. Generated in R[50] (version 3.6.3).

Argentina

Spike Amino Acid Position	No. of Cases (V vs. P) (Incidence per 100 PYRs)	VE (%) (95% CI)
18		
Match	28 (5) vs. 61 (11.4)	60.0 (38.2, 74.1)
Mismatch	15 (2.7) vs. 27 (5)	54.8 (15.8, 75.7)
20		
Match	28 (5) vs. 61 (11.4)	60.0 (38.2, 74.1)
Mismatch	15 (2.7) vs. 27 (5)	54.8 (15.8, 75.7)
26		
Match	28 (5) vs. 61 (11.4)	60.0 (38.2, 74.1)
Mismatch	15 (2.7) vs. 27 (5)	54.8 (15.8, 75.7)
75		
Match	34 (6.1) vs. 74 (13.8)	60.6 (41.7, 73.4)
Mismatch	9 (1.6) vs. 14 (2.6)	46.3 (−25.3, 77.0)
76		
Match	34 (6.1) vs. 74 (13.8)	60.6 (41.7, 73.4)
Mismatch	9 (1.6) vs. 14 (2.6)	46.3 (−25.3, 77.0)
138		
Match	28 (5) vs. 61 (11.4)	60.0 (38.2, 74.1)
Mismatch	15 (2.7) vs. 27 (5)	54.8 (15.8, 75.7)
190		
Match	28 (5) vs. 61 (11.4)	60.0 (38.2, 74.1)
Mismatch	15 (2.7) vs. 27 (5)	54.8 (15.8, 75.7)
246		
Match	34 (6.1) vs. 74 (13.8)	60.6 (41.7, 73.4)
Mismatch	9 (1.6) vs. 14 (2.6)	46.3 (−25.3, 77.0)
247		
Match	35 (6.3) vs. 74 (13.8)	59.5 (40.3, 72.5)
Mismatch	8 (1.4) vs. 14 (2.6)	52.3 (−15.1, 80.2)
248		
Match	35 (6.3) vs. 74 (13.8)	59.5 (40.3, 72.5)
Mismatch	8 (1.4) vs. 14 (2.6)	52.3 (−15.1, 80.2)
249		
Match	35 (6.3) vs. 74 (13.8)	59.5 (40.3, 72.5)
Mismatch	8 (1.4) vs. 14 (2.6)	52.3 (−15.1, 80.2)
250		
Match	35 (6.3) vs. 74 (13.8)	59.5 (40.3, 72.5)
Mismatch	8 (1.4) vs. 14 (2.6)	52.3 (−15.1, 80.2)
251		
Match	35 (6.3) vs. 74 (13.8)	59.5 (40.3, 72.5)
Mismatch	8 (1.4) vs. 14 (2.6)	52.3 (−15.1, 80.2)
252		
Match	35 (6.3) vs. 74 (13.8)	59.5 (40.3, 72.5)
Mismatch	8 (1.4) vs. 14 (2.6)	52.3 (−15.1, 80.2)
253		
Match	34 (6.1) vs. 74 (13.8)	60.7 (41.9, 73.5)
Mismatch	9 (1.6) vs. 14 (2.6)	45.9 (−26.0, 76.7)
414		
Match	39 (7) vs. 57 (10.6)	43.3 (16.0, 61.7)
Mismatch	4 (0.7) vs. 31 (5.8)	89.2 (66.2, 96.6)
417		
Match	28 (5) vs. 61 (11.4)	60.0 (38.2, 74.1)
Mismatch	15 (2.7) vs. 27 (5)	54.8 (15.8, 75.7)
452		
Match	34 (6.1) vs. 74 (13.8)	60.6 (41.7, 73.4)
Mismatch	9 (1.6) vs. 14 (2.6)	46.3 (−25.3, 77.0)
484		
Match	27 (4.9) vs. 58 (10.8)	59.2 (36.3, 73.9)
Mismatch	16 (2.9) vs. 30 (5.6)	56.8 (21.7, 76.2)
490		
Match	34 (6.1) vs. 74 (13.8)	60.6 (41.7, 73.4)
Mismatch	9 (1.6) vs. 14 (2.6)	46.3 (−25.3, 77.0)
501		
Match	24 (4.3) vs. 58 (10.8)	64.3 (43.0, 77.6)
Mismatch	19 (3.4) vs. 30 (5.6)	47.5 (8.4, 69.9)
655		
Match	28 (5) vs. 61 (11.4)	60.0 (38.2, 74.1)
Mismatch	15 (2.7) vs. 27 (5)	54.8 (15.8, 75.7)
778		
Match	42 (7.5) vs. 68 (12.7)	48.0 (24.7, 64.1)
Mismatch	1 (0.2) vs. 20 (3.7)	96.6 (47.8, 99.8)
859		
Match	34 (6.1) vs. 73 (13.6)	60.1 (40.9, 73.1)
Mismatch	9 (1.6) vs. 15 (2.8)	49.9 (−15.7, 78.3)
1027		
Match	28 (5) vs. 61 (11.4)	60.0 (38.2, 74.1)
Mismatch	15 (2.7) vs. 27 (5)	54.8 (15.8, 75.7)
1176		
Match	27 (4.9) vs. 59 (11)	59.9 (37.4, 74.3)
Mismatch	16 (2.9) vs. 29 (5.4)	55.6 (19.0, 75.6)

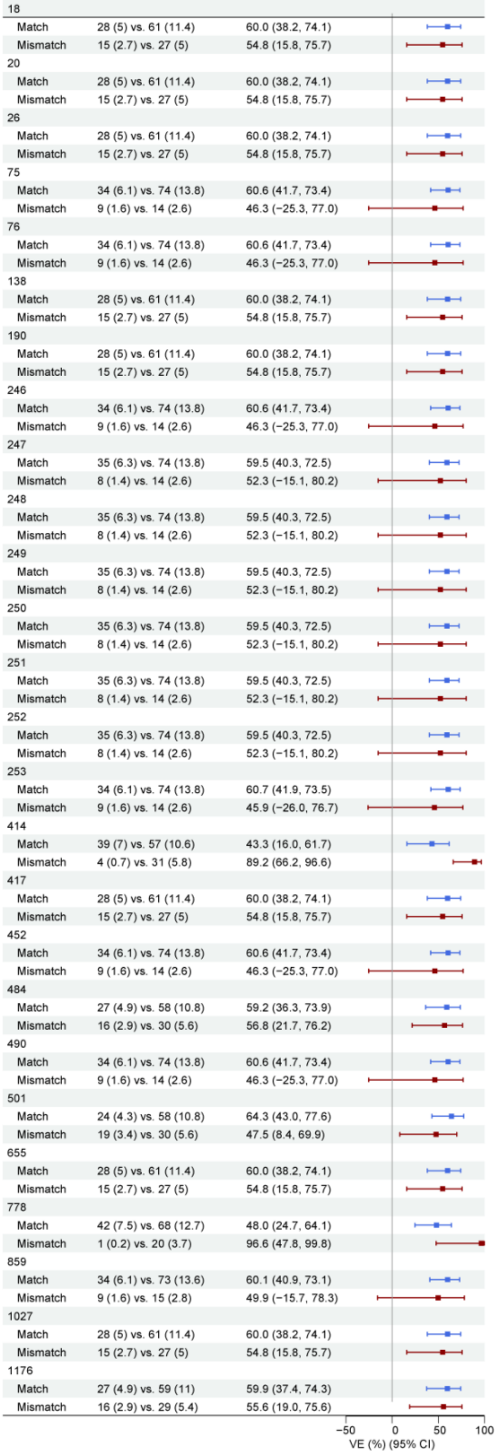

Supplementary Fig. 9

For Argentina, differential vaccine efficacy (VE) estimates against the primary COVID-19 endpoint caused by SARS-CoV-2 with a vaccine-matched (blue) or vaccine-mismatched (maroon) residue for all screened-in residues. Point estimates are shown with a filled square and horizontal bars extend through the 95% confidence interval (CI). PYRs, person-years.

Brazil

Spike Amino Acid Position	No. of Cases (V vs. P) (Incidence per 100 PYRs)	VE (%) (95% CI)
18		
Match	43 (4.4) vs. 130 (13.6)	66.3 (53.3, 75.7)
Mismatch	40 (4.1) vs. 48 (5)	15.8 (−24.0, 42.9)
20		
Match	44 (4.5) vs. 132 (13.8)	66.0 (53.1, 75.4)
Mismatch	39 (4) vs. 46 (4.8)	14.6 (−26.6, 42.4)
26		
Match	43 (4.4) vs. 131 (13.7)	66.5 (53.7, 75.8)
Mismatch	40 (4.1) vs. 47 (4.9)	14.1 (−26.8, 41.8)
138		
Match	42 (4.3) vs. 129 (13.5)	66.8 (53.8, 76.1)
Mismatch	41 (4.2) vs. 49 (5.1)	15.7 (−23.7, 42.5)
190		
Match	43 (4.4) vs. 131 (13.7)	66.5 (53.7, 75.8)
Mismatch	40 (4.1) vs. 47 (4.9)	14.1 (−26.8, 41.8)
417		
Match	43 (4.4) vs. 131 (13.7)	66.5 (53.7, 75.8)
Mismatch	40 (4.1) vs. 47 (4.9)	14.1 (−26.8, 41.8)
484		
Match	9 (0.9) vs. 38 (4)	73.6 (47.3, 86.8)
Mismatch	74 (7.5) vs. 140 (14.6)	46.6 (30.5, 58.9)
501		
Match	43 (4.4) vs. 127 (13.2)	65.7 (52.4, 75.3)
Mismatch	40 (4.1) vs. 51 (5.3)	19.9 (−17.4, 45.3)
655		
Match	42 (4.3) vs. 130 (13.6)	67.1 (54.3, 76.3)
Mismatch	41 (4.2) vs. 48 (5)	13.8 (−26.7, 41.4)
1027		
Match	43 (4.4) vs. 131 (13.7)	66.5 (53.7, 75.8)
Mismatch	40 (4.1) vs. 47 (4.9)	14.1 (−26.8, 41.8)
1176		
Match	6 (0.6) vs. 16 (1.7)	58.1 (−5.0, 83.3)
Mismatch	77 (7.8) vs. 162 (16.9)	51.4 (37.3, 62.3)

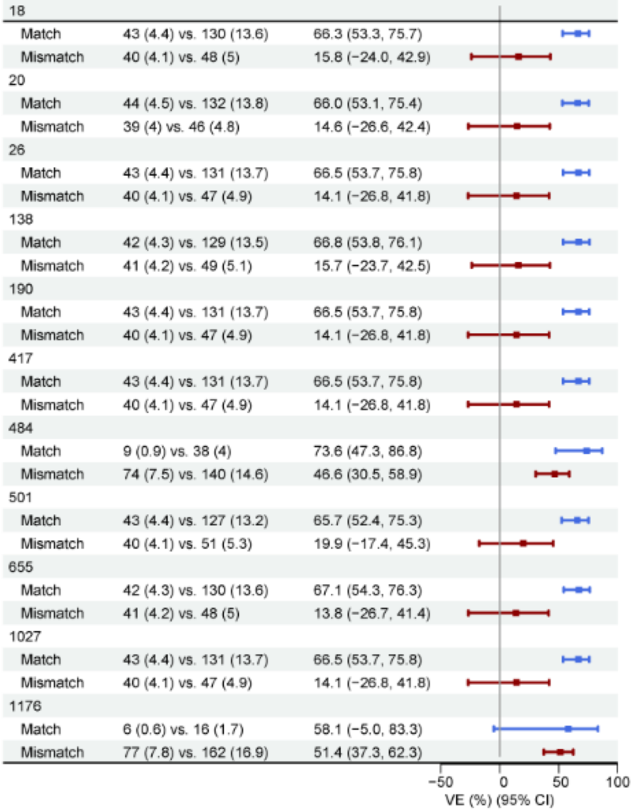

Supplementary Fig. 10
For Brazil, differential vaccine efficacy (VE) estimates against the primary COVID-19 endpoint caused by SARS-CoV-2 with a vaccine-matched (blue) or vaccine-mismatched (maroon) residue for all screened-in residues. Point estimates are shown with a filled square and horizontal bars extend through the 95% confidence interval (CI). PYRs, person-years.

Chile and Peru

Spike Amino Acid Position	No. of Cases (V vs. P) (Incidence per 100 PYRs)	VE (%) (95% CI)
18		
Match	45 (10.6) vs. 60 (14.5)	30.3 (−0.4, 51.6)
Mismatch	8 (1.9) vs. 20 (4.8)	66.5 (20.2, 86.0)
20		
Match	45 (10.6) vs. 62 (15)	32.5 (3.1, 53.0)
Mismatch	8 (1.9) vs. 18 (4.3)	63.3 (10.6, 84.9)
26		
Match	45 (10.6) vs. 61 (14.7)	31.5 (1.6, 52.4)
Mismatch	8 (1.9) vs. 19 (4.6)	64.9 (15.1, 85.5)
75		
Match	19 (4.5) vs. 49 (11.8)	63.9 (39.8, 78.4)
Mismatch	34 (8) vs. 31 (7.5)	−0.9 (−61.4, 37.0)
76		
Match	19 (4.5) vs. 49 (11.8)	63.9 (39.8, 78.4)
Mismatch	34 (8) vs. 31 (7.5)	−0.9 (−61.4, 37.0)
138		
Match	44 (10.3) vs. 59 (14.2)	31.1 (0.3, 52.5)
Mismatch	9 (2.1) vs. 21 (5.1)	62.3 (15.7, 83.2)
190		
Match	45 (10.6) vs. 61 (14.7)	31.5 (1.6, 52.4)
Mismatch	8 (1.9) vs. 19 (4.6)	64.9 (15.1, 85.5)
246		
Match	19 (4.5) vs. 48 (11.6)	63.0 (38.2, 77.9)
Mismatch	34 (8) vs. 32 (7.7)	3.0 (−54.4, 39.1)
247		
Match	19 (4.5) vs. 49 (11.8)	63.9 (39.8, 78.4)
Mismatch	34 (8) vs. 31 (7.5)	−0.9 (−61.4, 37.0)
248		
Match	19 (4.5) vs. 49 (11.8)	63.9 (39.8, 78.4)
Mismatch	34 (8) vs. 31 (7.5)	−0.9 (−61.4, 37.0)
249		
Match	19 (4.5) vs. 49 (11.8)	63.9 (39.8, 78.4)
Mismatch	34 (8) vs. 31 (7.5)	−0.9 (−61.4, 37.0)
250		
Match	19 (4.5) vs. 49 (11.8)	63.9 (39.8, 78.4)
Mismatch	34 (8) vs. 31 (7.5)	−0.9 (−61.4, 37.0)
251		
Match	19 (4.5) vs. 49 (11.8)	63.9 (39.8, 78.4)
Mismatch	34 (8) vs. 31 (7.5)	−0.9 (−61.4, 37.0)
252		
Match	19 (4.5) vs. 49 (11.8)	63.9 (39.8, 78.4)
Mismatch	34 (8) vs. 31 (7.5)	−0.9 (−61.4, 37.0)
253		
Match	19 (4.5) vs. 49 (11.8)	63.9 (39.8, 78.4)
Mismatch	34 (8) vs. 31 (7.5)	−0.9 (−61.4, 37.0)
417		
Match	45 (10.6) vs. 61 (14.7)	31.5 (1.6, 52.4)
Mismatch	8 (1.9) vs. 19 (4.6)	64.9 (15.1, 85.5)
452		
Match	19 (4.5) vs. 48 (11.6)	62.8 (38.0, 77.7)
Mismatch	34 (8) vs. 32 (7.7)	3.2 (−54.2, 39.3)
484		
Match	44 (10.3) vs. 60 (14.5)	32.2 (2.1, 53.0)
Mismatch	9 (2.1) vs. 20 (4.8)	61.1 (12.3, 82.7)
490		
Match	19 (4.5) vs. 50 (12.1)	64.8 (41.2, 78.9)
Mismatch	34 (8) vs. 30 (7.2)	−4.4 (−67.7, 34.9)
501		
Match	45 (10.6) vs. 60 (14.5)	30.2 (−0.5, 51.5)
Mismatch	8 (1.9) vs. 20 (4.8)	67.0 (20.1, 86.3)
655		
Match	45 (10.6) vs. 58 (14)	28.4 (−3.4, 50.4)
Mismatch	8 (1.9) vs. 22 (5.3)	69.1 (26.3, 87.0)
859		
Match	19 (4.5) vs. 49 (11.8)	63.9 (39.8, 78.4)
Mismatch	34 (8) vs. 31 (7.5)	−0.9 (−61.4, 37.0)
1027		
Match	45 (10.6) vs. 61 (14.7)	31.5 (1.6, 52.4)
Mismatch	8 (1.9) vs. 19 (4.6)	64.9 (15.1, 85.5)
1176		
Match	44 (10.3) vs. 60 (14.5)	32.2 (2.1, 53.0)
Mismatch	9 (2.1) vs. 20 (4.8)	61.1 (12.3, 82.7)

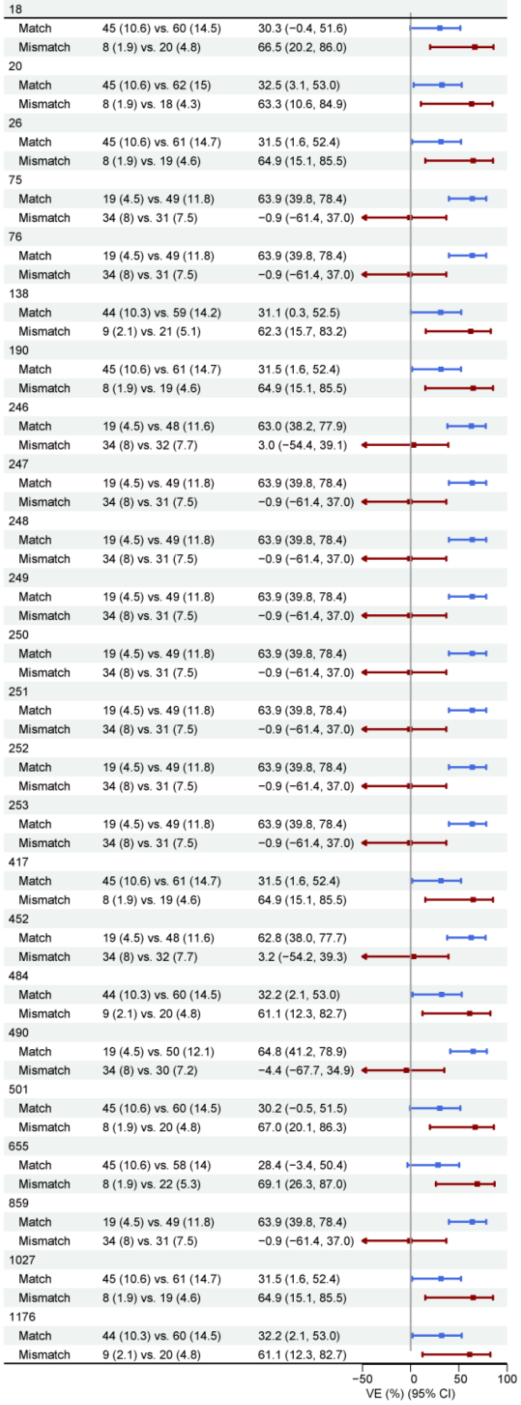

Supplementary Fig. 11

For Chile + Peru, differential vaccine efficacy (VE) estimates against the primary COVID-19 endpoint caused by SARS-CoV-2 with a vaccine-matched (blue) or vaccine-mismatched (maroon) residue for all screened-in residues. Point estimates are shown with a filled square and horizontal bars extend through the 95% confidence interval (CI). PYRs, person-years.

Colombia

Spike Amino Acid Position	No. of Cases (V vs. P) (Incidence per 100 PYRs)	VE (%) (95% CI)
18		
Match	69 (8.8) vs. 142 (18.7)	56.9 (43.6, 67.1)
Mismatch	13 (1.7) vs. 20 (2.6)	34.5 (−31.6, 67.4)
20		
Match	71 (9) vs. 144 (19)	56.1 (42.7, 66.4)
Mismatch	11 (1.4) vs. 18 (2.4)	39.6 (−26.6, 71.2)
26		
Match	72 (9.2) vs. 144 (19)	55.6 (42.1, 65.9)
Mismatch	10 (1.3) vs. 18 (2.4)	43.6 (−21.0, 73.7)
95		
Match	41 (5.2) vs. 98 (12.9)	62.2 (46.8, 73.2)
Mismatch	41 (5.2) vs. 64 (8.4)	41.4 (14.6, 59.8)
138		
Match	72 (9.2) vs. 144 (19)	55.6 (42.1, 65.9)
Mismatch	10 (1.3) vs. 18 (2.4)	43.6 (−21.0, 73.7)
144		
Match	40 (5.1) vs. 93 (12.3)	61.3 (45.1, 72.7)
Mismatch	42 (5.3) vs. 69 (9.1)	44.3 (19.5, 61.5)
145		
Match	45 (5.7) vs. 108 (14.2)	62.5 (47.9, 73.0)
Mismatch	37 (4.7) vs. 54 (7.1)	37.1 (5.7, 58.1)
145a		
Match	45 (5.7) vs. 109 (14.4)	62.8 (48.4, 73.2)
Mismatch	37 (4.7) vs. 53 (7)	35.9 (3.8, 57.3)
190		
Match	72 (9.2) vs. 144 (19)	55.6 (42.1, 65.9)
Mismatch	10 (1.3) vs. 18 (2.4)	43.6 (−21.0, 73.7)
346		
Match	43 (5.5) vs. 105 (13.8)	62.9 (48.2, 73.4)
Mismatch	39 (5) vs. 57 (7.5)	37.5 (7.4, 57.9)
417		
Match	72 (9.2) vs. 144 (19)	55.6 (42.1, 65.9)
Mismatch	10 (1.3) vs. 18 (2.4)	43.6 (−21.0, 73.7)
484		
Match	28 (3.6) vs. 71 (9.4)	65.5 (48.0, 77.1)
Mismatch	54 (6.9) vs. 91 (12)	45.0 (24.7, 59.9)
501		
Match	33 (4.2) vs. 85 (11.2)	66.0 (50.5, 76.7)
Mismatch	49 (6.2) vs. 77 (10.2)	40.5 (16.7, 57.5)
655		
Match	72 (9.2) vs. 144 (19)	55.6 (42.1, 65.9)
Mismatch	10 (1.3) vs. 18 (2.4)	43.6 (−21.0, 73.7)
681		
Match	43 (5.5) vs. 102 (13.5)	62.0 (46.8, 72.8)
Mismatch	39 (5) vs. 60 (7.9)	40.4 (12.0, 59.6)
950		
Match	42 (5.3) vs. 98 (12.9)	61.3 (45.7, 72.5)
Mismatch	40 (5.1) vs. 64 (8.4)	42.9 (16.5, 61.0)
1027		
Match	72 (9.2) vs. 144 (19)	55.6 (42.1, 65.9)
Mismatch	10 (1.3) vs. 18 (2.4)	43.6 (−21.0, 73.7)
1167		
Match	73 (9.3) vs. 140 (18.5)	52.7 (38.5, 63.6)
Mismatch	9 (1.1) vs. 22 (2.9)	63.9 (23.7, 82.9)
1176		
Match	72 (9.2) vs. 144 (19)	55.6 (42.1, 65.9)
Mismatch	10 (1.3) vs. 18 (2.4)	43.6 (−21.0, 73.7)

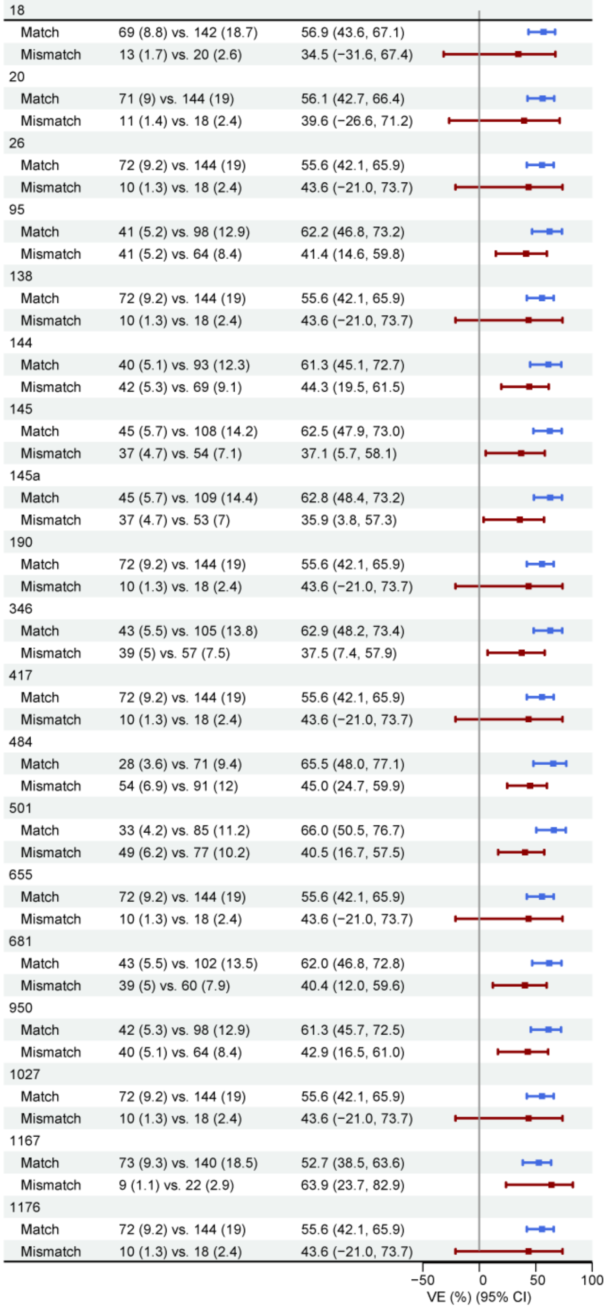

Supplementary Fig. 12

For Colombia, differential vaccine efficacy (VE) estimates against the primary COVID-19 endpoint caused by SARS-CoV-2 with a vaccine-matched (blue) or vaccine-mismatched (maroon) residue for all screened-in residues. Point estimates are shown with a filled square and horizontal bars extend through the 95% confidence interval (CI). PYRs, person-years.

A

Variant	No. of Cases (V vs. P) (Incidence per 100 PYRs)	VE (%) (95% CI)	Two-sided P-value	Two-sided Differential VE P-value
				0.17
Beta	36 (2.9) vs. 59 (4.8)	48.0 (21.6, 65.6)	0.0018	
Delta	11 (0.9) vs. 10 (0.8)	3.0 (−113.2, 55.9)	0.94	

0 25 50 75 100
VE (%) (95% CI)

B

Variant	No. of Cases (V vs. P) (Incidence per 100 PYRs)	VE (%) (95% CI)	Two-sided P-value
Not Beta	13 (1.1) vs. 17 (1.4)	23.9 (−56.4, 63.0)	0.46
Not Delta	38 (3.1) vs. 66 (5.4)	48.4 (25.7, 64.2)	<0.001

0 25 50 75 100
VE (%) (95% CI)

C

Comparison	Differential VE* (95% CI)	Two-sided Differential VE P-value	FWER P-value	Q-value
Beta vs. Not Beta	1.46 (0.60, 3.55)	0.40	0.46	0.40
Delta vs. Not Delta	0.53 (0.22, 1.27)	0.15	0.46	0.26
Beta vs. Delta	1.87 (0.77, 4.55)	0.17	0.46	0.26

*Differential VE (DVE) for genotype 1 vs. genotype 2, with VE(genotype 1) >= VE(genotype 2), is calculated as DVE = [1 – VE(genotype 2)]/[1- VE(genotype 1)], with interpretation that vaccine protection is DVE-fold better against genotype 1 than against genotype 2.

¶ Unadjusted P-value for differential VE is ≤ 0.05.

† FWER-adjusted P-value for differential VE is ≤ 0.05.

§ FDR-adjusted P-value (Q-value) for differential VE is ≤ 0.2 and unadjusted P-value for differential VE is ≤ 0.05.

D

Spike Amino Acid Position	No. of Cases (V vs. P) (Incidence per 100 PYRs)	VE (%) (95% CI)	Two-sided P-value	Two-sided Differential VE P-value	FWER P-value	Q-value
18				0.60	1	0.60
Match	34 (2.8) vs. 54 (4.4)	38.9 (8.6, 59.1)	0.017			
Mismatch	15 (1.2) vs. 22 (1.8)	51.4 (0.3, 76.3)	0.049			
19				0.15	1	0.39
Match	38 (3.1) vs. 66 (5.4)	48.4 (25.7, 64.2)	<0.001			
Mismatch	11 (0.9) vs. 10 (0.8)	3.0 (−113.2, 55.9)	0.94			
80				0.40	1	0.45
Match	13 (1.1) vs. 17 (1.4)	23.9 (−56.4, 63.0)	0.46			
Mismatch	36 (2.9) vs. 59 (4.8)	48.0 (21.6, 65.6)	0.0018			
142				0.15	1	0.39
Match	38 (3.1) vs. 66 (5.4)	48.4 (25.7, 64.2)	<0.001			
Mismatch	11 (0.9) vs. 10 (0.8)	3.0 (−113.2, 55.9)	0.94			
156				0.15	1	0.39
Match	38 (3.1) vs. 66 (5.4)	48.4 (25.7, 64.2)	<0.001			
Mismatch	11 (0.9) vs. 10 (0.8)	3.0 (−113.2, 55.9)	0.94			
157				0.15	1	0.39
Match	38 (3.1) vs. 66 (5.4)	48.4 (25.7, 64.2)	<0.001			
Mismatch	11 (0.9) vs. 10 (0.8)	3.0 (−113.2, 55.9)	0.94			
158				0.15	1	0.39
Match	38 (3.1) vs. 66 (5.4)	48.4 (25.7, 64.2)	<0.001			
Mismatch	11 (0.9) vs. 10 (0.8)	3.0 (−113.2, 55.9)	0.94			
215				0.26	1	0.39
Match	13 (1.1) vs. 15 (1.2)	15.5 (−76.7, 59.6)	0.65			
Mismatch	36 (2.9) vs. 61 (5)	49.4 (23.9, 66.4)	0.0011			
241				0.40	1	0.45
Match	13 (1.1) vs. 17 (1.4)	23.9 (−56.4, 63.0)	0.46			
Mismatch	36 (2.9) vs. 59 (4.8)	48.0 (21.6, 65.6)	0.0018			
242				0.26	1	0.39
Match	13 (1.1) vs. 15 (1.2)	15.5 (−76.7, 59.6)	0.65			
Mismatch	36 (2.9) vs. 61 (5)	49.4 (23.9, 66.4)	0.0011			
243				0.26	1	0.39
Match	13 (1.1) vs. 15 (1.2)	15.5 (−76.7, 59.6)	0.65			
Mismatch	36 (2.9) vs. 61 (5)	49.4 (23.9, 66.4)	0.0011			
* 417				0.40	1	0.45
Match	13 (1.1) vs. 17 (1.4)	23.9 (−56.4, 63.0)	0.46			
Mismatch	36 (2.9) vs. 59 (4.8)	48.0 (21.6, 65.6)	0.0018			
* 452				0.24	1	0.39
Match	38 (3.1) vs. 65 (5.3)	47.6 (24.5, 63.7)	<0.001			
Mismatch	11 (0.9) vs. 11 (0.9)	12.6 (−87.9, 59.3)	0.73			
478				0.15	1	0.39
Match	38 (3.1) vs. 66 (5.4)	48.4 (25.7, 64.2)	<0.001			
Mismatch	11 (0.9) vs. 10 (0.8)	3.0 (−113.2, 55.9)	0.94			
* 484				0.26	1	0.39
Match	13 (1.1) vs. 15 (1.2)	15.5 (−76.7, 59.6)	0.65			
Mismatch	36 (2.9) vs. 61 (5)	49.4 (23.9, 66.4)	0.0011			
501				0.22	1	0.39
Match	12 (1) vs. 13 (1.1)	9.6 (−96.4, 58.3)	0.80			
Mismatch	37 (3) vs. 63 (5.2)	49.6 (24.7, 66.3)	<0.001			
681				0.57	1	0.60
Match	37 (3) vs. 61 (5)	45.4 (20.7, 62.5)	0.0015			
Mismatch	12 (1) vs. 15 (1.2)	31.2 (−39.2, 66.0)	0.30			
701				0.32	1	0.44
Match	13 (1.1) vs. 16 (1.3)	19.9 (−65.7, 61.3)	0.55			
Mismatch	36 (2.9) vs. 60 (4.9)	48.8 (22.8, 66.1)	0.0014			
950				0.15	1	0.39
Match	38 (3.1) vs. 66 (5.4)	48.4 (25.7, 64.2)	<0.001			
Mismatch	11 (0.9) vs. 10 (0.8)	3.0 (−113.2, 55.9)	0.94			

−50 0 50 100
VE (%) (95% CI)

Supplementary Fig. 13

For the South Africa cohort, (A) vaccine efficacy (VE) estimates against the primary COVID-19 endpoint caused by specific SARS-CoV-2 lineages (lineage “X”); (B) VE estimates against the primary COVID-19 endpoint caused by all other lineages combined (“Not X”); (C) differential VE estimates against the primary COVID-19 endpoint across pairs of lineages or across a lineage (“X”) vs. all other lineages (“Not X”); and (D) VE estimates against the primary COVID-19 endpoint caused by SARS-CoV-2 with a vaccine-matched or vaccine-mismatched residue at each of the 19 screened-in Spike amino acid residues. Results for matched lineage (or residue) are shown in blue and for mismatched lineage (or residue) in maroon. In (D), the amino acid positions hypothesized to impact VE (417, 452, 484)[2-4,51] are identified with asterisks. In (A), (B), and (D), point estimates are shown with a filled square and horizontal bars extend through the 95% CI. To ensure adequate precision for estimation of genotype-specific VE, lineages with at least 20 COVID-19 endpoints were included, and amino acid positions with at least 20 vaccine-mismatched COVID-19 endpoints were included. CI, confidence interval; FWER, family-wise error rate; PYRs, person-years. In (A), (B), and (D), the “Two-sided P-value” is from testing the null hypothesis H_{Aj0} vs. H_{Aj2} using the test statistic U_{2j} (pp 17-18 of Heng et al.[52]). In (A), (C), and (D), the “Two-sided Differential VE P-value” is from testing the null hypothesis H_{B0} vs. H_{B2} using the test statistic T_2 (pp 18-19 of Heng et al.[52]).

A

Variant	No. of Cases (V vs. P) (Incidence per 100 PYRs)	VE (%) (95% CI)	Two-sided P-value	Two-sided Differential VE P-value
				0.27
Reference	52 (2) vs. 221 (9)	74.7 (66.8, 80.7)	<0.001	
Alpha	4 (0.2) vs. 16 (0.7)	82.2 (36.5, 95.0)	0.0079	
Epsilon	8 (0.3) vs. 15 (0.6)	50.8 (−9.1, 77.9)	0.081	

VE (%) (95% CI)

B

Variant	No. of Cases (V vs. P) (Incidence per 100 PYRs)	VE (%) (95% CI)	Two-sided P-value
Not Reference	14 (0.5) vs. 36 (1.5)	67.3 (40.0, 82.2)	<0.001
Not Alpha	62 (2.4) vs. 241 (9.8)	72.9 (65.2, 78.9)	<0.001
Not Epsilon	58 (2.2) vs. 242 (9.9)	75.1 (67.8, 80.7)	<0.001

VE (%) (95% CI)

C

Comparison	Differential VE* (95% CI)	Two-sided Differential VE P-value	FWER P-value	Q-value
Alpha vs. Not Alpha	1.53 (0.41, 5.68)	0.53	1	0.60
Reference vs. Not Reference	1.29 (0.66, 2.55)	0.46	1	0.60
Epsilon vs. Not Epsilon	0.51 (0.22, 1.18)	0.11	0.69	0.37
Alpha vs. Reference	1.43 (0.38, 5.34)	0.60	1	0.60
Alpha vs. Epsilon	2.77 (0.61, 12.57)	0.19	0.75	0.37
Reference vs. Epsilon	1.94 (0.83, 4.53)	0.13	0.69	0.37

*Differential VE (DVE) for genotype 1 vs. genotype 2, with VE(genotype 1) >= VE(genotype 2), is calculated as DVE = [1 − VE(genotype 2)]/[1- VE(genotype 1)], with interpretation that vaccine protection is DVE-fold better against genotype 1 than against genotype 2.

¶ Unadjusted P-value for differential VE is ≤ 0.05.

† FWER-adjusted P-value for differential VE is ≤ 0.05.

§ FDR-adjusted P-value (Q-value) for differential VE is ≤ 0.2 and unadjusted P-value for differential VE is ≤ 0.05.

D

Spike Amino Acid Position	No. of Cases (V vs. P) (Incidence per 100 PYRs)	VE (%) (95% CI)	Two-sided P-value	Two-sided Differential VE P-value	FWER P-value	Q-value
13				0.11	1	0.50
Match	58 (2.2) vs. 242 (9.9)	75.1 (67.8, 80.7)	<0.001			
Mismatch	8 (0.3) vs. 15 (0.6)	50.8 (−9.1, 77.9)	0.081			
69				0.47	1	0.57
Match	62 (2.4) vs. 240 (9.8)	72.8 (65.0, 78.8)	<0.001			
Mismatch	4 (0.2) vs. 17 (0.7)	83.2 (40.9, 95.2)	0.0054			
70				0.47	1	0.57
Match	62 (2.4) vs. 240 (9.8)	72.8 (65.0, 78.8)	<0.001			
Mismatch	4 (0.2) vs. 17 (0.7)	83.2 (40.9, 95.2)	0.0054			
144				0.49	1	0.57
Match	58 (2.2) vs. 239 (9.7)	74.3 (66.7, 80.1)	<0.001			
Mismatch	8 (0.3) vs. 18 (0.7)	64.2 (13.4, 85.2)	0.023			
152				0.016	0.19	0.10
Match	56 (2.1) vs. 242 (9.9)	76.3 (69.0, 81.8)	<0.001			
Mismatch	10 (0.4) vs. 15 (0.6)	34.3 (−40.6, 69.3)	0.28			
* 452				0.0056	0.073	0.073
Match	54 (2.1) vs. 241 (9.8)	76.6 (69.5, 82.0)	<0.001			
Mismatch	12 (0.5) vs. 16 (0.7)	31.1 (−39.7, 66.0)	0.30			
501				0.40	1	0.57
Match	61 (2.3) vs. 236 (9.6)	72.6 (64.7, 78.7)	<0.001			
Mismatch	5 (0.2) vs. 21 (0.9)	83.1 (50.1, 94.3)	0.0013			
570				0.53	1	0.57
Match	62 (2.4) vs. 241 (9.8)	72.9 (65.2, 78.9)	<0.001			
Mismatch	4 (0.2) vs. 16 (0.7)	82.2 (36.5, 95.0)	0.0079			
677				0.19	1	0.57
Match	64 (2.5) vs. 238 (9.7)	72.3 (64.6, 78.4)	<0.001			
Mismatch	2 (0.1) vs. 19 (0.8)	89.6 (56.5, 97.5)	0.0019			
681				0.37	1	0.57
Match	51 (2) vs. 218 (8.9)	75.0 (67.1, 81.0)	<0.001			
Mismatch	15 (0.6) vs. 39 (1.6)	66.0 (38.8, 81.1)	<0.001			
716				0.64	1	0.64
Match	61 (2.3) vs. 239 (9.7)	73.0 (65.3, 79.0)	<0.001			
Mismatch	5 (0.2) vs. 18 (0.7)	79.5 (38.2, 93.2)	0.0049			
982				0.53	1	0.57
Match	62 (2.4) vs. 241 (9.8)	72.9 (65.2, 78.9)	<0.001			
Mismatch	4 (0.2) vs. 16 (0.7)	82.2 (36.5, 95.0)	0.0079			
1118				0.53	1	0.57
Match	62 (2.4) vs. 241 (9.8)	72.9 (65.2, 78.9)	<0.001			
Mismatch	4 (0.2) vs. 16 (0.7)	82.2 (36.5, 95.0)	0.0079			

VE (%) (95% CI)

Supplementary Fig. 14

For the United States cohort, (A) vaccine efficacy (VE) estimates against the primary COVID-19 endpoint caused by specific SARS-CoV-2 lineages (lineage "X"); (B) VE estimates against the primary COVID-19 endpoint caused by all other lineages combined ("Not X"); (C) differential VE estimates against the primary COVID-19 endpoint across pairs of lineages or across a lineage ("X") vs. all other lineages ("Not X"); and (D) VE estimates against the primary COVID-19 endpoint caused by SARS-CoV-2 with a vaccine-matched or vaccine-mismatched residue at each of the 13 screened Spike amino acid residues. Results for matched lineage (or residue) are shown in blue and for mismatched lineage (or residue) in maroon. In (D), the amino acid position hypothesized to impact VE (452)[2-4] is identified with an asterisk. In (A), (B), and (D), point estimates are shown with a filled square and horizontal bars extend through the 95% CI. To ensure adequate precision for estimation of genotype-specific VE, lineages with at least 20 COVID-19 endpoints were included, and amino acid positions with at least 20 vaccine-mismatched COVID-19 endpoints were included. CI, confidence interval; FWER, family-wise error rate; PYRs, person-years. In (A), (B), and (D), the "Two-sided P-value" is from testing the null hypothesis H_{Aj0} vs. H_{Aj2} using the test statistic U_{2j} (pp 17-18 of Heng et al.[52]). In (A), (C), and (D), the "Two-sided Differential VE P-value" is from testing the null hypothesis H_{B0} vs. H_{B2} using the test statistic T_2 (pp 18-19 of Heng et al.[52]).

Spike Amino Acid Position	No. of Cases (V vs. P) (Incidence per 100 PYRs)	VE (%) (95% CI)	Two-sided P-value	Two-sided Differential VE P-value	FWER P-value	Q-value
18				0.08	1	0.16
Match	187 (6.6) vs. 398 (14.4)	55.5 (47.5, 62.3)	<0.001			
Mismatch	76 (2.7) vs. 115 (4.2)	40.0 (20.5, 54.8)	<0.001			
20				0.083	1	0.16
Match	190 (6.7) vs. 404 (14.6)	55.4 (47.4, 62.1)	<0.001			
Mismatch	73 (2.6) vs. 109 (3.9)	39.8 (19.6, 54.8)	<0.001			
26				0.099	1	0.16
Match	190 (6.7) vs. 402 (14.5)	55.2 (47.2, 62.0)	<0.001			
Mismatch	73 (2.6) vs. 111 (4)	40.5 (20.7, 55.3)	<0.001			
69				0.67	1	0.67
Match	253 (8.9) vs. 490 (17.7)	51.6 (44.2, 58.0)	<0.001			
Mismatch	10 (0.4) vs. 23 (0.8)	59.0 (12.9, 80.7)	0.02			
70				0.67	1	0.67
Match	253 (8.9) vs. 490 (17.7)	51.6 (44.2, 58.0)	<0.001			
Mismatch	10 (0.4) vs. 23 (0.8)	59.0 (12.9, 80.7)	0.02			
75				0.0013	0.049	0.0086
Match	219 (7.7) vs. 467 (16.9)	56.0 (48.8, 62.2)	<0.001			
Mismatch	44 (1.5) vs. 46 (1.7)	9.1 (−36.8, 39.6)	0.65			
76				0.0013	0.049	0.0086
Match	219 (7.7) vs. 467 (16.9)	56.0 (48.8, 62.2)	<0.001			
Mismatch	44 (1.5) vs. 46 (1.7)	9.1 (−36.8, 39.6)	0.65			
95				0.27	1	0.33
Match	220 (7.7) vs. 446 (16.1)	53.5 (45.9, 60.0)	<0.001			
Mismatch	43 (1.5) vs. 67 (2.4)	41.7 (15.7, 59.7)	0.0042			
138				0.096	1	0.16
Match	188 (6.6) vs. 398 (14.4)	55.3 (47.3, 62.1)	<0.001			
Mismatch	75 (2.6) vs. 115 (4.2)	40.7 (21.4, 55.2)	<0.001			
144				0.37	1	0.42
Match	213 (7.5) vs. 430 (15.5)	53.4 (45.6, 60.1)	<0.001			
Mismatch	50 (1.8) vs. 83 (3)	44.5 (21.9, 60.5)	<0.001			
145				0.18	1	0.24
Match	226 (7.9) vs. 459 (16.6)	53.6 (46.2, 60.1)	<0.001			
Mismatch	37 (1.3) vs. 54 (2)	37.5 (6.3, 58.4)	0.023			
145a				0.15	1	0.21
Match	226 (7.9) vs. 460 (16.6)	53.7 (46.3, 60.2)	<0.001			
Mismatch	37 (1.3) vs. 53 (1.9)	36.3 (4.4, 57.6)	0.03			
152				0.12	1	0.19
Match	259 (9.1) vs. 497 (18)	50.9 (43.5, 57.3)	<0.001			
Mismatch	4 (0.1) vs. 16 (0.6)	79.6 (38.9, 93.2)	0.0045			
190				0.099	1	0.16
Match	190 (6.7) vs. 402 (14.5)	55.2 (47.2, 62.0)	<0.001			
Mismatch	73 (2.6) vs. 111 (4)	40.5 (20.7, 55.3)	<0.001			
246				0.0018	0.059	0.0086
Match	219 (7.7) vs. 466 (16.8)	55.9 (48.7, 62.1)	<0.001			
Mismatch	44 (1.5) vs. 47 (1.7)	10.9 (−33.8, 40.7)	0.58			
247				0.0028	0.08	0.011
Match	220 (7.7) vs. 466 (16.8)	55.7 (48.5, 61.9)	<0.001			
Mismatch	43 (1.5) vs. 47 (1.7)	13.0 (−31.2, 42.2)	0.51			
248				0.0021	0.067	0.0086
Match	220 (7.7) vs. 467 (16.9)	55.8 (48.6, 62.0)	<0.001			
Mismatch	43 (1.5) vs. 46 (1.7)	11.1 (−34.1, 41.1)	0.57			
249				0.0092	0.23	0.025
Match	217 (7.6) vs. 458 (16.5)	55.5 (48.2, 61.8)	<0.001			
Mismatch	46 (1.6) vs. 55 (2)	21.9 (−15.1, 47.0)	0.21			
250				0.0021	0.067	0.0086
Match	220 (7.7) vs. 467 (16.9)	55.8 (48.6, 62.0)	<0.001			
Mismatch	43 (1.5) vs. 46 (1.7)	11.1 (−34.1, 41.1)	0.57			

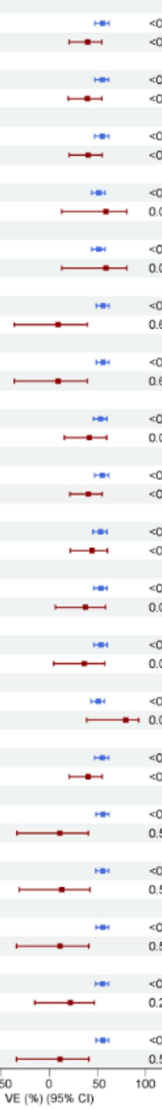

Spike Amino Acid Position	No. of Cases (V vs. P) (Incidence per 100 PYRs)	VE (%) (95% CI)	Two-sided P-value	Two-sided Differential VE P-value	FWER P-value	Q-value
251				0.0021	0.067	0.0086
Match	220 (7.7) vs. 467 (16.9)	55.8 (48.6, 62.0)	<0.001			
Mismatch	43 (1.5) vs. 46 (1.7)	11.1 (−34.1, 41.1)	0.57			
252				0.0021	0.067	0.0086
Match	220 (7.7) vs. 467 (16.9)	55.8 (48.6, 62.0)	<0.001			
Mismatch	43 (1.5) vs. 46 (1.7)	11.1 (−34.1, 41.1)	0.57			
253				0.0013	0.049	0.0086
Match	219 (7.7) vs. 467 (16.9)	56.0 (48.9, 62.2)	<0.001			
Mismatch	44 (1.5) vs. 46 (1.7)	9.1 (−36.9, 39.6)	0.65			
346				0.16	1	0.21
Match	223 (7.8) vs. 455 (16.4)	53.8 (46.3, 60.2)	<0.001			
Mismatch	40 (1.4) vs. 58 (2.1)	37.3 (7.4, 57.6)	0.019			
414				0.0093	0.23	0.025
Match	259 (9.1) vs. 482 (17.4)	49.8 (42.2, 56.4)	<0.001			
Mismatch	4 (0.1) vs. 31 (1.1)	86.5 (64.1, 94.9)	<0.001			
* 417				0.099	1	0.16
Match	190 (6.7) vs. 402 (14.5)	55.2 (47.2, 62.0)	<0.001			
Mismatch	73 (2.6) vs. 111 (4)	40.5 (20.7, 55.3)	<0.001			
* 452				0.0059	0.15	0.018
Match	219 (7.7) vs. 462 (16.7)	55.5 (48.3, 61.8)	<0.001			
Mismatch	44 (1.5) vs. 51 (1.8)	18.3 (−21.8, 45.2)	0.32			
* 484				0.45	1	0.51
Match	110 (3.9) vs. 232 (8.4)	54.9 (43.9, 63.7)	<0.001			
Mismatch	153 (5.4) vs. 281 (10.1)	49.5 (39.0, 58.2)	<0.001			
* 490				0.0014	0.049	0.0086
Match	219 (7.7) vs. 467 (16.9)	56.0 (48.8, 62.2)	<0.001			
Mismatch	44 (1.5) vs. 46 (1.7)	9.1 (−36.9, 39.7)	0.65			
501				0.024	0.53	0.056
Match	147 (5.1) vs. 335 (12.1)	58.0 (49.5, 65.1)	<0.001			
Mismatch	116 (4.1) vs. 178 (6.4)	40.9 (26.1, 52.8)	<0.001			
655				0.13	1	0.19
Match	189 (6.6) vs. 398 (14.4)	55.0 (47.0, 61.8)	<0.001			
Mismatch	74 (2.6) vs. 115 (4.2)	41.6 (22.4, 56.0)	<0.001			
681				0.51	1	0.55
Match	219 (7.7) vs. 438 (15.8)	52.9 (45.2, 59.6)	<0.001			
Mismatch	44 (1.5) vs. 75 (2.7)	46.2 (22.7, 62.5)	<0.001			
778				0.02	0.46	0.049
Match	262 (9.2) vs. 493 (17.8)	50.3 (42.8, 56.8)	<0.001			
Mismatch	1 (0) vs. 20 (0.7)	94.1 (64.7, 99.0)	0.0019			
859				0.0041	0.11	0.014
Match	219 (7.7) vs. 464 (16.8)	55.7 (48.4, 61.9)	<0.001			
Mismatch	44 (1.5) vs. 49 (1.8)	16.2 (−25.1, 43.9)	0.39			
950				0.37	1	0.42
Match	223 (7.8) vs. 449 (16.2)	53.2 (45.6, 59.7)	<0.001			
Mismatch	40 (1.4) vs. 64 (2.3)	43.3 (17.1, 61.3)	0.0035			
1027				0.099	1	0.16
Match	190 (6.7) vs. 402 (14.5)	55.2 (47.2, 62.0)	<0.001			
Mismatch	73 (2.6) vs. 111 (4)	40.5 (20.7, 55.3)	<0.001			
1167				0.27	1	0.33
Match	250 (8.8) vs. 479 (17.3)	50.9 (43.3, 57.4)	<0.001			
Mismatch	13 (0.5) vs. 34 (1.2)	65.7 (36.4, 81.5)	<0.001			
1176				0.67	1	0.67
Match	151 (5.3) vs. 284 (10.3)	50.5 (40.3, 59.0)	<0.001			
Mismatch	112 (3.9) vs. 229 (8.3)	53.6 (42.3, 62.8)	<0.001			

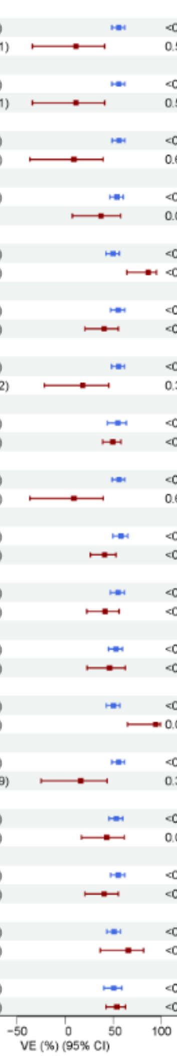

Supplementary Fig. 15

For the Latin America cohort, vaccine efficacy against the primary COVID-19 endpoint caused by SARS-CoV-2 genotype defined by a vaccine-matched (blue) or vaccine-mismatched (maroon) residue at each of the 36 screened-in Spike amino acid positions. Results for matched residue are shown in blue and for mismatched residue in maroon. Point estimates are shown with a filled square and horizontal bars extend through the 95% CI. The four amino acid positions (417, 452, 484, 490) hypothesized to impact VE[2-4,51] are identified with asterisks. CI, confidence interval; FWER, family-wise error rate; PYRs, person-years. The "Two-sided P-value" is from testing the null hypothesis H_{Aj0} vs. H_{Aj2} using the test statistic U_{2j} (pp 17-18 of Heng et al.[52]). The "Two-sided Differential VE P-value" is from testing the null hypothesis H_{B0} vs. H_{B2} using the test statistic T_2 (pp 18-19 of Heng et al.[52]).

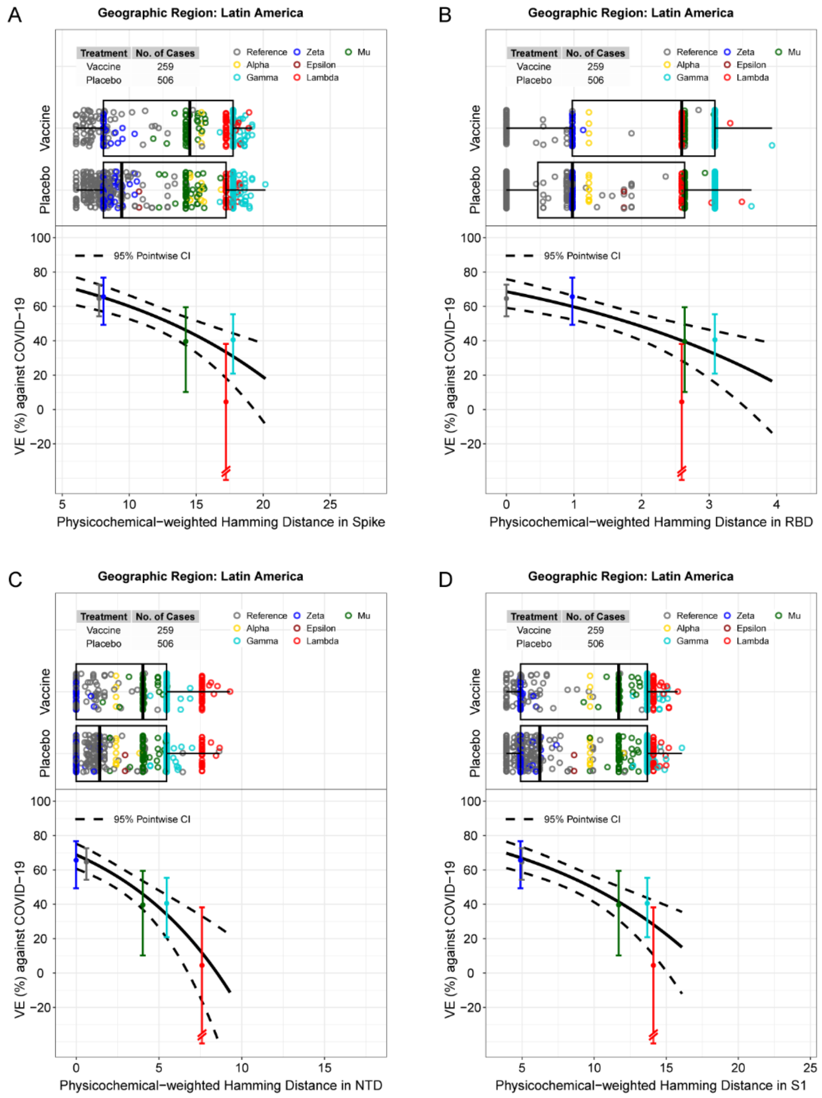

Supplementary Fig. 16

For the Latin America cohort, sensitivity analysis of vaccine efficacy (VE) against the primary COVID-19 endpoint by physicochemical-weighted (PCW) Hamming distances in (A) Spike, (B) the RBD domain, (C) the NTD domain, or (D) the S1 region of the disease-causing SARS-CoV-2 isolate to that of the vaccine-insert sequence. The analysis was performed after deleting observations with Spike physicochemical-weighted Hamming distance greater than 20 (11 observations corresponding to Zeta and Lambda). The top plot in each panel shows the distributions of distances by treatment arm, color-coded by lineage. The left and right edges of the box plots represent the 25th and 75th percentiles of PCW-Hamming distance in the designated region and the vertical middle line represents the 50th percentile. The horizontal bars extend from the 25th (or 75th) percentile of PCW-Hamming distance to the minimum (or maximum) PCW-Hamming distance within the 25th (or 75th) percentile of Hamming distance minus (or plus) 1.5 times the interquartile range. The bottom plot in each panel shows the estimated VE by SARS-CoV-2 sequence distance. The dotted lines are pointwise 95% confidence intervals. The dots are overall VE estimates for the given lineage placed at the lineage-specific median distance of placebo arm endpoints, with vertical bars indicating their pointwise 95% confidence intervals.

Supplementary Table 10.

Among COVID-19 primary endpoint cases, point estimates of means (95% confidence intervals) of physicochemical-weighted Hamming distances in the Spike protein of the disease-causing SARS-CoV-2 isolate to that of the vaccine-insert sequence by treatment arm, and of mean differences (vaccine - placebo). Doubly robust targeted minimum loss-based estimation was used to adjust for baseline covariates (the indicators of each biweekly calendar period of the enrollment date, and, for the geographic region-pooled analysis, also indicators representing the geographic regions [US, South Africa, Colombia, Latin America excluding Colombia]; the Latin America analysis also included the indicator of Colombia) and to bias-correct by modeling the probability of observing a sequence dependent on log10 viral load. Results are shown for each geographic region and for all geographic regions pooled.

Geographic Region	Sequence Region	Vaccine	Placebo	Difference (Vaccine – Placebo)
Pooled	Spike	12.31 (11.84, 12.79)	11.25 (10.94, 11.55)	1.07 (0.53, 1.60)
Pooled	RBD	1.63 (1.51, 1.75)	1.38 (1.30, 1.46)	0.25 (0.11, 0.39)
Pooled	NTD	3.19 (2.89, 3.48)	2.48 (2.29, 2.66)	0.71 (0.38, 1.04)
Pooled	S1	9.24 (8.82, 9.67)	8.21 (7.94, 8.49)	1.03 (0.55, 1.51)
Pooled	S2	3.01 (2.93, 3.09)	3.01 (2.95, 3.07)	0.0038 (-0.091, 0.099)
Latin America	Spike	13.55 (12.97, 14.13)	12.08 (11.66, 12.49)	1.47 (0.76, 2.19)
Latin America	RBD	1.93 (1.78, 2.07)	1.56 (1.45, 1.66)	0.37 (0.18, 0.55)
Latin America	NTD	3.88 (3.51, 4.25)	2.85 (2.59, 3.12)	1.03 (0.58, 1.49)
Latin America	S1	10.35 (9.82, 10.89)	8.83 (8.45, 9.22)	1.52 (0.86, 2.18)
Latin America	S2	3.24 (3.14, 3.34)	3.21 (3.15, 3.28)	0.024 (-0.09, 0.14)
South Africa	Spike	14.49 (13.21, 15.77)	14.86 (14.47, 15.25)	-0.37 (-1.71, 0.98)
South Africa	RBD	2.87 (2.42, 3.31)	3.04 (2.94, 3.15)	-0.17 (-0.63, 0.29)
South Africa	NTD	4.74 (4.02, 5.45)	4.93 (4.69, 5.17)	-0.19 (-0.95, 0.56)
South Africa	S1	11.67 (10.52, 12.82)	11.80 (11.46, 12.14)	-0.13 (-1.33, 1.07)
South Africa	S2	2.84 (2.71, 2.97)	3.03 (2.90, 3.17)	-0.19 (-0.38, 0.0005)
US	Spike	8.48 (7.75, 9.21)	7.90 (7.58, 8.22)	0.58 (-0.21, 1.37)
US	RBD	0.54 (0.35, 0.72)	0.31 (0.24, 0.38)	0.22 (0.027, 0.42)
US	NTD	1.06 (0.63, 1.49)	0.67 (0.53, 0.82)	0.39 (-0.064, 0.85)
US	S1	5.89 (5.27, 6.50)	5.30 (5.06, 5.54)	0.58 (-0.08, 1.24)
US	S2	2.57 (2.38, 2.76)	2.53 (2.42, 2.65)	0.035 (-0.18, 0.25)

Supplementary Table 11.
Among COVID-19 primary endpoint cases, point estimates of means (95% confidence intervals) of physicochemical-weighted Hamming distances in the Spike protein of the disease-causing SARS-CoV-2 isolate to that of the vaccine-insert sequence by treatment arm, and of mean differences (vaccine - placebo). Inverse probability weighting was used to accommodate missing SARS-CoV-2 AA sequences by modeling the probability of observing a sequence dependent on log10 viral load and region. Results are shown for each geographic region and for all geographic regions pooled.

Geographic Region	Sequence Region	Vaccine	Placebo	Difference (Vaccine – Placebo)
Pooled	Spike	12.80 (12.33, 13.27)	11.04 (10.73, 11.36)	1.76 (1.19, 2.32)
Pooled	RBD	1.77 (1.65, 1.90)	1.29 (1.20, 1.37)	0.49 (0.33, 0.64)
Pooled	NTD	3.48 (3.19, 3.76)	2.35 (2.16, 2.54)	1.13 (0.78, 1.47)
Pooled	S1	9.70 (9.28, 10.13)	8.03 (7.74, 8.31)	1.68 (1.17, 2.19)
Pooled	S2	3.07 (2.98, 3.15)	2.99 (2.93, 3.05)	0.081 (-0.023, 0.18)
Latin America	Spike	13.58 (13.00, 14.15)	12.05 (11.63, 12.46)	1.53 (0.82, 2.24)
Latin America	RBD	1.91 (1.77, 2.06)	1.54 (1.44, 1.65)	0.37 (0.19, 0.55)
Latin America	NTD	3.86 (3.49, 4.23)	2.83 (2.56, 3.10)	1.03 (0.58, 1.49)
Latin America	S1	10.31 (9.78, 10.84)	8.81 (8.43, 9.19)	1.50 (0.84, 2.15)
Latin America	S2	3.24 (3.14, 3.34)	3.22 (3.14, 3.29)	0.021 (-0.1, 0.14)
South Africa	Spike	14.91 (14.32, 15.50)	15.32 (14.85, 15.80)	-0.42 (-1.17, 0.34)
South Africa	RBD	2.79 (2.62, 2.96)	2.94 (2.80, 3.08)	-0.15 (-0.37, 0.076)
South Africa	NTD	4.90 (4.53, 5.26)	5.00 (4.71, 5.30)	-0.1 (-0.58, 0.37)
South Africa	S1	11.98 (11.44, 12.53)	12.25 (11.81, 12.69)	-0.27 (-0.97, 0.43)
South Africa	S2	2.91 (2.75, 3.06)	3.05 (2.92, 3.18)	-0.14 (-0.35, 0.06)
US	Spike	8.45 (7.82, 9.08)	7.83 (7.50, 8.15)	0.62 (-0.088, 1.33)
US	RBD	0.54 (0.4, 0.69)	0.31 (0.23, 0.39)	0.23 (0.068, 0.4)
US	NTD	1.05 (0.74, 1.36)	0.64 (0.48, 0.8)	0.41 (0.064, 0.76)
US	S1	5.86 (5.37, 6.35)	5.26 (5.00, 5.51)	0.61 (0.053, 1.16)
US	S2	2.56 (2.35, 2.76)	2.52 (2.42, 2.63)	0.032 (-0.2, 0.26)

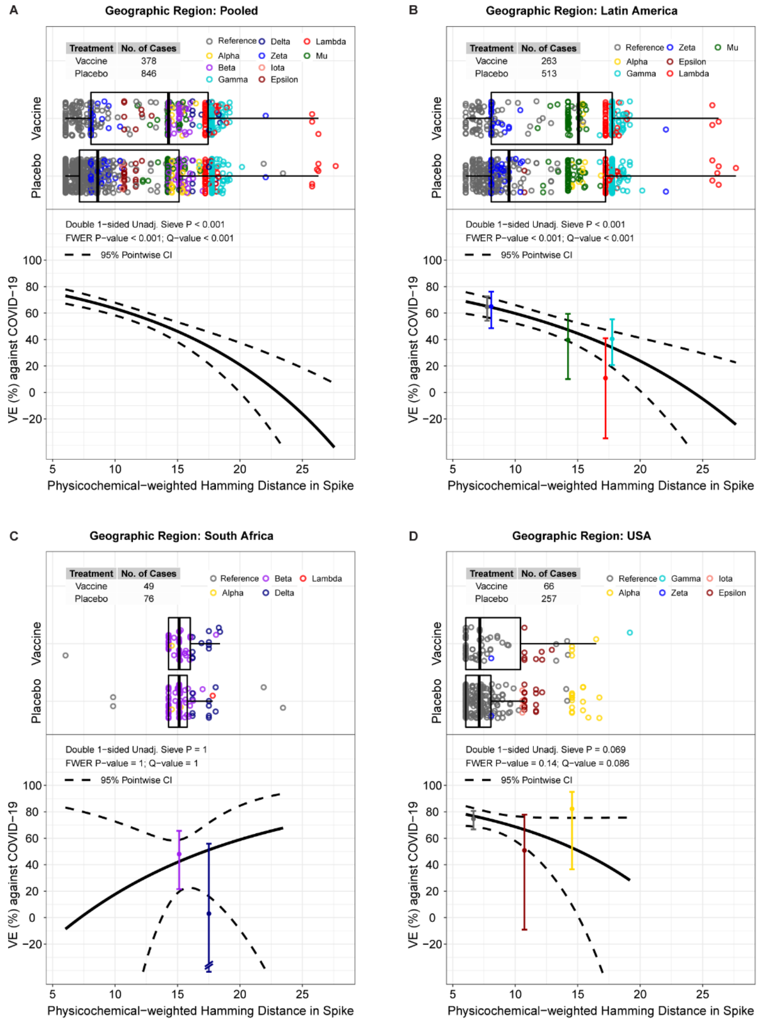

Supplementary Fig. 17

For (A) the geographic regions pooled, (B) the Latin America cohort, (C) the South Africa cohort, and (D) the United States cohort, hazard-based vaccine efficacy (VE) against the primary COVID-19 endpoint by physicochemical-weighted (PCW) Hamming distances in the Spike protein of the disease-causing SARS-CoV-2 isolate to that of the vaccine-insert sequence. The top plot in each panel shows the distributions of physicochemical weighted Hamming distances by treatment arm, color-coded by lineage. The left and right edges of the box plots represent the 25th and 75th percentiles of PCW-Hamming distance in Spike and the vertical middle line represents the 50th percentile. The horizontal bars extend from the 25th (or 75th) percentile of PCW-Hamming distance to the minimum (or maximum) PCW-Hamming distance within the 25th (or 75th) percentile of Hamming distance minus (or plus) 1.5 times the interquartile range. The bottom plot in each panel shows the estimated vaccine efficacy by physicochemical-weighted Hamming distance of the disease-causing SARS-CoV-2 isolate Spike to that of the vaccine insert Spike. The dotted lines are pointwise 95% confidence intervals. The dots are overall vaccine efficacy estimates for the given lineage, with the vertical bars showing the 95% confidence intervals. The "Double 1-sided unadjusted sieve p-value" doubles the p-value from a one-sided Wald test of the null hypothesis of constant VE vs. the alternative hypothesis of a decreasing VE with an increasing value of the feature on the x-axis (Juraska and Gilbert,[53] Section 5).

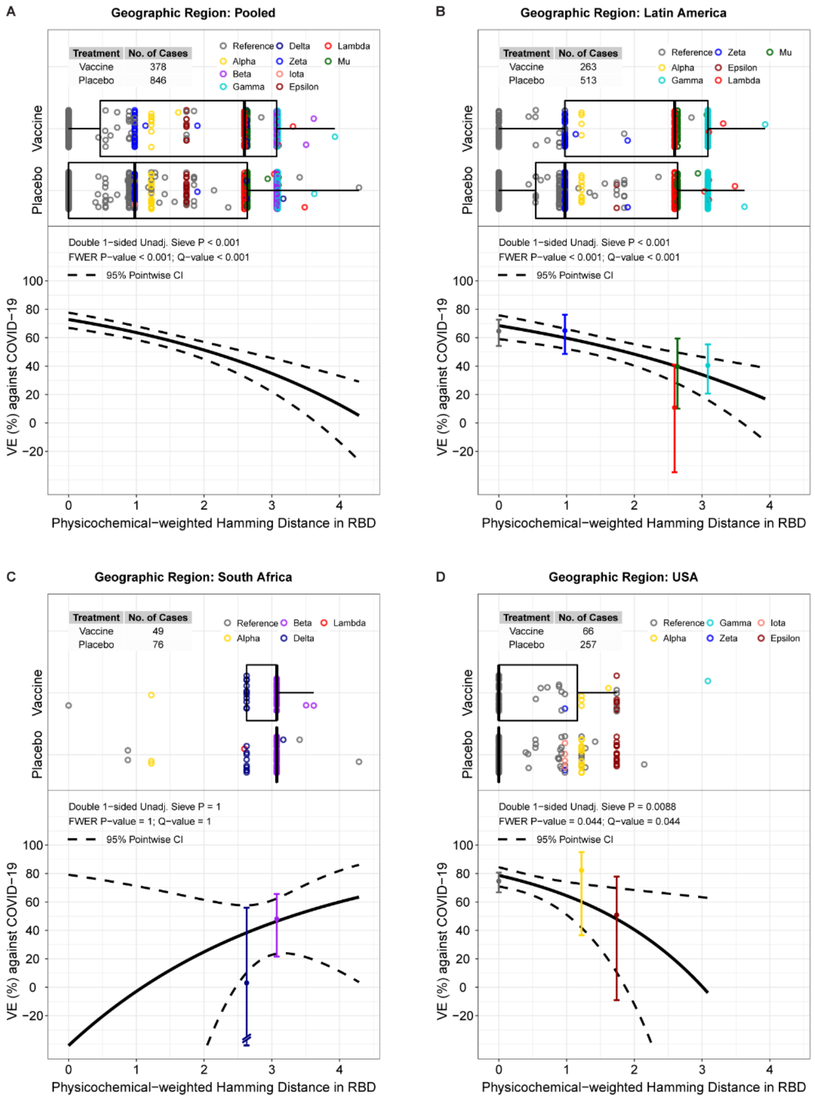

Supplementary Fig. 18

For (A) the geographic regions pooled, (B) the Latin America cohort, (C) the South Africa cohort, and (D) the United States cohort, hazard-based vaccine efficacy (VE) against the primary COVID-19 endpoint by physicochemical-weighted (PCW) Hamming distances in the RBD of the disease-causing SARS-CoV-2 isolate to that of the vaccine-insert sequence. The top plot in each panel shows the distributions of physicochemical weighted Hamming distances by treatment arm, color-coded by lineage. The left and right edges of the box plots represent the 25th and 75th percentiles of PCW-Hamming distance in the RBD and the vertical middle line represents the 50th percentile. The horizontal bars extend from the 25th (or 75th) percentile of PCW-Hamming distance to the minimum (or maximum) PCW-Hamming distance within the 25th (or 75th) percentile of Hamming distance minus (or plus) 1.5 times the interquartile range. The bottom plot in each panel shows the estimated vaccine efficacy by physicochemical-weighted Hamming distance of the disease-causing SARS-CoV-2 isolate RBD to that of the vaccine insert RBD. The dotted lines are pointwise 95% confidence intervals. The dots are overall vaccine efficacy estimates for the given lineage, with the vertical bars showing the 95% confidence intervals. The "Double 1-sided unadjusted sieve p-value" doubles the p-value from a one-sided Wald test of the null hypothesis of constant VE vs. the alternative hypothesis of a decreasing VE with an increasing value of the feature on the x-axis (Juraska and Gilbert,[53] Section 5).

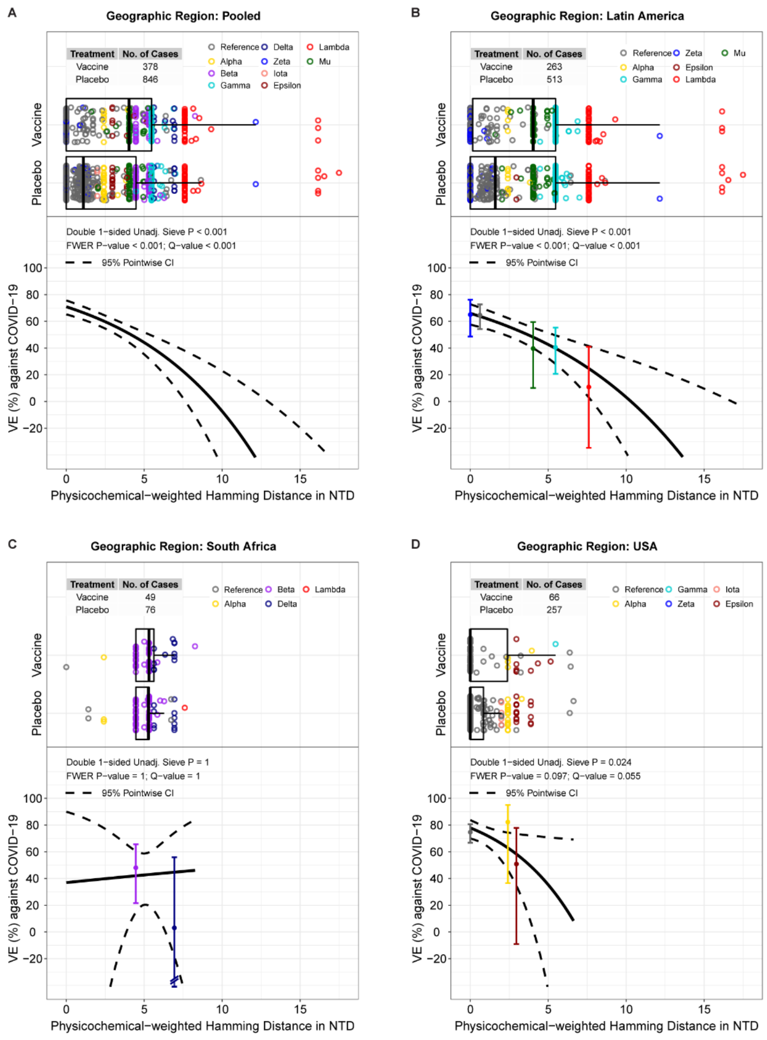

Supplementary Fig. 19

For (A) the geographic regions pooled, (B) the Latin America cohort, (C) the South Africa cohort, and (D) the United States cohort, hazard-based vaccine efficacy (VE) against the primary COVID-19 endpoint by physicochemical-weighted (PCW) Hamming distances in the NTD of the disease-causing SARS-CoV-2 isolate to that of the vaccine-insert sequence. The top plot in each panel shows the distributions of physicochemical weighted Hamming distances by treatment arm, color-coded by lineage. The left and right edges of the box plots represent the 25th and 75th percentiles of PCW-Hamming distance in the NTD and the vertical middle line represents the 50th percentile. The horizontal bars extend from the 25th (or 75th) percentile of PCW-Hamming distance to the minimum (or maximum) PCW-Hamming distance within the 25th (or 75th) percentile of Hamming distance minus (or plus) 1.5 times the interquartile range. The bottom plot in each panel shows the estimated vaccine efficacy by physicochemical-weighted Hamming distance of the disease-causing SARS-CoV-2 isolate NTD to that of the vaccine insert NTD. The dotted lines are pointwise 95% confidence intervals. The dots are overall vaccine efficacy estimates for the given lineage, with the vertical bars showing the 95% confidence intervals. The “Double 1-sided unadjusted sieve p-value” doubles the p-value from a one-sided Wald test of the null hypothesis of constant VE vs. the alternative hypothesis of a decreasing VE with an increasing value of the feature on the x-axis (Juraska and Gilbert,[53] Section 5).

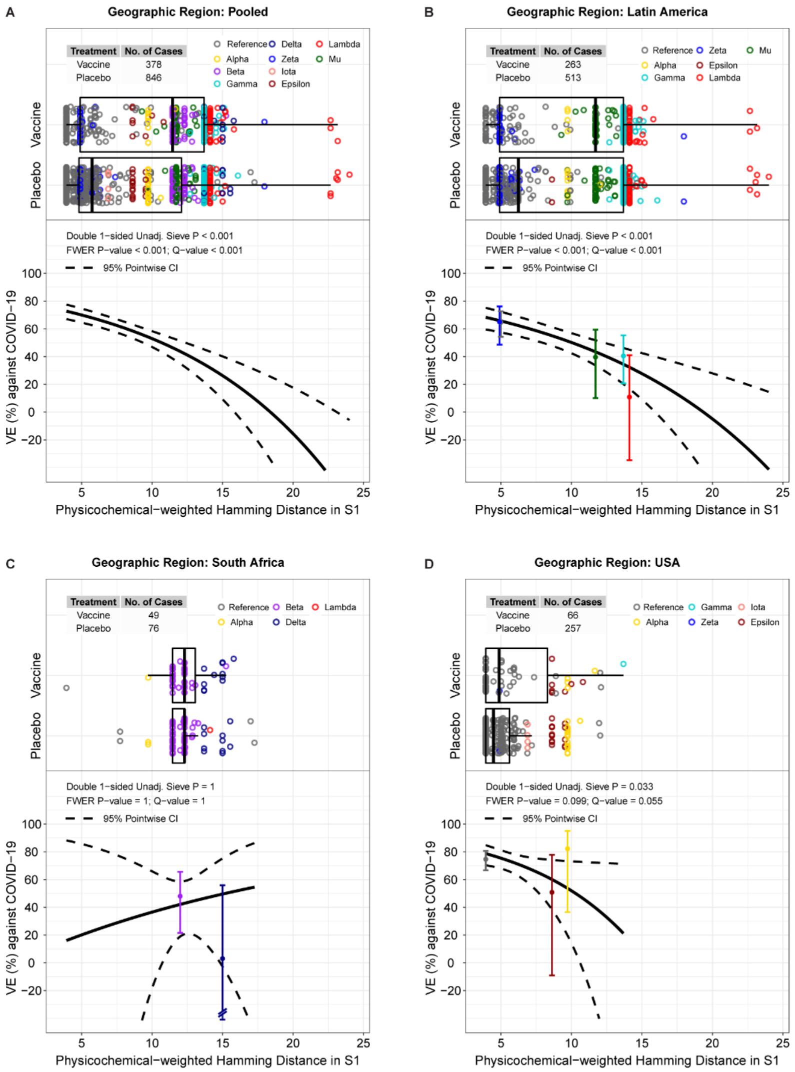

Supplementary Fig. 20

For (A) the geographic regions pooled, (B) the Latin America cohort, (C) the South Africa cohort, and (D) the United States cohort, hazard-based vaccine efficacy (VE) against the primary COVID-19 endpoint by physicochemical-weighted (PCW) Hamming distances in the S1 region of the disease-causing SARS-CoV-2 isolate to that of the vaccine-insert sequence. The top plot in each panel shows the distributions of physicochemical weighted Hamming distances by treatment arm, color-coded by lineage. The left and right edges of the box plots represent the 25th and 75th percentiles of PCW-Hamming distance in S1 and the vertical middle line represents the 50th percentile. The horizontal bars extend from the 25th (or 75th) percentile of PCW-Hamming distance to the minimum (or maximum) PCW-Hamming distance within the 25th (or 75th) percentile of Hamming distance minus (or plus) 1.5 times the interquartile range. The bottom plot in each panel shows the estimated vaccine efficacy by physicochemical-weighted Hamming distance of the disease-causing SARS-CoV-2 isolate S1 region to that of the vaccine insert S1 region. The dotted lines are pointwise 95% confidence intervals. The dots are overall vaccine efficacy estimates for the given lineage, with the vertical bars showing the 95% confidence intervals. The "Double 1-sided unadjusted sieve p-value" doubles the p-value from a one-sided Wald test of the null hypothesis of constant VE vs. the alternative hypothesis of a decreasing VE with an increasing value of the feature on the x-axis (Juraska and Gilbert,[53] Section 5).

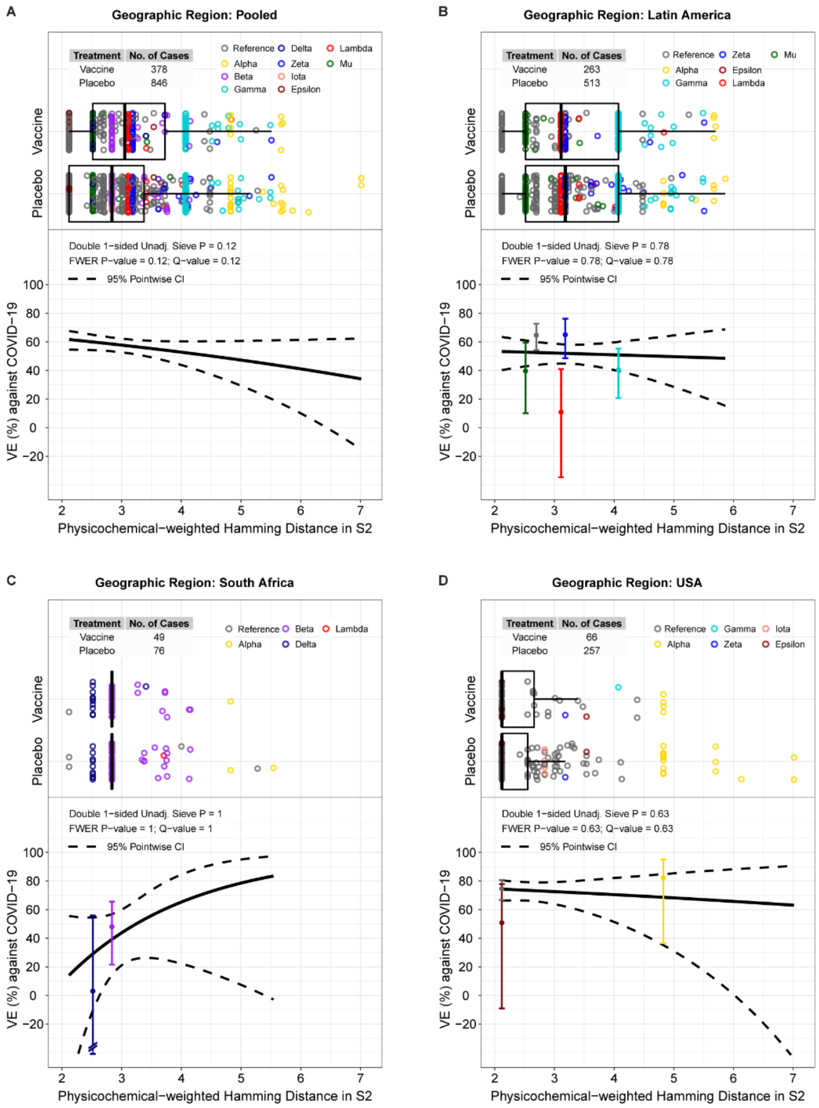

Supplementary Fig. 21

For (A) the geographic regions pooled, (B) the Latin America cohort, (C) the South Africa cohort, and (D) the United States cohort, hazard-based vaccine efficacy (VE) against the primary COVID-19 endpoint by physicochemical-weighted (PCW) Hamming distances in the S2 region of the disease-causing SARS-CoV-2 isolate to that of the vaccine-insert sequence. The top plot in each panel shows the distributions of physicochemical weighted Hamming distances by treatment arm, color-coded by lineage. The left and right edges of the box plots represent the 25th and 75th percentiles of PCW-Hamming distance in S2 and the vertical middle line represents the 50th percentile. The horizontal bars extend from the 25th (or 75th) percentile of PCW-Hamming distance to the minimum (or maximum) PCW-Hamming distance within the 25th (or 75th) percentile of Hamming distance minus (or plus) 1.5 times the interquartile range. The bottom plot in each panel shows the estimated vaccine efficacy by physicochemical-weighted Hamming distance of the disease-causing SARS-CoV-2 isolate S2 region to that of the vaccine insert S2 region. The dotted lines are pointwise 95% confidence intervals. The dots are overall vaccine efficacy estimates for the given lineage, with the vertical bars showing the 95% confidence intervals. The "Double 1-sided unadjusted sieve p-value" doubles the p-value from a one-sided Wald test of the null hypothesis of constant VE vs. the alternative hypothesis of a decreasing VE with an increasing value of the feature on the x-axis (Juraska and Gilbert,[53] Section 5).

Supplementary Table 12. For physicochemical-weighted Hamming distance to the vaccine insert, p-values from the Juraska and Gilbert[54] one sided weighted Wald test of $H_{10} : VE(v) = 0$ for all v and from double one-sided Wald test of $H_{20} : VE(v) = VE$ for all v against $H_{21} : VE(v)$ decreases with v (a test for a differential VE also called a sieve effect). Family-wise error rate (FWER) adjusted p-values are Holm-Bonferroni and FDR-adjusted p-values (q-values) are Benjamini-Hochberg.

			Test of H_{20}		
Geographic Region	Sequence Region	Test of H_{10} p-value	p-value	FWER p-value	q-value
Pooled	Spike¶§†	<0.001	<0.001	<0.001	<0.001
	RBD¶§†	<0.001	<0.001	<0.001	<0.001
	NTD¶§†	<0.001	<0.001	<0.001	<0.001
	S1¶§†	<0.001	<0.001	<0.001	<0.001
	S2	<0.001	0.12	0.12	0.12
Latin America	Spike¶§†	<0.001	<0.001	<0.001	<0.001
	RBD¶§†	<0.001	<0.001	<0.001	<0.001
	NTD¶§†	<0.001	<0.001	<0.001	<0.001
	S1¶§†	<0.001	<0.001	<0.001	<0.001
	S2	<0.001	0.78	0.78	0.78
South Africa	Spike	0.14	1.00	1.00	1.00
	RBD	0.0055	1.00	1.00	1.00
	NTD	0.0097	1.00	1.00	1.00
	S1	0.058	1.00	1.00	1.00
	S2	0.0079	1.00	1.00	1.00
US	Spike	<0.001	0.069	0.14	0.086
	RBD¶§†	<0.001	0.0088	0.044	0.044
	NTD¶§	<0.001	0.024	0.097	0.055
	S1¶§	<0.001	0.033	0.099	0.055
	S2	<0.001	0.63	0.63	0.63

¶ Unadjusted p-value for differential VE is ≤ 0.05.
† FWER-adjusted p-value for differential VE is ≤ 0.05.
§ FDR-adjusted p-value (q-value) for differential VE is ≤ 0.2 and unadjusted p-value for VE is ≤ 0.05.

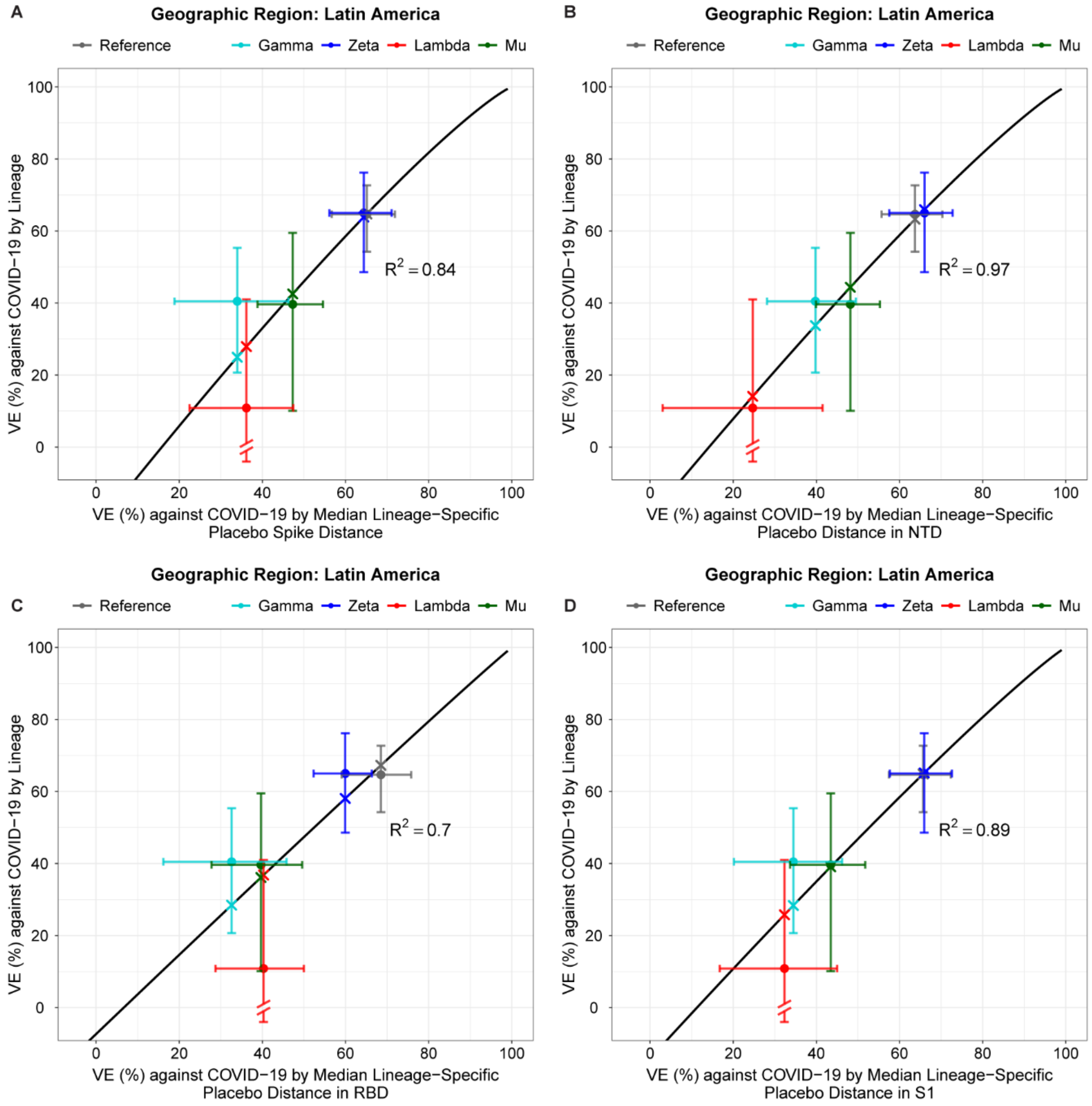

Supplementary Fig. 22

For the Latin America cohort, VE against each lineage (y-axis) vs. VE against COVID-19 with physicochemical weighted Hamming distance equal to the median of all placebo COVID-19 endpoint distances restricting to the given lineage (x-axis). Crosses indicate 95% confidence intervals. Results are shown for the distances computed for (A) Spike, (B) NTD, (C) RBD, and (D) S1. R^2 is the proportion of variance explained from a linear model fit.

Supplementary Table 13. For deep mutational scanning (DMS) antibody escape score and VE against the primary COVID-19 endpoint, p-values from the Juraska and Gilbert[54] one sided weighted Wald test of $H_{10}: VE(v) = 0$ for all v and from double one-sided Wald test of $H_{20}: VE(v) = VE$ for all v against H_{21} : $VE(v)$ decreases with v. Family-wise error rate (FWER) adjusted p-values are Holm-Bonferroni and FDR-adjusted p-values (q-values) are Benjamini-Hochberg.

Geographic Region	Feature Name	Spike Region	Antibody Set	Test of H_{10} p-value	Test of H_{20} p-value	FWER p-value	q-value
Pooled	DMS	RBD	All¶§ †	<0.001	<0.001	<0.001	<0.001
	DMS1	RBD	Cluster 1¶§†	<0.001	<0.001	0.0013	<0.001
	DMS2	RBD	Cluster 2¶§†	<0.001	<0.001	<0.001	<0.001
	DMS3	RBD	Cluster 3¶§†	<0.001	0.001	0.0013	0.001
	DMS5	RBD	Cluster 5¶§†	<0.001	<0.001	<0.001	<0.001
	DMS6	RBD	Cluster 6¶§†	<0.001	<0.001	<0.001	<0.001
	DMS7	RBD	Cluster 7¶§†	<0.001	<0.001	<0.001	<0.001
	DMS8	RBD	Cluster 8¶§†	<0.001	<0.001	0.0013	<0.001
	DMS9	RBD	Cluster 9¶§†	<0.001	<0.001	0.0013	<0.001
Latin America	DMS	RBD	All¶§ †	<0.001	0.0037	0.024	0.0084
	DMS1	RBD	Cluster 1¶§	<0.001	0.027	0.081	0.035
	DMS2	RBD	Cluster 2¶§†	<0.001	0.0034	0.024	0.0084
	DMS3	RBD	Cluster 3	<0.001	0.23	0.23	0.23
	DMS5	RBD	Cluster 5¶§	<0.001	0.014	0.055	0.021
	DMS6	RBD	Cluster 6¶§†	<0.001	0.0024	0.019	0.0084
	DMS7	RBD	Cluster 7¶§†	<0.001	<0.001	0.0065	0.0065
	DMS8	RBD	Cluster 8¶§†	<0.001	0.005	0.025	0.0089
	DMS9	RBD	Cluster 9¶§	<0.001	0.031	0.081	0.035
South Africa	DMS	RBD	All	<0.001	1.00	1.00	1.00
	DMS1	RBD	Cluster 1	<0.001	1.00	1.00	1.00
	DMS2	RBD	Cluster 2	<0.001	1.00	1.00	1.00
	DMS3	RBD	Cluster 3	<0.001	1.00	1.00	1.00
	DMS5	RBD	Cluster 5	0.0033	1.00	1.00	1.00
	DMS6	RBD	Cluster 6	<0.001	1.00	1.00	1.00
	DMS7	RBD	Cluster 7	<0.001	1.00	1.00	1.00
	DMS8	RBD	Cluster 8	<0.001	1.00	1.00	1.00
	DMS9	RBD	Cluster 9	<0.001	1.00	1.00	1.00
US	DMS	RBD	All¶§	<0.001	0.03	0.21	0.09
	DMS1	RBD	Cluster 1¶§	<0.001	0.012	0.11	0.09
	DMS2	RBD	Cluster 2	<0.001	0.052	0.26	0.094
	DMS3	RBD	Cluster 3	<0.001	0.19	0.63	0.24
	DMS5	RBD	Cluster 5	<0.001	0.16	0.63	0.24
	DMS6	RBD	Cluster 6¶§	<0.001	0.023	0.18	0.09
	DMS7	RBD	Cluster 7	<0.001	0.64	0.64	0.64
	DMS8	RBD	Cluster 8¶§	<0.001	0.043	0.26	0.094
	DMS9	RBD	Cluster 9	<0.001	0.22	0.63	0.24

¶ Unadjusted p-value for differential VE is ≤ 0.05.
† FWER-adjusted p-value for differential VE is ≤ 0.05.
§ FDR-adjusted p-value (q-value) for differential VE is ≤ 0.2 and unadjusted p-value for differential VE is ≤ 0.05.

Supplementary Table 14.
Among COVID-19 primary endpoint cases, point estimates of means (95% confidence intervals) of deep mutational scanning (DMS) antibody escape score of the disease-causing SARS-CoV-2 isolate by treatment arm, and of mean differences (vaccine - placebo). Doubly robust targeted minimum loss-based estimation was used to adjust for baseline covariates (the indicators of each biweekly calendar period of the enrollment date, and, for the geographic region-pooled analysis, also indicators representing the geographic regions [US, South Africa, Colombia, Latin America excluding Colombia]; the Latin America analysis also included the indicator of Colombia) and to bias-correct by modeling the probability of observing a sequence dependent on log10 viral load. Results are shown for each geographic region and for all geographic regions pooled.

Geographic Region	Feature Name	Spike Region	Antibody Set	Vaccine	Placebo	Difference (Vaccine – Placebo)
Pooled	DMS	RBD	All	0.22 (0.2, 0.24)	0.2 (0.18, 0.21)	0.026 (0.0047, 0.047)
	DMS1	RBD	Cluster 1	0.15 (0.13, 0.17)	0.13 (0.12, 0.15)	0.021 (-0.002, 0.044)
	DMS2	RBD	Cluster 2	0.15 (0.13, 0.17)	0.13 (0.11, 0.14)	0.023 (0.0029, 0.043)
	DMS3	RBD	Cluster 3	0.13 (0.12, 0.14)	0.12 (0.12, 0.13)	0.0078 (-0.0063, 0.022)
	DMS5	RBD	Cluster 5	0.53 (0.48, 0.57)	0.47 (0.44, 0.51)	0.051 (0.0032, 0.098)
	DMS6	RBD	Cluster 6	0.092 (0.081, 0.1)	0.068 (0.061, 0.075)	0.024 (0.011, 0.037)
	DMS7	RBD	Cluster 7	0.13 (0.12, 0.14)	0.11 (0.11, 0.12)	0.015 (0.0019, 0.028)
	DMS8	RBD	Cluster 8	0.094 (0.074, 0.11)	0.057 (0.046, 0.068)	0.037 (0.014, 0.059)
	DMS9	RBD	Cluster 9	0.048 (0.041, 0.055)	0.042 (0.037, 0.047)	0.0058 (-0.0021, 0.014)
Latin America	DMS	RBD	All	0.28 (0.26, 0.3)	0.24 (0.22, 0.25)	0.042 (0.013, 0.07)
	DMS1	RBD	Cluster 1	0.17 (0.15, 0.2)	0.14 (0.12, 0.15)	0.035 (0.0033, 0.067)
	DMS2	RBD	Cluster 2	0.17 (0.15, 0.2)	0.13 (0.12, 0.15)	0.04 (0.012, 0.068)
	DMS3	RBD	Cluster 3	0.17 (0.15, 0.18)	0.15 (0.14, 0.16)	0.013 (-0.0065, 0.032)
	DMS5	RBD	Cluster 5	0.69 (0.63, 0.74)	0.6 (0.56, 0.64)	0.082 (0.017, 0.15)
	DMS6	RBD	Cluster 6	0.11 (0.098, 0.13)	0.086 (0.076, 0.096)	0.026 (0.0085, 0.043)
	DMS7	RBD	Cluster 7	0.16 (0.15, 0.17)	0.13 (0.12, 0.14)	0.03 (0.013, 0.046)
	DMS8	RBD	Cluster 8	0.096 (0.075, 0.12)	0.061 (0.047, 0.075)	0.035 (0.0093, 0.061)
	DMS9	RBD	Cluster 9	0.053 (0.043, 0.062)	0.041 (0.035, 0.047)	0.012 (0.00083, 0.023)
South Africa	DMS	RBD	All	0.37 (0.29, 0.45)	0.42 (0.39, 0.46)	-0.057 (-0.15, 0.032)
	DMS1	RBD	Cluster 1	0.38 (0.29, 0.47)	0.43 (0.39, 0.48)	-0.055 (-0.15, 0.045)
	DMS2	RBD	Cluster 2	0.33 (0.26, 0.41)	0.38 (0.34, 0.42)	-0.048 (-0.14, 0.038)
	DMS3	RBD	Cluster 3	0.21 (0.16, 0.26)	0.24 (0.22, 0.27)	-0.033 (-0.087, 0.021)
	DMS5	RBD	Cluster 5	0.74 (0.58, 0.91)	0.85 (0.77, 0.92)	-0.11 (-0.29, 0.075)
	DMS6	RBD	Cluster 6	0.083 (0.063, 0.1)	0.083 (0.067, 0.1)	-0.00076 (-0.026, 0.025)
	DMS7	RBD	Cluster 7	0.2 (0.16, 0.25)	0.25 (0.23, 0.28)	-0.052 (-0.1, 0.0006)
	DMS8	RBD	Cluster 8	0.088 (0.039, 0.14)	0.085 (0.039, 0.13)	0.0028 (-0.063, 0.069)
	DMS9	RBD	Cluster 9	0.13 (0.095, 0.16)	0.14 (0.13, 0.16)	-0.017 (-0.052, 0.017)
US	DMS	RBD	All	0.039 (0.012, 0.065)	0.016 (0.0099, 0.023)	0.022 (-0.0049, 0.049)
	DMS1	RBD	Cluster 1	0.021 (0.0033, 0.038)	0.0088 (0.0065, 0.011)	0.012 (-0.0057, 0.029)
	DMS2	RBD	Cluster 2	0.017 (0.0011, 0.033)	0.0041 (0.0028, 0.0054)	0.013 (-0.0031, 0.029)
	DMS3	RBD	Cluster 3	0.017 (0.0014, 0.032)	0.0099 (0.0044, 0.015)	0.007 (-0.0094, 0.024)
	DMS5	RBD	Cluster 5	0.072 (0.014, 0.13)	0.039 (0.018, 0.06)	0.033 (-0.029, 0.095)
	DMS6	RBD	Cluster 6	0.045 (0.024, 0.066)	0.018 (0.011, 0.026)	0.027 (0.0042, 0.049)
	DMS7	RBD	Cluster 7	0.032 (0.015, 0.049)	0.027 (0.018, 0.036)	0.0053 (-0.014, 0.024)
	DMS8	RBD	Cluster 8	0.089 (0.045, 0.13)	0.038 (0.021, 0.054)	0.051 (0.004, 0.099)
	DMS9	RBD	Cluster 9	0.0071 (0.00075, 0.014)	0.0023 (0.00027, 0.0044)	0.0048 (-0.0019, 0.012)

Supplementary Table 15.
Among COVID-19 primary endpoint cases, point estimates of means (95% confidence intervals) of deep mutational scanning (DMS) antibody escape score of the disease-causing SARS-CoV-2 isolate by treatment arm, and of mean differences (vaccine - placebo). Inverse probability weighting was used to accommodate missing SARS-CoV-2 AA sequences by modeling the probability of observing a sequence dependent on log10 viral load and region. Results are shown for each geographic region and for all geographic regions pooled.

Geographic Region	Feature Name	Spike Region	Antibody Set	Vaccine	Placebo	Difference
Pooled	DMS	RBD	All	0.25 (0.23, 0.27)	0.19 (0.17, 0.2)	0.061 (0.037, 0.085)
	DMS1	RBD	Cluster 1	0.17 (0.15, 0.19)	0.12 (0.11, 0.14)	0.048 (0.023, 0.073)
	DMS2	RBD	Cluster 2	0.17 (0.15, 0.18)	0.12 (0.1, 0.13)	0.05 (0.028, 0.073)
	DMS3	RBD	Cluster 3	0.15 (0.13, 0.16)	0.12 (0.11, 0.13)	0.028 (0.012, 0.044)
	DMS5	RBD	Cluster 5	0.58 (0.53, 0.63)	0.45 (0.42, 0.48)	0.13 (0.073, 0.19)
	DMS6	RBD	Cluster 6	0.098 (0.088, 0.11)	0.066 (0.059, 0.073)	0.033 (0.02, 0.045)
	DMS7	RBD	Cluster 7	0.14 (0.13, 0.15)	0.11 (0.1, 0.12)	0.033 (0.019, 0.048)
	DMS8	RBD	Cluster 8	0.096 (0.079, 0.11)	0.057 (0.046, 0.069)	0.039 (0.018, 0.059)
	DMS9	RBD	Cluster 9	0.054 (0.047, 0.061)	0.038 (0.033, 0.043)	0.016 (0.0072, 0.025)
Latin America	DMS	RBD	All	0.28 (0.26, 0.3)	0.24 (0.22, 0.25)	0.042 (0.014, 0.07)
	DMS1	RBD	Cluster 1	0.17 (0.15, 0.2)	0.14 (0.12, 0.16)	0.035 (0.0038, 0.066)
	DMS2	RBD	Cluster 2	0.17 (0.15, 0.2)	0.13 (0.12, 0.15)	0.04 (0.013, 0.067)
	DMS3	RBD	Cluster 3	0.17 (0.15, 0.18)	0.15 (0.14, 0.16)	0.012 (-0.007, 0.032)
	DMS5	RBD	Cluster 5	0.69 (0.63, 0.74)	0.6 (0.56, 0.64)	0.083 (0.017, 0.15)
	DMS6	RBD	Cluster 6	0.11 (0.1, 0.13)	0.086 (0.076, 0.096)	0.029 (0.012, 0.046)
	DMS7	RBD	Cluster 7	0.16 (0.15, 0.17)	0.13 (0.12, 0.14)	0.03 (0.013, 0.046)
	DMS8	RBD	Cluster 8	0.097 (0.077, 0.12)	0.061 (0.046, 0.075)	0.037 (0.012, 0.062)
	DMS9	RBD	Cluster 9	0.053 (0.044, 0.061)	0.041 (0.035, 0.047)	0.012 (0.00099, 0.022)
South Africa	DMS	RBD	All	0.38 (0.33, 0.42)	0.42 (0.38, 0.46)	-0.04 (-0.1, 0.024)
	DMS1	RBD	Cluster 1	0.38 (0.33, 0.44)	0.42 (0.37, 0.47)	-0.035 (-0.11, 0.04)
	DMS2	RBD	Cluster 2	0.34 (0.29, 0.39)	0.37 (0.33, 0.41)	-0.032 (-0.097, 0.033)
	DMS3	RBD	Cluster 3	0.22 (0.18, 0.25)	0.24 (0.21, 0.26)	-0.023 (-0.062, 0.016)
	DMS5	RBD	Cluster 5	0.73 (0.63, 0.84)	0.83 (0.74, 0.91)	-0.091 (-0.22, 0.042)
	DMS6	RBD	Cluster 6	0.087 (0.065, 0.11)	0.089 (0.072, 0.11)	-0.0022 (-0.03, 0.025)
	DMS7	RBD	Cluster 7	0.21 (0.17, 0.24)	0.24 (0.22, 0.27)	-0.036 (-0.076, 0.0038)
	DMS8	RBD	Cluster 8	0.096 (0.039, 0.15)	0.096 (0.05, 0.14)	0.00045 (-0.073, 0.074)
	DMS9	RBD	Cluster 9	0.13 (0.11, 0.15)	0.14 (0.12, 0.16)	-0.011 (-0.037, 0.015)
US	DMS	RBD	All	0.034 (0.02, 0.048)	0.017 (0.0094, 0.024)	0.017 (0.001, 0.033)
	DMS1	RBD	Cluster 1	0.017 (0.0093, 0.025)	0.0087 (0.0048, 0.013)	0.0082 (-0.00037, 0.017)
	DMS2	RBD	Cluster 2	0.014 (0.0075, 0.02)	0.0042 (0.001, 0.0074)	0.0094 (0.0025, 0.016)
	DMS3	RBD	Cluster 3	0.018 (0.0065, 0.029)	0.01 (0.004, 0.016)	0.0079 (-0.005, 0.021)
	DMS5	RBD	Cluster 5	0.076 (0.032, 0.12)	0.039 (0.017, 0.062)	0.036 (-0.013, 0.086)
	DMS6	RBD	Cluster 6	0.044 (0.028, 0.06)	0.019 (0.011, 0.027)	0.025 (0.0073, 0.043)
	DMS7	RBD	Cluster 7	0.03 (0.013, 0.046)	0.027 (0.018, 0.035)	0.003 (-0.015, 0.021)
	DMS8	RBD	Cluster 8	0.09 (0.055, 0.12)	0.039 (0.021, 0.057)	0.051 (0.011, 0.09)
	DMS9	RBD	Cluster 9	0.0062 (0.0017, 0.011)	0.0024 (-1.6e-06, 0.0048)	0.0039 (-0.0013, 0.009)

Supplementary Table 16. For Protein Data Bank (PDB) antibody escape score and VE against the primary COVID-19 endpoint, p-values from the Juraska and Gilbert[54] one sided weighted Wald test of $H_{10} : VE(v) = 0$ for all v and from double one-sided Wald test of $H_{20} : VE(v) = VE$ for all v against H_{21} : $VE(v)$ decreases with v. Family-wise error rate (FWER) adjusted p-values are Holm-Bonferroni and FDR-adjusted p-values (q-values) are Benjamini-Hochberg.

Geographic Region	Feature Name	Spike Region	PDB Antibody Escape Score	Test of H_{10} p-value	Test of H_{20} p-value	FWER p-value	q-value
Pooled	PDB1	RBD	Cluster 1¶§†	<0.001	<0.001	0.0022	<0.001
	PDB2	RBD	Cluster 2¶§†	<0.001	<0.001	<0.001	<0.001
	PDB3	RBD	Cluster 3¶§†	<0.001	<0.001	0.0045	0.0014
	PDB4	RBD	Cluster 4¶§†	<0.001	0.0041	0.021	0.0065
	PDB5	RBD	Cluster 5	<0.001	0.17	0.69	0.24
	PDB6	RBD	Cluster 6	<0.001	0.94	1.00	1.00
	PDB7	RBD	Cluster 7¶§†	<0.001	<0.001	<0.001	<0.001
	PDB8	RBD	Cluster 8¶§†	<0.001	<0.001	<0.001	<0.001
	PDB9	RBD	Cluster 9	<0.001	1.00	1.00	1.00
	PDB13	NTD	Cluster 13¶§†	<0.001	<0.001	<0.001	<0.001
	PDB14	NTD	Cluster 14	<0.001	0.60	1.00	0.74
Latin America	PDB1	RBD	Cluster 1¶§	<0.001	0.012	0.073	0.022
	PDB2	RBD	Cluster 2	<0.001	0.21	1.00	0.33
	PDB3	RBD	Cluster 3¶§	<0.001	0.01	0.072	0.022
	PDB4	RBD	Cluster 4¶§†	<0.001	0.0026	0.021	0.0071
	PDB5	RBD	Cluster 5	<0.001	0.37	1.00	0.51
	PDB6	RBD	Cluster 6	<0.001	1.00	1.00	1.00
	PDB7	RBD	Cluster 7¶§†	<0.001	<0.001	0.008	0.0033
	PDB8	RBD	Cluster 8¶§†	<0.001	<0.001	<0.001	<0.001
	PDB9	RBD	Cluster 9	<0.001	1.00	1.00	1.00
	PDB13	NTD	Cluster 13¶§†	<0.001	<0.001	0.0047	0.0026
	PDB14	NTD	Cluster 14	<0.001	0.74	1.00	0.90
South Africa	PDB1	RBD	Cluster 1	0.0014	1.00	1.00	1.00
	PDB2	RBD	Cluster 2	<0.001	0.51	1.00	1.00
	PDB4	RBD	Cluster 4	0.0013	1.00	1.00	1.00
	PDB7	RBD	Cluster 7	<0.001	1.00	1.00	1.00
	PDB8	RBD	Cluster 8	0.0011	1.00	1.00	1.00
	PDB14	NTD	Cluster 14	0.0011	1.00	1.00	1.00
US	PDB1	RBD	Cluster 1	<0.001	0.58	1.00	0.68
	PDB2	RBD	Cluster 2	<0.001	0.063	0.38	0.22
	PDB4	RBD	Cluster 4	<0.001	0.18	0.90	0.38
	PDB7	RBD	Cluster 7	<0.001	0.40	1.00	0.57
	PDB8	RBD	Cluster 8¶§	<0.001	0.0096	0.067	0.067
	PDB13	NTD	Cluster 13	<0.001	0.22	0.90	0.38
	PDB14	NTD	Cluster 14	<0.001	0.94	1.00	0.94

¶ Unadjusted p-value for differential VE is ≤ 0.05.
† FWER-adjusted p-value for differential VE is ≤ 0.05.
§ FDR-adjusted p-value (q-value) for differential VE is ≤ 0.2 and unadjusted p-value for the differential VE is ≤ 0.05.

Supplementary Table 17.
Among COVID-19 primary endpoint cases, point estimates of means (95% confidence intervals) of Protein Data Bank (PDB) antibody escape score of the disease-causing SARS-CoV-2 isolate by treatment arm, and of mean differences (vaccine - placebo). Doubly robust targeted minimum loss-based estimation was used to adjust for baseline covariates (the indicators of each biweekly calendar period of the enrollment date, and, for the geographic region-pooled analysis, also indicators representing the geographic regions [US, South Africa, Colombia, Latin America excluding Colombia]; the Latin America analysis also included the indicator of Colombia) and to bias-correct by modeling the probability of observing a sequence dependent on log10 viral load. Results are shown for each geographic region and for all geographic regions pooled.

Geographic Region	Feature Name	Spike Region	Antibody Set	Vaccine	Placebo	Difference (Vaccine – Placebo)
Pooled	PDB1	RBD	Cluster 1	0.21 (0.18, 0.24)	0.18 (0.17, 0.2)	0.024 (-0.0065, 0.055)
	PDB2	RBD	Cluster 2	0.1 (0.092, 0.11)	0.094 (0.087, 0.1)	0.0092 (-0.0032, 0.021)
	PDB3	RBD	Cluster 3	0.38 (0.33, 0.43)	0.34 (0.3, 0.37)	0.043 (-0.01, 0.097)
	PDB4	RBD	Cluster 4	0.056 (0.047, 0.065)	0.04 (0.034, 0.047)	0.015 (0.0043, 0.026)
	PDB5	RBD	Cluster 5	0.034 (0.025, 0.042)	0.032 (0.025, 0.039)	0.0018 (-0.0082, 0.012)
	PDB6	RBD	Cluster 6	0.012 (0.0073, 0.017)	0.014 (0.009, 0.018)	-0.0016 (-0.0079, 0.0047)
	PDB7	RBD	Cluster 7	0.1 (0.087, 0.11)	0.076 (0.067, 0.086)	0.025 (0.009, 0.04)
	PDB8	RBD	Cluster 8	0.35 (0.32, 0.38)	0.29 (0.27, 0.31)	0.06 (0.03, 0.091)
	PDB9	RBD	Cluster 9	0.014 (0.0093, 0.018)	0.017 (0.014, 0.021)	-0.0036 (-0.009, 0.0018)
	PDB13	NTD	Cluster 13	0.89 (0.68, 1.09)	0.61 (0.5, 0.73)	0.27 (0.043, 0.5)
	PDB14	NTD	Cluster 14	0.072 (0.051, 0.093)	0.074 (0.06, 0.087)	-0.0017 (-0.026, 0.023)
Latin America	PDB1	RBD	Cluster 1	0.24 (0.21, 0.28)	0.19 (0.16, 0.21)	0.054 (0.012, 0.097)
	PDB2	RBD	Cluster 2	0.12 (0.1, 0.13)	0.1 (0.095, 0.11)	0.011 (-0.0048, 0.027)
	PDB3	RBD	Cluster 3	0.45 (0.39, 0.51)	0.35 (0.31, 0.39)	0.094 (0.022, 0.17)
	PDB4	RBD	Cluster 4	0.076 (0.063, 0.089)	0.053 (0.044, 0.061)	0.024 (0.0084, 0.039)
	PDB5	RBD	Cluster 5	0.057 (0.044, 0.071)	0.049 (0.039, 0.06)	0.0081 (-0.0078, 0.024)
	PDB6	RBD	Cluster 6	0.019 (0.012, 0.027)	0.02 (0.013, 0.027)	-0.00052 (-0.01, 0.0093)
	PDB7	RBD	Cluster 7	0.15 (0.13, 0.17)	0.11 (0.094, 0.12)	0.039 (0.015, 0.063)
	PDB8	RBD	Cluster 8	0.44 (0.4, 0.47)	0.35 (0.33, 0.38)	0.084 (0.042, 0.13)
	PDB9	RBD	Cluster 9	0.015 (0.011, 0.019)	0.023 (0.018, 0.028)	-0.0084 (-0.015, -0.0022)
	PDB13	NTD	Cluster 13	1.40 (1.08, 1.72)	0.79 (0.61, 0.97)	0.61 (0.25, 0.97)
	PDB14	NTD	Cluster 14	0.063 (0.037, 0.09)	0.047 (0.03, 0.065)	0.016 (-0.016, 0.048)
South Africa	PDB1	RBD	Cluster 1	0.48 (0.37, 0.59)	0.54 (0.48, 0.59)	-0.053 (-0.18, 0.071)
	PDB2	RBD	Cluster 2	0.24 (0.2, 0.28)	0.25 (0.23, 0.26)	-0.0042 (-0.045, 0.037)
	PDB4	RBD	Cluster 4	0.019 (0.0084, 0.031)	0.041 (0.0057, 0.076)	-0.021 (-0.058, 0.015)
	PDB7	RBD	Cluster 7	0.043 (0.036, 0.05)	0.068 (0.049, 0.088)	-0.025 (-0.046, -0.0045)
	PDB8	RBD	Cluster 8	0.48 (0.39, 0.57)	0.56 (0.53, 0.59)	-0.079 (-0.18, 0.019)
	PDB14	NTD	Cluster 14	0.23 (0.17, 0.28)	0.29 (0.25, 0.34)	-0.065 (-0.14, 0.0082)
US	PDB1	RBD	Cluster 1	0.032 (0.0049, 0.06)	0.03 (0.018, 0.042)	0.0021 (-0.028, 0.032)
	PDB2	RBD	Cluster 2	0.026 (0.0062, 0.047)	0.011 (0.0045, 0.017)	0.016 (-0.0054, 0.037)
	PDB4	RBD	Cluster 4	0.024 (0.011, 0.038)	0.011 (0.0057, 0.017)	0.013 (-0.0018, 0.027)
	PDB7	RBD	Cluster 7	0.0098 (0.0048, 0.015)	0.0058 (0.0025, 0.0091)	0.004 (-0.0019, 0.01)
	PDB8	RBD	Cluster 8	0.092 (0.048, 0.14)	0.037 (0.023, 0.051)	0.055 (0.009, 0.1)
	PDB13	NTD	Cluster 13	0.11 (0.067, 0.16)	0.079 (0.056, 0.1)	0.036 (-0.017, 0.09)
	PDB14	NTD	Cluster 14	0.049 (0.009, 0.089)	0.049 (0.029, 0.07)	-0.00019 (-0.045, 0.045)

Supplementary Table 18.
Among COVID-19 primary endpoint cases, point estimates of means (95% confidence intervals) of Protein Data Bank (PDB) antibody escape score of the disease-causing SARS-CoV-2 isolate by treatment arm, and of mean differences (vaccine - placebo). Inverse probability weighting was used to accommodate missing SARS-CoV-2 AA sequences by modeling the probability of observing a sequence dependent on log10 viral load and region. Results are shown for each geographic region and for all geographic regions pooled.

Geographic Region	Feature Name	Spike Region	PDB Antibody Escape Score	Vaccine	Placebo	Difference
Pooled	PDB1	RBD	Cluster 1	0.23 (0.2, 0.26)	0.17 (0.15, 0.19)	0.063 (0.03, 0.096)
	PDB2	RBD	Cluster 2	0.11 (0.1, 0.13)	0.088 (0.08, 0.096)	0.027 (0.013, 0.04)
	PDB3	RBD	Cluster 3	0.42 (0.37, 0.47)	0.32 (0.28, 0.35)	0.1 (0.045, 0.16)
	PDB4	RBD	Cluster 4	0.06 (0.05, 0.07)	0.039 (0.033, 0.046)	0.021 (0.009, 0.032)
	PDB5	RBD	Cluster 5	0.04 (0.029, 0.05)	0.031 (0.023, 0.038)	0.0091 (-0.0036, 0.022)
	PDB6	RBD	Cluster 6	0.013 (0.0069, 0.02)	0.013 (0.0087, 0.017)	0.00028 (-0.0073, 0.0079)
	PDB7	RBD	Cluster 7	0.11 (0.098, 0.13)	0.073 (0.063, 0.083)	0.039 (0.021, 0.057)
	PDB8	RBD	Cluster 8	0.38 (0.35, 0.41)	0.27 (0.25, 0.29)	0.11 (0.073, 0.14)
	PDB9	RBD	Cluster 9	0.015 (0.01, 0.019)	0.016 (0.013, 0.019)	-0.0019 (-0.0072, 0.0035)
	PDB13	NTD	Cluster 13	0.98 (0.79, 1.17)	0.51 (0.38, 0.64)	0.47 (0.24, 0.7)
	PDB14	NTD	Cluster 14	0.077 (0.057, 0.097)	0.069 (0.056, 0.082)	0.0082 (-0.016, 0.032)
Latin America	PDB1	RBD	Cluster 1	0.24 (0.21, 0.28)	0.19 (0.16, 0.21)	0.054 (0.012, 0.096)
	PDB2	RBD	Cluster 2	0.11 (0.1, 0.13)	0.1 (0.095, 0.11)	0.01 (-0.0054, 0.026)
	PDB3	RBD	Cluster 3	0.45 (0.39, 0.51)	0.35 (0.31, 0.39)	0.098 (0.026, 0.17)
	PDB4	RBD	Cluster 4	0.077 (0.065, 0.089)	0.052 (0.043, 0.061)	0.025 (0.0095, 0.04)
	PDB5	RBD	Cluster 5	0.058 (0.042, 0.073)	0.049 (0.038, 0.06)	0.0092 (-0.0098, 0.028)
	PDB6	RBD	Cluster 6	0.019 (0.01, 0.028)	0.02 (0.013, 0.027)	-0.00065 (-0.012, 0.011)
	PDB7	RBD	Cluster 7	0.15 (0.13, 0.17)	0.11 (0.093, 0.12)	0.045 (0.019, 0.07)
	PDB8	RBD	Cluster 8	0.44 (0.41, 0.47)	0.35 (0.33, 0.38)	0.086 (0.044, 0.13)
	PDB9	RBD	Cluster 9	0.015 (0.0088, 0.021)	0.023 (0.019, 0.027)	-0.0081 (-0.016, -0.0006)
	PDB13	NTD	Cluster 13	1.39 (1.11, 1.67)	0.78 (0.58, 0.98)	0.61 (0.26, 0.95)
	PDB14	NTD	Cluster 14	0.055 (0.031, 0.079)	0.048 (0.031, 0.066)	0.0067 (-0.023, 0.037)
South Africa	PDB1	RBD	Cluster 1	0.47 (0.4, 0.54)	0.52 (0.46, 0.58)	-0.052 (-0.15, 0.041)
	PDB2	RBD	Cluster 2	0.25 (0.23, 0.27)	0.24 (0.23, 0.26)	0.0073 (-0.021, 0.036)
	PDB4	RBD	Cluster 4	0.022 (-0.014, 0.057)	0.046 (0.017, 0.074)	-0.024 (-0.07, 0.022)
	PDB7	RBD	Cluster 7	0.044 (0.025, 0.063)	0.071 (0.055, 0.087)	-0.027 (-0.052, -0.002)
	PDB8	RBD	Cluster 8	0.49 (0.45, 0.54)	0.54 (0.51, 0.58)	-0.05 (-0.11, 0.0096)
	PDB14	NTD	Cluster 14	0.24 (0.18, 0.29)	0.29 (0.24, 0.33)	-0.051 (-0.12, 0.016)
US	PDB1	RBD	Cluster 1	0.029 (0.007, 0.052)	0.029 (0.017, 0.041)	0.0005 (-0.025, 0.026)
	PDB2	RBD	Cluster 2	0.022 (0.0083, 0.036)	0.012 (0.0046, 0.019)	0.01 (-0.0051, 0.026)
	PDB4	RBD	Cluster 4	0.023 (0.012, 0.035)	0.012 (0.0058, 0.018)	0.011 (-0.0018, 0.025)
	PDB7	RBD	Cluster 7	0.01 (0.004, 0.016)	0.006 (0.0028, 0.0092)	0.0042 (-0.0028, 0.011)
	PDB8	RBD	Cluster 8	0.087 (0.057, 0.12)	0.038 (0.023, 0.053)	0.049 (0.016, 0.082)
	PDB13	NTD	Cluster 13	0.11 (0.072, 0.16)	0.077 (0.055, 0.1)	0.037 (-0.011, 0.086)
	PDB14	NTD	Cluster 14	0.049 (0.012, 0.086)	0.046 (0.027, 0.066)	0.0028 (-0.039, 0.045)

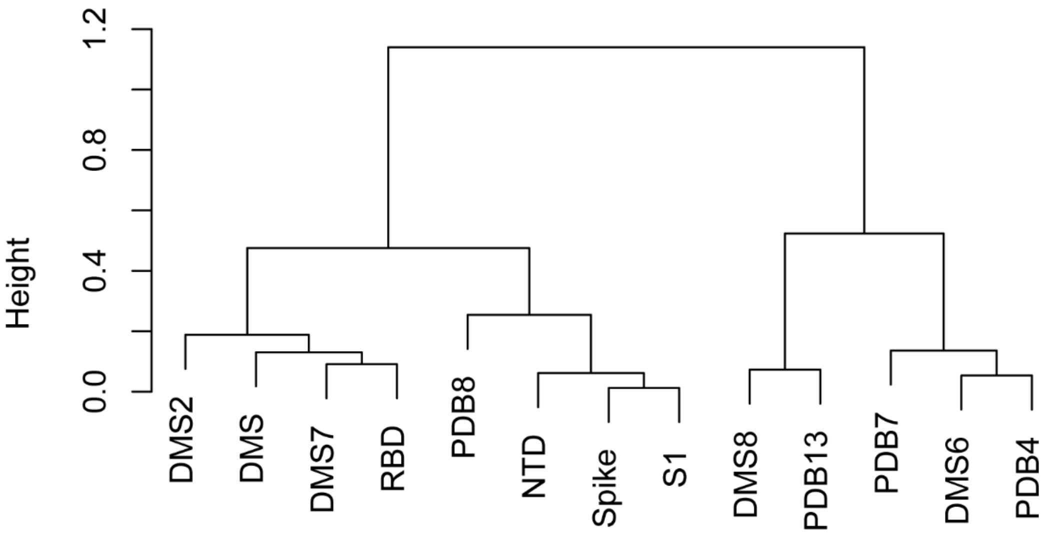

Supplementary Fig. 23
For the Latin America cohort, hierarchical clustering of physicochemical-weighted Hamming distance, DMS, and PDB sequence features. DMS, deep mutational scanning; PDB, Protein Data Bank.

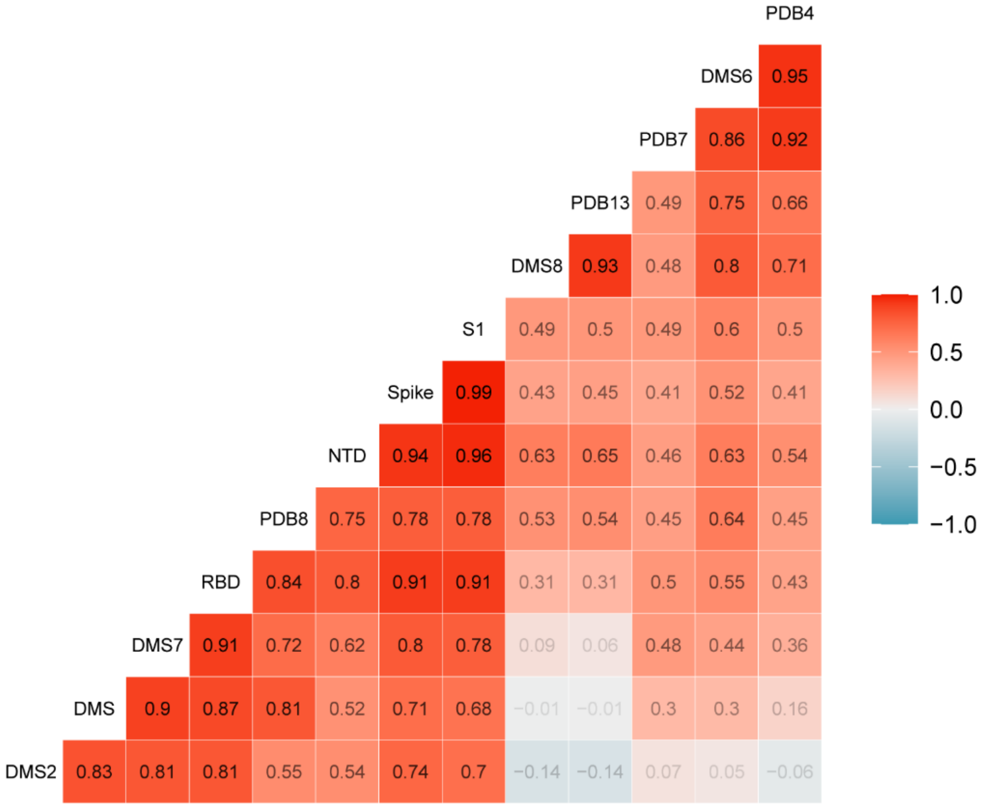

Supplementary Fig. 24

For the Latin America cohort, correlation matrix for physicochemical-weighted Hamming distance, DMS, and PDB sequence features ordered by the hierarchical clustering shown in Supplementary Fig. 23. Numbers shown are Spearman correlation coefficients. The matrix restricts to physicochemical-weighted Hamming distance sequence features that have FWER≤0.05, so it excludes S2.

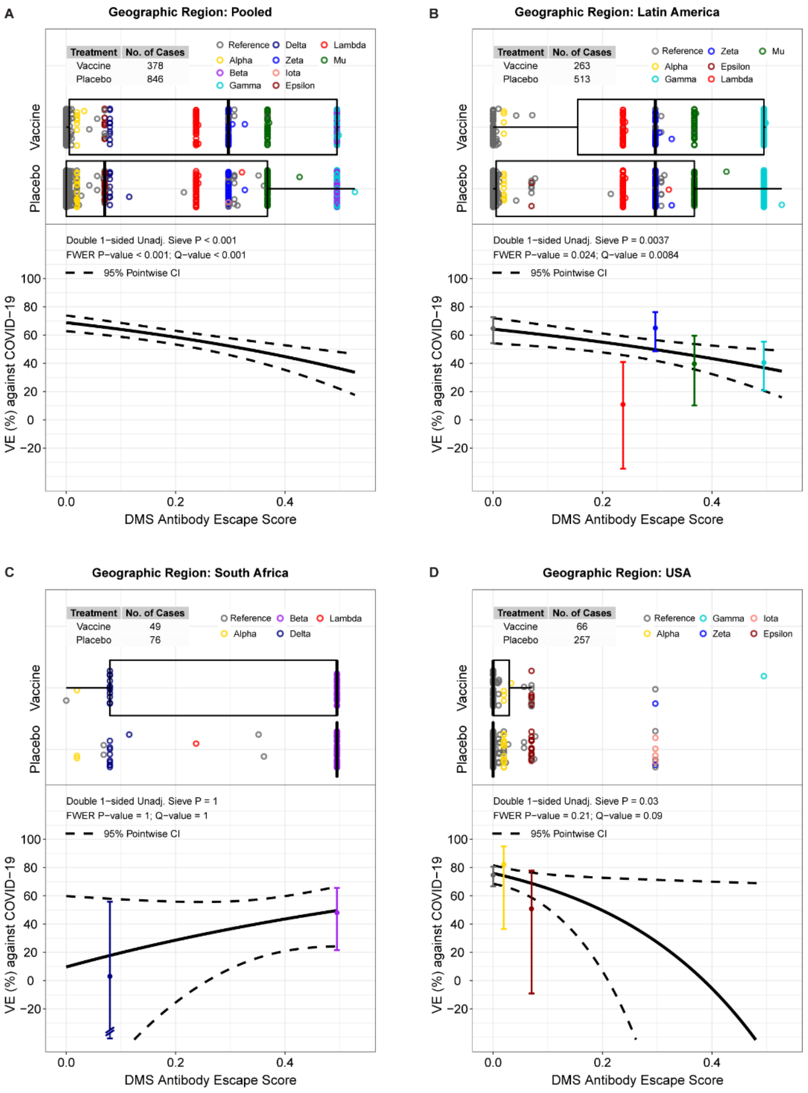

Supplementary Fig. 25

Hazard-based vaccine efficacy against the primary COVID-19 endpoint by the DMS antibody escape score of the disease-causing SARS-CoV-2 isolate. A) Geographic regions pooled, B) Latin America, C) South Africa, D) US. Dashed lines are 95% pointwise confidence intervals. The top plot in each panel shows the distributions of DMS antibody escape scores by treatment arm, color-coded by lineage of the disease-causing SARS-CoV-2 isolate. The left and right edges of the box plots represent the 25th and 75th percentiles of DMS antibody escape score and the vertical middle line represents the 50th percentile. The horizontal bars extend from the 25th (or 75th) percentile of DMS antibody escape score to the minimum (or maximum) DMS antibody escape score within the 25th (or 75th) percentile of DMS antibody escape score minus (or plus) 1.5 times the interquartile range. In each panel, the "Double 1-sided unadjusted sieve p-value" doubles the p-value from a one-sided Wald test of the null hypothesis of constant VE vs. the alternative hypothesis of a decreasing VE with an increasing value of the feature on the x-axis (Juraska and Gilbert,[53] Section 5).

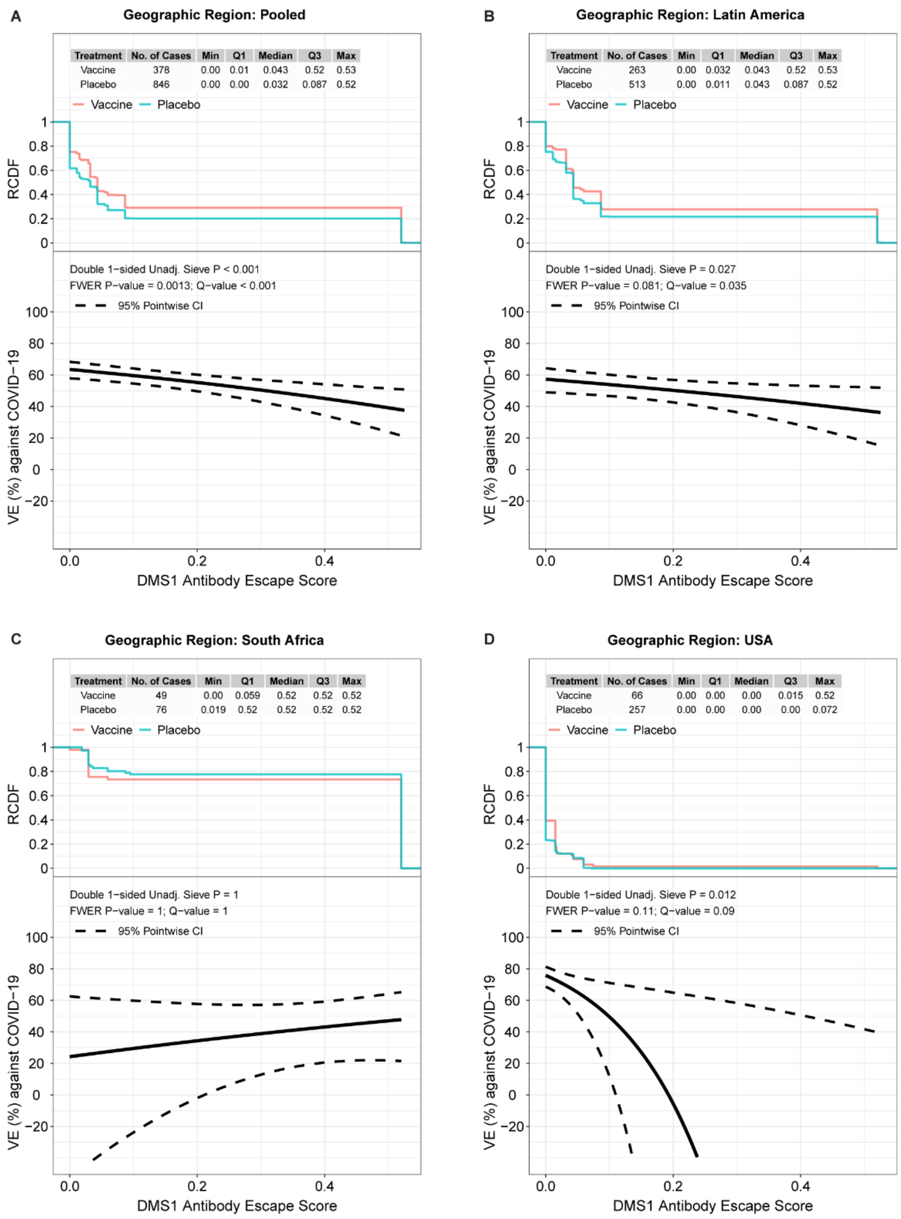

Supplementary Fig. 26

Hazard-based vaccine efficacy against the primary COVID-19 endpoint by the DMS1 antibody escape score of the disease-causing SARS-CoV-2 isolate. A) Geographic regions pooled, B) Latin America, C) South Africa, D) US. Dashed lines are 95% pointwise confidence intervals. The "Double 1-sided unadjusted sieve p-value" doubles the p-value from a one-sided Wald test of the null hypothesis of constant VE vs. the alternative hypothesis of a decreasing VE with an increasing value of the feature on the x-axis (Juraska and Gilbert, [53] Section 5). The plot at the top of each panel shows the reverse cumulative distribution function (RCDF) of the DMS1 antibody-binding escape score across SARS-CoV-2 isolates by treatment arm: Vaccine, pink; Placebo, turquoise.

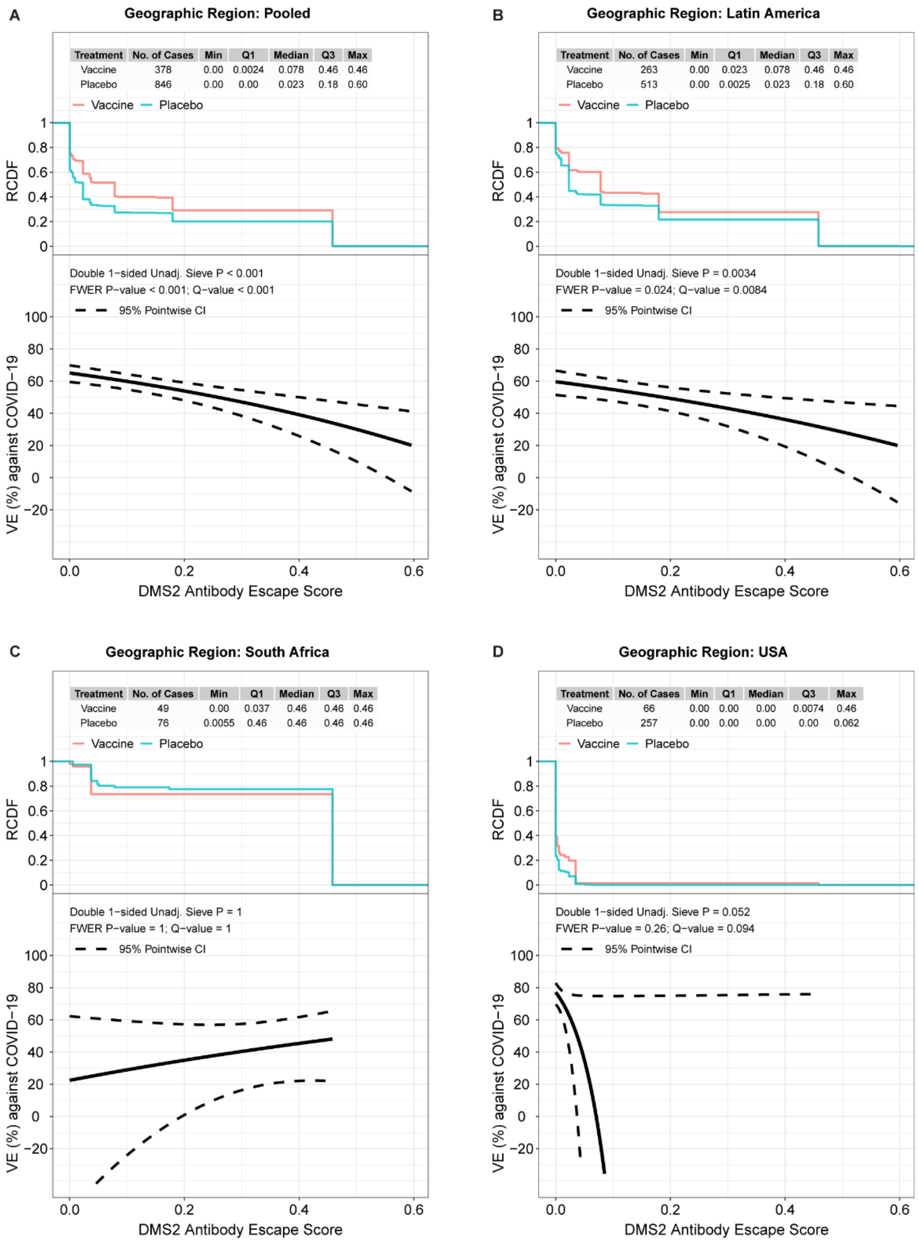

Supplementary Fig. 27

Hazard-based vaccine efficacy against the COVID-19 primary endpoint by the DMS2 antibody escape score of the disease-causing SARS-CoV-2 isolate. A) Geographic regions pooled, B) Latin America, C) South Africa, D) US. Dashed lines are 95% pointwise confidence intervals. The "Double 1-sided unadjusted sieve p-value" doubles the p-value from a one-sided Wald test of the null hypothesis of constant VE vs. the alternative hypothesis of a decreasing VE with an increasing value of the feature on the x-axis (Juraska and Gilbert, [53] Section 5). The plot at the top of each panel shows the reverse cumulative distribution function (RCDF) of the DMS2 antibody-binding escape score across SARS-CoV-2 isolates by treatment arm: Vaccine, pink; Placebo, turquoise.

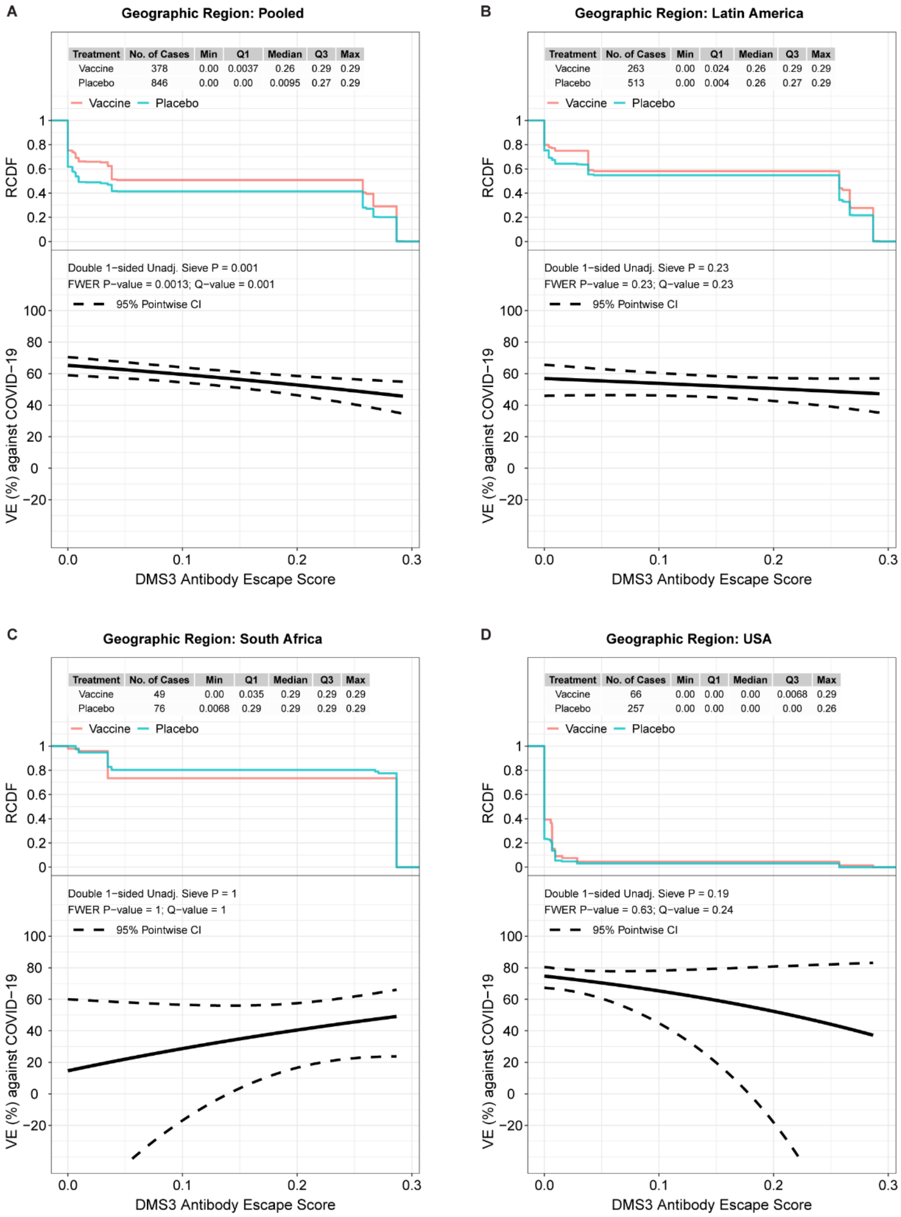

Supplementary Fig. 28

Hazard-based vaccine efficacy against the COVID-19 primary endpoint by the DMS3 antibody escape score of the disease-causing SARS-CoV-2 isolate. A) Geographic regions pooled, B) Latin America, C) South Africa, D) US. Dashed lines are 95% pointwise confidence intervals. The "Double 1-sided unadjusted sieve p-value" doubles the p-value from a one-sided Wald test of the null hypothesis of constant VE vs. the alternative hypothesis of a decreasing VE with an increasing value of the feature on the x-axis (Juraska and Gilbert, [53] Section 5). The plot at the top of each panel shows the reverse cumulative distribution function (RCDF) of the DMS3 antibody-binding escape score across SARS-CoV-2 isolates by treatment arm: Vaccine, pink; Placebo, turquoise.

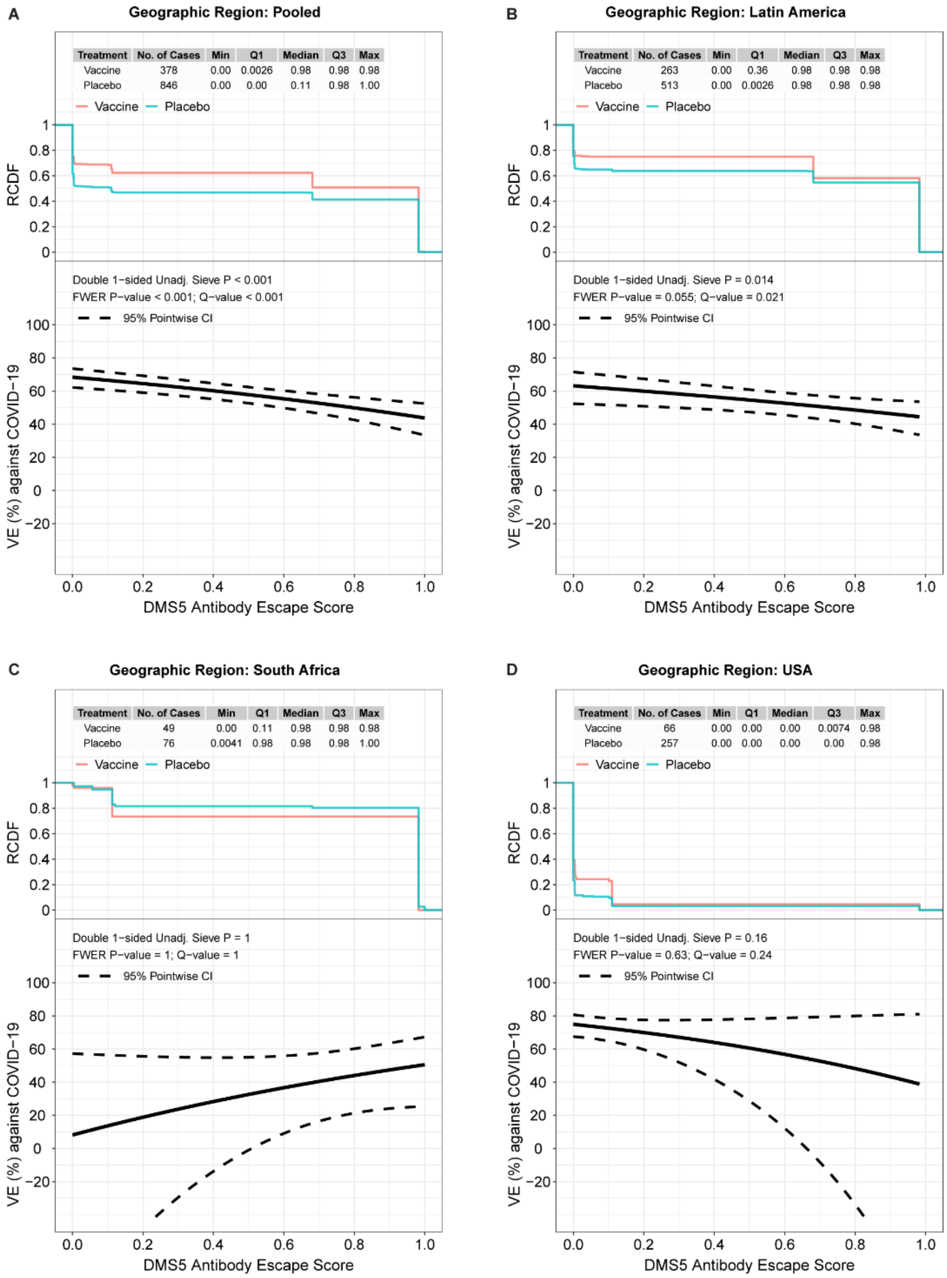

Supplementary Fig. 29

Hazard-based vaccine efficacy against the COVID-19 primary endpoint by the DMS5 antibody escape score of the disease-causing SARS-CoV-2 isolate. A) Geographic regions pooled, B) Latin America, C) South Africa, D) US. Dashed lines are 95% pointwise confidence intervals. The "Double 1-sided unadjusted sieve p-value" doubles the p-value from a one-sided Wald test of the null hypothesis of constant VE vs. the alternative hypothesis of a decreasing VE with an increasing value of the feature on the x-axis (Juraska and Gilbert, [53] Section 5). The plot at the top of each panel shows the reverse cumulative distribution function (RCDF) of the DMS5 antibody-binding escape score across SARS-CoV-2 isolates by treatment arm: Vaccine, pink; Placebo, turquoise.

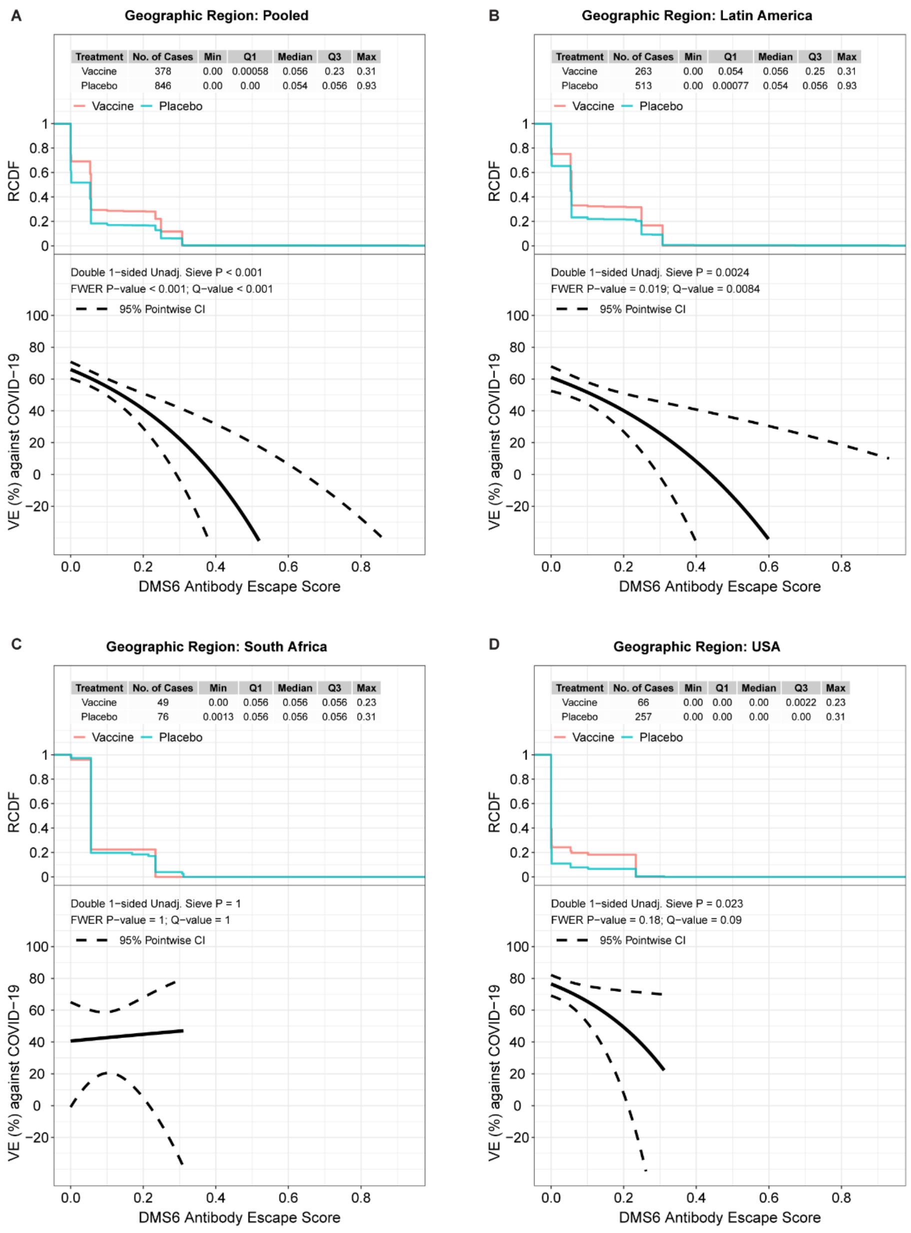

Supplementary Fig. 30

Hazard-based vaccine efficacy against the COVID-19 primary endpoint by the DMS6 antibody escape score of the disease-causing SARS-CoV-2 isolate. A) Geographic regions pooled, B) Latin America, C) South Africa, D) US. Dashed lines are 95% pointwise confidence intervals. The “Double 1-sided unadjusted sieve p-value” doubles the p-value from a one-sided Wald test of the null hypothesis of constant VE vs. the alternative hypothesis of a decreasing VE with an increasing value of the feature on the x-axis (Juraska and Gilbert, [53] Section 5). The plot at the top of each panel shows the reverse cumulative distribution function (RCDF) of the DMS6 antibody-binding escape score across SARS-CoV-2 isolates by treatment arm: Vaccine, pink; Placebo, turquoise.

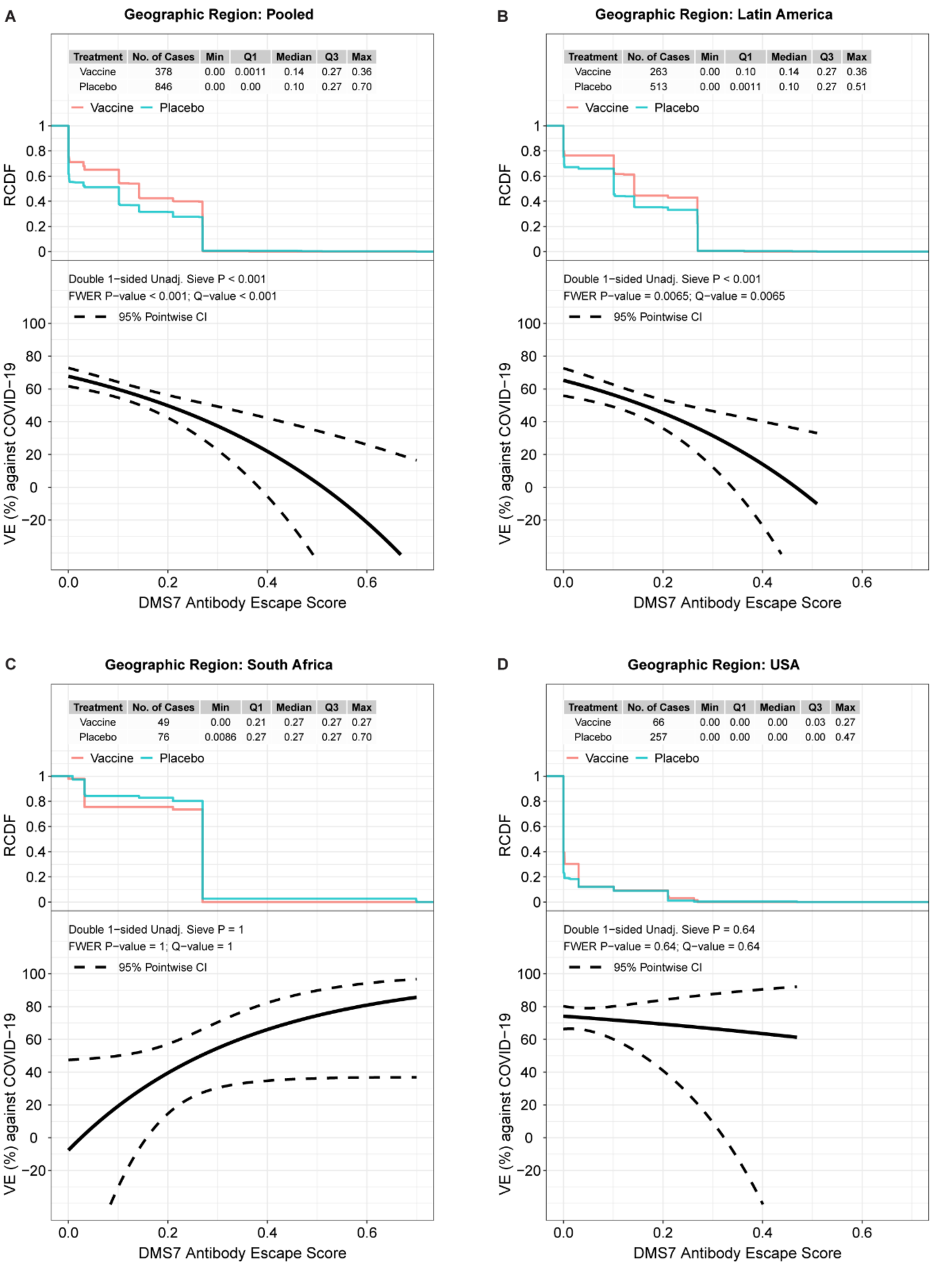

Supplementary Fig. 31

Hazard-based vaccine efficacy against the COVID-19 primary endpoint by the DMS7 antibody escape score of the disease-causing SARS-CoV-2 isolate. A) Geographic regions pooled, B) Latin America, C) South Africa, D) US. Dashed lines are 95% pointwise confidence intervals. The "Double 1-sided unadjusted sieve p-value" doubles the p-value from a one-sided Wald test of the null hypothesis of constant VE vs. the alternative hypothesis of a decreasing VE with an increasing value of the feature on the x-axis (Juraska and Gilbert, [53] Section 5). The plot at the top of each panel shows the reverse cumulative distribution function (RCDF) of the DMS7 antibody-binding escape score across SARS-CoV-2 isolates by treatment arm: Vaccine, pink; Placebo, turquoise.

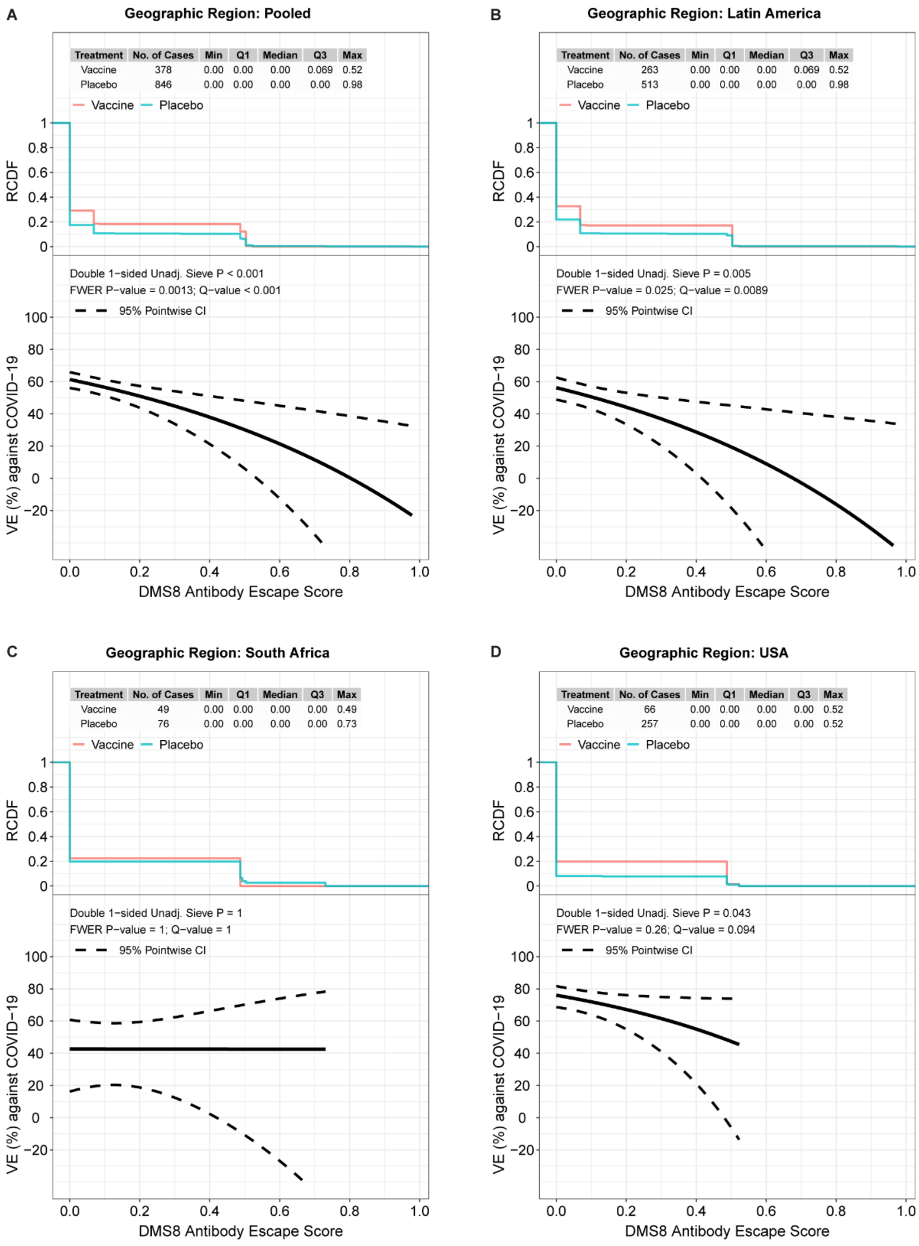

Supplementary Fig. 32

Hazard-based vaccine efficacy against the COVID-19 primary endpoint by the DMS8 antibody escape score of the disease-causing SARS-CoV-2 isolate. Dashed lines are 95% pointwise confidence intervals. A) Geographic regions pooled, B) Latin America, C) South Africa, D) US. The “Double 1-sided unadjusted sieve p-value” doubles the p-value from a one-sided Wald test of the null hypothesis of constant VE vs. the alternative hypothesis of a decreasing VE with an increasing value of the feature on the x-axis (Juraska and Gilbert, [53] Section 5). The plot at the top of each panel shows the reverse cumulative distribution function (RCDF) of the DMS8 antibody-binding escape score across SARS-CoV-2 isolates by treatment arm: Vaccine, pink; Placebo, turquoise.

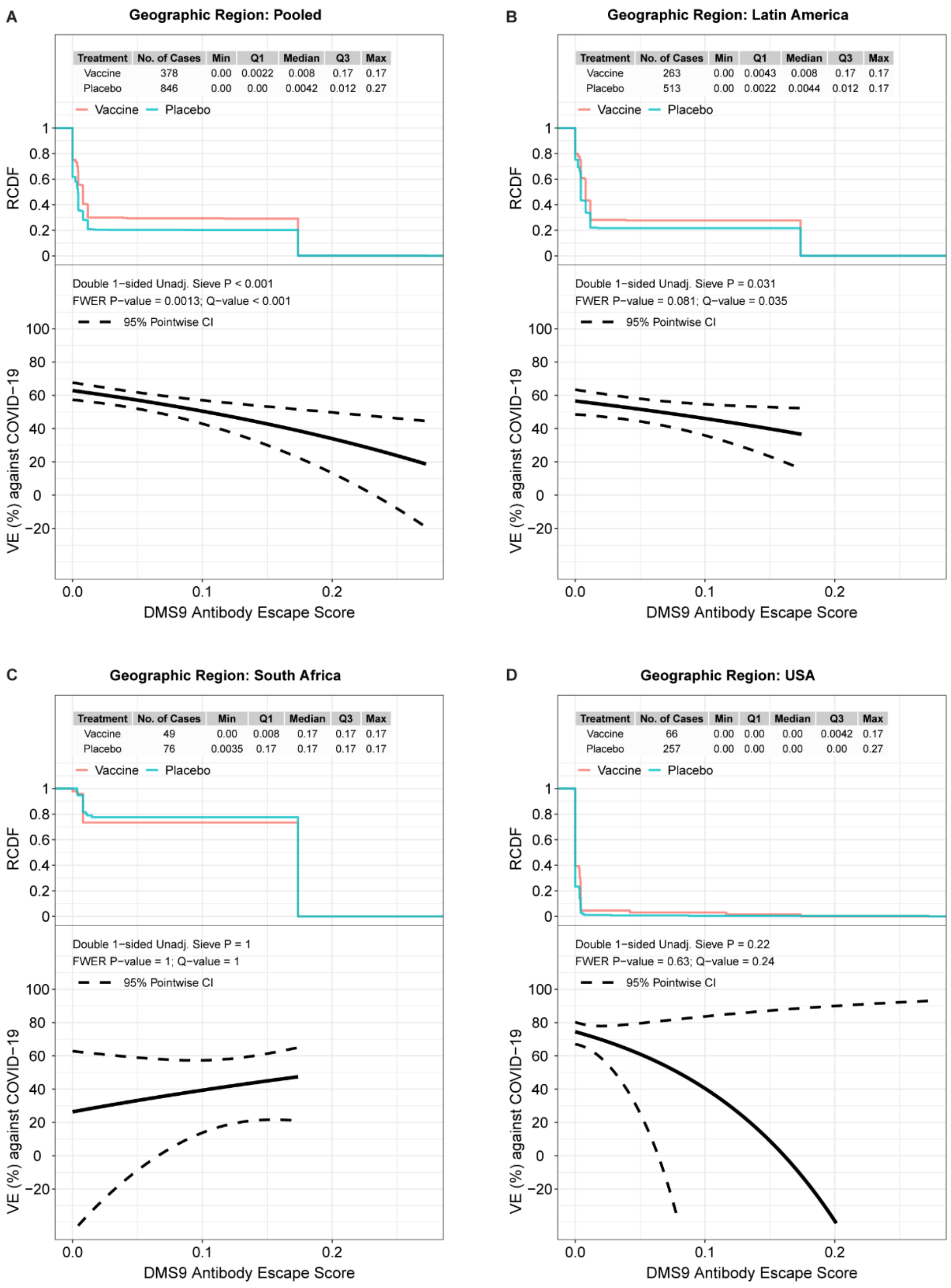

Supplementary Fig. 33
Hazard-based vaccine efficacy against the COVID-19 primary endpoint by the DMS9 antibody escape score of the disease-causing SARS-CoV-2 isolate. A) Geographic regions pooled, B) Latin America, C) South Africa, D) US. Dashed lines are 95% pointwise confidence intervals. The "Double 1-sided unadjusted sieve p-value" doubles the p-value from a one-sided Wald test of the null hypothesis of constant VE vs. the alternative hypothesis of a decreasing VE with an increasing value of the feature on the x-axis (Juraska and Gilbert, [53] Section 5). The plot at the top of each panel shows the reverse cumulative distribution function (RCDF) of the DMS9 antibody-binding escape score across SARS-CoV-2 isolates by treatment arm: Vaccine, pink; Placebo, turquoise.

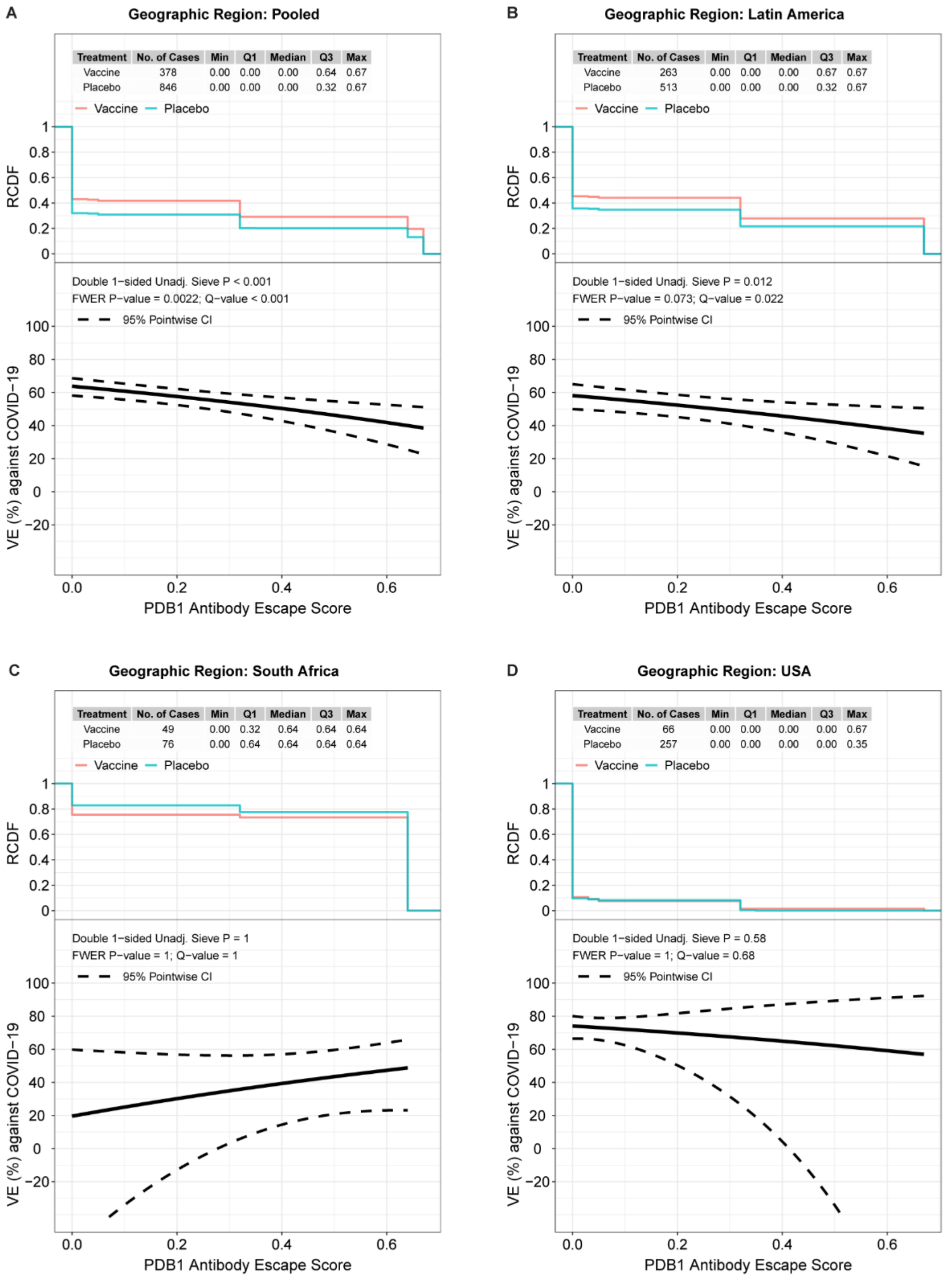

Supplementary Fig. 34

Hazard-based vaccine efficacy against the COVID-19 primary endpoint by the PDB1 antibody escape score of the disease-causing SARS-CoV-2 isolate. A) Geographic regions pooled, B) Latin America, C) South Africa, D) US. Dashed lines are 95% pointwise confidence intervals. The "Double 1-sided unadjusted sieve p-value" doubles the p-value from a one-sided Wald test of the null hypothesis of constant VE vs. the alternative hypothesis of a decreasing VE with an increasing value of the feature on the x-axis (Juraska and Gilbert, [53] Section 5). The plot at the top of each panel shows the reverse cumulative distribution function (RCDF) of the PDB1 antibody-binding escape score across SARS-CoV-2 isolates by treatment arm: Vaccine, pink; Placebo, turquoise.

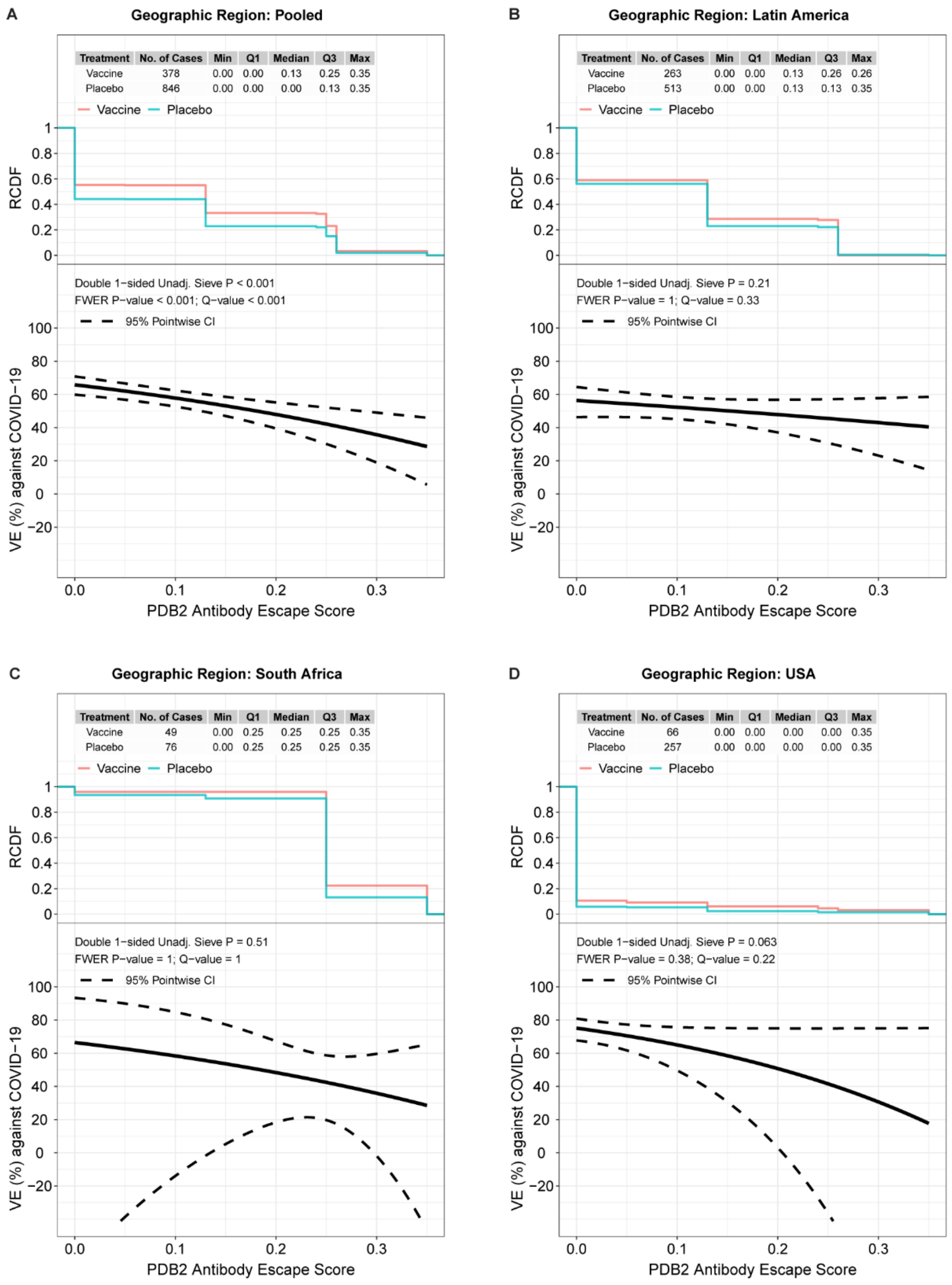

Supplementary Fig. 35

Hazard-based vaccine efficacy against the COVID-19 primary endpoint by the PDB2 antibody escape score of the disease-causing SARS-CoV-2 isolate. A) Geographic regions pooled, B) Latin America, C) South Africa, D) US. Dashed lines are 95% pointwise confidence intervals. The "Double 1-sided unadjusted sieve p-value" doubles the p-value from a one-sided Wald test of the null hypothesis of constant VE vs. the alternative hypothesis of a decreasing VE with an increasing value of the feature on the x-axis (Juraska and Gilbert, [53] Section 5). The plot at the top of each panel shows the reverse cumulative distribution function (RCDF) of the PDB2 antibody-binding escape score across SARS-CoV-2 isolates by treatment arm: Vaccine, pink; Placebo, turquoise.

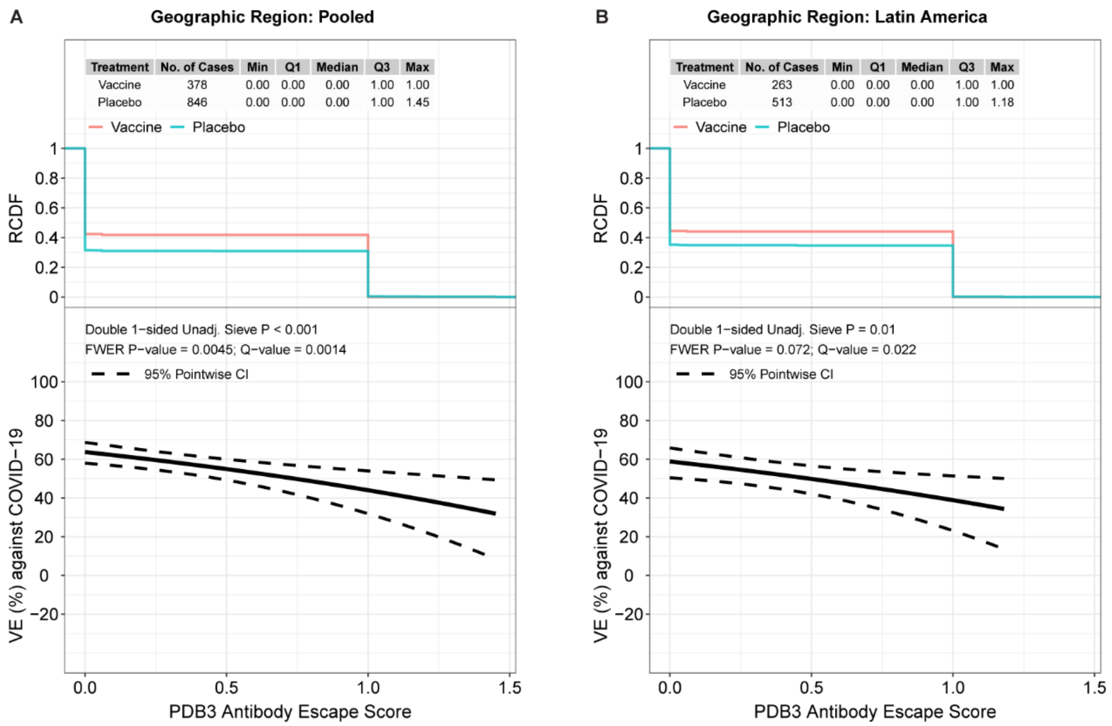

Supplementary Fig. 36
Hazard-based vaccine efficacy against the COVID-19 primary endpoint by the PDB3 antibody escape score of the disease-causing SARS-CoV-2 isolate. A) Geographic regions pooled, B) Latin America. Dashed lines are 95% pointwise confidence intervals. The "Double 1-sided unadjusted sieve p-value" doubles the p-value from a one-sided Wald test of the null hypothesis of constant VE vs. the alternative hypothesis of a decreasing VE with an increasing value of the feature on the x-axis (Juraska and Gilbert, [53] Section 5). The plot at the top of each panel shows the reverse cumulative distribution function (RCDF) of the PDB3 antibody-binding escape score across SARS-CoV-2 isolates by treatment arm: Vaccine, pink; Placebo, turquoise.

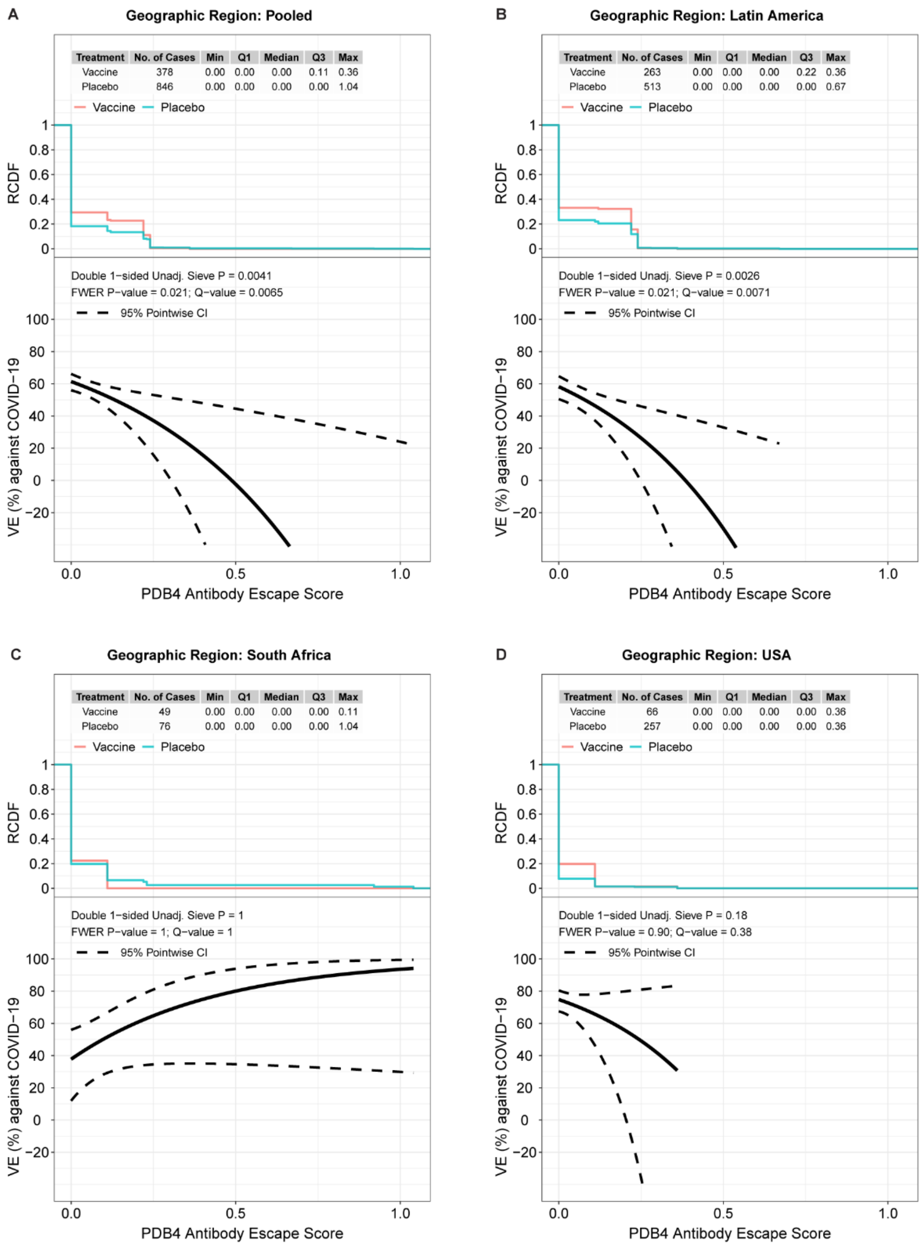

Supplementary Fig. 37

Hazard-based vaccine efficacy against the COVID-19 primary endpoint by the PDB4 antibody escape score of the disease-causing SARS-CoV-2 isolate. A) Geographic regions pooled, B) Latin America, C) South Africa, D) US. Dashed lines are 95% pointwise confidence intervals. The "Double 1-sided unadjusted sieve p-value" doubles the p-value from a one-sided Wald test of the null hypothesis of constant VE vs. the alternative hypothesis of a decreasing VE with an increasing value of the feature on the x-axis (Juraska and Gilbert, [53] Section 5). The plot at the top of each panel shows the reverse cumulative distribution function (RCDF) of the PDB4 antibody-binding escape score across SARS-CoV-2 isolates by treatment arm: Vaccine, pink; Placebo, turquoise.

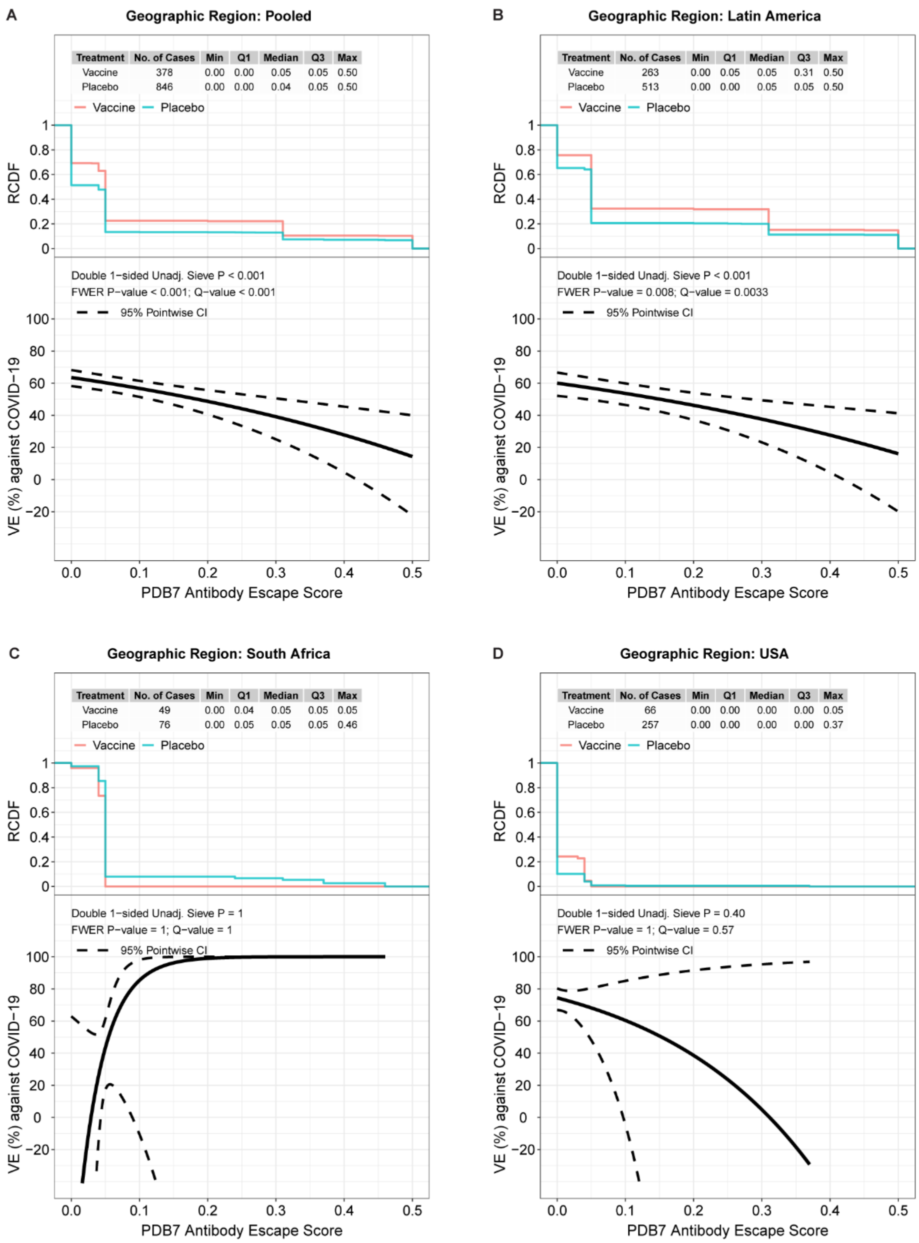

Supplementary Fig. 38
Hazard-based vaccine efficacy against the COVID-19 primary endpoint by the PDB7 antibody escape score of the disease-causing SARS-CoV-2 isolate. A) Geographic regions pooled, B) Latin America, C) South Africa, D) US. Dashed lines are 95% pointwise confidence intervals. The “Double 1-sided unadjusted sieve p-value” doubles the p-value from a one-sided Wald test of the null hypothesis of constant VE vs. the alternative hypothesis of a decreasing VE with an increasing value of the feature on the x-axis (Juraska and Gilbert, [53] Section 5). The plot at the top of each panel shows the reverse cumulative distribution function (RCDF) of the PDB7 antibody-binding escape score across SARS-CoV-2 isolates by treatment arm: Vaccine, pink; Placebo, turquoise.

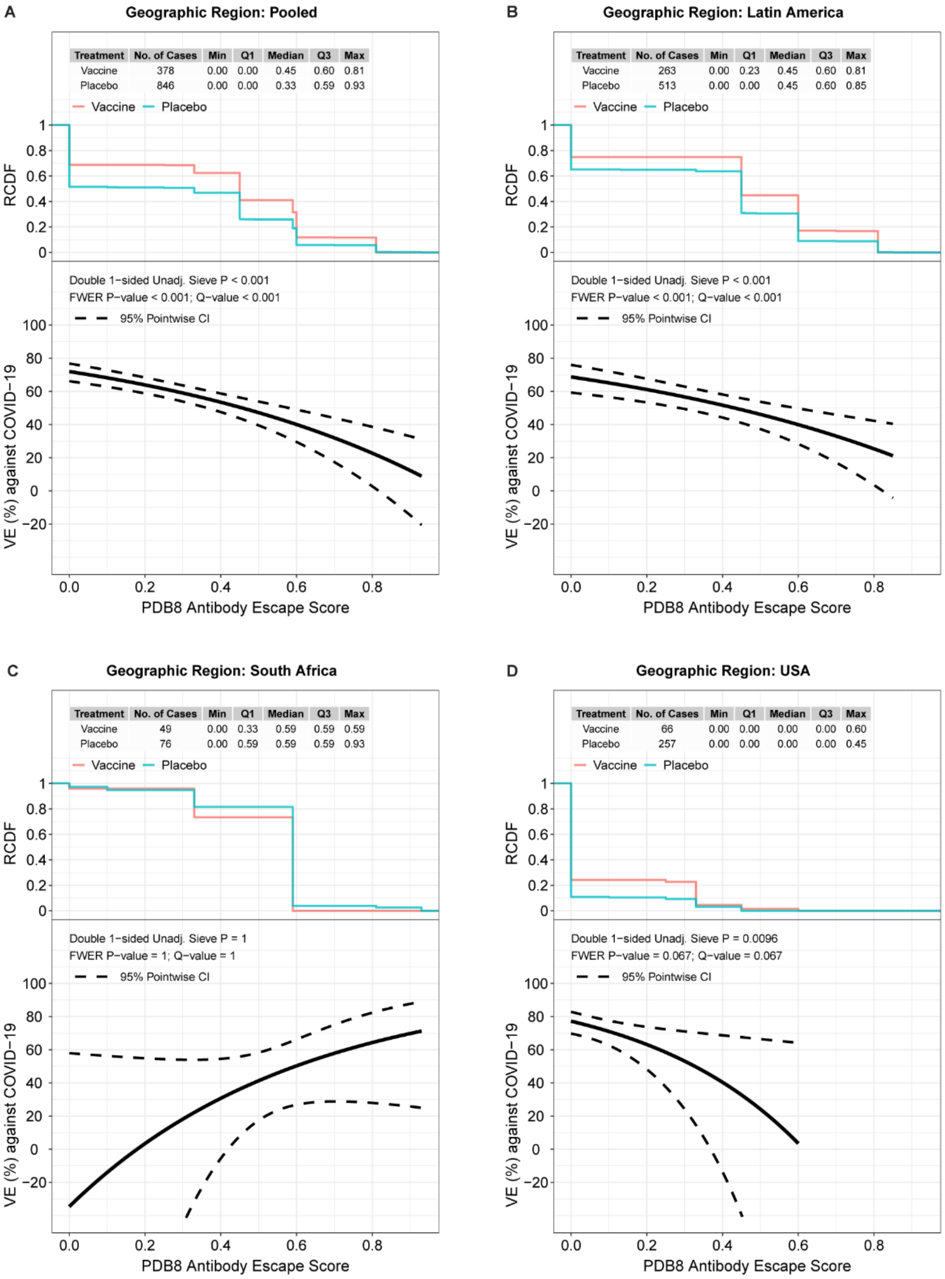

Supplementary Fig. 39

Hazard-based vaccine efficacy against the COVID-19 primary endpoint by the PDB8 antibody escape score of the disease-causing SARS-CoV-2 isolate. A) Geographic regions pooled, B) Latin America, C) South Africa, D) US. Dashed lines are 95% pointwise confidence intervals. The “Double 1-sided unadjusted sieve p-value” doubles the p-value from a one-sided Wald test of the null hypothesis of constant VE vs. the alternative hypothesis of a decreasing VE with an increasing value of the feature on the x-axis (Juraska and Gilbert, [53] Section 5). The plot at the top of each panel shows the reverse cumulative distribution function (RCDF) of the PDB8 antibody-binding escape score across SARS-CoV-2 isolates by treatment arm: Vaccine, pink; Placebo, turquoise.

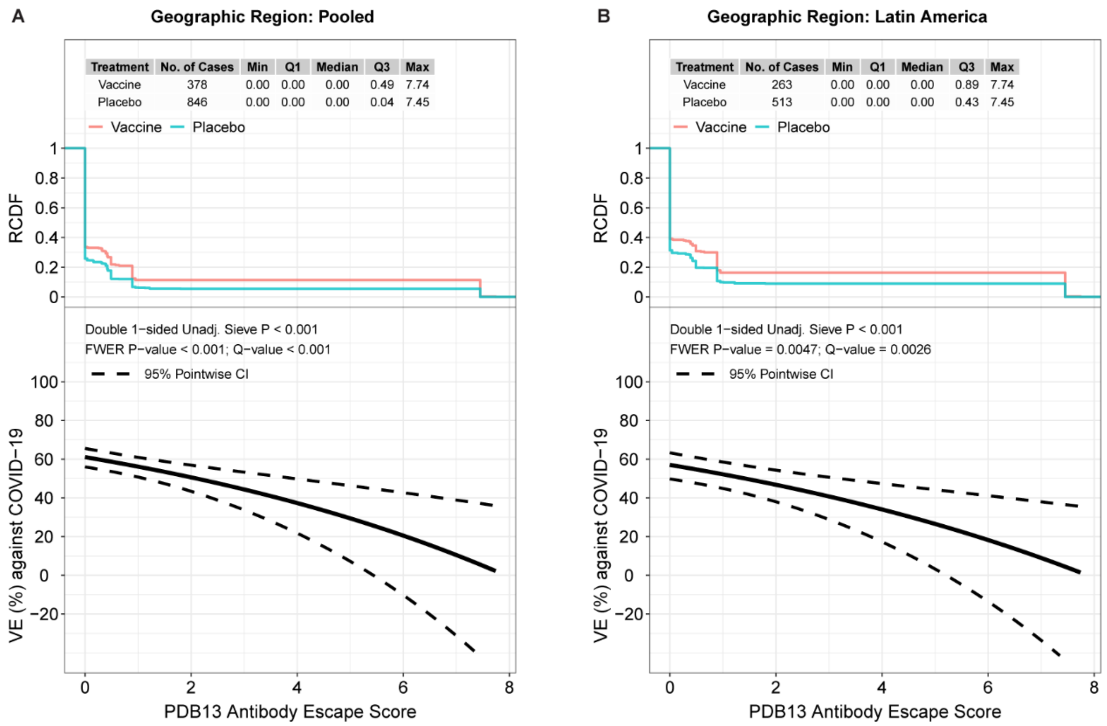

Supplementary Fig. 40

Hazard-based vaccine efficacy against the COVID-19 primary endpoint by the PDB13 antibody escape score of the disease-causing SARS-CoV-2 isolate. A) Geographic regions pooled, B) Latin America. Dashed lines are 95% pointwise confidence intervals. The "Double 1-sided unadjusted sieve p-value" doubles the p-value from a one-sided Wald test of the null hypothesis of constant VE vs. the alternative hypothesis of a decreasing VE with an increasing value of the feature on the x-axis (Juraska and Gilbert, [53] Section 5). The plot at the top of each panel shows the reverse cumulative distribution function (RCDF) of the PDB13 antibody-binding escape score across SARS-CoV-2 isolates by treatment arm: Vaccine, pink; Placebo, turquoise.

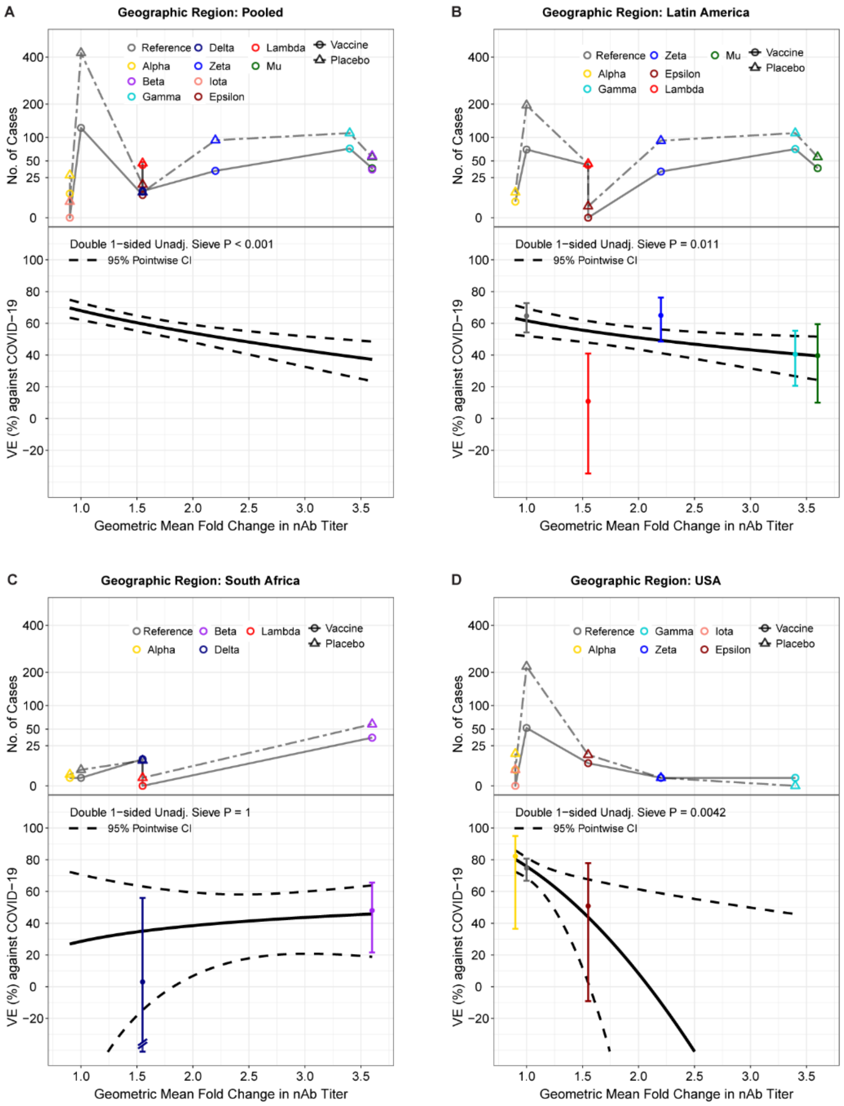

Supplementary Fig. 41

For (A) the geographic regions pooled, (B) the Latin America cohort, (C) the South Africa cohort, and (D) the United States cohort, hazard-based vaccine efficacy against the primary COVID-19 endpoint by geometric fold change in neutralizing antibody (nAb) titer against the disease-causing SARS-CoV-2 lineage vs. against the D614G Reference strain. The top plot in each panel shows the numbers of endpoints by treatment arm and color-coded by lineage. The bottom plot in each panel shows the estimated vaccine efficacy by geometric fold change in nAb titer against the disease-causing SARS-CoV-2 lineage vs. against the D614G Reference strain. The dotted lines are 95% confidence intervals. The dots are overall vaccine efficacy point estimates against the given lineage, with the vertical bars showing the 95% confidence intervals. The “Double 1-sided unadjusted sieve p-value” doubles the p-value from a one-sided Wald test of the null hypothesis of constant VE vs. the alternative hypothesis of a decreasing VE with an increasing value of the feature on the x-axis (Juraska and Gilbert,[53] Section 5).

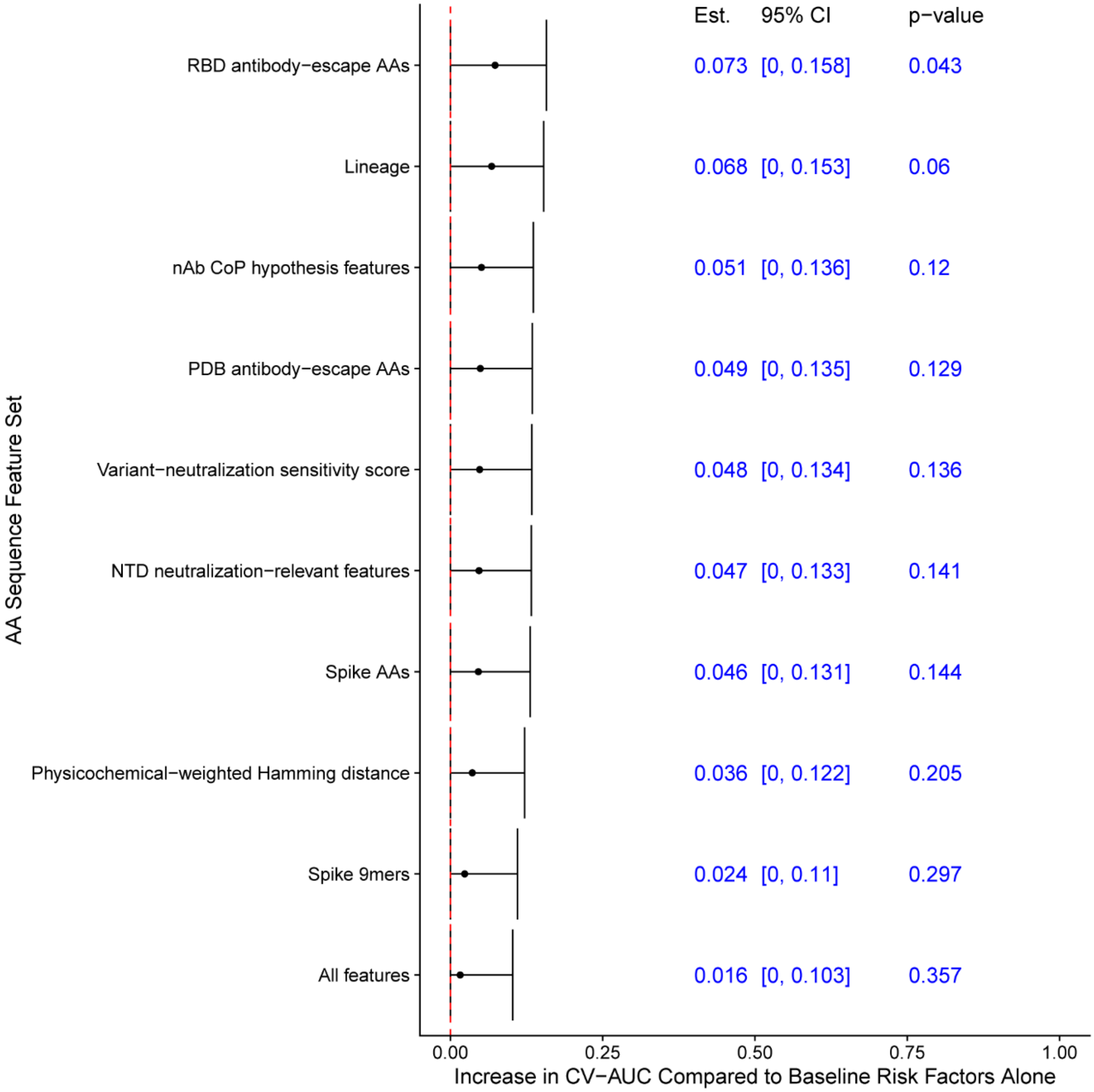

Supplementary Fig. 42
For the Latin America cohort among COVID-19 primary endpoints, variable importance measure (VIM) (defined as the increase in cross-validated area under the receiver operating characteristic curve [CV-AUC] from including a set of variables compared to using baseline risk factors alone), 95% confidence intervals, and p-values for the null hypothesis of zero variable importance for predicting assignment to vaccine vs placebo using each variable set. All analyses include the baseline risk factors.

Supplementary Table 19. Estimation and inference in the per-protocol baseline seronegative cohort about vaccine efficacy against the severe–critical COVID-19 endpoint caused by specific SARS-CoV-2 lineages.

Geographic Region	WHO-labeled Variant	No. of Cases (V vs. P) (Incidence per 100 PYRs)	VE (%) (95% CI)	p-value[1]	Two-sided Differential VE Unadjusted p-value[2]
Latin America					0.50
	Reference Lineage	8 (0.3) vs. 41 (1.4)	82.7 (64.0, 91.7)	<0.001	
	Gamma	9 (0.3) vs. 24 (0.8)	64.3 (25.9, 82.8)	0.0057	
	Zeta	1 (0) vs. 11 (0.4)	94.5 (-27.3, 99.8)	0.07	
	Lambda	3 (0.1) vs. 8 (0.3)	61.7 (-31.4, 88.9)	0.13	
	Mu	4 (0.1) vs. 19 (0.7)	84.0 (41.6, 95.6)	0.0056	
South Africa					-
	Beta	7 (0.6) vs. 25 (2)	75.8 (44.9, 89.4)	<0.001	
US					-
	Reference	4 (0.2) vs. 25 (1)	82.4 (54.0, 93.3)	<0.001	

1. This column tests the null hypothesis H_{Aj0} vs. H_{Aj2} using the test statistic U_{2j} (pp 17-18 of Heng et al.[52]).
2. This column tests the null hypothesis H_{B0} vs. H_{B2} using the test statistic T_2 (pp 18-19 of Heng et al.[52]).

Supplementary Table 20. Differential VE estimates against the severe-critical COVID-19 endpoint across pairs of lineages or across a lineage (X) vs. all other lineages combined (Not X) in the per-protocol baseline seronegative cohort in Latin America.

Comparison	Differential VE[1] (95% CI)	Two-sided Differential VE P-value[2]	FWER P-value	Q-value
Reference vs. Not Reference	1.42 (0.57, 3.51)	0.45	1	0.67
Gamma vs. Not Gamma	0.48 (0.19, 1.24)	0.13	1	0.63
Lambda vs. Not Lambda	0.53 (0.14, 1.99)	0.35	1	0.63
Mu vs. Not Mu	1.44 (0.35, 5.92)	0.61	1	0.71
Zeta vs. Not Zeta	4.26 (0.17, 105.12)	0.38	1	0.63
Reference vs. Gamma	2.06 (0.73, 5.83)	0.17	1	0.63
Reference vs. Lambda	2.21 (0.52, 9.33)	0.28	1	0.63
Reference vs. Mu	0.92 (0.21, 4.08)	0.91	1	0.93
Reference vs. Zeta	0.32 (0.01, 8.67)	0.50	1	0.67
Gamma vs. Lambda	1.07 (0.25, 4.53)	0.93	1	0.93
Gamma vs. Mu	0.45 (0.09, 2.15)	0.32	1	0.63
Gamma vs. Zeta	0.15 (0.01, 3.87)	0.26	1	0.63
Lambda vs. Mu	0.42 (0.07, 2.53)	0.34	1	0.63
Lambda vs. Zeta	0.14 (0.00, 4.37)	0.27	1	0.63
Mu vs. Zeta	0.34 (0.01, 10.31)	0.54	1	0.67

[1] Differential VE (DVE) for genotype 1 vs. genotype 2, with VE(genotype 1) >= VE(genotype 2), is calculated as DVE = [1 – VE(genotype 2)]/[1- VE(genotype 1)], with interpretation that vaccine protection is DVE-fold better against genotype 1 than against genotype 2.

[2]This column tests the null hypothesis H_{B0} vs. H_{B2} using the test statistic T_2 for J=2 (pp 18-19 of Heng et al.[52]).

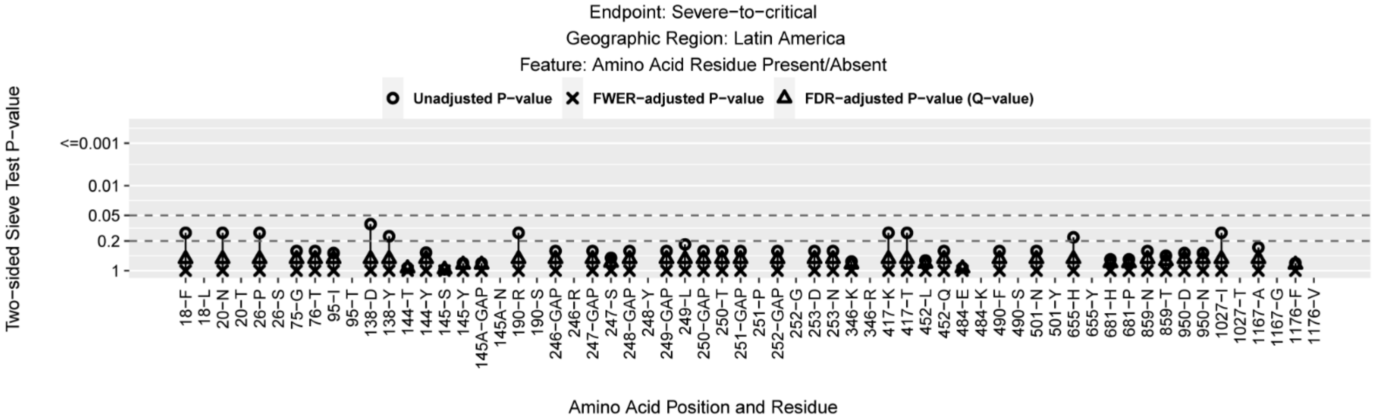

Supplementary Fig. 43
In the Latin America cohort, sieve test p-values for hazard-based vaccine efficacy against the severe–critical COVID-19 endpoint with a specific amino acid residue at screened-in positions. FDR, false discovery rate; FWER, family-wise error rate.

Supplementary Table 21. For deep mutational scanning (DMS) escape score and VE against the severe–critical COVID-19 endpoint, p-values from the Juraska and Gilbert[54] one sided weighted Wald test of H_{10} : $V E(v) = 0$ for all v and from double one-sided Wald test of H_{20} : $V E(v) = V E$ for all v against H_{21} : $V E(v)$ decreases with v. Family-wise error rate (FWER) adjusted p-values are Holm-Bonferroni and FDR-adjusted p-values (q-values) are Benjamini-Hochberg.

Geographic Region	Feature Name	Spike Region	Antibody Set	Test of H_{10} p-value	p-value	Test of H_{20} FWER p-value	q-value
Pooled	DMS	RBD	All	<0.001	0.59	1.00	0.76
	DMS1	RBD	Cluster 1	<0.001	0.41	1.00	0.64
	DMS2	RBD	Cluster 2	<0.001	0.38	1.00	0.64
	DMS3	RBD	Cluster 3	<0.001	0.97	1.00	0.97
	DMS5	RBD	Cluster 5	<0.001	0.90	1.00	0.97
	DMS6	RBD	Cluster 6	<0.001	0.17	1.00	0.64
	DMS7	RBD	Cluster 7	<0.001	0.30	1.00	0.64
	DMS8	RBD	Cluster 8[¶§]	<0.001	0.018	0.16	0.16
	DMS9	RBD	Cluster 9	<0.001	0.42	1.00	0.64
Latin America	DMS	RBD	All	<0.001	0.27	1.00	0.48
	DMS1	RBD	Cluster 1	<0.001	0.11	0.89	0.33
	DMS2	RBD	Cluster 2	<0.001	0.099	0.89	0.33
	DMS3	RBD	Cluster 3	<0.001	0.56	1.00	0.64
	DMS5	RBD	Cluster 5	<0.001	0.50	1.00	0.64
	DMS6	RBD	Cluster 6	<0.001	0.84	1.00	0.84
	DMS7	RBD	Cluster 7	<0.001	0.15	0.89	0.33
	DMS8	RBD	Cluster 8	<0.001	0.44	1.00	0.64
	DMS9	RBD	Cluster 9	<0.001	0.10	0.89	0.33
US	DMS	RBD	All	<0.001	0.71	1.00	1.00
	DMS1	RBD	Cluster 1	<0.001	0.55	1.00	1.00
	DMS2	RBD	Cluster 2[¶§]	<0.001	0.021	0.17	0.084
	DMS3	RBD	Cluster 3	<0.001	1.00	1.00	1.00
	DMS5	RBD	Cluster 5	<0.001	1.00	1.00	1.00
	DMS6	RBD	Cluster 6[¶§]	<0.001	0.021	0.17	0.084
	DMS7	RBD	Cluster 7	<0.001	0.60	1.00	1.00
	DMS9	RBD	Cluster 9	<0.001	1.00	1.00	1.00

¶ Unadjusted p-value for differential VE is ≤ 0.05.

† FWER-adjusted p-value for differential VE is ≤ 0.05.

§ FDR-adjusted p-value (q-value) for differential VE is ≤ 0.2 and unadjusted p-value for differential VE is ≤ 0.05.

Supplementary Table 22. For PDB antibody escape score and VE against the severe–critical COVID-19 endpoint, p-values from the Juraska and Gilbert[54] one sided weighted Wald test of $H_{10} : VE(v) = 0$ for all v and from double one-sided Wald test of $H_{20} : VE(v) = VE$ for all v against $H_{21} : VE(v)$ decreases with v. Family-wise error rate (FWER) adjusted p-values are Holm-Bonferroni and FDR-adjusted p-values (q-values) are Benjamini-Hochberg.

Geographic Region	Feature Name	Spike Region	PDB Antibody Escape Score	Test of H_{10} p-value	Test of H_{20} p-value	Test of H_{20} FWER p-value	Test of H_{20} q-value
Pooled	PDB1	RBD	Cluster 1	<0.001	0.38	1.00	0.96
	PDB2	RBD	Cluster 2	<0.001	0.29	1.00	0.96
	PDB4	RBD	Cluster 4	<0.001	0.32	1.00	0.96
	PDB5	RBD	Cluster 5	<0.001	1.00	1.00	1.00
	PDB6	RBD	Cluster 6	<0.001	1.00	1.00	1.00
	PDB7	RBD	Cluster 7	<0.001	1.00	1.00	1.00
	PDB8	RBD	Cluster 8	<0.001	0.24	1.00	0.96
	PDB9	RBD	Cluster 9	<0.001	1.00	1.00	1.00
	PDB13	NTD	Cluster 13	<0.001	0.61	1.00	1.00
	PDB14	NTD	Cluster 14	<0.001	0.82	1.00	1.00
Latin America	PDB1	RBD	Cluster 1	<0.001	0.13	1.00	0.94
	PDB2	RBD	Cluster 2	<0.001	0.31	1.00	0.94
	PDB4	RBD	Cluster 4	<0.001	0.71	1.00	1.00
	PDB5	RBD	Cluster 5	<0.001	1.00	1.00	1.00
	PDB6	RBD	Cluster 6	<0.001	1.00	1.00	1.00
	PDB7	RBD	Cluster 7	<0.001	0.98	1.00	1.00
	PDB8	RBD	Cluster 8	<0.001	0.24	1.00	0.94
	PDB13	NTD	Cluster 13	<0.001	0.62	1.00	1.00
	PDB14	NTD	Cluster 14	<0.001	1.00	1.00	1.00
US	PDB13	NTD	Cluster 13¶§†	<0.001	0.016	0.016	0.016

¶ Unadjusted p-value for differential VE is ≤ 0.05.

† FWER-adjusted p-value for differential VE is ≤ 0.05.

§ FDR-adjusted p-value (q-value) for differential VE is ≤ 0.2 and unadjusted p-value for differential VE is ≤ 0.05.

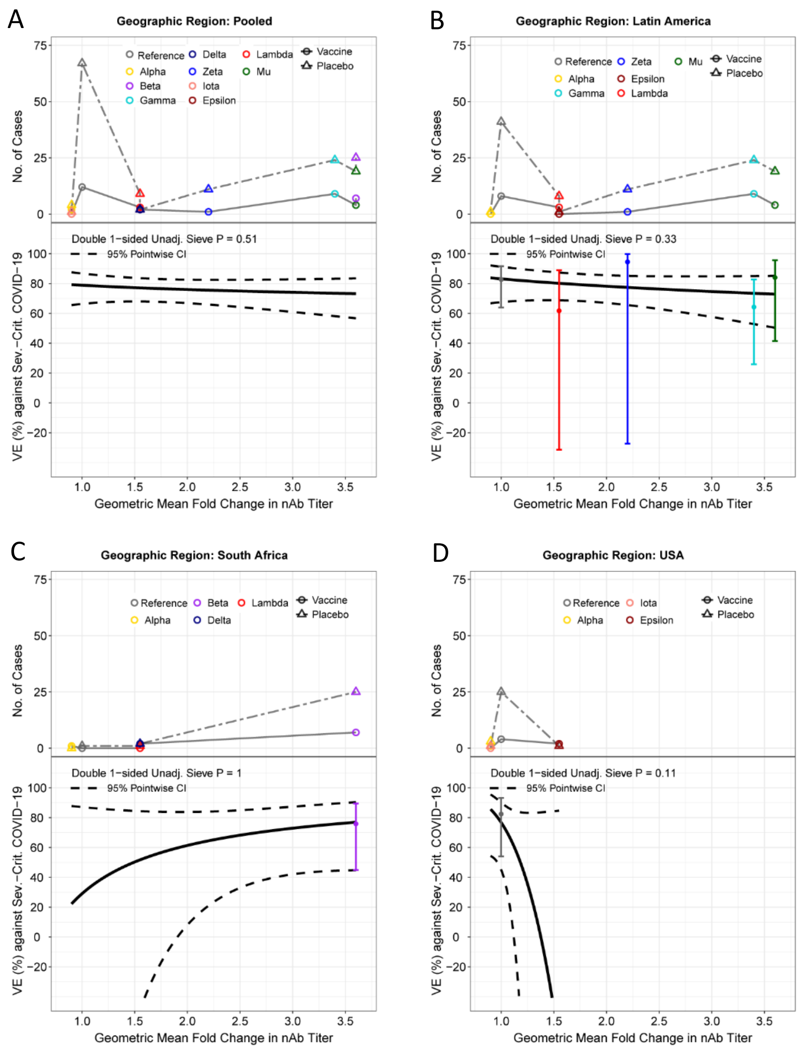

Supplementary Fig. 44

For (A) the geographic regions pooled, (B) the Latin America cohort, (C) the South Africa cohort, and (D) the United States cohort, hazard-based vaccine efficacy (VE) against the severe–critical COVID-19 endpoint by geometric fold change in neutralizing antibody (nAb) titer against the disease-causing SARS-CoV-2 lineage vs. against the D614G Reference strain. The top plot in each panel shows the distributions of geometric fold change in nAb titer by treatment arm, color-coded by lineage. The bottom plot in each panel shows the estimated vaccine efficacy by geometric fold change in nAb titer against the disease-causing SARS-CoV-2 lineage vs. against the D614G Reference strain. The dotted lines are 95% confidence intervals. The dots are overall vaccine efficacy estimates for the given lineage, with the vertical bars showing the 95% confidence intervals. The “Double 1-sided unadjusted sieve p-value” doubles the p-value from a one-sided Wald test of the null hypothesis of constant VE vs. the alternative hypothesis of a decreasing VE with an increasing value of the feature on the x-axis (Juraska and Gilbert,[53] Section 5).

Supplementary Table 23. For physicochemical-weighted Hamming distance to the vaccine insert, and for VE against the severe–critical COVID-19 endpoint, p-values from the Juraska and Gilbert[54] one sided weighted Wald test of $H_{10} : VE(v) = 0$ for all v and from double one-sided Wald test of $H_{20} : VE(v) = VE$ for all v against $H_{21} : VE(v)$ decreases with v. Family-wise error rate (FWER) adjusted p-values are Holm-Bonferroni and FDR-adjusted p-values (q-values) are Benjamini-Hochberg.

			Test of H_{20}		
Geographic Region	Feature Name	Test of H_{10} p-value	p-value	FWER p-value	q-value
Pooled	Spike¶§	<0.001	0.022	0.11	0.042
	RBD	<0.001	0.14	0.24	0.14
	NTD¶§	<0.001	0.023	0.11	0.042
	S1¶§	<0.001	0.025	0.11	0.042
	S2	<0.001	0.12	0.24	0.14
Latin America	Spike	0.0012	0.12	0.49	0.20
	RBD	<0.001	0.26	0.52	0.33
	NTD	<0.001	0.099	0.49	0.20
	S1	<0.001	0.11	0.49	0.20
	S2	<0.001	0.42	0.52	0.42
South Africa	Spike	0.0011	0.27	1.00	0.70
	NTD	0.0039	0.75	1.00	0.75
	S1	0.0029	0.51	1.00	0.70
	S2	<0.001	0.53	1.00	0.70
US	Spike¶§	<0.001	0.035	0.071	0.047
	RBD¶§	<0.001	0.048	0.071	0.048
	NTD¶§ †	<0.001	0.0035	0.014	0.014
	S1¶§	<0.001	0.021	0.063	0.042

¶ Unadjusted p-value for differential VE is ≤ 0.05.
† FWER-adjusted p-value for differential VE is ≤ 0.05.
§ FDR-adjusted p-value (q-value) for differential VE is ≤ 0.2 and unadjusted p-value for differential VE is ≤ 0.05.

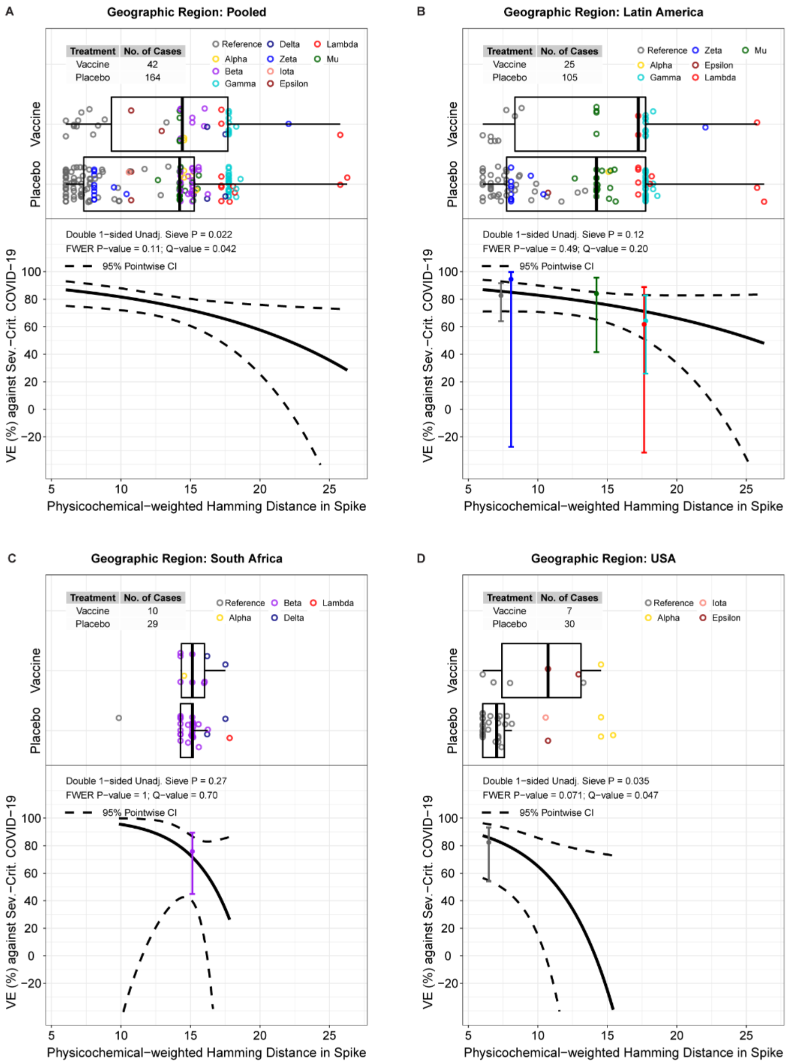

Supplementary Fig. 45

For (A) the geographic regions pooled, (B) the Latin America cohort, (C) the South Africa cohort, and (D) the United States cohort, hazard-based vaccine efficacy (VE) against the severe–critical COVID-19 endpoint by physicochemical-weighted (PCW) Hamming distance in Spike from the disease-causing SARS-CoV-2 isolate to that of the vaccine-insert sequence. The top plot in each panel shows the distributions of PCW-Hamming distance in Spike by treatment arm, color-coded by lineage. The left and right edges of the box plots represent the 25th and 75th percentiles of PCW-Hamming distance in Spike and the vertical middle line represents the 50th percentile. The horizontal bars extend from the 25th (or 75th) percentile of PCW-Hamming distance to the minimum (or maximum) PCW-Hamming distance within the 25th (or 75th) percentile of Hamming distance minus (or plus) 1.5 times the interquartile range. The bottom plot in each panel shows the estimated vaccine efficacy by PCW-Hamming distance in Spike from the disease-causing SARS-CoV-2 isolate to that of the vaccine-insert sequence. The dashed lines are pointwise 95% confidence intervals. The dots are overall vaccine efficacy estimates for the given lineage, with the vertical bars showing the 95% confidence intervals. In each panel, the “Double 1-sided unadjusted sieve p-value” doubles the p-value from a one-sided Wald test of the null hypothesis of constant VE vs. the alternative hypothesis of a decreasing VE with an increasing value of the feature on the x-axis (Juraska and Gilbert,[53] Section 5).

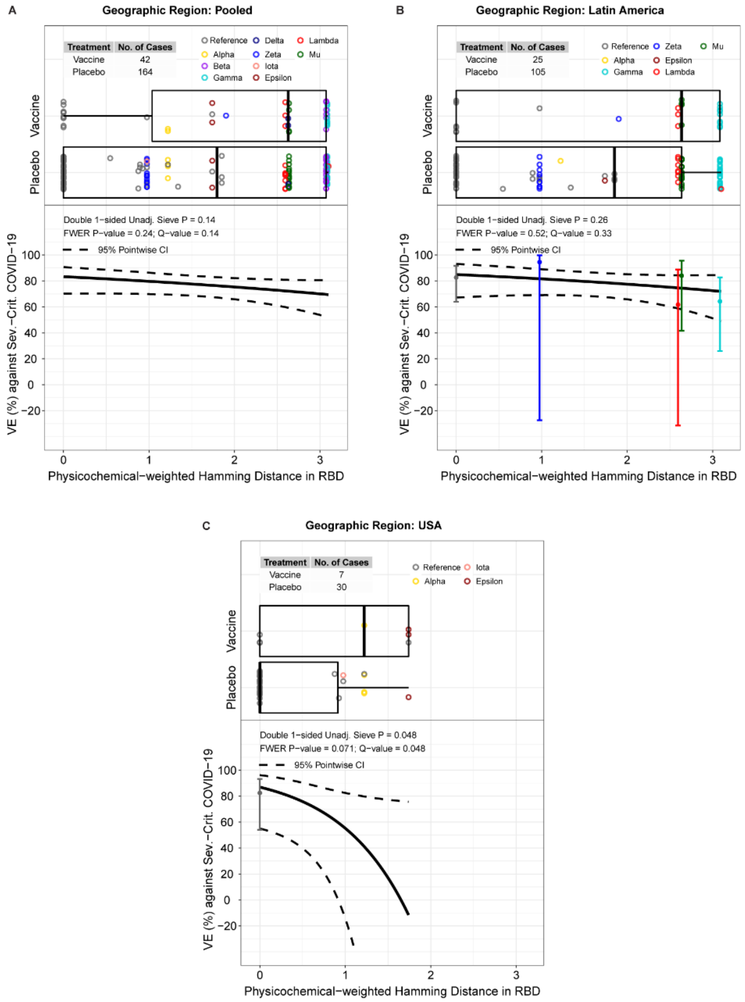

Supplementary Fig. 46

For (A) the geographic regions pooled, (B) the Latin America cohort, and (C) the United States cohort (note that the sequence feature for South Africa was not screened in), hazard-based vaccine efficacy (VE) against the severe–critical COVID-19 endpoint by physicochemical-weighted (PCW) Hamming distance from the disease-causing SARS-CoV-2 isolate RBD to that of the vaccine strain RBD. The top plot in each panel shows the distributions of PCW-Hamming distance in RBD by treatment arm, color-coded by lineage. The left and right edges of the box plots represent the 25th and 75th percentiles of PCW-Hamming distance in RBD and the vertical middle line represents the 50th percentile. The horizontal bars extend from the 25th (or 75th) percentile of PCW-Hamming distance to the minimum (or maximum) PCW-Hamming distance within the 25th (or 75th) percentile of Hamming distance minus (or plus) 1.5 times the interquartile range. The bottom plot in each panel shows the estimated vaccine efficacy by PCW-Hamming distance from the disease-causing SARS-CoV-2 isolate RBD to that of the vaccine strain RBD. The dashed lines are pointwise 95% confidence intervals. The dots are overall vaccine efficacy estimates for the given lineage, with the vertical bars showing the 95% confidence intervals. In each panel, the "Double 1-sided unadjusted sieve p-value" doubles the p-value from a one-sided Wald test of the null hypothesis of constant VE vs. the alternative hypothesis of a decreasing VE with an increasing value of the feature on the x-axis (Juraska and Gilbert,[53] Section 5).

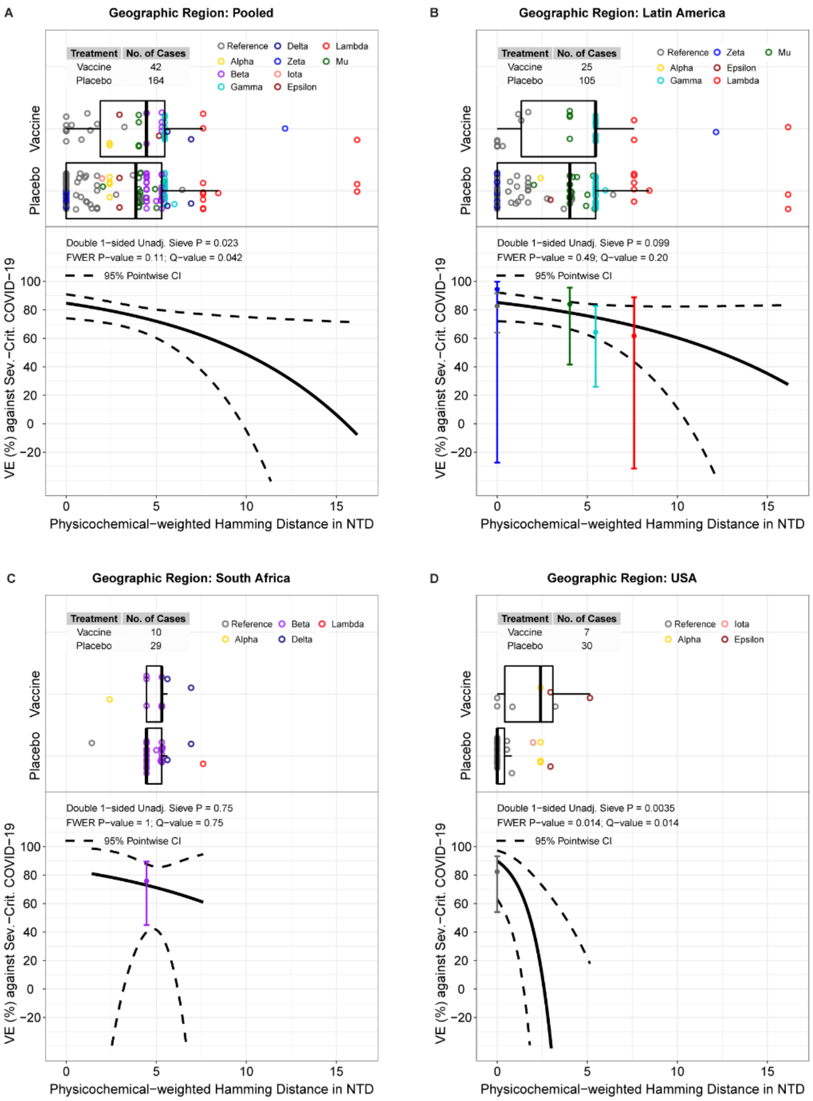

Supplementary Fig. 47

For (A) the geographic regions pooled, (B) the Latin America cohort, (C) the South Africa cohort, and (D) the United States cohort, hazard-based vaccine efficacy (VE) against the severe–critical COVID-19 endpoint by physicochemical-weighted (PCW) Hamming distance from the disease-causing SARS-CoV-2 isolate NTD to that of the vaccine strain NTD. The top plot in each panel shows the distributions of PCW-Hamming distance in NTD by treatment arm, color-coded by lineage. The left and right edges of the box plots represent the 25th and 75th percentiles of PCW-Hamming distance in NTD and the vertical middle line represents the 50th percentile. The horizontal bars extend from the 25th (or 75th) percentile of PCW-Hamming distance to the minimum (or maximum) PCW-Hamming distance within the 25th (or 75th) percentile of Hamming distance minus (or plus) 1.5 times the interquartile range. The bottom plot in each panel shows the estimated vaccine efficacy by PCW-Hamming distance from the disease-causing SARS-CoV-2 isolate NTD to that of the vaccine strain NTD. The dashed lines are pointwise 95% confidence intervals. The dots are overall vaccine efficacy estimates for the given lineage, with the vertical bars showing the 95% confidence intervals. In each panel, the "Double 1-sided unadjusted sieve p-value" doubles the p-value from a one-sided Wald test of the null hypothesis of constant VE vs. the alternative hypothesis of a decreasing VE with an increasing value of the feature on the x-axis (Juraska and Gilbert,[53] Section 5).

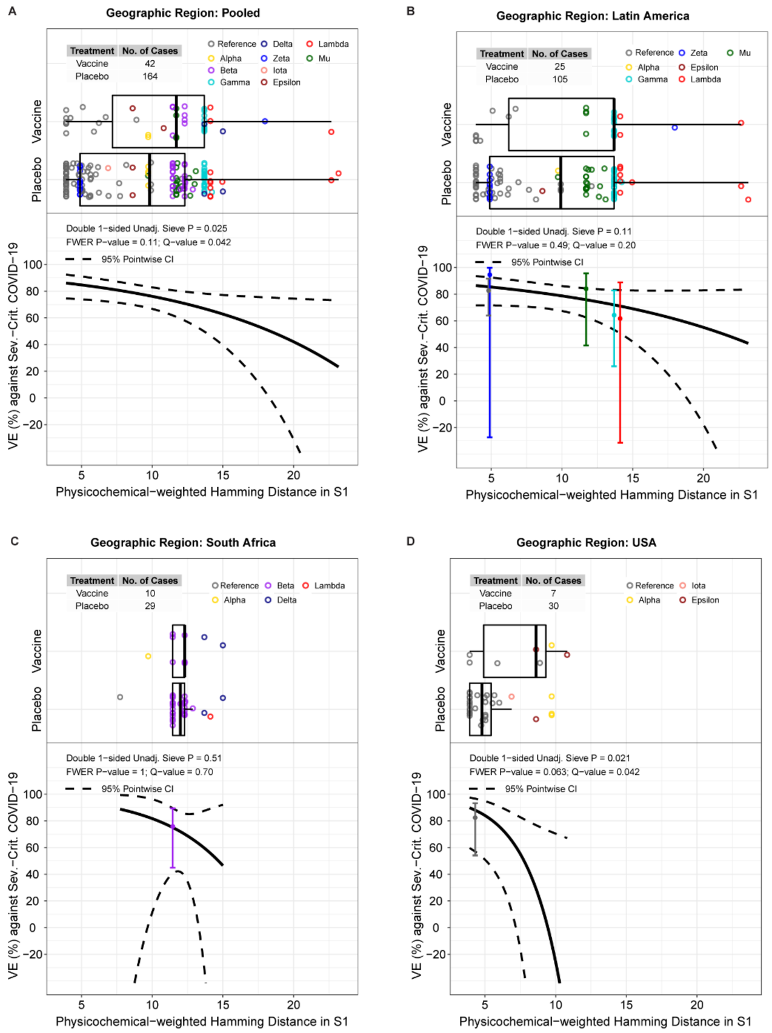

Supplementary Fig. 48

For (A) the geographic regions pooled, (B) the Latin America cohort, (C) the South Africa cohort, and (D) the United States cohort, hazard-based vaccine efficacy (VE) against the severe–critical COVID-19 endpoint by physicochemical-weighted (PCW) Hamming distance from the disease-causing SARS-CoV-2 isolate S1 to that of the vaccine strain S1. The top plot in each panel shows the distributions of PCW-Hamming distance in S1 by treatment arm, color-coded by lineage. The left and right edges of the box plots represent the 25th and 75th percentiles of PCW-Hamming distance in S1 and the vertical middle line represents the 50th percentile. The horizontal bars extend from the 25th (or 75th) percentile of PCW-Hamming distance to the minimum (or maximum) PCW-Hamming distance within the 25th (or 75th) percentile of Hamming distance minus (or plus) 1.5 times the interquartile range. The bottom plot in each panel shows the estimated vaccine efficacy by PCW-Hamming distance from the disease-causing SARS-CoV-2 isolate S1 to that of the vaccine strain S1. The dashed lines are pointwise 95% confidence intervals. The dots are overall vaccine efficacy estimates for the given lineage, with the vertical bars showing the 95% confidence intervals. In each panel, the "Double 1-sided unadjusted sieve p-value" doubles the p-value from a one-sided Wald test of the null hypothesis of constant VE vs. the alternative hypothesis of a decreasing VE with an increasing value of the feature on the x-axis (Juraska and Gilbert,[53] Section 5).

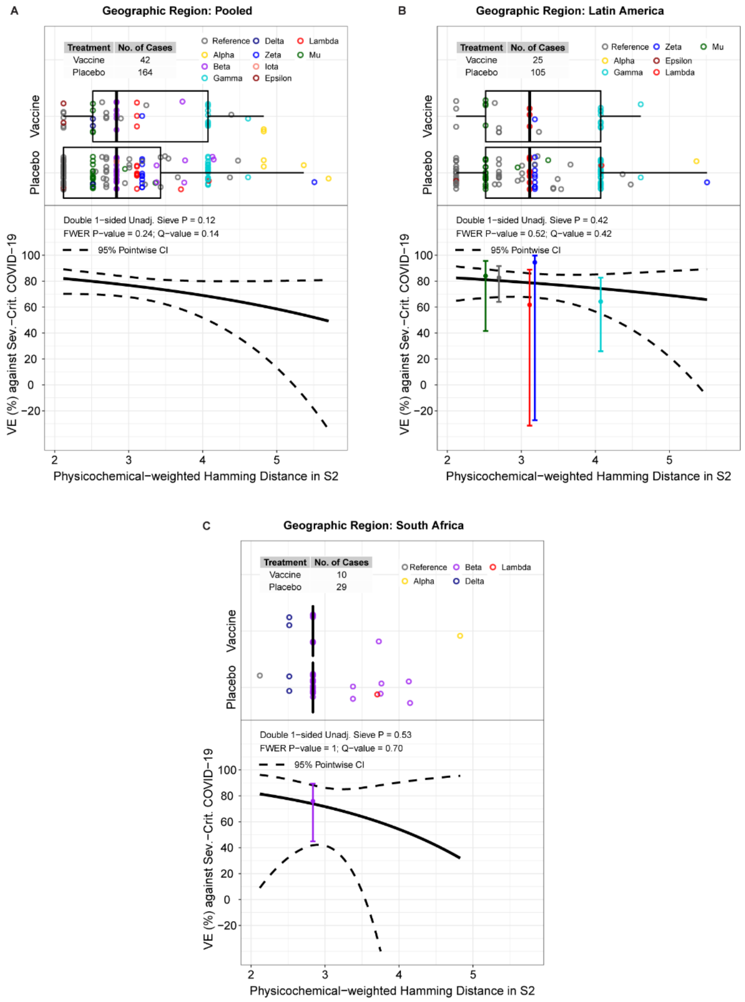

Supplementary Fig. 49

For (A) the geographic regions pooled, (B) the Latin America cohort, (C) the South Africa cohort (note that this feature for US was not screened in), hazard-based vaccine efficacy (VE) against the severe–critical COVID-19 endpoint by physicochemical-weighted (PCW) Hamming distance from the disease-causing SARS-CoV-2 isolate S2 to that of the vaccine strain S2. The top plot in each panel shows the distributions of PCW-Hamming distance in S2 by treatment arm, color-coded by lineage. The left and right edges of the box plots represent the 25th and 75th percentiles of PCW-Hamming distance in S2 and the vertical middle line represents the 50th percentile. The horizontal bars extend from the 25th (or 75th) percentile of PCW-Hamming distance to the minimum (or maximum) PCW-Hamming distance within the 25th (or 75th) percentile of Hamming distance minus (or plus) 1.5 times the interquartile range. The bottom plot in each panel shows the estimated vaccine efficacy by PCW-Hamming distance from the disease-causing SARS-CoV-2 isolate S2 to that of the vaccine strain S2. The dashed lines are pointwise 95% confidence intervals. The dots are overall vaccine efficacy estimates for the given lineage, with the vertical bars showing the 95% confidence intervals. In each panel, the “Double 1-sided unadjusted sieve p-value” doubles the p-value from a one-sided Wald test of the null hypothesis of constant VE vs. the alternative hypothesis of a decreasing VE with an increasing value of the feature on the x-axis (Juraska and Gilbert,[53] Section 5).

Supplementary Table 24. Estimation and inference in the per-protocol baseline seronegative cohort about VE against the severe–critical COVID-19 endpoint with a specific nAb-CoP hypothesis-driven screened-in NTD feature value.

		Sequence Feature Value = 1			Sequence Feature Value = 0			Two-sided Differential VE		
Geographic Region	NTD Feature	No. of Cases (V vs. P) (Incidence per 100 PYRs)	VE (%) (95% CI)	p-value[1]	No. of Cases (V vs. P) (Incidence per 100 PYRs)	VE (%) (95% CI)	p-value[1]	p-value[2]	FWER p-value	q-value
Pooled	NTD4	22 (0.3) vs. 66 (1)	70.5 (53.6, 81.2)	<0.001	20 (0.3) vs. 98 (1.5)	80.5 (69.1, 87.7)	<0.001	0.21	0.21	0.21
Latin America	NTD1	8 (0.3) vs. 33 (1.1)	79.9 (54.8, 91.1)	<0.001	17 (0.6) vs. 72 (2.5)	77.6 (62.9, 86.5)	<0.001	0.83	1.00	0.83
	NTD3	4 (0.1) vs. 9 (0.3)	59.8 (-19.9, 86.5)	0.10	21 (0.7) vs. 96 (3.3)	80.3 (69.1, 87.4	<0.001	0.24	0.73	0.36
	NTD4	15 (0.5) vs. 59 (2.1)	77.1 (60.9, 86.6)	<0.001	10 (0.3) vs. 46 (1.6)	80.0 (61.1, 89.7)	<0.001	0.76	1.00	0.83
	NTD5 ¶§	15 (0.5) vs. 37 (1.3)	61.2 (31.3, 78.1)	0.0012	10 (0.3) vs. 68 (2.4)	88.0 (75.5, 94.1)	<0.001	0.017	0.10	0.10
	NTD6	10 (0.3) vs. 24 (0.8)	60.5 (20.0, 80.5)	0.0099	15 (0.5) vs. 81 (2.8)	83.9 (71.9, 90.8)	<0.001	0.061	0.27	0.12
	NTD7	12 (0.4) vs. 32 (1.1)	63.6 (32.2, 80.5)	0.0015	13 (0.4) vs. 73 (2.5)	85.1 (72.6, 91.9)	<0.001	0.055	0.27	0.12

[1]This column tests the null hypothesis H_{Aj0} vs. H_{Aj2} using the test statistic U_{2j} (pp 17-18 of Heng et al.[52]).
[2]This column tests the null hypothesis H_{B0} vs. H_{B2} using the test statistic T_2 (pp 18-19 of Heng et al.[52]).
¶ Unadjusted p-value for differential VE is ≤ 0.05.
§ FDR-adjusted p-value (q-value) for differential VE is ≤ 0.2 and unadjusted p-value for differential VE is ≤ 0.05.

Spike Amino Acid Position	No. of Cases (V vs. P) (Incidence per 100 PYRs)	VE (%) (95% CI)
414		
Match	216 (7.6) vs. 437 (15.9)	53.2 (44.9, 60.2)
Mismatch	4 (0.1) vs. 31 (1.1)	87.5 (64.5, 95.6)
501		
Match	104 (3.7) vs. 290 (10.5)	65.0 (56.2, 72.0)
Mismatch	116 (4.1) vs. 178 (6.5)	40.5 (24.8, 52.9)
778		
Match	219 (7.7) vs. 448 (16.3)	53.7 (45.5, 60.6)
Mismatch	1 (0) vs. 20 (0.7)	95.1 (63.8, 99.3)

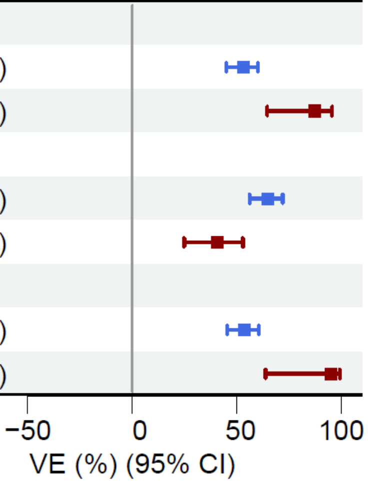

Supplementary Fig. 50
Estimation and inference in the per-protocol cohort about vaccine efficacy (VE) against the primary (moderate to severe-critical) COVID-19 endpoint with match (blue) or mismatch (maroon) to the vaccine insert at position 414, 501, and 778 in Latin America. This analysis restricts cases to lineages that are not Lambda. The filled square shows the point estimate and the horizontal bars extend through the 95% confidence interval (CI). PYRs, person-years.

Supplementary Table 25. Exact and phylogenetically corrected P values (nominal and FDR adjusted) from GenSig for the 16 sites associated with VE in Latin America (**Fig. 2d**). Fisher Exact tests are based on a 2x2 contingency table counting amino acid matches and mismatches compared to the reference sequence in the placebo and vaccine groups. Phylogenetically corrected Fisher tests are based on a 2x2 contingency table counting changes (or no change) from the ancestral node to the reference sequence amino acid in the placebo and vaccine groups. All P values are two-sided. Highlighted in bold are the internal positions of the RSYLTPGD246-253N mutation, which were significant after phylogenetic and multiple testing correction. The two flanking sites, R246 and D253, were no longer significant after multiple testing correction, however, as a sensitivity analysis, we performed an additional run with the 246-253 motifs compressed into a single mutation, and the N246 glycan was indeed significant after phylogenetic and multiple testing correction (FDR Adj p=0.002).

Site	Reference	Fisher Exact Test		Phylogenetically Corrected	
	AA	P Value	FDR Adj. P	P Value	FDR Adj. P
75	G	0.00244	1	0.0202	1
76	T	0.00244	1	0.0202	1
246	R	0.00272	1	0.0202	1
**247**	**S**	**0.00782**	**0.0929**	**0.0394**	**0.537**
**248**	**Y**	**0.00356**	**0.0159**	**0.0202**	**0.0929**
**249**	**L**	**0.00356**	**0.0159**	**0.0202**	**0.0929**
**250**	**T**	**0.00356**	**0.0159**	**0.0202**	**0.0929**
**251**	**P**	**0.00356**	**0.0159**	**0.0202**	**0.0929**
**252**	**G**	**0.00356**	**0.0159**	**0.0202**	**0.0929**
253	D	0.00517	0.543	0.0334	1
414	Q	0.186	1	0.906	1
452	L	0.00686	1	0.0373	1
490	F	0.27	0.27	1	1
501	N	0.0411	0.233	0.219	1
778	I	0.00339	1	0.0202	1
859	T	0.00244	1	0.0202	1

Supplementary Table 26. Exact and phylogenetically corrected P values (nominal and FDR adjusted) from GenSig for the 16 sites associated with VE in the United States (Supplementary Fig. S3D). Fisher Exact tests are based on a 2x2 contingency table counting amino acid matches and mismatches compared to the reference sequence in the placebo and vaccine groups. Phylogenetically corrected Fisher tests are based on a 2x2 contingency table counting changes (or no change) from the ancestral node to the reference sequence amino acid in the placebo and vaccine groups. All P values are two-sided.

Site	Reference AA	Fisher Exact Test P Value	Fisher Exact Test FDR Adj. P	Phylogenetically Corrected P Value	Phylogenetically Corrected FDR Adj. P
13	S	1	1	1	1
69	H	1	1	1	1
70	V	1	1	1	1
144	Y	1	1	1	1
152	W	1	1	1	1
452	L	0.0714	1	0.00551	1
501	N	1	1	1	1
570	A	1	1	1	1
677	Q	1	1	0.546	1
681	P	0.429	1	0.0832	1
716	T	1	1	0.784	1
716	!T	1	1	1	1
982	S	1	1	1	1
1118	I	1	1	1	1

Supplementary Table 27. Exact and phylogenetically corrected P Values (nominal and FDR adjusted) from GenSig for the 16 sites associated with VE in all regions, pooled. Fisher exact and phylogenetically corrected Fisher exact are calculated as described for Supplementary Tables 25 and 26. In rows labeled "Match" the amino acid tested is the reference sequence, whereas in rows labeled "Mismatch" we report the test against the non-reference amino acid found in the alignment that yielded the lowest nominal p-value. All P values are two-sided.

Site	Reference AA		Fisher Exact Test		Phylogenetically Corrected	
			P Value	FDR Adj. P	P Value	FDR Adj. P
13	S	Mismatch	1	1	1	1
13	S	Match	1	1	1	1
18	L	Mismatch	0.00245	0.0129	1	1
18	L	Match	0.00336	0.0162	0.582	1
19	L	Mismatch	0.00245	0.0129	1	1
19	L	Match	0.00336	0.0162	0.557	1
69	H	Mismatch	0.719	1	1	1
69	H	Match	1	1	1	1
70	V	Mismatch	1	1	1	1
70	V	Match	0.719	1	1	1
75	G	Mismatch	0.000207	0.00221	1	1
75	G	Match	0.000207	0.00221	1	1
76	T	Mismatch	0.000241	0.00272	1	1
76	T	Match	0.000241	0.00272	1	1
80	D	Mismatch	0.0754	0.212	1	1
80	D	Match	0.0875	0.239	1	1
142	G	Mismatch	0.468	1	1	1
142	G	Match	0.468	1	1	1
144	Y	Mismatch	0.0462	0.171	1	1
144	Y	Match	0.0548	0.172	1	1
152	W	Mismatch	1	1	1	1
152	W	Match	0.307	1	1	1
157	F	Mismatch	0.0933	0.512	1	1
157	F	Match	0.0933	0.512	1	1
158	R	Mismatch	1	1	1	1
158	R	Match	1	1	1	1
215	D	Mismatch	0.0677	0.197	0.298	1
215	D	Match	0.101	0.512	1	1
241	L	Mismatch	1	1	1	1
241	L	Match	1	1	1	1
242	L	Mismatch	0.0516	0.171	0.298	1
242	L	Match	0.0673	0.197	0.298	1

243	A	Mismatch	0.0393	0.159	0.507	1
243	A	Match	0.0504	0.171	0.507	1
246	R	Match	0.000151	0.00221	0.497	1
246	R	Mismatch	0.0035	0.0162	0.0864	0.474
247	S	Match	0.000976	0.00594	0.204	1
247	S	Mismatch	0.0035	0.0162	0.0283	0.116
248	Y	Match	0.000325	0.00296	0.00979	0.145
248	Y	Mismatch	0.0035	0.0162	0.00979	0.095
249	L	Match	0.000325	0.00296	0.00979	0.145
249	L	Mismatch	0.0035	0.0162	0.00979	0.095
250	T	Match	0.000566	0.00401	0.026	0.145
250	T	Mismatch	0.0035	0.0162	0.00979	0.095
251	P	Match	0.000566	0.00401	0.026	0.145
251	P	Mismatch	0.0035	0.0162	0.00979	0.095
252	G	Match	0.000325	0.00296	0.00979	0.145
252	G	Mismatch	0.0035	0.0162	0.00979	0.095
253	D	Match	0.00457	0.0217	1	1
253	D	Mismatch	0.00117	0.00218	1	1
414	Q	Match	0.0136	0.0635	1	1
414	Q	Mismatch	0.0136	0.0635	1	1
417	K	Match	0.00214	0.0123	1	1
417	K	Mismatch	0.0204	0.0721	1	1
452	L	Match	0.000104	0.00221	1	1
452	L	Mismatch	0.000207	0.00221	0.468	1
478	T	Match	1	1	1	1
478	T	Mismatch	1	1	1	1
484	E	Match	0.000862	0.0057	0.541	1
484	E	Mismatch	0.00136	0.00643	0.268	1
490	F	Match	0.000111	0.00221	0.631	1
490	F	Mismatch	0.000207	0.00221	0.59	1
501	N	Match	0.000432	0.00342	0.615	1
501	N	Mismatch	0.000697	0.0045	0.59	1
570	A	Match	0.582	1	1	1
570	A	Mismatch	0.582	1	1	1
677	Q	Match	0.84	1	0.0947	1
677	Q	Mismatch	0.0947	1	1	1
681	P	Match	0.281	1	1	1
681	P	Mismatch	1	1	1	1
701	A	Match	0.287	1	1	1
701	A	Mismatch	0.287	1	1	1
716	T	Match	0.597	1	1	1
716	T	Mismatch	0.597	1	1	1
778	T	Match	0.00721	0.0303	1	1

778	T	Mismatch	0.0119	0.0594	1	1
859	T	Match	0.000111	0.00221	1	1
859	T	Mismatch	5.27E-05	0.00221	1	1
950	D	Match	0.0206	0.0721	1	1
950	D	Mismatch	0.0469	0.171	1	1
982	S	Match	0.582	1	1	1
982	S	Mismatch	0.582	1	1	1
1118	D	Match	0.471	1	1	1
1118	D	Mismatch	0.582	1	1	1

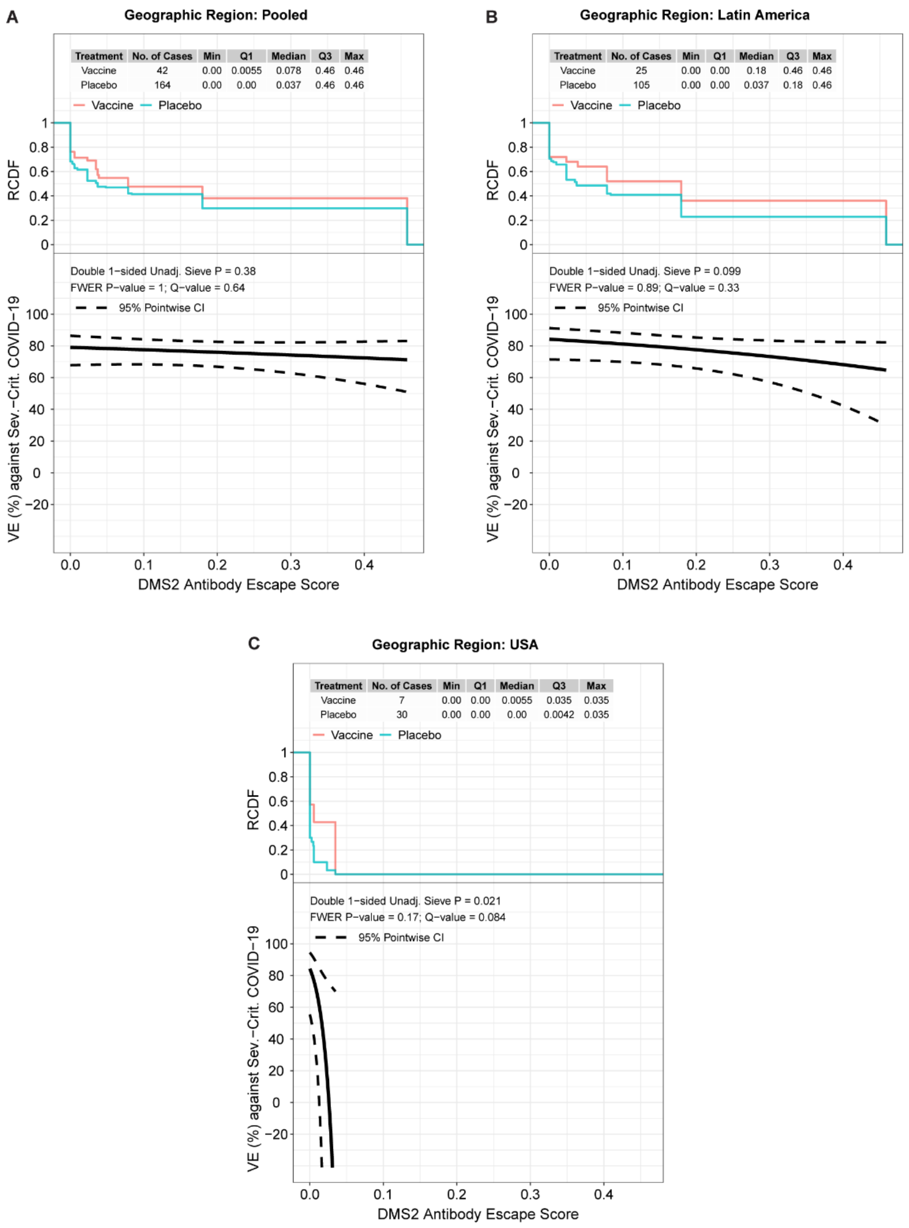

Supplementary Fig. 51

Hazard-based vaccine efficacy against the severe–critical COVID-19 endpoint by DMS2 antibody escape score of the disease-causing SARS-CoV-2 isolate. A) Geographic regions pooled, B) Latin America, C) US (note that the sequence feature for South Africa was not screened in). Dashed lines are 95% pointwise confidence intervals. The "Double 1-sided unadjusted sieve p-value" doubles the p-value from a one-sided Wald test of the null hypothesis of constant VE vs. the alternative hypothesis of a decreasing VE with an increasing value of the feature on the x-axis (Juraska and Gilbert, [53] Section 5). The plot at the top of each panel shows the reverse cumulative distribution function (RCDF) of the DMS2 antibody-binding escape score across SARS-CoV-2 isolates by treatment arm: Vaccine, pink; Placebo, turquoise.

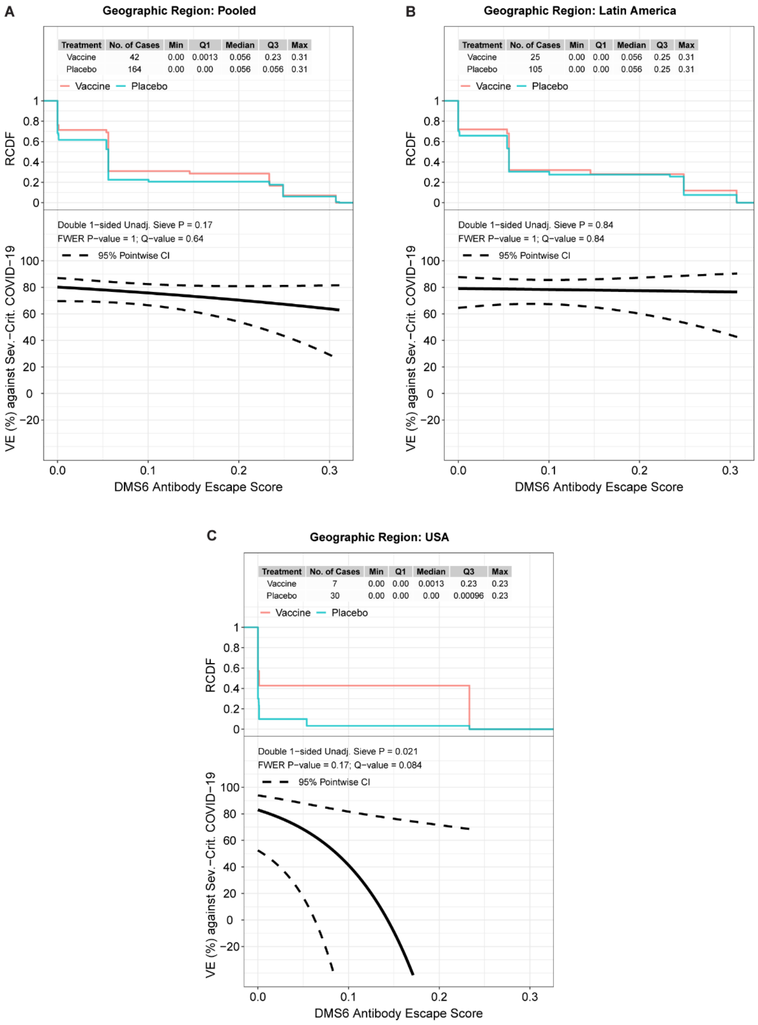

Supplementary Fig. 52

Hazard-based vaccine efficacy against the severe–critical COVID-19 endpoint by DMS6 antibody escape score of the disease-causing SARS-CoV-2 isolate. A) Geographic regions pooled, B) Latin America, C) US (note that this sequence feature for South Africa was not screened in). Dashed lines are 95% pointwise confidence intervals. The "Double 1-sided unadjusted sieve p-value" doubles the p-value from a one-sided Wald test of the null hypothesis of constant VE vs. the alternative hypothesis of a decreasing VE with an increasing value of the feature on the x-axis (Juraska and Gilbert, [53] Section 5). The plot at the top of each panel shows the reverse cumulative distribution function (RCDF) of the DMS6 antibody-binding escape score across SARS-CoV-2 isolates by treatment arm: Vaccine, pink; Placebo, turquoise.

Supplementary Table 28. **Cryo-EM data collection, refinement and validation statistics**

	**S Lambda + S309 + S2L20 + S2X303 Global Refinement** PDB 8VYE EMD-43658	**S RBD + S309 Local refinement** PDB 8VYG EMD-43660	**S Lambda + S2L20 + S2X303 Local refinement** PDB 8VYF EMD-43658
**Data collection and processing**			
Magnification	105,000	105,000	105,000
Voltage (kV)	300	300	300
Electron exposure (e–/Å^2)	63	63	63
Defocus range (μm)	-0.4 - -2	-0.4 - -2	-0.4 - -2
Pixel size (Å)	0.843	0.843	0.843
Symmetry imposed	C3	C1	C1
Initial particle images (no.)	303,264	442,254	442,254
Final particle images (no.)	147,418	90,442	95,557
Map resolution (Å)	2.7	3.1	3.2
FSC threshold	0.143	0.143	0.143
**Refinement**			
Model resolution (Å)	2.7	2.7	2.7
FSC threshold	0.143	0.143	0.143
Map sharpening *B* factor (Å^2)	-79.7	-91.5	-101.0
Model composition			
Non-hydrogen atoms	41588	3298	5851
Protein residues	5217	426	726
Glycans	75	2	13
B factors (Å^2)			
Protein	64.2	46.8	47.8
Glycans	41.6	37.8	30.3
R.m.s. deviations			
Bond lengths (Å)	0.01	0.01	0.01
Bond angles (°)	0.95	1.02	1.03
**Validation**			
MolProbity score	0.81	0.82	0.74
Clashscore	0.65	0.47	0.17
Poor rotamers (%)	0.00	0.29	0.00
Ramachandran plot			
Favored (%)	97.6	97.1	97.1
Allowed (%)	2.4	2.9	2.9
Disallowed (%)	0	0	0

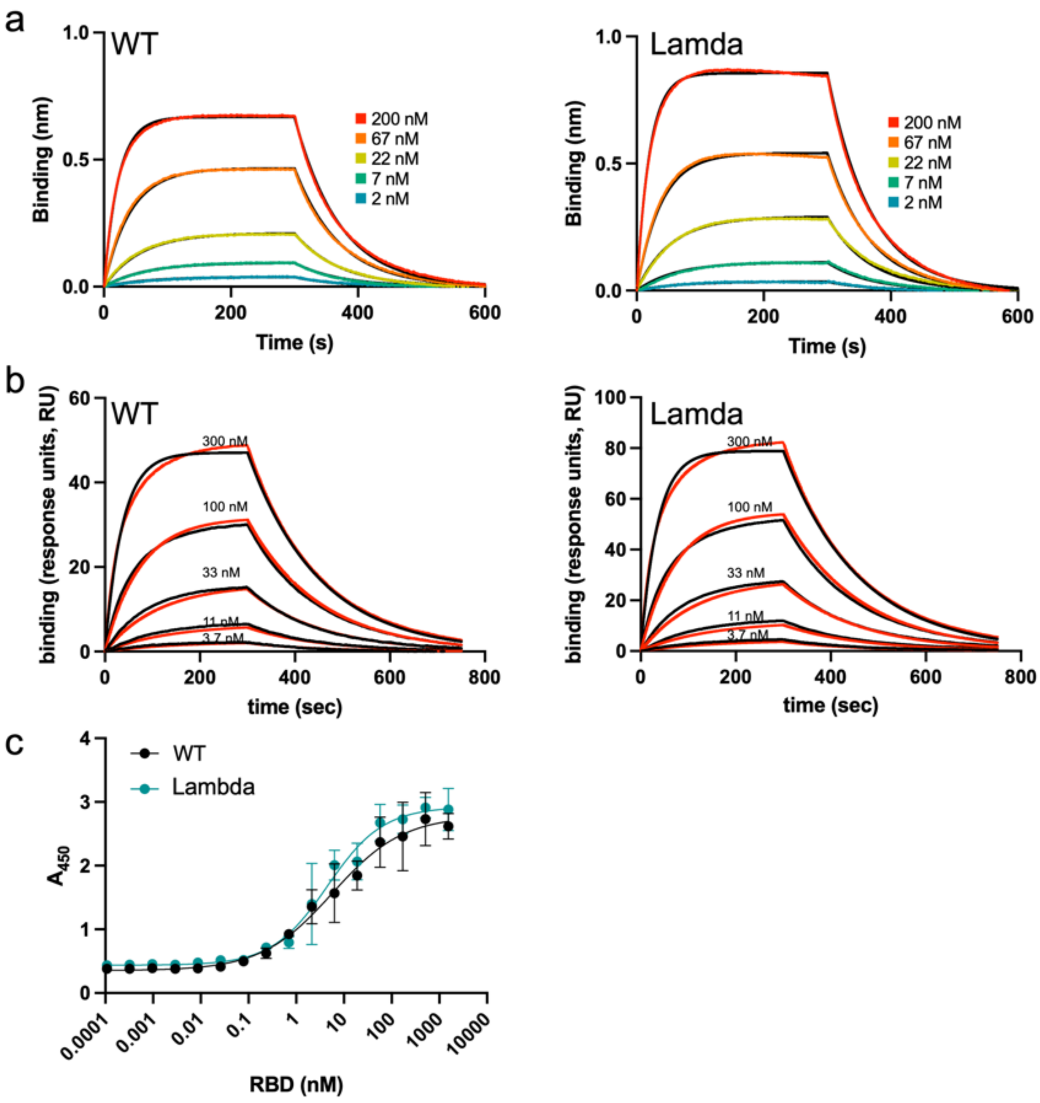

Supplementary Fig. 53
Binding kinetics of receptor binding domains (RBDs) to ACE2 using biolayer interferometry (BLI), surface plasmon resonance (SPR), and ELISA. A) Binding human ACE2 to the immobilized SARS-CoV-2 wildtype and Lambda RBDs analyzed by BLI. B) Binding of human ACE2 to the immobilized SARS-CoV-2 wildtype and Lambda RBDs analyzed by SPR. C) Binding of the SARS-CoV-2 wildtype and Lambda RBDs to immobilized human ACE2 ectodomain analyzed by ELISA. Source data are provided as a Source Data file.

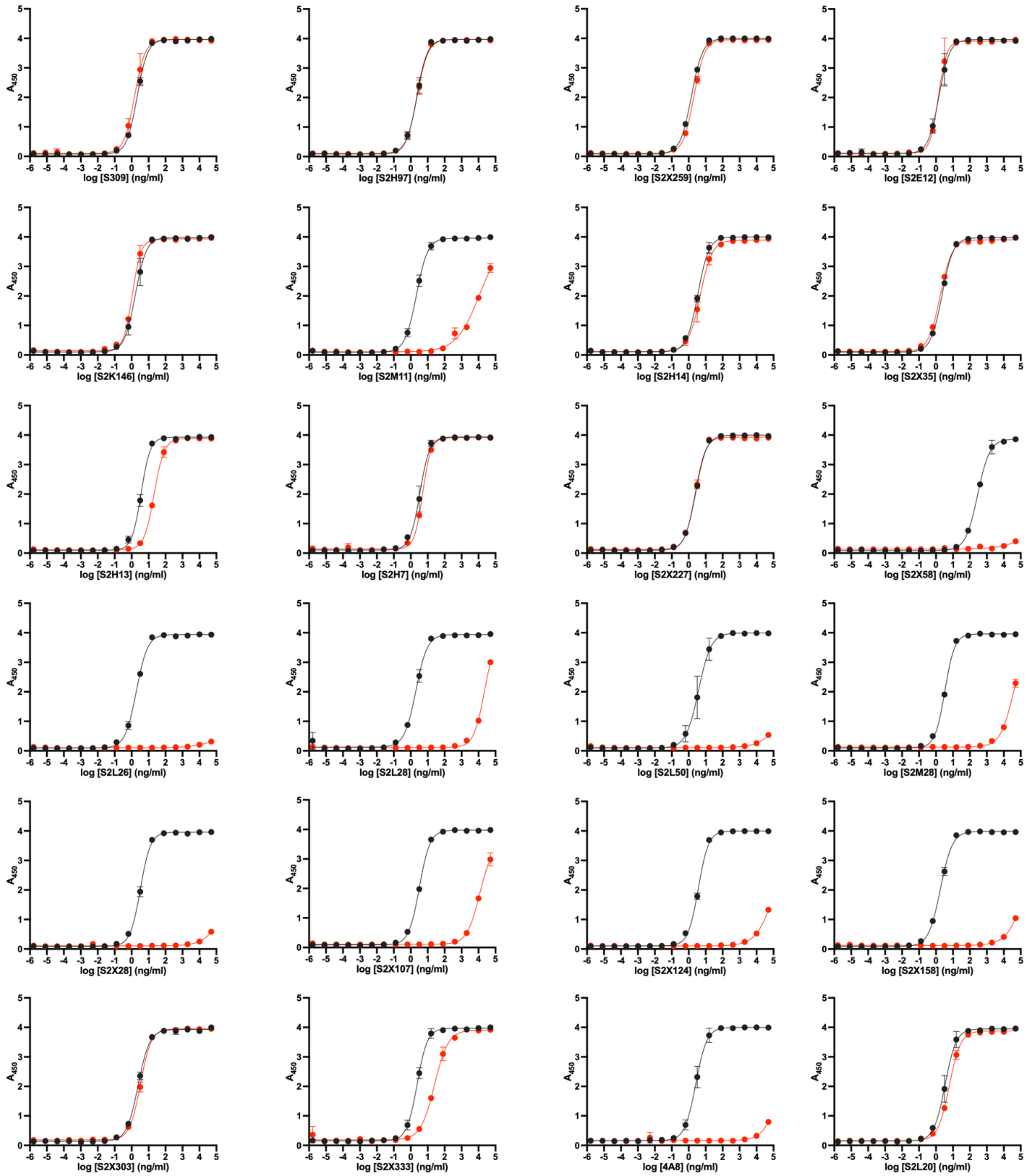

Supplementary Fig. 54

Effect of Lambda mutations on neutralizing monoclonal antibody binding. Data show binding of a panel of monoclonal antibodies (as labeled) to recombinant SARS-CoV-2 ectodomain from wildtype (black) or Lambda (red) analyzed by ELISA. Source data are provided as a Source Data file.

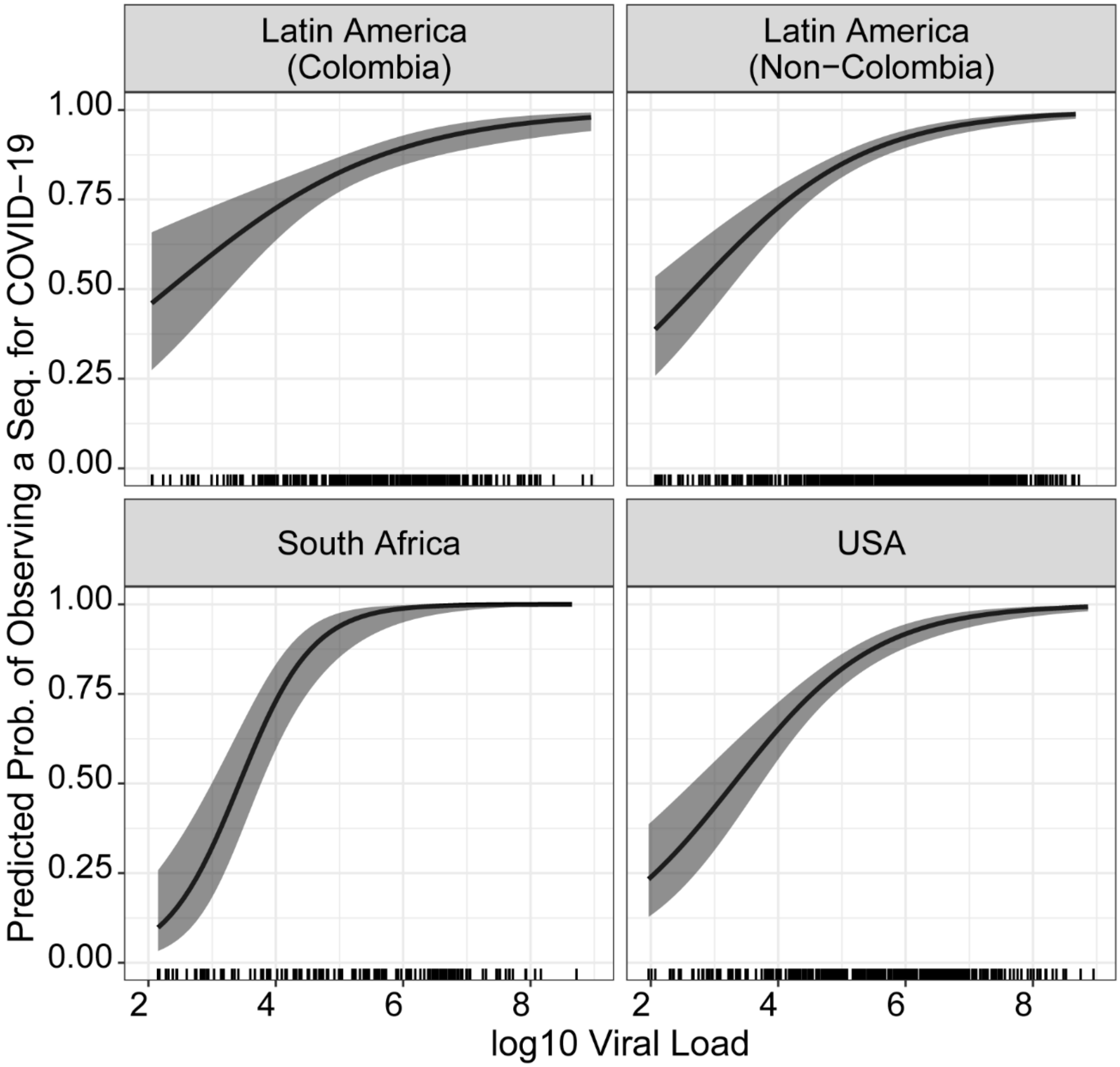

Supplementary Fig. 55
Predicted probability of observing a sequence for the primary endpoint as a function of log_{10} viral load separately by region and subregion based on region-specific logistic regressions. For Latin America, the logistic regression includes an indicator of geographic subregion (Colombia vs. Non-Colombia), log_{10} viral load and their interactions. For South Africa and USA, the logistic regression includes log_{10} viral load as the predictor. Shaded regions correspond to the pointwise 95% confidence intervals, and the rug ticks at the bottom are the observed log10 viral load measurements of primary endpoint cases.

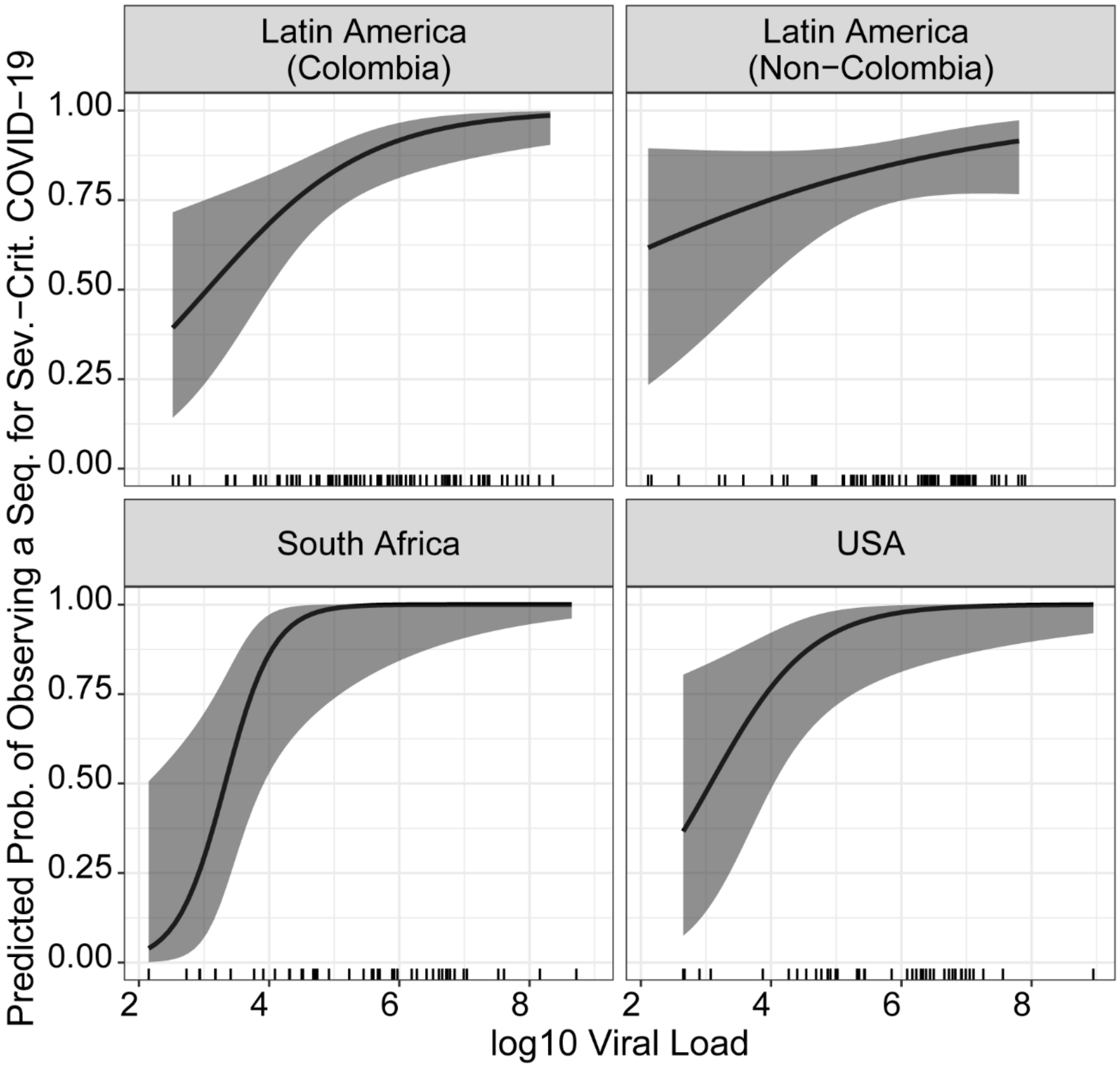

Supplementary Fig. 56
Predicted probability of observing a sequence for the severe-critical COVID-19 endpoint as a function of log_{10} viral load separately by region and subregion based on region-specific logistic regressions. For Latin America, the logistic regression includes an indicator of geographic subregion (Colombia vs. Non-Colombia), log_{10} viral load and their interactions. For South Africa and USA, the logistic regression includes log_{10} viral load as the predictor. Shaded regions correspond to the pointwise 95% confidence intervals, and the rug ticks at the bottom are the observed log10 viral load measurements of severe-critical COVID-19 endpoint cases.

Supplementary References

1. Gilbert PB, Novitsky V, Essex M. Covariability of selected amino acid positions for HIV type 1 subtypes C and B. *AIDS Res Hum Retroviruses* 2005; **21**(12): 1016-30.
2. Motozono C, Toyoda M, Zahradnik J, et al. SARS-CoV-2 spike L452R variant evades cellular immunity and increases infectivity. *Cell Host Microbe* 2021; **29**(7): 1124-36 e11.
3. Deng X, Garcia-Knight MA, Khalid MM, et al. Transmission, infectivity, and neutralization of a spike L452R SARS-CoV-2 variant. *Cell* 2021; **184**(13): 3426-37 e8.
4. Acevedo ML, Gaete-Argel A, Alonso-Palomares L, et al. Differential neutralizing antibody responses elicited by CoronaVac and BNT162b2 against SARS-CoV-2 Lambda in Chile. *Nat Microbiol* 2022; **7**(4): 524-9.
5. Liu Y, Liu J, Plante KS, et al. The N501Y spike substitution enhances SARS-CoV-2 infection and transmission. *Nature* 2022; **602**(7896): 294-9.
6. Sandberg M, Eriksson L, Jonsson J, Sjostrom M, Wold S. New chemical descriptors relevant for the design of biologically active peptides. A multivariate characterization of 87 amino acids. *J Med Chem* 1998; **41**(14): 2481-91.
7. Bhattacharya T, Daniels M, Heckerman D, et al. Founder effects in the assessment of HIV polymorphisms and HLA allele associations. *Science* 2007; **315**(5818): 1583-6.
8. Bricault CA, Yusim K, Seaman MS, et al. HIV-1 Neutralizing Antibody Signatures and Application to Epitope-Targeted Vaccine Design. *Cell Host Microbe* 2019; **25**(1): 59-72 e8.
9. Folashade D, Microsoft Corporation, Weston S, Tenenbaum D. doParallel: Foreach Parallel Adaptor for the 'parallel' Package. R package. https://CRAN.R-project.org/package=doParallel 2022.
10. Folashade D, Ooi H, Calaway R, Microsoft, Weston S. foreach: Provides Foreach Looping Construct. R package. https://CRAN.R-project.org/package=foreach. 2022.
11. Folashade D, Revolution Analytics, Weston S. iterators: Provides Iterator Construct. R package. https://CRAN.R-project.org/package=iterators. 2022.
12. Mueller K, Bryan J. here: A Simpler Way to Find Your Files. https://CRAN.R-project.org/package=here 2020.
13. Wickham H, Miller E, Smith D, Posit Software, PBC. haven: Import and Export 'SPSS', 'Stata' and 'SAS' Files. R package. https://cran.r-project.org/package=haven. 2023.
14. Wickham H, Pedersen T, Seidel D. scales: Scale Functions for Visualization. R package, https://github.com/r-lib/scales, https://scales.r-lib.org. 2023.
15. Wickham H, et al. "Welcome to the tidyverse." Journal of Open Source Software, 4(43), 1686. doi:10.21105/joss.01686. . 2019.
16. Wickham H. The Split-Apply-Combine Strategy for Data Analysis. https://www.jstatsoft.org/v40/i01/. . *Journal of Statistical Software* 2011; **40**(1): 1-29.
17. Wickham HF, R., Henry L, Muller K, Vaughan D. dplyr: A Grammar of Data Manipulation. R package, https://github.com/tidyverse/dplyr, https://dplyr.tidyverse.org 2023.
18. Venables WN, Ripley BD. Modern Applied Statistics with S, Fourth edition. Springer, New York. ISBN 0-387-95457-0, https://www.stats.ox.ac.uk/pub/MASS4/. 2002.
19. Kassambara A. ggpubr: 'ggplot2' Based Publication Ready Plots. R package version 0.6.0, https://rpkgs.datanovia.com/ggpubr/. 2023.
20. Aphalo P. ggpmisc: Miscellaneous Extensions to 'ggplot2'. R package. https://github.com/aphalo/ggpmisc, https://docs.r4photobiology.info/ggpmisc/. 2023.
21. Tiedemann F. ggpol: Visualizing Social Science Data with 'ggplot2'. R package. https://cran.r-project.org/package=ggpol. 2020.
22. Auguie B, Antonov A. gridExtra: Miscellaneous Functions for "Grid" Graphics. R package. https://cran.r-project.org/package=gridExtra. 2017.
23. Wickham H, Pedersen TL, Posit Software, PBC. gtable: Arrange 'Grobs' in Tables. R package. https://cran.r-project.org/package=gtable. 2023.
24. Bion R. ggradar. R package. https://github.com/ricardo-bion/ggradar.

25. Wickham H, Vaughan D, Girlich M. tidyr: Tidy Messy Data. R package version 1.3.0, https://github.com/tidyverse/tidyr, https://tidyr.tidyverse.org. 2023.
26. Wickham H, Hester J, Francois R, Bryan J, Bearrows S. readr: Read Rectangular Text Data. https://readr.tidyverse.org, https://github.com/tidyverse/readr. 2023.
27. Davis TL, Day A, Python Software Foundation, Newell P. argparse: Command Line Optional and Positional Argument Parser. R package. https://CRAN.R-project.org/package=argparse 2023.
28. van der Laan MJ, Polley EC, Hubbard AE. Super learner. *Stat Appl Genet Mol Biol* 2007; **6**: Article25.
29. Polley EC, van der Laan MJ. Super Learner in Prediction. U.C. Berkeley Division of Biostatistics Working Paper Series. Paper 226. http://biostats.bepress.com/ucbbiostat/paper266/. 2010.
30. Gelman A, Su Y-S, Yajima M, et al. arm: Data Analysis Using Regression and Multilevel/Hierarchical Models. https://CRAN.R-project.org/package=arm 2022.
31. Karatzoglou A, Smola A, Hornik K, Zeileis A. kernlab - An S4 Package for Kernel Methods in R. *Journal of Statistical Software* 2004; **11**(9): 1 - 20.
32. Wright MN, Ziegler A. ranger: A Fast Implementation of Random Forests for High Dimensional Data in C++ and R. *Journal of Statistical Software* 2017; **77**(1): 1 - 17.
33. Chen T, Guestrin C. XGBoost: A Scalable Tree Boosting System. Proceedings of the 22nd ACM SIGKDD International Conference on Knowledge Discovery and Data Mining. San Francisco, California, USA: Association for Computing Machinery; 2016. p. 785–94.
34. Friedman J, Hastie T, Tibshirani R. Regularization paths for generalized linear models via coordinate descent. *Journal of Statistical Software* 2010; **33**(1): 1.
35. Milborrow S. Derived from mda:mars by T. Hastie and R. Tibshirani. earth: Multivariate Adaptive Regression Splines. R package 2011.
36. Mueller K, Wickham H, Francois R, Bryan J, RStudio. tibble: Simple Data Frames. https://tibble.tidyverse.org/, https://github.com/tidyverse/tibble. 2023.
37. Williamson BD, Gilbert PB, Carone M, Simon N. Nonparametric variable importance assessment using machine learning techniques. *Biometrics* 2021; **77**(1): 9-22.
38. Williamson B, Gilbert PB, Simon N, Carone M. A General Framework for Inference on Algorithm-Agnostic Variable Importance. doi: 10.1080/01621459.2021.2003200. *Journal of the American Statistical Association* 2022.
39. Williamson BD, Feng J, Wolock C, Simon N, Carone M. vimp: Perform Inference on Algorithm-Agnostic Variable Importance. R package version 2.3.3. URL https://CRAN.R-project.org/package=vimp. 2023.
40. Wilke C. cowplot: Streamlined Plot Theme and Plot Annotations for 'ggplot2'. R package version 1.1.2, https://wilkelab.org/cowplot/. 2023.
41. Barrett T, Dowle M, Srinivasan A. data.table: Extension of 'data.frame'. R package version 1.14.99, https://Rdatatable.gitlab.io/data.table, https://github.com/Rdatatable/data.table, https://r-datatable.com. 2023.
42. Davis TL, et al. optparse: Command Line Option Parser. R package. https://CRAN.R-project.org/package=optparse. 2022.
43. Xie Y. knitr: A General-Purpose Package for Dynamic Report Generation in R. R package version 1.45, https://yihui.org/knitr/. 2023.
44. Greaney AJ, Starr TN, Bloom JD. An antibody-escape estimator for mutations to the SARS-CoV-2 receptor-binding domain. *Virus Evol* 2022; **8**(1): veac021.
45. Greaney AJ, Starr TN, Barnes CO, et al. Mapping mutations to the SARS-CoV-2 RBD that escape binding by different classes of antibodies. *Nat Commun* 2021; **12**(1): 4196.
46. Barnes CO, Jette CA, Abernathy ME, et al. SARS-CoV-2 neutralizing antibody structures inform therapeutic strategies. *Nature* 2020; **588**(7839): 682-7.
47. Sadoff J, Le Gars M, Brandenburg B, et al. Durable antibody responses elicited by 1 dose of Ad26.COV2.S and substantial increase after boosting: 2 randomized clinical trials. *Vaccine* 2022; **40**(32): 4403-11.

48. Korber B, Fischer WM, Gnanakaran S, et al. Tracking Changes in SARS-CoV-2 Spike: Evidence that D614G Increases Infectivity of the COVID-19 Virus. *Cell* 2020; **182**(4): 812-27 e19.
49. Sadoff J, Gray G, Vandebosch A, et al. Safety and Efficacy of Single-Dose Ad26.COV2.S Vaccine against Covid-19. *N Engl J Med* 2021; **384**(23): 2187-201.
50. R Core Team. R: A Language and Environment for Statistical Computing. R Foundation for Statistical Computing, Vienna, Austria. https://www.R-project.org/. 2022.
51. Liu Y, Liu J, Xia H, et al. Neutralizing Activity of BNT162b2-Elicited Serum. *N Engl J Med* 2021; **384**(15): 1466-8.
52. Heng F, Sun Y, Gilbert PB. Estimation and Hypothesis Testing of Strain-Specific Vaccine Efficacy with Missing Strain Types, with Applications to a COVID-19 Vaccine Trial. arXiv:2201.08946 [stat.ME] https://doi.org/10.48550/arXiv.2201.08946. 2022.
53. Juraska M, Gilbert PB. Mark-specific hazard ratio model with missing multivariate marks. *Lifetime Data Anal* 2016; **22**(4): 606-25.
54. Juraska M, Gilbert PB. Mark-specific hazard ratio model with multivariate continuous marks: an application to vaccine efficacy. *Biometrics* 2013; **69**(2): 328-37.

ENSEMBLE Phase 3 Trial Sieve Analysis Statistical Analysis Plan

Coronavirus Prevention Network Biostatistics
Fred Hutchinson Cancer Center

Peter Gilbert, Morgane Rolland, Allan deCamp, Michal Juraska, Li Li, James Ludwig, Cindy Molitor, Brian Williamson, Ollivier Hyrien, David Benkeser, Craig Magaret

October 24, 2023

Version 1.0

Contents

List of Tables 5

List of Figures 7

1 Introduction and Design of Sieve Analysis 8
1.1 Three stages of sieve analysis for a Phase 3 trial . . . 9
1.2 Study endpoints for sieve analysis . . . 10
1.3 Unsupervised learning to specify AA sequence features for sieve analysis . . . 11
1.3.1 **All** Spike AA sequence features . . . 11
1.3.2 Rationale for **nAb-CoP-hypothesis** Spike AA sequence features . . . 12
1.4 Supervised learning sieve analysis (to detect sieve effects) . . . 12
1.5 Cohorts for AA sequence sieve analyses, and COVID-19 endpoints included in the analyses . . . 13
1.6 Filters/screens for sufficient variability of a mark feature for conducting sieve analysis 13
1.7 Sequencing technology and samples obtained for sequencing (ENSEMBLE) . . . 14

2 AA Sequence Sieve Analysis Methods: Approach A and Approach B 14
2.0.1 Overall cumulative-incidence based sieve analysis . . . 16
2.0.2 Geographic region-specific sieve analyses . . . 16
2.1 Structuring AA sequence features for sieve analysis . . . 17
2.1.1 All features: All variants as defined by WHO . . . 18
2.1.2 All features: All AA position features in Spike . . . 18
2.1.3 All features: Physicochemical-weighted (Sandberg et al., 1998) Hamming distances to the vaccine strain (S, RBD, NTD, S1, S2) . . . 18
2.1.4 bnAb CoP-hypothesis driven features: AA features based on Deep Mutational Scanning RBD antibody-escape scores impacting neutralization . . . 19
2.1.5 bnAb CoP-hypothesis driven features: AA features based on Spike antibody-escape scores impacting neutralization from the Protein Data Bank . . . 19
2.1.6 bnAb CoP-hypothesis driven features: NTD neutralization-relevant features (all features binary)[1] . . . 22
2.1.7 bnAb CoP-hypothesis driven features: Variant-neutralization sensitivity score assigned to variants . . . 24
2.2 Listing of application of AA sequence sieve analyses methods for the **All feature** sequence sets and the **nAb-CoP-hypothesis feature** sequence sets . . . 25
2.3 Graphical descriptive analyses of AA sequence features . . . 26
2.3.1 Example descriptive plots for expected mark distributions . . . 27
2.4 Approach to handling missing sequences/marks . . . 28
2.4.1 Defining the viral load auxiliary variable accounting for multiple viral load measurements . . . 29
2.5 Details of the Approach A conditional sieve analysis method . . . 29
2.6 Details of the Approach B hazard-based VE sieve analysis methods . . . 31
2.7 Details of the Approach B cumulative incidence based VE sieve analysis method . . 32

2.8 Understanding the missing sequence data and adapting the statistical methods . . . 33
2.8.1 Diagnostics to help decide the versions of the methods to use 33
2.9 Superlearner classification sieve analysis 34
2.10 Multiple hypothesis testing adjustment for AA sequence sieve analysis 37

3 Appendix A: Additional statistical details of sieve analysis methods 42
3.1 Details of the Approach B sieve analysis methods 42
3.2 R code for the sieve analysis methods 44

List of Tables

1 List of AA Sequence Features V for Sieve Analysis: **All Features** 17
2 List of AA Sequence Features V for Sieve Analysis: **nAb-CoP-hypothesis** 17
3 RBD-antibody binding escape score based putative epitope footprints. For each cluster (footprint) this table gives the number of antibodies per cluster (n), the number of sites per cluster (n sites), and the cluster site list. Singleton clusters 4 and 10 will be excluded from the analysis. 20
4 RBD-antibody binding escape score based putative epitope footprints: important sites for neutralization 20
5 Epitope footprints for antibodies representative of 14 clusters. 274 Spike antibodies were grouped in 14 clusters based on the similarity of their epitope footprint. Each cluster includes at least 3 antibodies. The epitope footprint and the weight of the interaction are provided for each epitope site. 22
6 Epitope footprints for antibodies representative of 14 clusters. 274 Spike antibodies were grouped in 14 clusters based on the similarity of their epitope footprint. Each cluster includes at least 3 antibodies. The epitope footprint and the weight of the interaction are provided for each epitope site (continued from Table 5). 23
7 Neutralization activity of serum antibodies elicited by Ad26.COV2.S against SARS-CoV-2 spike pseudotyped virus variants. Serum samples (N=8) from participants in the phase 3 ENSEMBLE trial immunized with one dose of 5 $\times 10^{10}$ viral particles of Ad26.COV2.S were obtained on day 71 past immunization. Samples were tested in a pseudotyped lentivirus neutralization assay on SARS-CoV-2 spike protein variants of concern, measuring neutralizing titers with the pseudovirus neutralization assay at a 50% inhibitory concentration (IC, serum dilution). The table reports, for each virus variant, the geometric mean titer (GMT) and the fold changes compared to B.1. The P-value tests whether the GM Fold-Change differs between the variant and the Reference virus B.1. 25
8 Sieve analysis methods applied to the **All AA sequence features** 26
9 Sieve analysis methods applied to the **nAb-CoP-hypothesis** AA sequence features 26
10 Candidate learners in the Super Learner ensemble for predicting randomization arm, along with their R implementation, tuning parameter values, and description of the tuning parameters. All tuning parameters besides those listed here (and below) are set to their default values. In particular, stepwise regression is done via AIC (setting $k = 2$ in `step`); SVMs are fit with the radial basis (Gaussian) kernel, automatic hyperparameter selection, no cross-validation, $\nu = 0.2$, and $C = 1$; multivariate adaptive regression splines are fit allowing two-way interactions, no cross-validation, maximum number of model terms in the forward pass equal to $\max\{21, 2p + 1\}$, backward pruning, and no mininum number of observations between knots or before the first and after the final knot; the random forests are grown with 500 trees, a subsampling fraction of 0.632, and select $\sqrt{p}$ features to possibly split on at each node; the boosted trees are grown with a maximum of 500 trees, max depth of 4, and a minimum of 10 observations per node. The total number of features is denoted by p. †: $\alpha = 1$ denotes the lasso (i.e., no ℓ_2 regularization). 35

11 R code for implementing the sieve analyses, where all methods account for missing SARS-CoV-2 sequences from some COVID-19 endpoint cases 44

List of Figures

1 Concept of sieve analysis 45

2 Amino acid position scanning across Spike, showing for each position the number of sequences with a residue mismatch to the Wuhan vaccine strain: 1122 randomly sampled sequences from GISAID from U.S. study sites between September 8 2020 and February 1 2021. If these were the trial sequences, the conclusion would be that 25 of the 1273 amino acid positions would be included in the sieve analysis (sufficient variability). Special note: almost all observed sequences have the G614 mutation, which differs from the D614 in the vaccine strain. This results in the displayed variability at site 614 exceeding the y-axis's upper limit. In this sample, however, only two sequences have D614, which does not equal or exceed the 20-sequence threshold, so site 614 would not be included in the sieve analysis. 46

3 Hamming distances to the Wuhan vaccine strain: 1122 randomly sampled sequences from GISAID from U.S. trial study sites from September 8 2020 to February 1 2021. If these were the trial sequences, the conclusion would be that the all five Hamming distances would be included in the sieve analysis (sufficient variability). 47

4 Based on all ENSEMBLE trial primary moderate to severe-critical COVID-19 endpoints starting 14 days after vaccination with sequence data, the plot shows clusters representing major antibody epitope footprints in SARS-CoV-2 Spike. Epitope footprints are represented across the Spike sequence for 274 antibodies. Antibodies with similar epitope footprints are grouped in 14 clusters. Each cluster includes at least 3 antibodies. A representative antibody was identified for each cluster and these representative antibodies are indicated by a line spanning the epitope footprint. . . . 48

1 Introduction and Design of Sieve Analysis

This SAP describes the statistical analyses for addressing the sieve analysis exploratory objective in the ENSEMBLE Phase 3 trial, a randomized, placebo-controlled COVID-19 vaccine efficacy trial:

Exploratory Objective: To evaluate the genetic and/or phenotypic relationships of isolated SARS-CoV-2 strains to the vaccine sequence.

The basis of addressing this objective is measurement of SARS-CoV-2 Spike protein sequences from trial participants who experienced the symptomatic infection/COVID-19 primary study endpoint. The sieve analyses restrict to per-protocol primary endpoints starting 14 days post vaccination. The sieve analyses focus on baseline SARS-CoV-2 seronegative individuals, again mirroring the population for primary analyses of vaccine efficacy.

For all symptomatic infection/COVID-19 endpoint cases, a metagenomic sequencing approach is applied based on RT-PCR of NP swab, or nasal swab, or saliva samples drawn at the COVID-19 diagnosis time point (first RNA positive time point sometimes referred to as the "illness visit"), or shortly afterwards if not available. When the sequencing was successful, the sequence variable for analysis is the consensus sequence from the set of sequencing reads. When the sequencing fails, for example due to low sample viral load, the result of a missing value is noted, and any auxiliary variables that may be predictive of whether the sequencing fails. The statistical methods account for missing sequence data.

Based on the sequences observed in COVID-19 case vaccine recipients in comparison to COVID-19 case placebo recipients (Figure 1), sieve analysis addresses whether and how vaccine efficacy (VE) depend on SARS-CoV-2 viral features. A measurable viral feature that modifies vaccine efficacy can be thought of as a biomarker of the virus that can be useful for predicting the level of vaccine efficacy against a specific virus. We use the terminology "sieve effect" to denote statistical evidence that VE differs across the levels of a given AA sequence feature (e.g., VE lower against SARS-CoV-2 with vaccine-mismatched residue K at Spike site 484 than against SARS-CoV-2 with vaccine-matched residue E at Spike site 484 would be a sieve effect). In the statistical literature, a sequence feature is often referred to as a "mark" variable, to denote that the feature is only meaningfully defined in endpoint failure cases, which distinguishes it from a covariate that is well defined before failure.

Sieve analysis can also provide insights into potential molecular causes of vaccine failure. For example, if vaccine efficacy is found to be lower against viruses with an amino acid (AA) mismatch to the vaccine strain at a particular Spike AA position, and it is also found that vaccinee sera neutralize the wildtype (vaccine-matched) virus better than the vaccine-mismatched virus, this result suggests a molecular mechanism of vaccine failure. Rolland and Gilbert (2021) briefly discussed motivation and applications of sieve analysis in SARS-CoV-2 vaccine efficacy trials. Through these various investigations, sieve analysis informs models for predicting vaccine efficacy against populations of viruses and for optimizing vaccine strain selection.

The virus features measured in COVID-19 cases can be of two types: Immunological phenotype characteristics, and AA sequence characteristics. An immunological phenotype of interest is virus

neutralization sensitivity to vaccinee sera from a post vaccination immunogenicity time point (such as 4 weeks post vaccination), which, for example, could be defined by geometric mean serum pseudovirus nAb ID50 titer to a COVID-19 cases's virus measured using post vaccination sera from a random sample of per-protocol vaccine recipients in the case-cohort immune correlates study. Virus sensitivity to other immune functions (e.g., Fc effector functions such as ADCP) could also be characterized. The goal of immunological phenotype sieve analysis, which may be called neutralization sieve analysis in the case of a neutralization assay, is to discriminate which immune functions, and which immunoassays, are important as correlates of protection. In this SAP, we restrict attention to virus features defined solely by AA sequence features (with one exception the 'variant-neutralization resistance score' defined below), although importantly the selection and specification of these features may be informed by immunological data as discussed below.

1.1 Three stages of sieve analysis for a Phase 3 trial

We structure the sieve analysis in three stages.

1. (Placebo-controlled period:) Sequence the *spike* gene from all COVID-19 endpoints occurring post dose 1, obtaining one consensus Spike AA sequence per endpoint. Restricting to endpoints starting 14 days post dose 1, conduct treatment-blinded unsupervised learning to define the AA sequence features for sieve analysis. Assess VE and differential VE by all genotypes that are defined by the unsupervised learning.

2. (Placebo-controlled period:) Prepare for neutralization sieve analysis that may be pursued, by running prototypic SARS-CoV-2 strains in pseudovirus nAb assays using vaccinee sera from the Day 29 or D71 time point post-vaccination. These data are used to construct a mapping of Spike protein sequences to neutralization resistance level to vaccinee sera via machine learning. In addition, knowledge of the literature is used to specify AA sequence features relevant for neutralization. These tasks enable study of how vaccine efficacy depends on a sequence feature defined as predicted neutralization sensitivity to vaccinee sera.

3. (Post placebo-controlled period:) Assess durability of VE by genotype (and possibly by phenotype) and differential VE by genotype / phenotype, based on sequence data from COVID-19 cases diagnosed after unblinding and after cross-over of placebo recipients to receive the vaccine under EUA.

The first two stages restrict to the pre-unblinding/pre-crossover period, and thus validity of inferences is relatively straightforward based on randomization and double-blinding. In contrast Stage 3 is more complicated as sieve analysis is done based on events occurring after unblinding, and the placebo arm is lost. Likely the Stage 3 sieve analysis will be based on comparing the relative frequency of sequence features of acquired viruses in vaccine recipients compared to distributions in the GISAID sequence database that are "matched" to the time period and geography of follow-up of ENSEMBLE trial participants.

Given that Stage 3 is more complicated than Stages 1 and 2, the remainder of this SAP restricts to Stages 1 and 2, and the sieve analysis manuscript Magaret et al. restricts to Stages 1 and 2. At a later point in time it will be decided whether to pursue Stage 3 sieve analysis, and the SAP would be updated to accomplish that purpose.

Connected to Stage 2, the priority level of directly characterizing neutralization sensitivity phenotypes (to peak antibody vaccinee sera) of COVID-19 endpoint case viruses will be explored. If this neutralization sieve analysis is pursued, then it would be sufficient to measure the virus neutralization sensitivity phenotype from only a random sample of placebo arm COVID-19 endpoints, to limit resource expenditure. The current SAP does not include direct neutralization sieve analysis. However, it does include a 'variant-neutralization resistance score' sieve analysis based on neutralization experiments (Section 2.1.7).

1.2 Study endpoints for sieve analysis

The main endpoint for sieve analysis is the same COVID-19 primary endpoint that was used in the primary analyses (Sadoff et al., 2021, 2022). While these papers conducted analyses both counting endpoints starting 14 or 28 days post vaccination, all of the sieve analyses restrict to starting 14 days post vaccination, given that similar results are expected and this allows including more COVID-19 endpoints in the analysis.

In addition, sieve analysis is also conducted for severe-critical COVID-19, again using the same definition as used in the primary analyses.

Viral load measurements used in this analysis had to be obtained from a sample drawn within five days following the infection's RNA-positive date. Some participants had multiple VL readouts from within the five-day window. In these cases, the VL readout obtained closest to the RNA-positive date was used in the analysis. If multiple VL readouts were available on the same (earliest) date, then the mean of the readouts was used in the analysis. If no VL readouts were available within five days following the RNA-positive date, then this patient was regarded as missing VL data.

Some primary endpoint cases had multiple sequences available, typically from different (multiple) infections. Unless otherwise specified, any analyses described in this SAP pertain to the participants' first infections leading to COVID-19 primary endpoints.

For a sequence to be associated with a primary endpoint case, it had to have been obtained from a sample drawn within 0-36 days following the RNA-positive date of a given COVID-19 endpoint event. This range allows for participants to have had samples drawn and then return to the site on Day 29 following their diagnosis (as specified by the protocol), and within the seven-day grace period.

If a participant did not have a sequence from within the 0-36-day window of a given illness-onset/COVID-19 endpoint event, but had a sequence available from a later COVID-19 event, then that participant is regarded as missing the sequence for the earlier COVID-19 event.

For each of the two study endpoints and SARS-CoV-2 sequence feature, a filter is applied to decide whether the feature has enough variability to warrant sieve analysis. The filter differs by endpoint type, being more permissive (requiring less variability) for the severe-critical disease endpoint. These filters are defined in Section 1.6.

1.3 Unsupervised learning to specify AA sequence features for sieve analysis

To conduct sieve analysis, first unsupervised learning of the treatment-blinded trial sequence data set is done. This process fully specifies / down-selects the set of AA sequence features that will be studied for sieve effects, and leverages external knowledge and data. Through this process, all statistical inferences are pre-specified in the SAP before treatment unblinding. Then, inferential statistical analysis (supervised learning that produces VE estimates, relative VE estimates, confidence intervals and p-values) is conducted in automated/press-button fashion, with the inferences valid based on the pre-specification of inferences and the reproducibility of the computer coding.

Once the Phase 3 trial sequence data set is available, treatment-blinded unsupervised learning is conducted to define Spike AA sequence features of interest. Variability of a variety of features derived from the Spike AA sequence will be displayed graphically. We divide the features into two kinds. The first kind of features are all Spike AA sequence features with sufficient variability to study for potential sieve effects, essentially an unbiased analysis by considering the full set of features. We call these **All features**. The second kind of features is the subset of **All features** that are directly connected to a hypothesis that neutralizing antibodies are a correlate of protection, and are selected based on knowledge/data or hypotheses that different levels of the feature affect neutralization. We call these **nAb-CoP-hypothesis features**.

1.3.1 All Spike AA sequence features

1. The variant status for the virus. This is indicated by the Greek-lettered label defined by the WHO's SARS-CoV-2 Variant Classifications and Definitions, or as the Wuhan ancestral lineage.
2. All AA position features in Spike, and their match status to the vaccine insert
3. Physicochemical-weighted Hamming distances of the virus to the vaccine strain for different viral regions, including all of Spike (including the signal peptide), RBD, NTD, subunit S1, and subunit S2 (for five Hamming distances total)

One purpose of the descriptive analyses is to define AA sequence features that have enough variability across COVID-19 endpoint cases to potentially detect a sieve effect. Given limited Spike protein variability during the primary period of trial follow-up (e.g., Figure 1 in Rolland and Gilbert 2021, showing that circulating strains during the blinded period of the Moderna COVE trial are expected to have a median of 2 AA mismatches to the Wuhan vaccine strain in the Spike protein), it is known that a large winnowing will occur. Quantitative ordinal or continuous score/distance viral marks have advantage for low-diversity sequence data that they can be evaluable for sieve effects as long as at least 20 COVID-19 cases (pooled over treatment arms) have viral mark value different from the most frequent value and at least 5 COVID-19 cases have viral mark value different from the most and the second most frequent mark value (our specified guideline for sufficient variability). Previous sieve analyses of an RTS,S malaria vaccine efficacy trial may most closely resemble the level of pathogen variation available for sieve analysis for the blinded-period objectives of the ENSEMBLE trial sieve analysis (Neafsey et al., 2015; Follmann and Huang, 2018; Benkeser et al., 2020).

1.3.2 Rationale for nAb-CoP-hypothesis Spike AA sequence features

The rationale for conducting a sieve analysis focused on **nAb-CoP-hypothesis** features is that neutralizing antibodies are strongly hypothesized to be a correlate of protection, with data from the Phase 3 trials supporting this hypothesis (Gilbert et al., 2022; Fong et al., 2022, 2023) and sieve analysis is an integral part of immune correlates evaluation as the other side of the same coin. In this metaphor the 'tails side' is host marker immune CoP analyses, which assess how the vaccine-elicited antibody response to specific viral sequences links to vaccine efficacy. The 'heads side' is viral sequence sieve analysis of how VE depends on viral sequences from participants acquiring the COVID-19 endpoint. Yet because antibodies recognize specific viral epitopes, it is natural to think of immune CoP analyses and sieve analysis as part of the same investigation, enquiring how does VE against feature-specific disease depend on the vaccine-elicited antibody response? For instance, if certain epitope-specific immune responses block acquisition of disease, then we expect to see a relative absence of these epitope sequences in COVID-19 endpoint vaccine recipients compared to COVID-19 endpoint placebo recipients (a sieve effect). Insights from the immunogenicity and CoP analyses will be accounted for in helping to define AA features that are hypothesized to impact VE through neutralization. Another motivation to conduct sieve analysis for a set of **nAb-CoP-hypothesis** features is to improve statistical power: The dimensionality of the set of features may be reduced compared to the **All features** sieve analyses, thus lowering the multiple testing penalty and improving power. Rolland, Edlefsen et al. (2012) is an example of a sieve analysis that focused on immune-correlate-hypothesized and antibody-relevant Envelope sequence features.

In sum, based on the treatment-blinded descriptive analysis, interrogation of literature and data on knowledge of the genotype-serotype map, AA sequence features with sufficient variability for possible detection of a sieve effect are down-selected into one of two sets of features: **All** and **nAb-CoP-hypothesis**. Section 2.1 and Tables 1 and 2 list the AA sequence features that are studied in the unsupervised learning phase for potential down-selection.

1.4 Supervised learning sieve analysis (to detect sieve effects)

Once the AA sequence features and their levels are defined/pre-specified, sieve analysis is conducted, which yields point and confidence interval (CI) estimation of VE against each feature level, as well as hypothesis testing for whether VE differs across levels of a feature (i.e., a sieve effect). The sieve analyses also address a related question whose answers are not in terms of VE but can afford additional insights: Among COVID-19 endpoint cases, how do Spike AA sequence characteristics differ between the vaccine and placebo arms?

Note that AA sequence sieve analyses focus on Spike protein AA sequences; sieve analyses are not conducted to assess differential vaccine efficacy by non-Spike protein AA sequences, given that the vaccine-immune response only targets the insert (Spike) protein. Such sieve analyses could be of interest as as "control", but it is not clear that the added value is great enough to invest the effort for these analyses. It is not planned in this SAP.

1.5 Cohorts for AA sequence sieve analyses, and COVID-19 endpoints included in the analyses

Sieve analyses are of interest for conduct in the following two study cohorts.

- **(PPBN) Per-protocol baseline seronegative cohort:** The primary analysis cohort for evaluation of overall vaccine efficacy, as defined in the study protocol and Sadoff et al. (2021).
- **(FASBN) Full analysis set baseline seronegative cohort:** The full analysis set baseline seronegative cohort as defined in the study protocol and Sadoff et al. (2021).

The sieve analyses of the PPBN cohort count all COVID-19 endpoints starting 14 days post vaccination and through to a participant's unblinding date. The time origin of failure time analyses is the day of administration of vaccine/placebo, consistent with Figure 2 of Sadoff et al. (2021). Also consistent with this Figure 2, the at-risk population for analyses of the PPBN cohort excludes participants who were SARS-CoV-2 RT-PCR-positive between days 1 and 14 after the administration of vaccine or placebo. Therefore participants with the COVID-19 primary endpoint before 14 days post vaccine/placebo administration are excluded from the data analysis.

Note that for a COVID-19 endpoint case to be included in the analysis it is required that the Spike sequence for analysis was sampled on or after the failure event date (as defined in the study protocol).

Given that evidence suggests the vaccine had limited protection during the first 14 days post vaccination, only analyses of the PPBN cohort are conducted.

1.6 Filters/screens for sufficient variability of a mark feature for conducting sieve analysis

For the primary endpoint COVID-19, for each specified binary and nominal categorical mark, vaccine efficacy against each specific genotype (i.e., mark level) is assessed only for genotypes represented by at least 20 primary endpoint cases pooling over treatment arms. For example, for a given variant, variant-specific VE is only assessed for variants with at least 20 endpoints. This rule is applied separately for each of the four sieve analyses (i.e., for each of the three geographic regions and pooling over the three geographic regions).

For the geographic-region pooled analyses by methods that adjust for geographic region, there is an additional requirement for binary and nominal categorical marks that each mark level is represented by at least 5 endpoints within each of the three regions (added to the 'at-least 20' rule).

For quantitative marks (ordered categorical marks or a continuous score) with 3 or more levels, sieve analysis is only performed if, for a given mark, there are at least 20 primary endpoints with a mark value different from the most frequent value and at least 5 primary endpoints with a mark value different from the most and second most frequent value.

For the severe-critical COVID-19 endpoint, the filtering principle is the same, except the rule is changed to at least 10 endpoints instead of at least 20 endpoints. For quantitative marks with 3 or more levels, the rule for severe-critical COVID-19 is at least 10 endpoints with a mark value different from the most frequent value and at least 3 endpoints with a mark value different from the

most and second most frequent mark value. In addition, for the geographic-region pooled analyses by methods that adjust for each geographic region, the same additional requirement for binary and nominal categorical marks is made as for the severe-critical COVID-19 endpoint (i.e., 'for each mark value, at least 5 endpoints within each of the three regions').

1.7 Sequencing technology and samples obtained for sequencing (ENSEMBLE)

Nasal swab specimens from SARS-CoV-2 RT-PCR confirmed cases were used for sequence analysis. One sample per participant, taken as close as possible to the onset of symptoms, was selected if the SARS-CoV-2 viral load was approximately 1000 copies/mL or above to maximize the amplification/sequencing success rate. Selections were made regardless of the timing of onset of symptoms, severity of illness or any other parameter to ensure that sequencing was performed in an unbiased manner.

Next-generation sequencing was performed using the Swift Biosciences SNAP Version 2.0, performed at the Virology Laboratory of the University of Washington, Department of Laboratory Medicine and Pathology. The SNAP assay utilizes multiple overlapping amplicons in a single tube to prepare ready-to-sequence libraries. Primer pairs used in SNAP are designed for generating libraries from first- or second-strand cDNA produced from viral isolates or clinical specimens. This unique design enables successful SARS-CoV-2 library preparation from samples with low viral titers and provides powerful solutions for the confident detection of nucleotide variants. The Swift Biosciences SARS-CoV2 Version 2.0 kit (Catalog # CovG1 V2-96) has been optimized to achieve additional genome coverage. The assay is optimized for Illumina sequencing platforms. A full clinical validation with determination of analytical sensitivity and specificity, limit of detection, accuracy, and assay precision (reproducibility and repeatability) was done. The SARS-CoV-2 Wuhan-Hu1 variant including the D614G mutation (B.1 lineage) is referred to as the Reference Sequence. Amino acid substitution profiles were created to identify the different SARS-CoV-2 lineages and WHO variant labels using the SARS-CoV-2 Wuhan-Hu1 S gene sequence as a reference (NCBI $NC_0 45512.2$). The vaccine-insert strain/sequence is from the Wuhan-Hu-1 strain (prior to the D614G mutation) with two stabilizing mutations at K986P and V987P, and two additional mutations (R682S and R685G) in the furin cleavage site.

Methodology of the sequencing is described at

https://www.medrxiv.org/content/10.1101/2021.10.22.21265255v1

2 AA Sequence Sieve Analysis Methods: Approach A and Approach B

Sieve analysis questions are posed in two ways, first the assessment of whether and how VE depends on AA sequence features at the first SARS-CoV-2 PCR RNA+ time point connected to a COVID-19 primary endpoint (prospective VE sieve analysis, Gilbert et al., 1998) and second the assessment of whether and how these AA sequence features differ among the subsets of the randomized treatment arms who acquired the COVID-19 primary endpoint (case-conditional sieve analysis). The first kind is normative sieve analysis, given the direct interpretation in terms of vaccine efficacy, whereas the second kind can have improved statistical power and can provide additional insights, but does not

have an interpretation in terms of differential vaccine efficacy. We arbitrarily refer to the first kind and second kind as Approach B and Approach A sieve analysis, respectively, following the nomenclature used previously for neutralization sieve analysis (Corey et al., 2021). Because of the interpretation in terms of prospective vaccine efficacy, the Approach B sieve analysis results are prioritized.

For Approach B sieve analysis, we consider two Spike AA sequence feature-specific VE estimands for measuring sieve effects:

1. $VE_h(v)$ defined as one minus the hazard ratio (vaccine/placebo) of the primary study endpoint throughout the treatment-blinded follow-up period with a specific univariate AA sequence feature $V = v$ measured from the COVID-19 case's earliest SARS-CoV-2 RNA+ sample.

2. $VE_c(t, v)$ defined as one minus the ratio (vaccine/placebo) of cumulative incidences at time t of the primary study endpoint with a specific univariate AA sequence feature $V = v$ measured from the COVID-19 cases's earliest SARS-CoV-2 RNA+ sample. This parameter is estimated using a stratified estimator for all times t through a time point τ selected as the latest time point post first vaccination such that at least 250 still-blinded placebo recipients and at least 250 still-blinded vaccine recipients are at-risk for the COVID-19 primary endpoint in each stratum. For all analyses of $VE_c(t, v)$, participants who are under follow-up at τ and have not experienced the endpoint by τ have their failure time right-censored at τ; if they are lost to follow-up or unblinded earlier than τ, they are right-censored at the loss to follow-up or unblinding date, whichever occurred earlier. For proportional-hazards based analyses, failure times are right-censored at the earliest event of the loss to follow-up date or the date of unblinding.

An Approach B AA sequence sieve effect is defined as statistically significant evidence for differential VE across multiple levels of a given AA sequence feature. The primary estimand of interest will be $VE_h(v)$, and sieve effect evidence will be evaluated for every feature V, for both V discrete categorical and V quantitative (count variable or continuous-valued). Additionally, for binary features V such as haplotype match vs. mismatch to the vaccine strain, we will also evaluate AA sequence sieve effect evidence in parallel based on the estimand $VE_c(t, v)$ for t varying over all time points through time τ, where hypothesis testing for a sieve effect focuses on $t = \tau$.

We use published sieve analysis methods for analyzing various types of AA sequence marks V. For binary V (e.g., haplotype vaccine-match $V = 0$ vs. vaccine-mismatch $V = 1$), an extension of the Lunn and McNeil (1995) and Gilbert (2000) competing risks Cox model are used for assessing $VE_h(v)$ against each of the two genotypes (with 95% confidence intervals for each parameter $VE_h(0)$ and $VE_h(1)$, 2-sided p-values for $VE_h(v)$ departing from 0% for each $v = 0, 1$, and a 2-sided p-value for differential vaccine efficacy that tests $H_0 : VE_h(0) = VE_h(1)$). The extension uses augmented inverse probability weighting (AIPW) to account for the fact that some COVID-19 endpoint cases are missing a SARS-CoV-2 AA sequence, published as Hyun, Lee, and Sun (2012) and the pre-print Heng et al. (2022). The method is implemented in Fei Heng's R package *cmprskPH* that is set up to handle log10 viral load copies/ml at the illness visit as a key auxiliary covariate. For analyses of the pooled geographic regions, Heng et al. (2022) is implemented adjusting for indicators of two geographic regions in the mark-specific proportional hazards models.

For binary V, an extension of the cumulative incidence estimator and its influence function-based variance estimator in Benkeser et al. (2019) are used for estimating $VE_c(t, v)$ for all time points t starting 14 days post vaccination through time τ for each $v = 0, 1$ (with 95% pointwise confidence intervals in t for each $VE_c(t, v)$, $v = 0, 1$, 2-sided p-values for $VE_c(\tau, v)$ departing from 0% for each $v = 0, 1$, and a 2-sided p-value for a sieve effect that tests $H_0 : VE_c(\tau, 0) = VE_c(\tau, 1)$). The extension accommodates the missing SARS-CoV-2 AA sequences through efficient and doubly-robust TMLE, which includes use of inverse probability weighting.

For count variable $V = 0, 1, 2, ...$ and for quantitative V with at least 3 levels such as a variant-neutralization resistance score, the Juraska and Gilbert (2016) method is used (again with confidence intervals and a 2-sided p-value for $VE_h(v)$ varying in v), which accounts for the missing sequence data through AIPW (this method is implemented in the R package *sievePH* available on CRAN). For analyses of the pooled geographic regions, Juraska and Gilbert (2016) is implemented using separate marginal baseline hazard functions for each of the three geographic regions.

For ordered categorical or otherwise quantitative variable V with $K > 2$ discrete levels (e.g., weighted Hamming distances), an extension of the cumulative incidence estimator and its influence function-based variance estimator in Benkeser et al. (2020) are used for assessing $VE_c(\tau, v)$ for each level v of V (with 95% pointwise CIs in t at each v, and a 2-sided p-value for a linear trend in $\log\{1 - VE_c(\tau, v)\}$ with v, which tests for a sieve effect). The extension accommodates the missing SARS-CoV-2 AA sequences through efficient and doubly-robust TMLE, which leverages inverse probability weighting.

2.0.1 Overall cumulative-incidence based sieve analysis

For the ENSEMBLE overall analysis (pooling over the three geographic regions), the $VE_c(v)$ parameters are estimated adjusting for the U.S., Latin America, and South Africa geographic regions. For this overall analysis each sequence mark is required to pass the 'at least 20' rule for each of the three geographic regions; otherwise the mark is not assessed. Simulation studies suggested that this minimum information requirement corresponds to stable inferences.

2.0.2 Geographic region-specific sieve analyses

All unsupervised descriptive figures/tables are produced for each of the three geographic regions U.S., Latin America, and South Africa, and each supervised sieve analysis is conducted for each of the three geographic regions, with all procedures such as screening/filtering applied to each individual geographic region as if it were a stand-alone trial. This is done because the COVID-19 epidemic differed across the three geographic regions including in the distribution of circulating strains. It also affords an opportunity to study whether some sieve effect results are found to occur consistently across the geographic regions, which may support replicability/robustness of findings.

In addition, all analyses are done for the overall ENSEMBLE trial. Some of the geographic region-pooled analyses adjust for region as a covariate as described for each method.

The AIPW and TMLE methods include a "missingness model" for whether a COVID-19 endpoint case is missing the sequence data. The details of how this model is constructed, together with

additional implementation details for Approach B sieve analysis, are described in Sections 2.4 and 2.6.

2.1 Structuring AA sequence features for sieve analysis

As noted above, two AA sequence sieve analyses are conducted, one for **All features** and one for **nAb-CoP-hypothesis features**. Table 1 lists the set of AA sequence marks V studied in the **All features** sieve analyses (four sets). Table 2 lists the set of AA sequence marks V studied in the **nAb-CoP-hypothesis** sieve analyses. Following the tables, sub-sections provide additional details for the each of the sets. All of these AA sequence sieve analyses are pre-specified in a treatment-blinded manner. Section 2.10 describes the approach to adjustment for multiplicity of hypothesis tests.

Table 1: List of AA Sequence Features V for Sieve Analysis: **All Features**

1. All variants/lineages as defined by WHO[1]
2. All AA position features in Spike[2,3]
3. Physicochemical-weighted (Sandberg et al., 1998) Hamming distances to the vaccine strain (S, RBD, NTD, S1, S2)[4]

[1]Sufficiently variable = at least 20 COVID-19 cases with the variant (at least 10 for severe-critical COVID-19)

[2]Sufficiently variable = at least 20 COVID-19 cases with a non-modal level of the AA sequence feature (at least 10 for severe-critical COVID-19)

[3]Types of AA position features: Indicators of vaccine-mismatched residue (or gap or deletion); indicators of specific residues (or gap or deletion)

[4]Sufficiently variable = at least 20 COVID-19 cases with a mark value different from the most frequent mark value and at least 5 COVID-19 cases with a mark value different from the most and second most frequent mark value (for severe-critical COVID: at least 10 COVID-19 cases, at least 3 COVID-19 cases)

Table 2: List of AA Sequence Features V for Sieve Analysis: **nAb-CoP-hypothesis**

4. AA features based on Deep Mutational Scanning RBD antibody-escape scores impacting neutralization[1,2]
5. AA features based on Protein Data Bank Spike antibody-escape scores impacting neutralization from the PDB data base
6. NTD neutralization-relevant features (all features binary)[1]
8. Variant-neutralization sensitivity scores assigned to variants (Section 2.1.7)

[1]For binary features, sufficiently variable = at least 20 COVID-19 cases with a non-modal level of the AA sequence feature (at least 10 for severe-critical COVID-19)

[2]Binary features are included, as well as viral distance marks defined by RBD-antibody escape scores assigned to putative antibody footprint sets

2.1.1 All features: All variants as defined by WHO

For each specific analysis, sieve analysis is conducted for each lineage/variant with at least 20 primary endpoint cases (or at least 10 for the severe-critical COVID-19 endpoint).

The rationale for the at-least 20 criterion is as follows. If there are 250 vaccine and 750 placebo recipient COVID-19 cases, with 10 vaccine and 10 placebo recipients having a vaccine-mismatch, then the Fisher's exact test p-value is 0.017, which is small enough that a sieve effect can potentially (just barely) be detected after multiplicity adjustment. This scenario reflects a moderately strong sieve effect, with estimated VE of 75% against vaccine-matched COVID-19 and estimated VE 0% against vaccine-mismatched COVID. It is useful to detect such strong sieve effects. It would be possible to detect even stronger sieve effects with fewer primary endpoint cases with a sequence with a minority residue. Yet, these sieve effects would have a negative estimated VE against vaccine-mismatched COVID-19, and we seek to reduce risk of false positive inferences about negative VE that could only occur through such a 'qualitative interaction.' Another advantage of the at-least 20 rule is to assure satisfactory precision about genotype-specific VE. The 'at-least 20' filter rule is used for all analyses of the COVID-19 primary endpoint. Moreover, the 'at-least 10' filter rule is used for all analyses of the severe-critical COVID-19 primary endpoint; a more permissive filter is needed given the much smaller number of severe than non-severe-critical COVID-19 primary endpoints.

2.1.2 All features: All AA position features in Spike

For each specific analysis, an amino acid position-specific analysis on vaccine match vs. mismatch residue is conducted for all positions in the Spike protein that exhibit sufficient residue variability. In addition, at each amino acid position, a residue-specific analysis on whether a residue is present is conducted for all residues that exhibit sufficient variability. In these analyses we consider a gap or deletion as another 'residue' level to include as a feature for sieve analysis. Sufficient variability at a given position or for a specific residue at a given position is defined by at least 20 primary endpoint cases (pooling over treatment arms) having a sequence with a non-modal level of the feature (as stated in Section 1.6) (and an at-least 10 rule for severe-critical COVID-19).

For each qualifying AA position for vaccine match vs. mismatch analysis, we will make inferences about mark-specific vaccine efficacy for a binary position-specific sequence feature V defined as vaccine-matched residue ($v = 1$) vs. vaccine-mismatched residue ($v = 0$). In addition, for each qualifying specific residue at each given position, we will make inferences about mark-specific vaccine efficacy for a binary sequence feature V defined as specific residue being present ($v = 1$) vs. specific residue being absent ($v = 0$).

2.1.3 All features: Physicochemical-weighted (Sandberg et al., 1998) Hamming distances to the vaccine strain (S, RBD, NTD, S1, S2)

For each of the AA proteins/subproteins S, RBD, NTD, S1, S2, vaccine efficacy will be estimated across all levels of the PC-weighted Hamming distance. A protein/subprotein is deemed sufficiently variable for analysis if at least 20 COVID-19 cases have a distance value different from the most frequent value and at least 5 COVID-19 cases have a distance value different from the most and second most frequent value (for severe-critical COVID-19, at least 10 COVID-19 cases with a

distance value different from the most frequent value and at least 3 COVID-19 cases with a distance value different from the most and second most frequent value), as noted in the filtering section (Section 1.6).

2.1.4 bnAb CoP-hypothesis driven features: AA features based on Deep Mutational Scanning RBD antibody-escape scores impacting neutralization

Two regions of the spike protein are hypothesized to have the greatest potential for disruption of neutralization by vaccine-induced antibodies: 1) the receptor binding domain (RBD) and 2) the N-terminal domain (NTD). This subsection focuses on 1).

We define RBD features using an antibody-escape calculator for mutations to the SARS-CoV-2 RBD developed by Greaney et al. (biorxiv, https://www.biorxiv.org/content/10.1101/2021.12.04.471236v1) where mutations impact binding in deep mutational scanning experiments (Starr et al., 2020). For each sequence we calculate an antibody-escape score relative to the Wuhan ancestral strain, based on the RBD sequence. The score, defined as one minus the binding retained (see Greaney et al.), is a distance from 0 to 1 where 0 is no escape and 1 is full escape. This distance is analyzed as its quantitative score using the distance-based sieve analysis approach. In particular, the same approach described in Section 2.1.3 is applied to analyze these quantitative viral marks as is used for analysis of the PC-weighted Hamming distances.

In addition to the RBD-escape score mark described above, marks are defined for specific epitope classes. Based on the antibody-escape calculator, the antibodies used to define the calculator are clustered into ten epitope-specific clusters. We generated between 2 and 21 non-singleton clusters based on hierarchical clustering using Euclidean distances and complete linkage then computed the number of sites for each cluster by counting the number of AA positions with an average escape of greater than 0.05 (Table 3). Based on a desire to have approximately 15 AA positions per cluster (in order to represent a putative epitope footprint specificity) and a relatively small number of clusters, we select K=8 non-singleton clusters. Singleton clusters (clusters 4 and 10) will be excluded from the analysis. For each cluster we define an epitope-specific antibody-escape score mark across all sites in RBD using the set of selected antibodies in each cluster. Each of these marks is analyzed using the same approach described in Section 2.1.3 for PC-weighted Hamming distances.

In addition, the set of amino acid positions that have high RBD binding escape scores are flagged as nAb-important positions for AA position scanning sieve analysis. Among the RBD sites listed in Table 3, we select the sites most important for neutralization. These sites are selected by restricting to sites in the set of eight non-singleton clusters that satisfy one of the following two conditions: 1) the site is in 3 or more putative antibody footprints (clusters) or 2) the maximum within-cluster mean escape-score is at least 0.4. This results in 20 important RBD sites for neutralization listed in Table 4.

2.1.5 bnAb CoP-hypothesis driven features: AA features based on Spike antibody-escape scores impacting neutralization from the Protein Data Bank

In addition, a second approach is used to define putative antibody footprint site sets based on the Protein Data Bank (PDB), which includes all of Spike such that positions outside of RBD may be included.

Table 3: RBD-antibody binding escape score based putative epitope footprints. For each cluster (footprint) this table gives the number of antibodies per cluster (n), the number of sites per cluster (n sites), and the cluster site list. Singleton clusters 4 and 10 will be excluded from the analysis.

cluster	n	n sites	sites
1	39	14	417,420,421,453,455,456,460,463,472,473,475,476,487,489
2	56	12	346,348,352,417,420,455,456,468,473,475,493,504
3	33	10	456,472,473,475,476,484,485,486,487,489
4	1	9	472,478,479,481,482,483,484,485,486
5	59	11	449,452,456,472,483,484,485,486,490,493,494
6	26	13	346,443,444,445,446,447,448,449,450,452,490,494,499
7	7	14	443,445,446,447,449,455,456,484,490,494,496,498,500,501
8	3	15	356,357,444,445,446,447,448,449,450,452,490,494,496,519,525
9	18	18	335,361,369,370,372,374,375,376,378,383,384,385,408,413,417,427,441,503
10	1	13	349,356,374,377,378,381,394,410,435,466,468,507,525

Table 4: RBD-antibody binding escape score based putative epitope footprints: important sites for neutralization

Site	Max. escape	Number of clusters
486	0.99	2
484	0.98	3
447	0.93	3
446	0.88	3
496	0.74	2
475	0.72	3
444	0.69	2
456	0.66	5
449	0.61	4
378	0.52	1
490	0.50	4
487	0.48	2
473	0.46	3
498	0.45	1
417	0.42	3
494	0.38	4
445	0.38	3
455	0.34	3
452	0.33	3
472	0.16	3

For each primary COVID-19 endpoint their virus is assigned a quantitative mark based on an epitope or class of epitopes. Unique SARS-CoV-2 Spike and human antibody complexes were downloaded from the PDB database (n=274 on May 4, 2022). For each PDB complex, epitope

sites are defined as antigen sites that are in contact with the antibody in the antigen-antibody complex (i.e. all sites that have non-hydrogen atoms within 4 Angstrom of the antibody).

The interaction between an epitope site i and the antibody is defined as the weight w_i:

$$w_i = 1/2(n_{c_i}/\langle n_c \rangle + n_{b_i}/\langle n_{nb} \rangle) \quad (1)$$

in which, n_{c_i} is the number of contacts with the antibody (i.e. the number of non-hydrogen antibody atoms within 4 Angstrom of the site); n_{b_i} is the number of neighboring antibody residues; $\langle n_c \rangle$ is the mean number of contacts n_c and $\langle n_{nb} \rangle$ is the mean number of neighboring antibody residues n_{nb} across all epitope sites. A weight of 1.0 is attributed to the average interaction across all epitope sites. Neighboring residue pairs were identified by Delaunay tetrahedralization of side-chain centers of residues (Ca is counted as a side chain atom, pairs further than 8.5 Angstrom were excluded).

Antibody epitope footprints were compared for all pairs of antibodies. The similarity between antibodies was defined as:

$$sim = \Sigma_i(min(wa_i, wb_i))/nsim = \Sigma_i(min(wa_i, wb_i))/n \quad (2)$$

in which, wa_i and wb_i are the interaction weight of antibody a and b at site i, and n is the number of unique contact sites (for antibodies a and b combined). The similarity ranges between 0 (no overlap of the binding sites) and 1 (identical binding sites). The similarity is transformed into a distance (1 - similarity). Hierarchical clustering of all ENSEMBLE primary COVID-19 endpoints with sequence data is used to define clusters based on the distance matrix with a distance cutoff of 0.8. Fourteen clusters were identified (Figure 4). Each cluster included a minimum of three antibodies, these antibodies mapped to the RBD ($n = 12$) and the NTD ($n = 2$). A representative antibody is identified for each cluster.

For each cluster, the epitope distance between a virus sequence × and a reference sequence R (corresponding to the vaccine insert) was defined as the weighted mean of the distance between all epitope sites:

$$\begin{aligned} D(R, X) &= \sum_i [w_i * Dist(X_i, R_i)] / \sum_i w_i \\ Dist(X_i, R_i) &= 1/2 * [Sim(R_i, R_i) + Sim(X_i, X_i)] - Sim(X_i, R_i) \end{aligned}$$

in which, $Dist(X_i, R_i)$ is the sequence distance between epitope site i; $Sim(X_i, R_i)$ is the amino acid similarity according to the BLOSUM62 matrix. The distance between amino acid pairs includes insertion/deletion and glycosylation (match, 0; mismatch, -13, the worst substitution).

Individual epitope distances are compiled to obtain summary measures for the 14 Spike clusters, yielding 14 quantitative viral marks for sieve analysis.

For each antibody footprint set, the sieve analysis assesses vaccine efficacy by the quantitative mark variable computed in each of the two ways described above (deep mutational scanning RBD-escape calculator and PDB data base).

Table 5: Epitope footprints for antibodies representative of 14 clusters. 274 Spike antibodies were grouped in 14 clusters based on the similarity of their epitope footprint. Each cluster includes at least 3 antibodies. The epitope footprint and the weight of the interaction are provided for each epitope site.

cluster	location	n_{Abs}	Rep. Ab	Rep. PDB complex	n sites
1	RBD	61	P22A-1D1	7chs (E:HL)	36
2	RBD	39	S2E12	7r6x (R:CD)	19
3	RBD	12	Beta-40	7ps7 (R:HL)	16
4	RBD	18	A19-61.1	7tbf (A:DE)	20
5	RBD	12	BD-812	7ezv (A:HL)	18
6	RBD	9	A5-10	7f7e (E:CL)	18
7	RBD	10	35B5	7e9p (B:HL)	29
8	RBD	44	MW01	7djz (C:AB)	29
9	RBD	11	C022	7rku (A:GI)	29
10	RBD	15	3D11	7m7b (A:HL)	20
11	RBD	4	FD20	7cyv (B:H)	20
12	RBD	4	47D11	7akd (B:HL)	23
13	NTD	16	2-51	7l2c (B:CD)	17
14	NTD	4	2490	7dzy (B:HL)	26

2.1.6 bnAb CoP-hypothesis driven features: NTD neutralization-relevant features (all features binary)[1]

The same approach described in Section 2.1.2 for analyzing match vs. mismatch AA positions is applied, given that all NTD neutralization-relevant features are dichotomous. In particular, the NTD supersite epitope defined by McCallum et al. (2021) encompasses residues 14-20, 140-158, and 245-264. In addition, we anticipate that mutations in the NTD signal peptide potentially impact where the signal peptide is cleaved off (e.g., S12P or S13I). As additional mutations and neutralizing antibodies have emerged, we expand the hot spot to include positions 12–13, 14–26, 138–158, and 242–264. D80 mutations also appear and have a mild impact on neutralizing antibody binding. Deletions at positions 69 and 70, mutation T95I, and R190 mutations could also be involved in neutralizing antibody evasion based on their nearby locations (Matthew McCallum, personal communication Dec. 10, 2021). Based on these observations we define NTD features to include as potentially important for neutralization as either very important or moderately important. These features are listed below:

Very important

- One or more deletions or insertions in positions 138–158 and 242–264
- Signal peptide cleavage delay caused by mutations at positions 12 or 13. Note, these mutations are typically accompanied by disulphide bond rearranging mutations (C15X or C136X)
- One or more of the following mutations, R246X, G142X, K147X, or L18P

Table 6: Epitope footprints for antibodies representative of 14 clusters. 274 Spike antibodies were grouped in 14 clusters based on the similarity of their epitope footprint. Each cluster includes at least 3 antibodies. The epitope footprint and the weight of the interaction are provided for each epitope site (continued from Table 5).

cluster	(site:weight)
1	403:1.04,405:0.16,406:0.16,409:0.16,415:1.21,416:0.92,417:2.10,420:0.91,421:1.79,453:0.62
1	455:1.79,456:1.24,457:0.29,458:1.66,459:0.32,460:0.91,473:1.34,474:0.32,475:2.24,476:1.08
1	477:0.46,486:1.69,487:1.81,489:1.50,493:1.93,494:0.16,495:0.30,496:0.76,497:0.16,498:0.78
1	500:0.78,501:1.36,502:1.04,503:0.30,504:0.16,505:2.57
2	417:0.41,455:1.23,456:0.55,458:0.14,473:0.79,475:1.50,476:1.12,477:1.12,478:1.12,479:0.14
2	480:0.14,483:0.14,484:0.60,485:1.45,486:3.38,487:1.67,488:0.33,489:1.86,493:1.31
3	403:0.14,444:0.14,445:2.76,446:1.46,447:0.14,449:1.31,493:0.73,494:0.14,496:0.44,498:1.16
3	499:1.16,500:2.91,501:1.89,502:0.58,504:0.14,505:0.87
4	345:0.50,346:1.59,440:0.43,441:0.72,442:0.17,443:0.60,444:2.96,445:2.52,446:1.10,447:0.72
4	448:1.15,449:3.34,450:0.83,452:0.33,490:0.33,492:0.38,493:0.72,494:1.10,496:0.33,499:0.17
5	345:0.61,346:2.04,439:0.61,440:1.78,441:1.05,442:0.33,443:0.33,444:2.37,445:2.43,446:0.63
5	447:0.77,448:0.33,449:0.33,450:0.47,498:0.61,499:0.91,500:2.25,502:0.16
6	343:0.93,345:1.97,346:0.53,439:0.92,440:2.11,441:2.24,442:0.92,443:1.45,444:0.94,445:1.47
6	446:0.14,448:0.67,450:0.27,451:0.39,498:0.14,499:1.33,500:1.18,509:0.39
7	339:0.18,340:1.71,341:0.37,344:0.84,345:1.79,346:4.37,347:0.66,348:1.89,349:0.55,351:0.95
7	352:0.66,354:1.31,356:0.95,399:0.18,449:2.18,450:1.97,451:0.37,452:0.18,466:1.31,468:1.42
7	469:0.37,470:1.89,472:0.18,481:0.29,482:0.37,483:0.37,484:0.37,490:1.13,492:0.18
8	351:0.36,403:0.19,417:0.72,444:0.38,445:0.38,446:0.73,447:0.19,449:2.52,450:0.54,452:1.46
8	453:0.72,455:0.74,456:0.36,470:0.55,472:0.19,475:0.19,481:0.19,482:0.38,483:1.28,484:3.27
8	485:2.20,486:3.06,487:0.91,488:0.19,489:1.63,490:2.01,492:0.74,493:1.81,494:1.09
9	369:1.03,370:0.17,371:0.52,372:0.17,374:0.17,377:1.03,378:1.72,379:1.72,380:1.38,381:1.22
9	382:0.69,383:1.55,384:1.04,385:0.52,405:0.17,408:2.41,409:0.17,411:0.35,412:1.04,413:1.91
9	414:1.74,415:1.39,416:0.52,417:0.17,427:2.76,428:1.72,429:1.03,430:0.52,460:0.17
10	366:0.17,369:1.18,370:1.89,371:0.68,372:1.53,374:0.68,375:1.53,376:1.20,377:1.20,378:2.22
10	379:0.85,380:0.17,381:0.17,383:1.53,384:1.36,385:1.71,407:0.17,408:1.21,411:0.17,414:0.34
11	353:0.39,354:0.81,355:2.40,356:0.39,357:0.98,396:0.39,426:0.19,427:0.39,428:1.19,459:0.77
11	461:0.40,462:3.40,463:1.38,464:0.20,465:2.79,466:2.61,468:0.19,516:0.39,518:0.39,519:0.39
12	332:0.83,333:0.18,334:0.91,335:2.44,336:1.52,337:1.89,338:1.48,339:3.01,340:1.89,342:0.69
12	343:2.69,362:0.18,363:0.36,364:1.20,365:0.18,367:0.55,368:0.83,370:0.18,371:0.18,373:0.18
12	374:0.18,527:0.36,529:1.06
13	144:0.64,145:1.33,146:0.39,147:2.36,148:0.66,150:1.01,152:0.60,246:1.45,247:0.34,248:2.11
13	249:1.22,250:1.56,251:2.02,252:0.66,253:0.39,254:0.14,257:0.14
14	66:0.19,68:0.19,69:0.39,70:0.78,71:0.39,72:0.78,73:0.19,75:0.19,98:0.19,180:0.19,181:4.72
14	182:4.92,183:0.97,184:1.36,185:0.97,186:0.19,211:4.53,212:0.39,213:1.16,214:1.16,215:1.16
14	218:0.19,261:0.19,262:0.19,263:0.19,264:0.19

Moderately important

- One or more substitutions in 138–158 excluding positions 142 and 147
- One or more substitutions in 14–26 and 242–264 excluding substitutions R246X and L18P
- One or more substitutions D80X or L18F
- One or more substitutions that add or remove a glycan sequon within the NTD

For sieve analysis, we include all of the above features, regardless of whether they are deemed Very important or Moderately important. All features are evaluated as binary marks, using the binary mark sieve analysis method approach.

2.1.7 bnAb CoP-hypothesis driven features: Variant-neutralization sensitivity score assigned to variants

The same approach described in Section 2.1.3 for PC-weighted Hamming distances is applied.

For each variant that caused a COVID-19 primary endpoint, a variant-neutralization resistance score is assigned to the variant/lineage defined based on clinical trials that studied 50% neutralization titers of Day 71 vaccinee sera to a panel of variants. For each variant, the score is defined as log10 fold-change of the geometric mean ID50 titer to the variant vs. to the Ancestral strain for per-protocol vaccine recipients in a phase one trial of the Ad26.COV2S vaccine.

Janssen (Mathieu Le Gars) collated data assessing the cross-neutralization of several SARS-CoV-2 variants in COV3001/ENSEMBLE vaccine recipient Day 71 serum samples:

- COV3001/ENSEMBLE Day 71 samples were analyzed in a Janssen-internal Wuhan (D614G) psVNA for the reference strain.
- 8 samples were selected based on high D614G psVNA titers.
- These samples were then run in internal psVNAs for different variants including Alpha, Beta, Delta, Gamma and Zeta.
- Results of this assessment are shown in Table 7.
- The table reports GMTs and fold-change in GMT compared to the Reference virus B.1 for each variant.
- The results are consistent with the Monogram psVNA in terms of fold change compared to the D614G psVNA from Monogram for the Delta and Beta variants (the only variants assessed in both assays).
- The log10 fold-change (based on row 2 of the table) is the value of the mark V assigned to each of the variants listed in Table 7.
- For variants/lineages not represented in the table, some variants are known to have similar neutralization resistance as certain other variants, which we use to make the following assignments: Epsilon is assigned the same score as Delta; Iota is assigned the same score as Alpha; Lambda is assigned the same score as Delta; Mu is assigned the same score as Beta.

Table 7: Neutralization activity of serum antibodies elicited by Ad26.COV2.S against SARS-CoV-2 spike pseudotyped virus variants. Serum samples (N=8) from participants in the phase 3 ENSEMBLE trial immunized with one dose of 5 $\times 10^{10}$ viral particles of Ad26.COV2.S were obtained on day 71 past immunization. Samples were tested in a pseudotyped lentivirus neutralization assay on SARS-CoV-2 spike protein variants of concern, measuring neutralizing titers with the pseudovirus neutralization assay at a 50% inhibitory concentration (IC, serum dilution). The table reports, for each virus variant, the geometric mean titer (GMT) and the fold changes compared to B.1. The P-value tests whether the GM Fold-Change differs between the variant and the Reference virus B.1.

	Reference (B.1)	Alpha (B.1.1.7)	Beta (B.1.351)	Delta (B.1.617)	Delta (B.1.617.2)	Gamma (P.1)	Zeta (P.2)
GMT	246	266	68	162	154	72	115
Fold-Ch Over B.1	N/A	0.9	3.6	1.5	1.6	3.4	2.2
95% LCL Fold-Ch	N/A	0.8	2.1	1.0	1.2	2.1	1.2
95% UCL Fold-Ch	N/A	1.0	6.2	2.4	2.2	5.7	3.8
P-value	N/A	0.118	0.001	0.060	0.007	0.001	0.016

- Table 7 lists mark values for both Delta (B.1.617) and the Delta sub-lineage B.1.617.2. Because our actual variant calls only identify sequences generally as Delta (they do not characterize which sequences are of the B.1.617.2 lineage), we are using the mean of the readouts for these two Delta variants (i.e., fold change = 1.55) for all Delta sequences. This also applies to the Epsilon and Lambda sequences which are using Delta as an equivalent (as described above).
- Other variants with no score assignment are treated as having a missing mark value in the sieve analysis.
- The correlation of this variant-neutralization resistance score with the RBD antibody-escape calculator score will be evaluated.
- The variant-neutralization resistance score will be analyzed in the same way as the RBD antibody-escape calculator quantitative viral mark.

2.2 Listing of application of AA sequence sieve analyses methods for the All feature sequence sets and the nAb-CoP-hypothesis feature sequence sets

Table 8 lists the methods that will be applied to the **All Features** listed in Table 1. Table 9 lists the methods that will be applied to the **nAb-CoP-hypothesis** features listed in Table 2.

Table 8: Sieve analysis methods applied to the **All AA sequence features**

Mark/Analysis (Marks V defined in Table 1)
Marks 1 (Variants) (nominal categorical)
Approach B $VE_h(v)$, $VE_c(t,v)$
Marks 2 (AA position features) (binary)
Approach B $VE_h(v)$, $VE_c(t,v)$
Marks 3 (PC-weighted Hamming distances) (ordinal/quantitative variable)
Approach A DRTMLE
Approach B $VE_h(v)$, $VE_c(t,v)$

Table 9: Sieve analysis methods applied to the **nAb-CoP-hypothesis** AA sequence features

Mark/Analysis (Marks V defined in Table 2)
Marks 4 (AA position features from RBD-Ab escape scores[1]) (binary)
Approach B $VE_h(v)$, $VE_c(t,v)$
Marks 4 (AA set Deep Mutational Scanning RBD-Ab viral mark scores/distances[1]) (quant. variable)
Approach A DRTMLE
Approach B $VE_h(v)$, $VE_c(t,v)$
Marks 5 (AA set Spike-Ab viral mark scores PDB data base[1]) (quant. variable)
Approach A DRTMLE
Approach B $VE_h(v)$, $VE_c(t,v)$
Marks 6 (NTD neutralization-relevant features[1]) (binary)
Approach B $VE_h(v)$, $VE_c(t,v)$
Marks 7 (Variant-neutralization sensitivity score assigned to variant)
Approach B $VE_h(v)$, $VE_c(t,v)$

[1]Defined as important positions for antibody escape: see Sections 2.1.4, 2.1.5, and 2.1.6.

Sieve analysis of variants/lineages will include estimation and hypothesis testing for differential VE. Differential VE (DVE) for lineage 1 vs. lineage 2, with the point estimate of VE(lineage 1) $\geq$ the point estimate of VE(lineage 2), is calculated as

$$\text{DVE} = [1 - \text{VE(lineage 2)}]/[1 - \text{VE(lineage 1)}],$$

with interpretation that vaccine protection is DVE-fold better against lineage 1 than against lineage 2. DVE is estimated to compare all pairs of lineages with enough cases for evaluation, as well as for each lineage vs. not that lineage. Corresponding to the point and 95% CI estimates for DVE is a p-value testing whether DVE departs from unity.

2.3 Graphical descriptive analyses of AA sequence features

Graphical descriptive analyses are done for all of the AA sequence features, which can be classified into the following types:

- WHO Variant labels (nominal categorical marks)
- AA position features (binary marks) [types of features: Indicators of vaccine-mismatched residue (or gap or deletion), indicators of specific residues
- Discontiguous site set haplotypes (quantitative score marks)
- PC-weighted Hamming distances (quantitative marks)
- Deep Mutational Scanning RBD antibody-escape distances (quantitative marks)
- Spike antibody-escape distances from PDB data base (quantitative marks)
- NTD features (binary marks)

Plots of COVID-19 cases with sequence data will compare marks between the PPBN vaccine arm vs. the PPBN placebo arm, stratified by geographic region.

For variants, plots will describe how the distributions of variants change over calendar time, stratified by geographic region.

For AA sequence position features, we will plot across all Spike AA positions the number of sequences with a vaccine-mismatched residue or gap/deletion at a position. These plots determine the set of AA positions with sufficient variability for sieve analysis (≥ 20 endpoints with a non-modal residue (or gap), or ≥ 10 for severe-critical COVID-19). In addition, heatmaps are used to describe which AA positions are included for match/mismatch sieve analysis. Heatmaps are also used to describe which specific residue indicators at AA positions are included in residue sieve analysis. For all positions with sufficient variability, a logo plot will be used to describe the residue distribution.

For AA set haplotypes we will plot across all sets spanning Spike AA the number of sequences with a vaccine-mismatched haplotype. This plot determines the set of haplotype positions with sufficient variability for sieve analysis (≥ 20 endpoints with a non-modal haplotype, or ≥ 10 for severe-critical COVID-19). For all haplotype positions with sufficient variability, a barplot will be used to describe the haplotype distribution.

For quantitative marks such as PC-weighted Hamming distances and RBD antibody-escape values, violin plots and reverse cumulative distribution function plots will be used.

Phylogenetic trees are estimated to display viral variation over calendar time and for each of the three geographic regions, focusing on the calendar periods of follow-up in the blinded phase for each of the three geographic regions. Phylogenetic trees will be constructed using all ENSEMBLE nucleotide sequences and the vaccine status will be visualized in the trees. Trees will be analyzed to identify all lineages and to describe (without formal inferences) if sequences from vaccine recipients appear to be over-represented in specific clusters.

2.3.1 Example descriptive plots for expected mark distributions

To start to understand the mark features, descriptive plots are provided in Figures 2 and 3), based on Spike protein sequences randomly sampled from GISAID, where the sampled data set consists of 1122 sequences from individuals in the United States and deposited into GISAID between September 8 2020 and February 1 2021.

2.4 Approach to handling missing sequences/marks

For primary endpoint COVID-19 cases, either all mark features (based on a Spike sequence) are observed, or none of the mark features are observed. Thus the structure of the missing data pattern for primary endpoint cases is simple, with complete mark data or no mark data. For each specific sieve analysis method, the section dedicated to the method describes how the method handles the missing mark data.

As also noted elsewhere in this SAP, viral load is a key auxiliary variable that is used in many of the sieve analyses. Among primary endpoint COVID-19 cases, only those that have a viral load value are included in the analysis. Only a single viral load auxiliary is used for each primary endpoint case, and zero or one Spike sequence is used for each primary endpoint case.

Some samples are not sent for sequencing because the viral load is low, with 1000 copies/ml used as a guide for low viral load. Despite the guidance, several samples with viral load < 1000 copies/ml were sequenced in each combination of geographic region and treatment group. The data base includes information on which samples were sent for sequencing. We explored whether this information improves prediction of sequence availability. To this end, we assessed cross-validated AUCs (CV-AUCs) of logistic regression models with the indicator of an available sequence as the binary outcome and the following sets of predictors:

1. $I(VL \geq 1000)$ for the US and South Africa models, $I(VL \geq 1000)$, indicators of geographic regions (Colombia, Latin America excluding Colombia for Latin America, US, South Africa, Colombia, Latin America excluding Colombia for region pooled), and two-way interactions between $I(VL \geq 1000)$ and the indicators of geographic regions for the Latin America and region-pooled models,

2. $\log_{10} VL$ for the US and South Africa models, $\log_{10} VL$, indicators of geographic regions, two-way interactions between $\log_{10} VL$ and the indicators of geographic regions for the Latin America and region-pooled models,

3. $I(VL \geq 1000)$ and $I(VL \geq 1000) \times \log_{10} VL$ for the US and South Africa models, $I(VL \geq 1000)$, $I(VL \geq 1000) \times \log_{10} VL$, indicators of geographic regions, two-way interactions between $I(VL \geq 1000)$ and the indicators of geographic regions, and two-way interactions between $I(VL \geq 1000) \times \log_{10} VL$ and the indicators of geographic regions for the Latin America and region-pooled model.

The rationale for using $I(VL \geq 1000)$ as a predictor in models 1. and 3. is to incorporate the guidance that samples with viral load < 1000 copies/ml would not be sent for sequencing. We found that logistic models described in 2. maximized the CV-AUC, and the inclusion of $I(VL \geq 1000)$ as a predictor did not improve the prediction of observing a sequence. We also added randomized treatment arm to the model, and it did not improve the prediction of observing a sequence.

In addition, we used the superlearner with prediction algorithms SL.mean, SL.glm, SL.step, SL.gam, SL. bayesglm, SL. glmnet, SL.earth, SL.ranger, SL. xgboost for each of the three sets of predictors described in 1.–3. above. Logistic regression (SL.glm) was consistently rated among the top four estimators according to CV-AUC, with virtually no loss in prediction accuracy compared with the superlearner estimator. As a result, logistic models in 2. will be used for estimating the probability

of observing a sequence in all employed AIPW methods.

2.4.1 Defining the viral load auxiliary variable accounting for multiple viral load measurements

The viral load variable is available at the same time point as the Spike sequence/mark from more than 92% of primary endpoint cases. Based on this high rate, only primary endpoint cases with viral load measured are included in the data analyses. For the participants that have a Spike sequence but not a viral load value at the sequence sampling time point, the following approach is used to define the viral load auxiliary: Take the viral load after the sequence sampling time point and closest to the time point, and if the number of days post sequence sampling exceeds 5 days, set the viral load auxiliary to NA. As noted when the primary cohort PPBN is introduced, the sequencing sampling time point must occur on or after the date of the primary endpoint; cases with the sequence sampled before the primary endpoint date are excluded from the analysis.

2.5 Details of the Approach A conditional sieve analysis method

The Approach A method compares the marginalized mean value of the AA sequence feature V between the vaccine group viruses and the placebo group viruses. This approach will be applied using doubly robust targeted minimum loss-based estimation (DRTMLE) for a quantitative readout (Benkeser et al., 2019), to estimate the mean readout/mark of each group with a Wald 95% confidence interval, as well as the mean difference (vaccine - placebo) with a Wald 95% confidence interval. P-values are not presented because inferences for sieve analysis are based on the prospective Approach B sieve analysis.

The DRTMLE method will accommodate the missing data on viral sequences by including a model of the probability of observing the mark, where the model will be simpler if there is less missing data. Given the results of the modeling reported above in Section 2.4, DRTMLE will be implemented with the same logistic regression model as used for the other sieve analyses.

To explain what question the Approach A DRTMLE analysis is addressing, given the goal of studying post-infection vaccine pressure on the virus, the underlying causal parameter of interest is the survivor average causal effect (SACE) (e.g., (Gilbert et al., 2003)), defined as

$$\text{SACE} = E\left[V(1)|Y(1) = Y(0) = 1\right] - E\left[V(0)|Y(1) = Y(0) = 1\right]$$

where V is a viral mark being analyzed, Y is the indicator of experiencing the COVID-19 primary endpoint, and (1) denotes potential outcomes if assigned vaccine whereas (0) denotes potential outcomes if assigned placebo. Under an assumption that all baseline covariates W predictive of both V and Y are included in regression analysis, the SACE equals $E_W E[V|Y = 1, A = 1, W] - E_W E[V|Y = 1, A = 0, W]$, where each of the two marginalized means are estimated by DRTMLE. To handle missing sequence data, if we let Δ be the indicator that a primary endpoint case has the viral mark V observed, then under a missing at random assumption one actually estimates the statistical parameter $E_W E[V|Y = 1, A = a, \Delta = 1, W]$ for each $a = 0, 1$.

Model for predicting the sequence feature

In addition, all implementations of the DRTMLE method adjust for baseline covariates that are judged to be possibly associated with both the COVID-19 endpoint and with the mark feature under analysis. The covariate adjustment is needed because the analysis must control for all dual predictors of the COVID-19 endpoint and the mark outcome V in order that the estimated contrast in distributions/means across treatment arms assesses a causal effect in the sub-population of participants who would be a COVID-19 endpoint case under either treatment assignment (vaccine or placebo) (e.g., Shepherd et al., 2006). The modeling is designed to guard against including adjustment for a covariate that is associated with occurrence of COVID-19 but not with the mark feature. It is desirable to only adjust for baseline covariates associated with the AA sequence feature because if the covariate is associated with COVID-19 risk but not the AA sequence feature, then its inclusion will have no benefit and will tend to reduce precision. Therefore, a superlearner model will be used that is designed to only select covariates predictive of the given sequence feature under study. The model will include covariates that are putatively correlated with phylogenetic clusters of the epidemic, including geographic region and calendar time of enrollment. Specifically, we will use as predictors the indicators of each biweekly calendar period of the enrollment date, and, for the geographic region-pooled analysis, also indicators representing the geographic regions (US, South Africa, Colombia, Latin America excluding Colombia). Also, the Latin America analysis will include the indicator of Colombia. The super learner library includes algorithms designed specifically to detect interactions in these covariates (e.g., as a result of a variant emerging at one site, but not others).

The covariate adjustment is achieved using a superlearner model for the conditional outcome regression (where the mark V is the outcome). If V is quantitative, this model is fit using the non-negative least squares (NNLS) loss function (the default for the SuperLearner R package with a quantitative outcome), and including the following learners for predicting a quantitative outcome (as hard-coded into the Superlearner R package): SL.ranger (default tuning parameters), SL.xgboost (tree depth = 2, 4, 6), SL.mean, SL.glmnet (default tuning parameters), SL.glm, SL.earth. All of these learners are documented in the SuperLearner R package.

If V is binary, then a superlearner model with minus the binomial log likelihood-loss is used to estimate the probability of $V = 1$. The library of learners is the same as for quantitative V, except using a logistic link for each learner. If fewer than 50 COVID-19 cases have each class level $V = 0$ and $V = 1$, then the library of learners is simplified by removing SL.xgboost and SL.glmnet.

Details on R code implementing the DRTMLE Approach A sieve analysis. The analysis will be done using the *drtmle* R package by Dr. David Benkeser available at CRAN. Logistic regression is used to estimate the propensity score for treatment assignment to vaccine vs. placebo, using the same modeling approach for whether a primary endpoint case has a sequence is observed. The reduced dimension regressions will use super learner libraries SL.glm and SL.gam. The analysis will use partial-cross-validated standard error estimates for constructing confidence intervals and hypothesis tests, which are described in the package documentation.

The implementation of the analysis reports the following results for a quantitative mark V (e.g., Hamming distance):

1. Report point and 95% confidence interval estimates of the mean of V for each of the two groups: PPBN vaccine COVID-19 cases and PPBN placebo COVID-19 cases.

2. Report point and 95% CI estimates of the mean difference of V (vaccine - placebo) with a Wald 95% confidence interval.

The implementation of the analysis reports the following results for a binary mark V (the first two items are the same as for quantitative V):

1. Report point and 95% confidence interval estimates of the mean of V (probability that $V = 1$) for each of the two groups: PPBN vaccine COVID-19 cases and PPBN placebo COVID-19 cases.

2. Report point and 95% CI estimates of the difference of means of V (differences in probability that $V = 1$) (vaccine - placebo) with a Wald 95% confidence interval.

3. Report point and 95% CI estimates of the ratio of means of V (ratio of probabilities that $V = 1$) (vaccine / placebo) with a Wald 95% confidence interval.

In addition, a sensitivity analysis is done reporting results using inverse probability weighting methodology instead of DRTMLE methodology, with the former method implemented with simple weighted linear regression with the *glm* R package.

2.6 Details of the Approach B hazard-based VE sieve analysis methods

We describe implementation details of the semiparametric proportional hazards estimation and hypothesis testing methods. For the geographic region-pooled analysis of mark V that is a count or quantitative variable, the Cox model portion of the mark-specific hazard-ratio model in Juraska and Gilbert (2016) will be stratified by geographic region, while the mark density ratio model will remain unstratified. For marks that are count or quantitative variables, the following Juraska and Gilbert (2016) hypothesis tests will be employed: First, the one-sided weighted Wald test of $\{H_{10} : VE(v) = 0 \text{ for all } v\}$ is applied, where the test was designed to increase power to detect alternatives where both the marginal $VE > 0$ and $VE(v)$ decreases with v. Second, the one-sided Wald test of $\{H_{20} : VE(v) = VE \text{ for all } v\}$ against $\{H_{21} : VE(v) \text{ decreases with } v\}$ is applied. The Wald tests of H_{10} and H_{20} above are described in Juraska and Gilbert (2016, Section 5).

For both AIPW hazard-based methods, Juraska and Gilbert (2016) and Heng et al. (2022), the logistic regression model for predicting the probability of observing a mark will use as predictors log10 viral load, and, for the geographic region-pooled analysis, additionally indicators of the geographic regions (US, South Africa, Colombia, Latin America excluding Colombia) together with all two-way interactions of region indicators and log10 viral load. The US and South Africa analyses will include log10 viral load only, and the Latin America analysis will include the indicator of Colombia, log10 viral load, and their interaction.

For predicting the mean mark in the augmentation term, we will use logistic regression for binary marks in the application of Heng et al. (2002) and linear regression for quantitative marks in the application of Juraska and Gilbert (2016). For both types of regression model for the mean mark, we will use as predictors indicators of each biweekly calendar period of the primary endpoint event, and, for the geographic region-pooled analysis, also indicators representing the geographic regions (US, South Africa, Colombia, Latin America excluding Colombia) together with all two-way interactions of these region indicators and biweekly calendar periods of the primary endpoint

event. The US and South Africa analyses will include the biweekly calendar periods, and the Latin America analysis will include the indicator of Colombia, the biweekly calendar periods, and all of their interactions.

The mark-specific proportional hazards model in Heng et al. (2022) will additionally adjust for two indicators of geographic regions (US, South Africa, Latin America) in the region-pooled analysis of all binary and non-binary categorical marks. In the region-specific analysis in Latin America, the mark-specific proportional hazards model in Heng et al. (2022) will not adjust for the indicator of Colombia. Covariate adjustment is preferred over stratification in applications of Heng et al. (2022) because stratification requires the same support of the mark across all strata for this method.

2.7 Details of the Approach B cumulative incidence based VE sieve analysis method

For binary mark V (taking values 0 and 1), an extension of the approach of Benkeser et al. (2019) is used to estimate $VE_c(t, v)$ for all time points through time τ, with 95% pointwise CIs in t and 2-sided p-valued for $H_0 : VE_c(\tau, v) = 0$, for each $v = 0, 1$. The approach is also applied to estimate the differential vaccine efficacy sieve effect parameter $VSE_c(t) = (1 - VE_c(t, 1))/(1 - VE_c(t, 0))$ with 95% pointwise CIs in t, and to obtain a two-sided p-value for testing $H_0 : VSE_c(\tau) = 0$.

Results will be presented as in Figure 1 of Benkeser et al. (2019). The original method is coded in the R package *survtmle*, while extensions to account for missing marks have been validated via extensive realistic simulations. The implementation requires an integer failure time, that may be on a day scale or binned into discrete failure times. We will estimate all parameters using the daily scale through to time τ. If instability is observed, particularly for t late in follow-up, we may require some late failure times to be binned together.

The approach requires estimation of several quantities. We describe these quantities and their estimation approach below.

- The probability of receiving vaccine as a function of covariates (i.e., the propensity score). This will be estimated using a main terms glm adjusting for geographic region and biweekly calendar indicators of date of enrollment; here geographic region is (US, South Africa, Colombia, Latin America excluding Colombia) for the geographic-region pooled analyses and is (Colombia, Latin America excluding Colombia) for the Latin America analysis.
- The total hazard for COVID-19 as a function of time given vaccination status and baseline covariates. This will be estimated using a pooled super learner (as in Benkeser et al. 2019) with negative log likelihood loss. The library that includes the following learners: SL.ranger, SL.xgboost (tree depth = 2, 4, 6, 8), SL.earth, and several implementations of SL.glm ((i) with region-specific cubic splines for each treatment arm; (ii) with region- and treatment-specific intercepts, and linear and squared terms for time; (iii) model (i), but additionally adjusting for calendar time of enrollment; (iv) model (ii), but additionally adjusting for calendar time of enrollment).
- The mark distribution as a function of time and given vaccination status, baseline covariates, and viral load. This will be estimated using the same super learner library as the total hazard with the viral load variable included in the models.

- The mean of the mark distribution estimate above (i.e., a sequential regression, as described in e.g., Benkeser et. al 2019) as a function of time and given vaccination status, baseline covariates. This will be estimated using the same super learner library as the total hazard.
- The probability of mark missingness, which will be estimated using the logistic regression model described above.
- The censoring survival distribution, which will be estimated using pooled super learning and the same library as for the total hazard.

Appendix A contains additional details, including how estimation and inference is done for $VE_c(t, v)$ when V is an ordered categorical/count variable with three or more levels.

2.8 Understanding the missing sequence data and adapting the statistical methods

"Phase 1 data" refers to data measured in all endpoint cases, whereas "Phase 2 data" refers to the sequence data that are only available/measured in a subset of cases. The Approach A and Approach B methods described above include models for both: (1) the probability of observing a sequence conditional on phase 1 data; and (2) the mean of the mark V conditional on phase 1 data, where 'phase 1 data' refers to variables collected in all participants, which may include variables only collected in COVID-19 endpoint cases such as calendar date of sampling for sequencing and viral load of the sample used for sequencing. The DRTMLE and survtmle methods are doubly robust, in providing consistent estimation of the parameter of interest if either the model in (1) or (2) is correctly estimated. (A small amount of loss-to-follow-up or consistent estimation of the model for right-censoring is also needed.)

2.8.1 Diagnostics to help decide the versions of the methods to use

The phase 3 trials quantify SARS-CoV-2 viral load at illness visits (COVID-19 endpoint times) in various ways, e.g. by real-time PCR (qPCR) Cycle threshold value. The viral load variable is reported as log10 copies/ml at the illness visit. Note that viral load is viewed as phase one data, as only primary endpoint cases with viral load data are included in sieve analyses.

Let $\pi(O_i)$ be the probability that COVID-19 endpoint case i has a sequence successfully measured. Several studies have shown that the probability of missing a sequence increases with decreasing log10 viral copies/ml (V_c): $\pi(V_c)$ is increasing with increasing V_c. All of the methods described above use inverse probability weighting (IPW), which assume that for all COVID-19 endpoint cases i

$$\pi(O_i) > \epsilon > 0$$

for some constant $\epsilon > 0$ (the positivity assumption).

Therefore, if there exists a constant L such that $\pi(V_c < L) = 0$, then it may be well argued that methods using IPW weights should not be used. To guide methods selection, we will do diagnostic plots of the estimated probabilities $\pi(O_i)$ as part of the unsupervised learning analysis. If the diagnostics support some outlying small estimates, especially due to low V_c value, then the methods using IPW will not be used. Instead, the following will be done:

- Based on the diagnostic plots, especially of the number of sequence reads versus V_c value, a threshold value $V_c = L$ will be selected such that at least 5-10% of endpoint cases with V_c value near L have a clearly successfully measured sequence. Then, the sieve analysis is done for the study endpoint modified to be the original endpoint definition and also requiring $V_c \geq L$. For studying sieve effects against this new endpoint, the positivity assumption now holds by design, such that methods that use IPW are expected to perform satisfactorily. Therefore, the sieve analysis now assesses differential VE against COVID-19 with a minimal requirement for the level of viral load. This endpoint is clinically and public-health relevant, because viral load is associated with the severity of symptomatic disease and with transmissibility. Note that the V_c value for the new endpoint definition is taken from the same sample from which the sequence is measured, which is typically the virological-confirmation sample for the COVID-19 endpoint (the illness-visit).

Analysis of $\pi(V_c)$ via logistic regression modeling showed that estimated $\pi(V_c)$ is above 0.1 for all values of V_c of the COVID-19 endpoint, for all three geographic regions. For the severe-critical COVID-19 endpoint, the estimated $\pi(V_c)$ is above 0.1 for Latin America and the US and above 0.04 for South Africa, which are all deemed sufficient for stable inference via IPW-based methods.

In addition, diagnostics are also needed to decide whether the simple non-doubly robust IPW versions of Hyun et al. (2012), Juraska and Gilbert (2016), and Benkeser et al. (2020) will be used, as compared to use of the doubly robust versions that include models of the mark V conditional on phase 1 data. Diagnostics will study how well sequence features V can be predicted from the phase 1 data O, where the better the prediction, the stronger justification for using a doubly-robust method. If the decision is unclear based on these diagnostics, a simulation study with true data generating distribution defined to match features of the Phase 3 trial will be used to decide this question. After the sieve analysis is done, post-hoc diagnostics will be done to help interpret the validity of the analysis. The AA sequence features V will be plotted by V_c value, stratified by vaccine COVID-19 cases and placebo COVID-19 cases. A similar relationship by treatment arm may help support use of a complete-case method that makes a missing completely at random assumption.

2.9 Superlearner classification sieve analysis

A classification sieve analysis provides information about any association between AA sequence features and assignment to the vaccine or placebo group; an association constitutes a sieve effect. In this analysis, Superlearner is applied to build models for classifying Spike protein sequences into the vaccine vs. placebo arm classes as a function of all the AA sequence features V. Only sequence features passing the sufficient variability screens are included.

The superlearner classification analysis is conducted two times:

1. All PPBN COVID-19 endpoint cases, restricting to screened-in **All features**
2. All PPBN COVID-19 endpoint cases, restricting to screened-in **nAb-CoP-hypothesis features**

We use a similar superlearner ensemble modeling procedure for this task as in the binary mark outcome approach in Section 2.5; we use a logistic link function for each candidate learner and 20-

Candidate Learner	R Implementation (Super Learner function)	Tuning Parameter and possible values	Tuning parameter description
Sample mean	`mean` (`SL.mean`)	—	—
GLM	`glm` (`SL.glm`)	—	—
Bayesian GLM	`arm` (`SL.bayesglm`)	—	—
GLM with all pairwise interactions	`glm` (`SL.glm.interaction`)	—	—
Support Vector Machines (SVMs)	`kernlab` (`SL.ksvm`)	—	—
Multivariate adaptive regression splines	`SL.earth`		
Random forests	`ranger` (`SL.ranger`)	`min.node.size` $\in \{1, 10, 25, 50\}$	Minimum number of observations in terminal node
Gradient boosted trees	`xgboost` (`SL.xgboost`)	`shrinkage` $\in \{.1, .01, .001\}$	Learning rate
Elastic net	`glmnet` (`SL.glmnet`)	mixing parameter $\alpha = 1$	Trade-off between ℓ_1 and ℓ_2 regularization†

Table 10: Candidate learners in the Super Learner ensemble for predicting randomization arm, along with their R implementation, tuning parameter values, and description of the tuning parameters. All tuning parameters besides those listed here (and below) are set to their default values. In particular, stepwise regression is done via AIC (setting $k = 2$ in `step`); SVMs are fit with the radial basis (Gaussian) kernel, automatic hyperparameter selection, no cross-validation, $\nu = 0.2$, and $C = 1$; multivariate adaptive regression splines are fit allowing two-way interactions, no cross-validation, maximum number of model terms in the forward pass equal to $\max\{21, 2p+1\}$, backward pruning, and no mininum number of observations between knots or before the first and after the final knot; the random forests are grown with 500 trees, a subsampling fraction of 0.632, and select $\sqrt{p}$ features to possibly split on at each node; the boosted trees are grown with a maximum of 500 trees, max depth of 4, and a minimum of 10 observations per node. The total number of features is denoted by p.

†: $\alpha = 1$ denotes the lasso (i.e., no ℓ_2 regularization).

fold cross-validation to select the combination of learners that minimizes the non-negative binomial log-likelihood loss function. The cross-validation is stratified by the outcome to ensure a similar ratio of intervention-to-control within each cross-validation fold. The specific candidate learners that we consider are listed in Table 10; this is an augmented version of the treatment and propensity score models listed in Section 2.5, where we have added random forests and are using several tuning parameter values for both the random forests and boosted trees. Since there are many AA sequence features, we also consider two variable screens within the superlearner: a lasso pre-screen (only variables with non-zero lasso coefficient are retained) and a univariate correlation screen (only variables in the top half of univariate outcome-feature correlations are retained). We will run two sets of superlearner analyses: pooled across geographic regions and specific to Latin America. All pooled superlearner analyses will be adjusted for a baseline variable defined as indicators coding for the geographic regions (US, South Africa, Colombia, Latin America excluding Colombia), indicators of biweekly calendar period of enrollment in the study, and all two-way interactions between these region indicators and calendar periods. The Latin America analyses will be adjusted for a baseline variable defined as an indicator coding for Colombia, indicators of biweekly calendar period of enrollment in the study, and all two-way interactions between the Colombia indicator and calendar periods. These variables are defined based on treatment-blinded data.

All amino acid sequence features undergo pre-filtering for enough representation in the data set, requiring at least 10 COVID-19 endpoints with a given feature to include the feature. For example, the variant mu is only included if at least 10 COVID-19 endpoints with variant mu, and K163 is only included if at least 10 COVID-19 endpoints have residue K at spike AA position 163. A second pre-filtering step is applied that removes one of any pair of variables with pairwise correlation greater than 0.95.

To quantify classification accuracy and hence the strength of an overall sieve effect, the cross-validated area under the ROC curve (CV-AUC) of the superlearner model will be estimated using an outer layer of five-fold cross-validation, along with a 95% confidence interval, implemented in the *cvAUC* package on CRAN (LeDell et al., 2015). For comparison, the point and 95% CI estimates of CV-AUC for the null model with baseline factors only will also be reported, and the point and 95% CI estimate of the difference in CV-AUC (AA sequence inputs plus baseline factors vs. baseline factors) will be reported. In addition, the variable importance of each defined set of AA sequence features will be computed (implemented in the *vimp* package on CRAN)(Williamson et al., 2021, 2020). This *intrinsic* variable importance is defined as the increase in population prediction potential when adding the AA feature set to the baseline factors; this prediction potential is measured using AUC. For the analyses of **All features**, the sets of features are listed in Table 1. For the analyses of **nAb-CoP-hypothesis features**, the sets of features are listed in Table 2. A forest plot will be shown of point and 95% CI estimates of CV-AUC, for each superlearner model defined by a set of AA sequence features (including the no AA features and all AA features models). In addition, a variable importance forest plot will be made showing the differences in population AUC, using the model with no AA sequence features as the reference model.

Given that the diagnostics confirmed appropriateness of the use of methods that use inverse probability weighting, all of the analyses will use inverse probability weighting to account for missing sequence features.

2.10 Multiple hypothesis testing adjustment for AA sequence sieve analysis

For the AA sequence sieve analysis, the following plan is implemented for multiple testing adjustment, separately for each region-specific and the region-pooled analysis:

Holm-Bonferroni p-values are used to estimate family-wise error rate (FWER) adjusted p-values and FDR-adjusted p-values, done separately for the two PPBN cohort analyses defined by **All features** and **nAb-CoP-hypothesis features**, and within each of these analysis types separately for each of the following classes of defined feature set.

For **All features**, the following classes of feature sets are defined:

- (1a) WHO lineages/variants;
- (1b) all match/mismatch amino acid position scanning analyses (local features);
- (1c) all present/absent amino acid position scanning analyses (local features);
- (1d) spike and spike subprotein distance-based sieve analyses (global features) (physicochemical-weighted Hamming distances to the vaccine strain).

For **nAb-CoP-hypothesis features**, the following classes of feature sets are defined:

- (2a) variant-neutralization resistance score assigned to lineage/variant;
- (2b) all neutralization-relevant match/mismatch amino acid position analyses (local features);
- (2c) all neutralization-relevant present/absent amino acid position analyses (local features);
- (2d) NTD neutralization-relevant features (local features);
- (2e) antibody escape scores (global features) using Bloom et al. deep mutational scanning RBD antibody-escape scores;
- (2f) antibody escape scores (global features) using spike antibody-escape scores from the PDB database.

Differential VE tests for classes (1a)-(1c), (2b)-(2d) are based on $VE_h(v)$ by Heng et al. Cox modeling, and differential VE tests for class (1d), (2a), and (2e)-(2f) are based on $VE_h(v)$ Juraska et al. Cox modeling. For (1a), note that for the overall test for any variation in $VE_h(v)$ across the lineages/variants in a given region, there is no multiplicity adjustment for the single p-value used to test the global null hypothesis. However, for pairwise comparisons of $VE_h(v)$ across pairs of variants as well as comparisons of each lineage vs. not that lineage within a geographic region, the multiplicity adjustment is applied. For (2a), there is no multiplicity adjustment for the single p-value from testing any variation in $VE_h(v)$ across the variant-neutralization resistance score in a given region.

For each set of marks for multiplicity correction within each region-specific or region-pooled analysis, the FWER- and FDR-adjusted p-values are computed for each set of sieve analyses that are conducted, restricting only to differential $VE_h(v)$ sieve effect tests that are included for every feature type. (Therefore multiplicity adjustment is not done for the $VE_c(t, v)$ sieve effect analyses.) Unadjusted p-values are reported for all sieve analyses that are conducted.

Definition of statistical significance in the context of multiple hypothesis testing. For 2-sided testing, FWER statistical significance is defined by FWER p-value ≤ 0.05 (and also requiring unadjusted p-value ≤ 0.05), whereas FDR statistical significance is defined by FDR p-value ≤ 0.20 (and also requiring unadjusted p-value ≤ 0.05). For 1-sided testing, 1-sided unadjusted p-values will first be doubled, then the multiplicity adjustment will be applied to them, and afterwards they will be presented as doubled 1-sided p-values, unadjusted and adjusted, with the same significance thresholds as used for 2-sided testing.

References

Aalen, O. and Johansen, S. (1978), “An empirical transition matrix for non-homogeneous Markov chains based on censored observations.” *Scandinavian Journal of Statistics*, 5, 141–150.

Benkeser, D., Carone, M., Laan, M. V. D., and Gilbert, P. (2017), “Doubly robust nonparametric inference on the average treatment effect,” *Biometrika*, 104, 863–880.

Benkeser, D., Gilbert, P. B., and Carone, M. (2019), “Estimating and Testing Vaccine Sieve Effects Using Machine Learning,” *Journal of the American Statistical Association*, 114, 1038–1049.

Benkeser, D., Juraska, M., and Gilbert, P. B. (2020), “Assessing Trends in Vaccine Efficacy by Pathogen Genetic Distance.” *Societe Francaise de Statistique*, 161.

Corey, L., Gilbert, P., Juraska, M., Montefiori, D., Morris, L., Karuna, S., Edupuganti, S., and et al. (2021), “Two randomized trials of neutralizing antibodies to prevent HIV-1 acquisition,” *New England Journal of Medicine*.

Follmann, D. and Huang, C.-Y. (2018), “Sieve analysis using the number of infecting pathogens,” *Biometrics*, 74, 1023–1033.

Fong, Y., Huang, Y., Benkeser, D., Carpp, L., Anez, G., Woo, W., McGarry, A., Dunkle, L., Cho, I., Houchens, C., Martins, K., Jayashankar, L., Castellino, F., Petropoulos, C., Leith, A., Haugaard, D., Webb, B., Lu, Y., Yu, C., Borate, B., van der Laan, L., Hejazi, N., Randhawa, A., Andrasik, M., Kublin, J., Hutter, J., Keshtkar-Jahromi, M., Beresnev, T., Corey, L., Neuzil, K., Follmann, D., Ake, J., Gay, C., Kotloff, K., Koup, R., Donis, R., Gilbert, P., the Immune Assays Team, t. C. V. P. N. C.-. P. I., and Study Team, t. U. S. G. U. B. T. (2023), “Immune Correlates Analysis of the PREVENT-19 COVID-19 Vaccine Efficacy Clinical Trial,” *Nature Communications*.

Fong, Y., McDermott, A. B., Benkeser, D., Roels, S., Stieh, D. J., Vandebosch, A., Le Gars, M., Van Roey, G. A., Houchens, C. R., Martins, K., Jayashankar, L., Castellino, F., Amoa-Awua, O., Basappa, M., Flach, B., Lin, B., Moore, C., Naisan, M., Naqvi, M., Narpala, S., O'Connell, S., Mueller, A., Serebryannyy, L., Castro, M., Wang, J., Petropoulos, C., Luedtke, A., Hyrien, O., Lu, Y., Yu, C., Borate, B., van der Laan, L., Hejazi, N., Kenny, A., Carone, M., Wolfe, D., Sadoff, J., Gray, G., Grinsztejn, B., Goepfert, P., Little, S., de Sousa, L., Maboa, R., Randhawa, A., Andrasik, M., Hendriks, J., Truyers, C., Struyf, F., Schuitemaker, H., Douoguih, M., Kublin, J., Corey, L., Neuzil, K., Carpp, L., Follmann, D., Gilbert, P., Koup, R., Donis, RO on behalf of the Immune Assays Team, t. J. T. t. C. V. P. N. C. T., and the United States Government CoVPN Biostatistics Team (2022), “Immune correlates analysis of the ENSEMBLE single Ad26.COV2.S dose vaccine efficacy clinical trial,” *Nature Microbiology*, 7, 1996–2010.

Gilbert, P. B. (2000), “Comparison of competing risks failure time methods and time-independent methods for assessing strain variations in vaccine protection.” *Statistics in Medicine*, 19, 3065–3086.

Gilbert, P. B., Bosch, R., and Hudgens, M. (2003), “Sensitivity analysis for the assessment of vaccine effects on viral load in HIV vaccine trials,” *Biometrics*, 59, 531–541.

Gilbert, P. B., Montefiori, D. C., McDermott, A. B., Fong, Y., Benkeser, D., Deng, W., Zhou, H., Houchens, C. R., Martins, K., Jayashankar, L., et al. (2022), "Immune correlates analysis of the mRNA-1273 COVID-19 vaccine efficacy clinical trial," *Science*, 375, 43–50.

Gilbert, P. B., Self, S. G., and Ashby, M. A. (1998), "Statistical methods for assessing differential vaccine protection against human immunodeficiency virus types," *Biometrics*, 799–814.

Grambsch, P. and Therneau, T. (1994), "Proportional hazards tests and diagnostics based on weighted residuals." *Biometrika*, 81, 515–526.

Heng, F., Sun, Y., and Gilbert, P. (2022), "Estimation and hypothesis testing of strain-specific vaccine efficacy with missing strain types, with applications to a COVID-19 vaccine trial," *arXiv*, 2201.08946.

Hyun, S., Lee, J., and Sun, Y. (2012), "Proportional hazards model for competing risks data with missing cause of failure." *Journal of Statistical Planning and Inference*, 142, 1767–1779, pMCID: PMC3314432.

Juraska, M. and Gilbert, P. B. (2016), "Mark-specific hazard ratio model with missing multivariate marks." *Lifetime Data Analysis*, 22, 606–625, pMCID: PMC4848257.

Kalbfleisch, J. and Prentice, R. (2002), *The Statistical Analysis of Failure Time Data, Second Edition.*, New York: John Wiley and Sons.

LeDell, E., Petersen, M., and van der Laan, M. (2015), "Computationally efficient confidence intervals for cross-validated area under the ROC curve estimates," *Electronic Journal of Statistics*, 9, 1583, pMCID: PMC4533123.

Lunn, M. and McNeil, D. (1995), "Applying Cox regression to competing risks." *Biometrics*, 51, 524–532.

McCallum, M., De Marco, A., Lempp, F. A., Tortorici, M. A., Pinto, D., Walls, A. C., Beltramello, M., Chen, A., Liu, Z., Zatta, F., et al. (2021), "N-terminal domain antigenic mapping reveals a site of vulnerability for SARS-CoV-2," *Cell*, 184, 2332–2347.

Neafsey, D. E., Juraska, M., Bedford, T., Benkeser, D., Valim, C., Griggs, A., Lievens, M., Abdulla, S., Adjei, S., Agbenyega, T., Agnandji, S. T., Aide, P., Anderson, S., Ansong, D., Aponte, J. J., Asante, K. P., Bejon, P., Birkett, A. J., Bruls, M., Connolly, K. M., D'Alessandro, U., Dobaño, C., Gesase, S., Greenwood, B., Grimsby, J., Tinto, H., Hamel, M. J., Hoffman, I., Kamthunzi, P., Kariuki, S., Kremsner, P. G., Leach, A., Lell, B., Lennon, N. J., Lusingu, J., Marsh, K., Martinson, F., Molel, J. T., Moss, E. L., Njuguna, P., Ockenhouse, C. F., Ogutu, B. R., Otieno, W., Otieno, L., Otieno, K., Owusu-Agyei, S., Park, D. J., Pellé, K., Robbins, D., Russ, C., Ryan, E. M., Sacarlal, J., Sogoloff, B., Sorgho, H., Tanner, M., Theander, T., Valea, I., Volkman, S. K., Yu, Q., Lapierre, D., Birren, B. W., Gilbert, P. B., and Wirth, D. F. (2015), "Genetic diversity and protective efficacy of the RTS,S/AS01 malaria vaccine," *New England Journal of Medicine*, 373, 2025–2037, pMCID: PMC4762279.

Rolland*, M., Edlefsen*, P., Larsen, B., Tovanabutra, S., Sanders-Buell, E., Hertz, T., DeCamp, A., Carrico, C., Menis, S., Magaret, C., Ahmed, H., Juraska, M., Chen, L., Konopa, P., Nariya, S., Stoddard, J., Wong, K., Zhao, H., Deng, W., Maust, B., Bose, M., Howell, S., Bates, A., Lazzaro, M., O'Sullivan, A., Lei, E., Bradfield, A., Ibitamuno, G., Assawadarachai, V., O'Connell, R., deSouza, M., Nitayaphan, S., Rerks-Ngarm, S., Robb, M., McLellan, J., Georgiev, I., Kwong, P., Carlson, J., Michael, N., Schief, W., Gilbert*, P., Mullins*, J., and Kim*, J. (2012), "Increased HIV-1 vaccine efficacy against viruses with genetic signatures in Env V2." *Nature*, 490, 417–420, *Contributed equally. PMCID: PMC3551291.

Rolland, M. and Gilbert, P. (2021), "Sieve analysis to understand how SARS-CoV-2 diversity can impact vaccine protection," *PLoS Pathogens*, in press.

Sadoff, J., Gray, G., Vandebosch, A., Cárdenas, V., Shukarev, G., Grinsztejn, B., Goepfert, P. A., Truyers, C., Fennema, H., Spiessens, B., et al. (2021), "Safety and Efficacy of Single-Dose Ad26.COV2.S Vaccine against Covid-19," *New England Journal of Medicine*.

Sadoff, J., Gray, G., Vandebosch, A., Cárdenas, V., Shukarev, G., Grinsztejn, B., Goepfert, P. A., Truyers, C., Van Dromme, I., Spiessens, B., et al. (2022), "Final analysis of efficacy and safety of single-dose Ad26. COV2. S," *New England Journal of Medicine*, 386, 847–860.

Sandberg, M., Eriksson, L., Jonsson, J., Sjöström, M., and Wold, S. (1998), "New chemical descriptors relevant for the design of biologically active peptides. A multivariate characterization of 87 amino acids," *Journal of medicinal chemistry*, 41, 2481–2491.

Shepherd, B., Gilbert, P. B., Jemiai, Y., and Rotnitzky, A. (2006), "Sensitivity analyses comparing outcomes only existing in a subset selected post-randomization, conditional on covariates, with application to HIV vaccine trials." *Biometrics*, 62, 332–342.

Starr, T. N., Greaney, A. J., Hilton, S. K., Ellis, D., Crawford, K. H., Dingens, A. S., Navarro, M. J., Bowen, J. E., Tortorici, M. A., Walls, A. C., et al. (2020), "Deep mutational scanning of SARS-CoV-2 receptor binding domain reveals constraints on folding and ACE2 binding," *Cell*, 182, 1295–1310.

Williamson, B., Gilbert, P., Simon, N., and Carone, M. (2021), "A general framework for inference on algorithm-agnostic variable importance," *Journal of the American Statistical Association (Theory & Methods)*.

Williamson, B. D., Simon, N., and Carone, M. (2020), "vimp: Perform Inference on Algorithm-Agnostic Variable Importance," R package version 2.0.2.

3 Appendix A: Additional statistical details of sieve analysis methods

3.1 Details of the Approach B sieve analysis methods

Let V be some discrete AA sequence feature of the acquired SARS-CoV-2 virus. We suppose V takes J distinct values, $j = 0, \cdots, J$. Two types of target parameters are used, based on cumulative incidence parameters and proportional hazards parameters. Let $F_z(t, j) \equiv P(T \leq t, V = j | Z = z)$ be the cumulative incidence of COVID-19 of genotype j by the end of discrete interval t, for group $Z = z$ ($z = 0$ indicates assignment to vaccine and $z = 1$ indicates assignment to placebo). Let $\lambda_{zj}(t)$ be the genotype j-specific discrete hazard of COVID-19 in discrete interval t for group $Z = z$, for $z = 0, 1$.

Then, for each genotype $j = 0, \cdots, J$, the two types of genotype-specific vaccine efficacy parameters are defined by:

$$VE^{\text{cum}}(t, j) = 1 - \frac{F_1(t, j)}{F_0(t, j)} \quad (3)$$

$$VE^{\text{haz}}(j) = 1 - \frac{\lambda_1(t, j)}{\lambda_0(t, j)}. \quad (4)$$

The cumulative vaccine efficacy parameter $VE^{\text{cum}}(t, j)$ can vary with time, and thus has advantage that time-effects can be directly assessed. In contrast, use of the proportional hazards vaccine efficacy parameter $VE^{\text{haz}}(j)$ assumes proportional cause-specific hazards, and, if the model is approximately correct, has advantage of being a single summary measure that accounts for all follow-up time.

The Grambsch and Therneau (1994) test and graphical procedures are applied to assess the cause-specific proportional hazards assumption for the case of discrete genotypes.

Models and estimation procedures without covariate adjustment. For the analyses that do not adjust for covariates, the cumulative incidence functions $F(t, j | Z = z) \equiv P(T \leq t, V = j | Z = z)$, $z = 0, 1$, will be estimated by the nonparametric maximum likelihood estimator (NPMLE) (Aalen and Johansen, 1978), implemented in the R `cmprsk` package, based on the Kaplan-Meier estimate of the overall survival function and the Nelson-Aalen estimates of the genotype-specific cumulative hazard functions (see, e.g., Kalbfleisch and Prentice, 2002). The adjusted analyses that estimate the same parameter use targeted minimum loss-based estimation (Benkeser et al., 2019), which were applied to the RTS,S malaria VE trial sieve analysis (Neafsey et al., 2015).

For quantitative marks V such as count variables, we will estimate $VE^{\text{haz}}(j)$ for each level j of the genetic distance using the competing risks Cox model

$$\lambda(t, j | Z = z) = \lambda_0(t, j) \exp\{\widetilde{\alpha}_j z\}.$$

The genotype-specific log hazard ratios $\widetilde{\alpha}_j$ are estimated by the maximum partial likelihood estimation method. The parameter $VE^{\text{haz}}(j)$ is estimated by the method of Juraska and Gilbert (2016).

Hypothesis testing. Statistical tests for a "sieve effect" assess the following null hypothesis:

H_0: Constant vaccine efficacy against all SARS-CoV-2 genotypes j under consideration.

We use the following procedure to test this null hypothesis in terms of the cumulative vaccine efficacy parameter $VE^{\text{cum}}(\tau, j)$, where τ is defined near the top of Section 2. Here j takes two levels (match or mismatch) or it takes J levels, either as the levels of a Hamming distance to the vaccine strain or as unordered categorical genotypes. For the first two cases, we specify these J levels as integers $j = 0, 1, \cdots, J$ (e.g., numbers of mismatches for Hamming distances), unifying the testing approach with a single procedure for an ordered categorical/count type. (Below we also consider testing for an unordered categorical genotype.)

In particular, the following procedure can be used for unadjusted or covariate-adjusted analysis. Denote $\theta_{zj} = F(t_0, j|Z = z)$, where $z = 0, 1$, $j = 1, \ldots, J$, $J \geq 2$, is an ordered categorical/count genotype, and t_0 is fixed. Let $\boldsymbol{\theta} = (\theta_{01}, \theta_{11}, \ldots, \theta_{0J}, \theta_{1J})^T$ and $\widehat{\boldsymbol{\theta}}$ be the Aalen-Johansen (1978) estimator for $\boldsymbol{\theta}$. Then

$$\sqrt{n}\left(\widehat{\boldsymbol{\theta}} - \boldsymbol{\theta}\right) \xrightarrow[n\to\infty]{\mathcal{D}} \mathsf{N}_{2J}(\mathbf{0}, \boldsymbol{V}).$$

Consider the influence curve-based variance estimator $\widehat{\boldsymbol{V}}$ for $\boldsymbol{V}$. Next consider the transformation $\boldsymbol{g}(\boldsymbol{\theta}) = \left(\log \frac{\theta_{01}}{\theta_{11}}, \ldots, \log \frac{\theta_{0J}}{\theta_{1J}}\right)^T$ and denote

$$\dot{\boldsymbol{g}}(\boldsymbol{\theta}) = \left(\frac{\partial g_j(\boldsymbol{\theta})}{\partial \theta_i}\right)_{\substack{i=1,\ldots,2J\\ j=1,\ldots,J}}.$$

The delta method yields

$$\sqrt{n}\left(\boldsymbol{g}(\widehat{\boldsymbol{\theta}}) - \boldsymbol{g}(\boldsymbol{\theta})\right) \xrightarrow[n\to\infty]{\mathcal{D}} \mathsf{N}_J(\mathbf{0}, \boldsymbol{\Sigma}),$$

where $\boldsymbol{\Sigma} = \dot{\boldsymbol{g}}(\boldsymbol{\theta})^T \boldsymbol{V} \dot{\boldsymbol{g}}(\boldsymbol{\theta})$. Consider the estimator $\widehat{\boldsymbol{\Sigma}} = \dot{\boldsymbol{g}}(\widehat{\boldsymbol{\theta}})^T \widehat{\boldsymbol{V}} \dot{\boldsymbol{g}}(\widehat{\boldsymbol{\theta}})$ for $\boldsymbol{\Sigma}$. It is of interest to test the null hypothesis

$$H_0 : VE(t_0, j) = VE(t_0) \text{ for all } j = 1, \ldots, J.$$

To this end, consider approximating $\log \frac{\theta_{0j}}{\theta_{1j}}$ as a function of j with a straight line fit to the points $\log \frac{\widehat{\theta}_{01}}{\widehat{\theta}_{11}}, \ldots, \log \frac{\widehat{\theta}_{0J}}{\widehat{\theta}_{1J}}$:

$$\log \frac{\widehat{\theta}_{0j}}{\widehat{\theta}_{1j}} \approx \beta_0 + \beta_1 (j-1).$$

Then H_0 is equivalent to $H_0^* : \beta_1 = 0$. Denote $\boldsymbol{\beta} = (\beta_0, \beta_1)^T$ and

$$\boldsymbol{X} = \begin{pmatrix} 1 & 0 \\ 1 & 1 \\ \vdots & \vdots \\ 1 & J-1 \end{pmatrix}.$$

Using the generalized least squares method, we estimate $\boldsymbol{\beta}$ with $\widehat{\boldsymbol{\beta}} = (\boldsymbol{X}^T \widehat{\boldsymbol{\Sigma}}^{-1} \boldsymbol{X})^{-1} \boldsymbol{X}^T \widehat{\boldsymbol{\Sigma}}^{-1} \boldsymbol{g}(\widehat{\boldsymbol{\theta}})$. If $\widehat{\boldsymbol{\Sigma}}$ is the true asymptotic covariance matrix of $\boldsymbol{g}(\widehat{\boldsymbol{\theta}})$, then

$$\sqrt{n}\left(\widehat{\boldsymbol{\beta}} - \boldsymbol{\beta}\right) \xrightarrow[n\to\infty]{\mathcal{D}} \mathsf{N}_2(\mathbf{0}, \boldsymbol{Q}),$$

where $\boldsymbol{Q} = (q_{ij})_{i,j=1,2} = (\boldsymbol{X}^T \widehat{\boldsymbol{\Sigma}}^{-1} \boldsymbol{X})^{-1}$. Finally, the Wald test of H_0^* is based on the test statistic $\widehat{\beta}_1/\sqrt{q_{22}/n}$ that is approximately $\mathsf{N}(0,1)$ under validity of H_0^*. This testing procedure has been studied in simulations and been shown to have nominal size and to have power to reject the null hypothesis against various alternative hypotheses.

For testing for a sieve effect in terms of genotype-specific hazard ratios, i.e. for testing $H_0 : VE^{\text{haz}}(j)$ is constant in j, a test such as that originally proposed by Lunn and McNeil (1995) will be used, extended to account for missing sequence data through AIPW (Sun, Lee, and Hyun, 2012). This procedure can be applied for J ordered or unordered types; the former via a Wald test of whether $log(HR(j)) - log(HR(j-1))$ assumed linear in j is horizontal, and the latter (unordered) via a generalized Wald test.

3.2 R code for the sieve analysis methods

The planned sieve analysis methods are implemented in R and are publicly available. The following table summarizes the R code that is used.

Table 11: R code for implementing the sieve analyses, where all methods account for missing SARS-CoV-2 sequences from some COVID-19 endpoint cases

Method	R code
Study covariability of AA site pairs	https://github.com/CoVPN/sieve_reporting
Hyun et al. (2012) AIPW mark-specific proportional hazards model Approach B sieve analysis for a binary mark	R package *cmprskPH* at CRAN[1]
Juraska and Gilbert (2016) AIPW mark-specific proportional hazards model Approach B sieve analysis for a quantitative mark	R package *sievePH* at CRAN
Benkeser et al. (2019) TMLE cumulative incidence based VE Approach B sieve analysis for a binary mark	R package *survtmle* at CRAN[2]
Benkeser et al. (2019) TMLE cumulative incidence based VE Approach B sieve analysis for a count mark	R package *sievetrend* at https://github.com/benkeser/
Benkeser et al. (2017) DRTMLE Approach A sieve analysis for a quantitative mark	R package *drtmle* at CRAN[2]

[1]The implementation in Fei Heng's R package cmprskPH is designed to handle the missing data encountered in the COVID-19 VE trials; see the file cmprskPH.0.4.0.pdf in this repository. [2]The implementations handling missing marks are used.

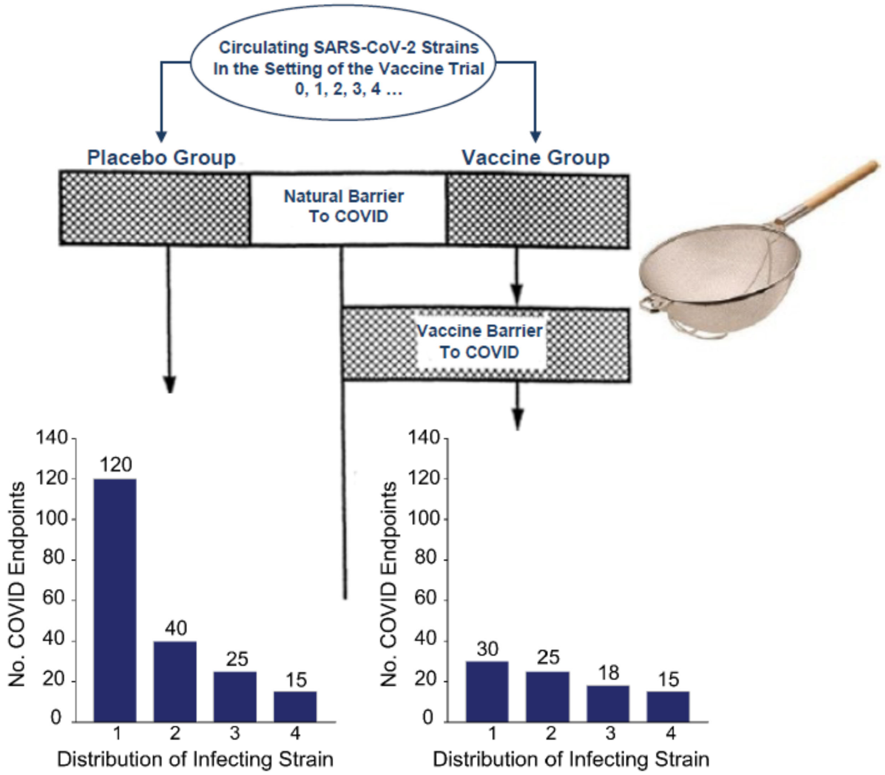

Figure 1: Concept of sieve analysis

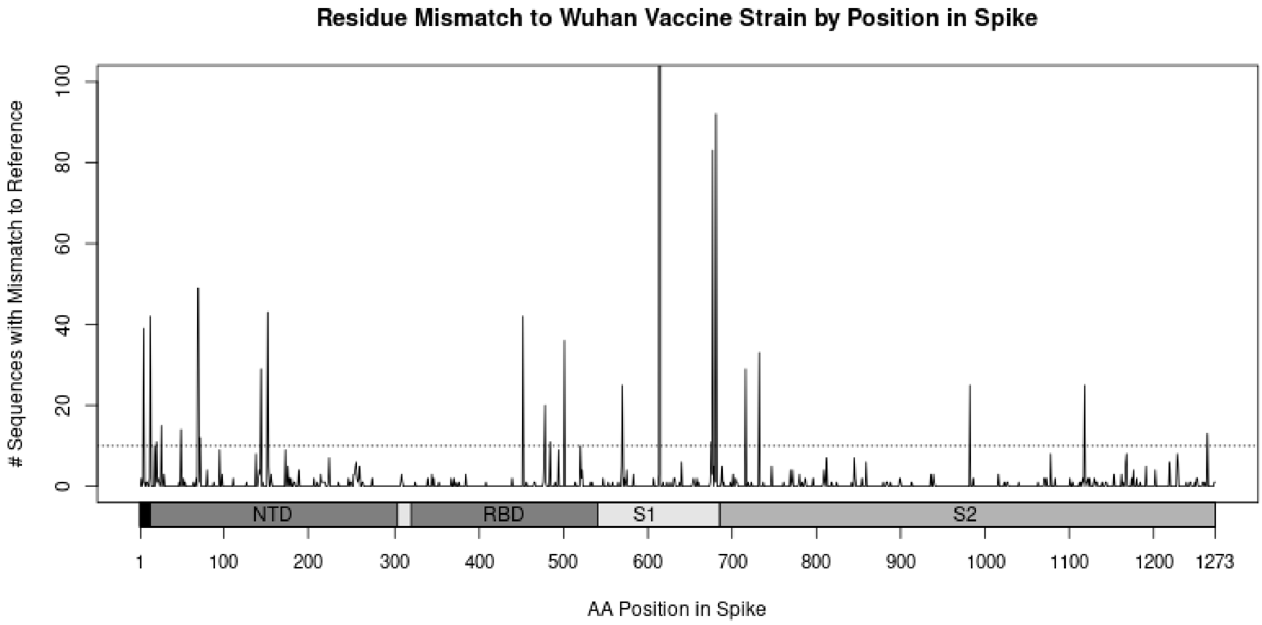

Figure 2: Amino acid position scanning across Spike, showing for each position the number of sequences with a residue mismatch to the Wuhan vaccine strain: 1122 randomly sampled sequences from GISAID from U.S. study sites between September 8 2020 and February 1 2021. If these were the trial sequences, the conclusion would be that 25 of the 1273 amino acid positions would be included in the sieve analysis (sufficient variability). Special note: almost all observed sequences have the G614 mutation, which differs from the D614 in the vaccine strain. This results in the displayed variability at site 614 exceeding the y-axis's upper limit. In this sample, however, only two sequences have D614, which does not equal or exceed the 20-sequence threshold, so site 614 would not be included in the sieve analysis.

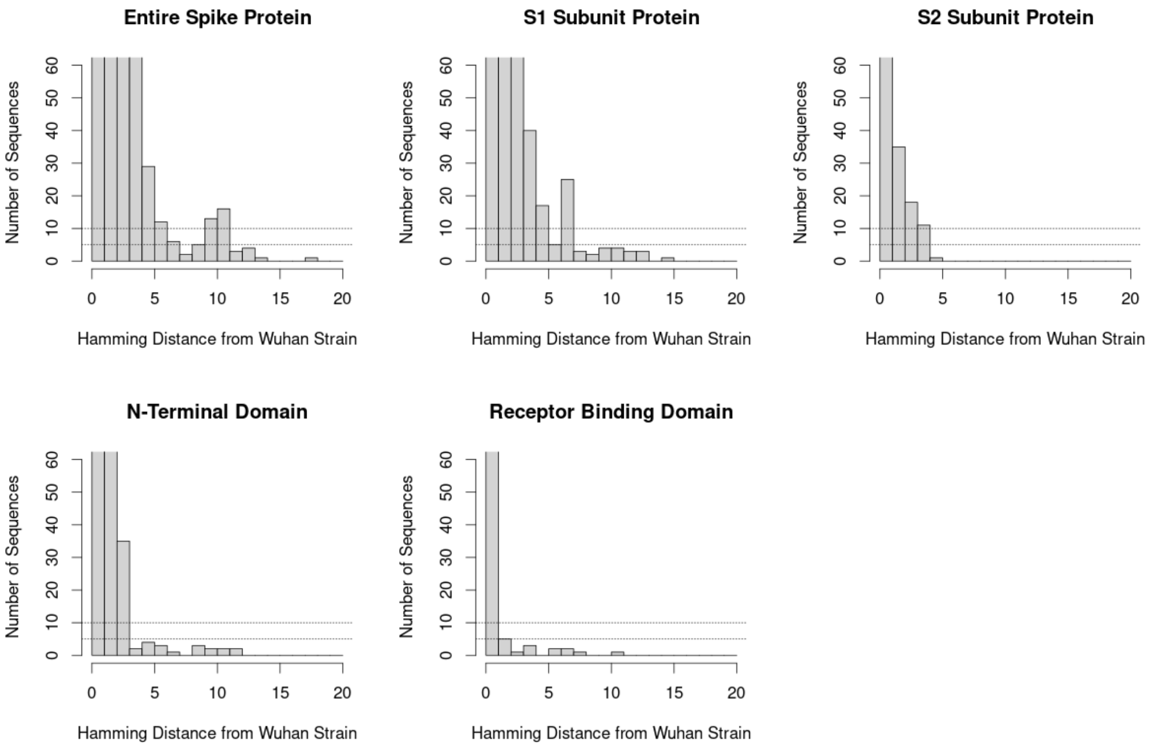

Figure 3: Hamming distances to the Wuhan vaccine strain: 1122 randomly sampled sequences from GISAID from U.S. trial study sites from September 8 2020 to February 1 2021. If these were the trial sequences, the conclusion would be that the all five Hamming distances would be included in the sieve analysis (sufficient variability).

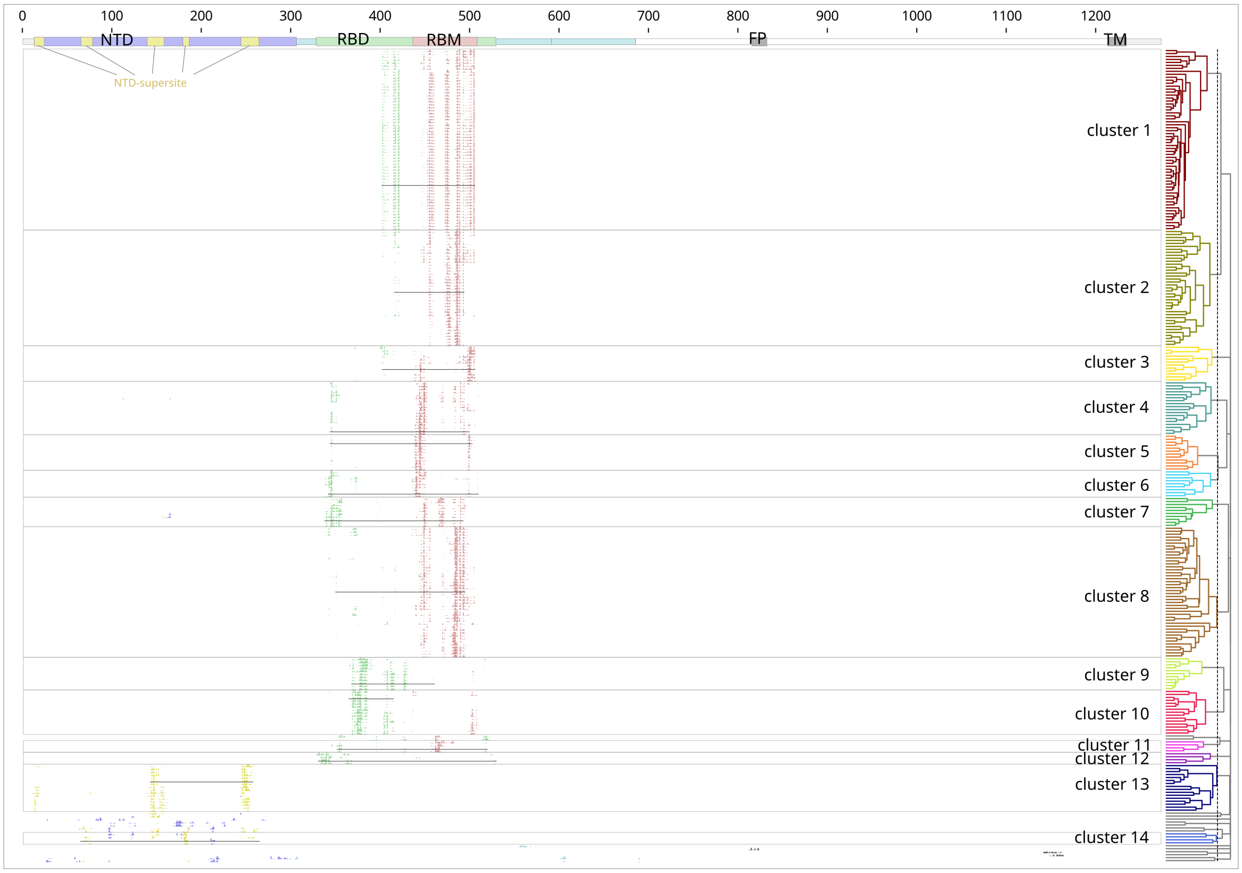

Figure 4: Based on all ENSEMBLE trial primary moderate to severe-critical COVID-19 endpoints starting 14 days after vaccination with sequence data, the plot shows clusters representing major antibody epitope footprints in SARS-CoV-2 Spike. Epitope footprints are represented across the Spike sequence for 274 antibodies. Antibodies with similar epitope footprints are grouped in 14 clusters. Each cluster includes at least 3 antibodies. A representative antibody was identified for each cluster and these representative antibodies are indicated by a line spanning the epitope footprint.
