# Supplementary material for: Quantifying how single dose Ad26.COV2.S vaccine efficacy depends on Spike sequence features
Source: Nat Commun. 2024 Mar 11;15:2175. doi: 10.1038/s41467-024-46536-w (PMC10928100; doi:10.1038/s41467-024-46536-w)
Supplement: Supplementary file 3 — Description of Additional Supplementary Files [file 41467_2024_46536_MOESM3_ESM.pdf]

Description of Additional Supplementary Files

File Name: Supplementary Data 1
Description: Information pertaining to the SARS-CoV-2 sequences obtained from study participants, including their GISAID accession numbers.
