# Supplementary material for: Quantifying how single dose Ad26.COV2.S vaccine efficacy depends on Spike sequence features
Source: Nat Commun. 2024 Mar 11;15:2175. doi: 10.1038/s41467-024-46536-w (PMC10928100; doi:10.1038/s41467-024-46536-w)
Supplement: Supplementary file 5 — Reporting Summary [file 41467_2024_46536_MOESM5_ESM.pdf]

Corresponding author(s): Peter B. Gilbert

Last updated by author(s): Feb 14, 2024

Reporting Summary

Nature Portfolio wishes to improve the reproducibility of the work that we publish. This form provides structure for consistency and transparency in reporting. For further information on Nature Portfolio policies, see our Editorial Policies and the Editorial Policy Checklist.

Statistics

For all statistical analyses, confirm that the following items are present in the figure legend, table legend, main text, or Methods section.

n/a	Confirmed	
☐	☒	The exact sample size (*n*) for each experimental group/condition, given as a discrete number and unit of measurement
☐	☒	A statement on whether measurements were taken from distinct samples or whether the same sample was measured repeatedly
☐	☒	The statistical test(s) used AND whether they are one- or two-sided<br>*Only common tests should be described solely by name; describe more complex techniques in the Methods section.*
☐	☒	A description of all covariates tested
☐	☒	A description of any assumptions or corrections, such as tests of normality and adjustment for multiple comparisons
☐	☒	A full description of the statistical parameters including central tendency (e.g. means) or other basic estimates (e.g. regression coefficient) AND variation (e.g. standard deviation) or associated estimates of uncertainty (e.g. confidence intervals)
☐	☒	For null hypothesis testing, the test statistic (e.g. *F*, *t*, *r*) with confidence intervals, effect sizes, degrees of freedom and *P* value noted<br>*Give P values as exact values whenever suitable.*
☒	☐	For Bayesian analysis, information on the choice of priors and Markov chain Monte Carlo settings
☒	☐	For hierarchical and complex designs, identification of the appropriate level for tests and full reporting of outcomes
☐	☒	Estimates of effect sizes (e.g. Cohen's *d*, Pearson's *r*), indicating how they were calculated

Our web collection on statistics for biologists contains articles on many of the points above.

Software and code

Policy information about availability of computer code

Data collection	SARS-CoV-2 S gene sequences were obtained by next-generation sequencing using Swift Biosciences SNAP workflow version 2.0, on the Illumina platform.<br>For the neutralizing antibody assay, relative light units were measured using an EnSight Multimode Plate Reader (Perkin Elmer) running Kaleido software (version Kaleido 3.0.3067.117x).<br>For cryoEM, data were acquired using Leginon software (Suloway et al. 2005).
Data analysis	Sieve analysis:<br>All custom code for the sieve analysis, including code for: the unsupervised learning of the treatment-blinded trial sequence data to fully specify and down-select the set of amino acid (AA) sequence features that were studied for sieve effects, implementing hazard-based sieve analysis, covariability analysis of any pairs of AA positions, the SuperLearner-based supervised learning sieve analysis, reproducing the figures in the supplemental material, calculating the epitope distance analyses, and generating the structural visualizations in the manuscript is publicly available at Figshare (https://doi.org/10.6084/m9.figshare.24911373.v1).<br>The sequences and clinical data were pre-processed into an analysis dataset as specified by the SAP, using R (version 4.3.1) with the seqinr package (version 4.2-30).<br>For hazard ratio-based prospective VE sieve analyses, the following software was used: R 4.2.3 and Rstudio61 2023.03.0+386. For hazard ratio-based sieve model fitting, the R packages sievePH (version 1.0.4) and cmprskPH63 (unpublished; under the folder "cmprskPH-master") were used. In addition, for parallel computation, the R packages doParallel (version 1.0.17), foreach (version 1.5.2), iterators (1.0.14), and parallel (4.2.2) were used. For data pre-processing, the R packages here (version 1.0.1), haven (version 2.5.2), scales (version 1.2.1), tidyverse (version 2.0.0), plyr (version 1.8.8), and dplyr (version 1.1.2) were used. Additional R packages used for hazard ratio-based sieve model fitting included survival (3.5-5), MASS (version 7.3-60), and nnet (version 7.3-19). For creating tables and plots with results, the R packages ggpubr (version 0.6.0), ggpmisc (version 0.5.2), ggpol (version 0.0.7), gridExtra (version 2.3), gtable (version 0.3.3) and ggradar (GitHub version) were

used.
For classification sieve analysis, R (version 4.3.1) was used. For data pre-processing, the R packages here (version 1.0.1), dplyr (version 1.1.2), tidyr (version 1.3.0), readr (version 2.1.4), and argparse (version 2.2.2) were used. For estimating functions that predict trial arm given covariates, the R packages here (version 1.0.1), dplyr (version 1.1.2), tidyr (version 1.3.0), readr (version 2.1.4), argparse (version 2.2.2), SuperLearner (version 2.0-28.1), MASS (version 7.3-60), arm (version 1.13-1), kernlab (version 0.9-32), ranger (version 0.15.1), xgboost (version 1.7.5.1), glmnet (version 4.1-7), earth (version 5.3.2), and parallel (version 4.3.1) were used. For VIM analysis, the R packages here (version 1.0.1), dplyr (version 1.1.2), tidyr (version 1.3.0), tibble (version 3.2.1), argparse (version 2.2.2), SuperLearner (version 2.0-28.1), and vimp (version 2.3.1) were used. For creating plots and tables with results, the R packages tidyverse (version 2.0.0), cowplot (version 1.1.1), vimp (version 2.3.1), data.table (version 1.14.8), optparse (version 1.7.3), and knitr (version 1.45) were used.

All protein structures were generated using VMD56 (version 1.9.4). Using Python (version 3.9), NumPy57 (version 1.20.3) was used for data processing and MDAnalysis (version 2.0.0) was used for processing, editing, and generating PDB structure files.
For SARS-CoV-2 sequences, Nextclade (https://clades.nextstrain.org/) and Pangolin (https://cov-lineages.org/resources/pangolin.html) were used for lineage assignments.
For the neutralizing antibody assay, R (version 3.4.3) was used to calculate SARS-CoV-2 neutralizing titers using a four-parameter curve fit as the sample dilution at which a 50% reduction (IC50) of luciferase readout was observed compared with luciferase readout in the absence of serum (“High Control”).
For phylogenetic trees, FastTree v2.1.11 (Price et al. 2010) was used. The trees for each region were extracted and then visualized using the ggtree and patchwork packages in R (version 4.1.1.4).
To identify signature sites associated with vaccine status after phylogenetic correction that accounts for potentially spurious associations due to lineage effects, the LANL tool GenSig (Bricault et al. 2019, Bhattacharya et al. 2007) was used.
Table S5 was generated in Python (version 3.10.2). Fig. S8 was generated in R (version 3.6.3).

For cryoEM: Movie frame alignment, estimation of the microscope contrast-transfer function parameters, particle picking, and extraction (with a down-sampled pixel size of 1.686 Å and box size of 256 pixels2) were carried out using Warp (Tegunov et al. 2019); reference-free 2D classification was performed using cryoSPARC (Punjani et al. 2017); and particle images were subjected to Bayesian polishing using Relion (Zivanov et al. 2019). UCSF Chimera (Pettersen et al. 2004) and Coot (Emsley et al. 2010) were used to fit atomic models of S2L20, S309, and SARS-CoV-2 S (PDB 7SOB) into the cryo-EM maps. The model was then refined and rebuilt into the map using Coot, Rosetta (Frenz et al. 2019, Wang et al. 2016), and ISOLDE (Croll 2018). Model validation and analysis used Phenix (Liebschner et al. 2019). Figures were generated using UCSF ChimeraX (Goddard et al. 2018).

For ACE2 binding measurements using Biolayer interferometry: The data were baseline subtracted and the plots fitted using the Pall FortéBio / Sartorius analysis software (v.12.0). Data were plotted in GraphPad Prism (v.9.0.2).
For ACE2 binding measurements using surface plasmon resonance: Data were double reference-subtracted and fit to a 1:1 binding model using Biacore Evaluation software.

For manuscripts utilizing custom algorithms or software that are central to the research but not yet described in published literature, software must be made available to editors and reviewers. We strongly encourage code deposition in a community repository (e.g. GitHub). See the Nature Portfolio guidelines for submitting code & software for further information.

Data

Policy information about availability of data

All manuscripts must include a data availability statement. This statement should provide the following information, where applicable:

- Accession codes, unique identifiers, or web links for publicly available datasets
- A description of any restrictions on data availability
- For clinical datasets or third party data, please ensure that the statement adheres to our policy

The sequence data used in this study are available in two groups: Information pertaining to the SARS-CoV-2 sequences obtained from study participants, including their GISAID accession numbers, is provided in the Supplementary Data 1 file. The sequences curated by LANL to define the canonical variant sequences are available on GISAID through identifier EPI_SET_221208yn (https://doi.org/10.55876/gis8.221208yn).89 Available information includes contributors’ details, such as accession number, virus name, collection date, originating lab, submitting lab and the list of authors.
The deep mutational scanning (DMS) data used to identify the DMS antibody escape scores are available at https://raw.githubusercontent.com/jbloomlab/SARS2_RBD_Ab_escape_maps/651fe6fa5a7fccec2b662ddbb45b6d2c7421ae74/processed_data/escape_calculator_data.csv. The representative Protein Data Bank (PDB) complexes for the PDB escape scores (Supplementary Table 2) are available from the PDB (https://www.rcsb.org/).
The cryoEM structures have been deposited at the PDB (https://www.rcsb.org/) and at the EMDB (https://www.ebi.ac.uk/emdb/) under the following accession numbers: D_1000281320 = Lambda global refinement, PDB: 8VYE, EMDB: EMD-43658; D_1000281321 = Lambda NTD local refinement, PDB: 8VYF, EMDB: EMD-43659; D_1000281322 = Lambda RBD local refinement, PDB: 8VYG, EMDB: EMD-43660.
The data sharing policy of Janssen Pharmaceutical Companies of Johnson & Johnson is available at https://www.janssen.com/clinical-trials/transparency. The data needed to execute the custom code for the sieve analysis as well as the neutralizing antibody data supporting the findings of this study are proprietary to Janssen and may be obtained from the authors upon reasonable request as determined by an agreement with the Yale Open Data Access (YODA) Project to serve as the independent review panel for evaluation of data requests. Project metrics for past data requests via YODA are available at https://yoda.yale.edu/metrics/.

Research involving human participants, their data, or biological material

Policy information about studies with human participants or human data. See also policy information about sex, gender (identity/presentation), and sexual orientation and race, ethnicity and racism.

Reporting on sex and gender | Information on participant sex was self-reported, solicited, and collected by four predefined options (female, male, unknown,

Reporting on sex and gender	intersex).<br>Sadoff et al. 2021 NEJM (DOI: 10.1056/NEJMoa2101544) determined that sex had no meaningful impact on vaccine efficacy. As such, we scoped our sieve analyses accordingly, as any finding of viral features impacting vaccine efficacy by sex would likely be a false discovery or would need to be interpreted as a qualitative interaction.
Reporting on race, ethnicity, or other socially relevant groupings	While the present manuscript does not report on these groupings, the primary manuscripts (Sadoff et al. 2021 NEJM, Sadoff et al. 2022 NEJM) reported on Race or ethnic group (American Indian or Alaskan Native, Indigenous South American, Asian, Black, Native Hawaiian or other Pacific Islander, White, Multiracial, Not reported/unknown/missing) as well as Hispanic ethic group (Hispanic, Non-Hispanic, Not reported/unknown/missing). Race and ethnic group were reported by the participants. American Indian or Alaskan Native was reported only by participants residing in the United States.
Population characteristics	Table 2 of the manuscript reports the demographics of participants in Latin America in the sieve analysis cohort, and Supplementary Tables 7, 8, and 9 report similar information of participants in the US, South Africa, and all regions pooled, respectively, in the sieve analysis cohort.
Recruitment	To ensure diversity and inclusion in the ENSEMBLE trial and based on years of clinical trial experience, Janssen implemented a multifaceted plan for recruitment and enrollment of participants from underrepresented communities. The approach included intentional site selection, community engagement and awareness building, and educational and training support for investigators. Janssen also took steps to remove barriers clinical trial participants often face, including the use of demographic data to identify and utilize clinical trial sites located in underrepresented communities.<br>“We are committed to developing medicines and therapies that meet the needs of all people, and we know that diseases and drugs may impact people differently based on their race and ethnicity, so the alignment of clinical trial enrollment with patient population demographics is key,” said Staci Hargraves, Vice President of Patient and Portfolio Solutions, Janssen Research & Development, LLC, and Executive Sponsor of Janssen’s Diversity, Equity & Inclusion in Clinical Trials program. “Simple yet impactful decisions, such as making sure trial sites were located in accessible places within historically underserved communities, made a big difference in our ability to reach more participants.”<br>Once Janssen selected the ENSEMBLE sites and began recruitment efforts, Janssen’s employees built relationships with trial site investigators and staff to provide cultural competency training to help stimulate dialogue about diversity and maintain focus on enrolling and supporting underrepresented groups. These close collaborations with site leaders allowed Janssen to identify any roadblocks in real time and make changes to the recruitment efforts as needed.<br>Identifying clinical trial sites in diverse communities was only the first step, because other barriers to recruitment and enrollment also exist. Clinical research in the U.S. has a complicated history when it comes to marginalized populations. Past events such as the Tuskegee Syphilis Study, combined with ongoing systemic disparities in the healthcare system, have contributed to distrust in clinical research among many people. Building trust is critical, particularly given the urgency the pandemic presented.<br>“We felt it was our role to help people understand how clinical trials work — and how trials have evolved to ensure that participant safety and human rights are protected today,” said Hargraves.<br>To build trust with communities of color, Janssen worked with both local and national organizations, including prominent community advocacy groups and leaders, along with healthcare professional organizations. These groups helped Janssen identify trusted voices within communities who could disseminate information about ENSEMBLE and clinical research in general. Janssen also used its Research Includes Me patient education program to conduct local outreach, including the consumer-facing website ResearchIncludesMe.com, and the dispatch of mobile units of bilingual educators to large community events. These tools helped to dispel misinformation about present-day medical research by providing accessible and empowering education about the clinical trial process and the protections given to participants’ rights and privacy.<br>source: https://www.jnj.com/our-company/janssen-takes-multifaceted-approach-to-ensuring-diversity-equity-and-inclusion-in-its-covid-19-vaccine-trial<br>The fact that the trial was a randomized trial, with careful allocation concealment, minimizes the potential for selection bias. As stated in the Protocol (available with Sadoff et al. NEJM 2021): A placebo control was used to establish the frequency and magnitude of changes in clinical and immunological endpoints that may occur in the absence of active vaccine.<br>Randomization was used to minimize bias in the assignment of participants to vaccine groups, to increase the likelihood that known and unknown participant attributes (eg, demographic and baseline characteristics) were evenly balanced across vaccine groups, and to enhance the validity of statistical comparisons across vaccine groups. Blinded study vaccine was used to reduce potential bias during data collection and evaluation of study endpoints. Blinding was guaranteed by the preparation of the study vaccine by an unblinded pharmacist or other qualified study-site personnel with primary responsibility for study vaccine preparation and dispensing, and by the administration of vaccine in a masked syringe by a blinded study vaccine administrator. Participants were randomly assigned to 1 of the groups based on a computer-generated randomization schedule prepared before the study by or under the supervision of the sponsor and using the interactive web response system.<br>Participants were not compensated for their participation.
Ethics oversight	Inclusion & Ethics<br>The COV3001 (ENSEMBLE) study was reviewed and approved by all relevant local ethics committees and Institutional Review Boards. All participants provided written informed consent. All experiments were performed in accordance with the relevant guidelines and regulations.<br>Site PIs were invited as co-authors according to the enrollments performed in the study, and were given the opportunity for intellectual contribution.<br><br>The COV3001 (ENSEMBLE) study was reviewed and approved by the following local ethics committees and IRBs:<br>Argentina: ANMAT - Administración Nacional de Medicamentos, Alimentos y Tecnologia Médica (Capital Federal, La Plata, Ramos Mejia – Buenos Aires; Ciudad Autonoma de Buenos Aires), Comite de Etica Dr Carlos Barclay (Capital Federal, Buenos Aires; Ciudad Autonoma de Buenos Aires), Comision Conjunta de Investigacion en Salud – CCIS (La Plata, Ramos Mejia - Buenos Aires), Comite de Bioetica de Fundacion Huesped (Ciudad Autonoma de Buenos Aires), Comité de Docencia e Investigación DIM Clínica Privada (Ramos Mejia, Buenos Aires), Comité de Ética en Investigación Clínica y Maternidad Suizo

Argentina (Ciudad Autonoma de Buenos Aires), Comité de Ética en Investigación de CEMIC (Ciudad Autonoma de Buenos Aires), Comite de Etica en Investigacion DIM Clinica Privada (Ramos Mejia, Buenos Aires), Comite de Etica Hospital Italiano de La Plata (La Plata, Buenos Aires), Comite de Etiica en Investigacion Hospital General de Agudos J.M. Ramos Mejia (Ciudad Autonoma de Buenos Aires), Comitéde ética del Instituto Médico Platense (CEDIMP) (La Plata, Buenos Aires), IBC Fundacion Huesped (Ciudad Autonoma de Buenos Aires), IBC Helios Salud (Ciudad Autonoma de Buenos Aires), IBC Hospital General de Agudos J.M. Ramos Mejia (Ciudad Autonoma de Buenos Aires)
Brazil: ANVISA – Agência Nacional de Vigilância Sanitária (Salvador, Bahia; Barretos, Campinas, São Paulo, São Jose Rio Preto, Ribeirão Preto, São Caetano do Sul – São Paulo; Santa Maria, Porto Alegre – Rio Grande do Sul; Natal, Rio Grande do Norte; Para, Pará; Belo Horizonte, Minas Gerais; Rio de Janeiro, Nova Iguaçu – Rio de Janeiro; Curitiba, Paraná; Brasília, Distrito Federal; Campo Grande, Mato Grosso do Sul; Criciúma, Santa Catarina; Cuiabá, Mato Grosso), CONEP - Comissão Nacional de Ética em Pesquisa (Salvador, Bahia; São Paulo, São Paulo; Santa Maria, Rio Grande do Sul; Para, Pará;), CAPPESq – Comissão de Ética de Análise para Projetos de Pesquisa – HCFMUSP (São Paulo, São Paulo), CEP da Faculdade de Medicina de São José do Rio Preto – FAMERP (São Jose Rio Preto, São Paulo), CEP da Faculdade de Medicina do ABC/SP (São Paulo, São Paulo), CEP da Fundação Pio XII - Hospital do Câncer de Barretos/SP (Barretos, São Paulo), CEP da Liga Norteriograndense Contra o Câncer (Natal, Rio Grande do Norte), CEP da Pontificia Universidade Catolica de Campinas / PUC Campinas (Campinas, São Paulo), CEP da Real Benemérita Associaçao Portuguesa de Beneficência - Hospital São Joaquim (São Paulo, São Paulo), CEP da Santa Casa de Misericórdia de Belo Horizonte (Belo Horizonte, Minas Gerais), CEP da Secretaria Municipal De Saúde do Rio de Janeiro – SMS/RJ (Rio de Janeiro, Rio de Janeiro), CEP da Universidade de São Caetano do Sul (CEP da Universidade de São Caetano do Sul, São Paulo), CEP da Universidade Federal de Mato Grosso do Sul – UFMS (Campo Grande, Mato Grosso do Sul), CEP da Universidade Federal de Minas Gerais (Belo Horizonte, Minas Gerais), CEP do Centro de Referência e Treinamento DST/AIDS (São Paulo, São Paulo), CEP do do INI-Ipec/Fiocruz (Rio de Janeiro, Rio de Janeiro), CEP do Grupo Hospitalar Conceição / RS (Porto Alegre, Rio Grande do Sul), CEP do Hospital das Clínicas da Faculdade de Medicina de Ribeirão Preto/USP (Ribeirão Preto, São Paulo), CEP do Hospital de Clinicas da Universidade Federal do Parana - HCUFPR / PR (Curitiba, Paraná), CEP do Hospital de Clínicas de Porto Alegre/HCPA (Porto Alegre, Rio Grande do Sul), CEP do Hospital Geral de Nova Iguaçu (Nova Iguaçu, Rio do Janeiro), CEP do Hospital Municipal São José (Criciúma, Santa Catarina), CEP do Hospital Pró-Cardíaco/RJ (Rio de Janeiro, Rio de Janeiro), CEP do Hospital Sírio Libanês (São Paulo, Sao Paulo), CEP do Hospital Universitário Júlio Muller / MT (Cuiabá, Mato Grosso), CEP do Hospital Universitário Professor Edgard Santos – UFBA (Salvador, Bahia), CEP do Instituto de Cardiologia do Distrito Federal (Brasília, Distrito Federal), CEP do Instituto de Infectologia Emílio Ribas/SP (São Paulo, Sao Paulo), CEP do Instituto de Saude e Bem Estar da Mulher - ISBEM / SP (São Paulo, Sao Paulo), CEP em Seres Humanos do HFSE - Hospital Federal dos Servidores do Estado (Rio de Janeiro, Rio de Janeiro), CONEP - Comissão Nacional de Ética em Pesquisa (Brasília, Distrito Federal, Salvador, Bahia; Belo Horizonte, Minas Gerais; Cuiabá, Mato Grosso; Campo Grande, Mato Grosso do Sul; Nova Iguaçu, Rio Janeiro – Rio Janeiro; Barretos, Campinas, Sao Jose Rio Preto, São Caetano do Sul, Sao Paulo, Ribeirão Preto – Sao Paulo; Porto Alegre, Rio Grande do Sul; Natal, Rio Grande do Norte; Curitiba, Paraná; Criciúma, Santa Catarina)
Chile: Comité de Ética de Investigación en Seres Humanos (Santiago, Region Met), Comité Ético Científico Servicio de Salud Metropolitano Central (Santiago, Region Met), Instituto de Salud Pública de Chile (Santiago, Region Met; Talca, Temuco), Comité Ético-Científico Servicio de Salud Metropolitano Sur Oriente (Talca, Santiago), Comité de Evaluación Ética Científica Servicio de Salud Araucanía Sur Temuco (Temuco), Comité Ético Científico Servicio de Salud Metropolitano Central (Viña del Mar)
Colombia: CEI de la Fundación Cardiovascular de Colombia (Floridablanca), Comité de Ética en Investigación Clínica de la Costa (Barranquilla), INVIMA - Instituto Nacional de Vigilancia de Medicamentos y Alimentos (Colombia) (Barranquilla), Comite de Etica en Investigacion de la E.S.E. Hospital Mental de Antioquia (Santa Marta), Comite de Etica en la Investigacion CAIMED (Bogotá), INVIMA - Instituto Nacional de Vigilancia de Medicamentos y Alimentos (Colombia) (Bogotá), Comite Corporativo de Etica en Investigacion de la Fundacion Santa Fe de Bogota (Bogotá), Comité de Ética e Investigación Biomédica de la Fundación Valle del Lili (Cali), Comite de Etica e Investigacion IPS Universitaria (Medellin), Comite de Etica en Investigacion Asustencial Cientifica de Alta Complejidad (Bogotá), Comite de Etica en Investigacion Biomedica de la Corporacion Cientifica Pediatrica de Cali (Cali), Comité de Ética en Investigación Clínica de la Costa (Barranquilla), Comite de Etica en Investigacion de la E.S.E. Hospital Mental de Antioquia (Barrio Barzal Villavicencio), Comite de Etica en Investigacion del area de la Salud de la Universidad del Norte (Barranquilla), Comite de Etica en Investigacion Medplus Centro de Recuperación Integral S.A.S (Bogotá), Comité de Ética en Investigaciones CEI-FOSCAL (Floridablanca), Comite de Etica en la Investigacion CAIMED (Bogotá), Comite de Etica para Investigacion Clinica(CEIC) de la Fundacion Centro de Investigacion Clinica CIC (Medellin), Comite de Investigaciones y Etica en Investigaciones Hospital Pablo Tobon Uribe (Medellin), INVIMA - Instituto Nacional de Vigilancia de Medicamentos y Alimentos (Colombia) (Barranquilla, Bogotá, Cali, Floridablanca, Medellin
Mexico: CEI del Hospital Civil de Guadalajara Fray Antonio Alcalde (Guadalajara, Jalisco), CEI Hospital La Mision (Tijuana, Baja California Norte), CI del Hospital Civil de Guadalajara Fray Antonio Alcalde (Guadalajara, Jalisco), CI Hospital La Mision (Tijuana, Baja California Norte), Comite de Bioseguridad del Instituto Nacional de Salud Publica (Mexico, Distrito Federal; Cuernavaca, Morelos), Comite de Etica en Investigacion del Instituto Nacional de Salud Publica (Mexico, Distrito Federal; Cuernavaca, Morelos), Comité de Bioseguridad del Hospital La Misión S.A. de C.V. (Tijuana, Baja California Norte; Oaxaca, Oaxaca; Merida, Yucatán; Tijuana, Baja California Norte), Comité de Bioseguridad de la Coordinación de Investigación en Salud (IMSS) (Mexico, Estado de Mexico), Comité de Bioseguridad de Médica Rio Mayo (CLINBOR) (Mexico, Distrito Federal), Comité de Bioseguridad del Hospital Universitario "Dr. José Eleuterio González" (Monterrey, Nuevo León), COFEPRIS (Comisión Federal para la Protección contra Riesgos Sanitarios) (Cuernavaca, Morelos; Mexico, Distrito Federal; Monterrey, Nuevo León; Oaxaca, Oaxaca; Merida, Yucatán), Comite de Etica de la Fac de Med de la UANL y Hospital Universitario "Dr. Jose Eleuterio Gonzalez" (Monterrey, Nuevo León), Comite de Etica en Investigacion de la Unidad de Atencion Medica e Investigacion en Salud S.C. (Merida, Yucatán), Comite de Etica en Investigacion de Medica Rio Mayo S.C. (Mexico, Distrito Federal), Comite de Etica en Investigacion de Oaxaca Site Management Organization, S.C. (Oaxaca, Oaxaca), Comite de Etica en Investigacion del Centro Medico Nacional Siglo XXI (IMSS) (Mexico, Estado do Mexico), Comité de Investigación de la Coordinación de Investigación en Salud (IMSS) (Mexico, Estado do Mexico), Comite de Investigacion de la Unidad de Atencion Medica e Investigacion en Salud S.C. (Merida, Yucatán), Comite de Investigacion de Oaxaca Site Management Organization, S.C. (Oaxaca, Oaxaca), Comité de Investigación del Hospital Universitario José Eleuterio González (Monterrey, Nuevo León), Comite de Investigacion Medica Rio Mayo, S.C. (Mexico, Distrito Federal)
Peru: Comite Nacional Transitorio de Etica en Invest. de los Ensayos Clinicos de la enfermedad COVID-19 (Iquitos - Maynas, Loreto; Lima, San Miguel – Lima), INS - Instituto Nacional de Salud (Peru) (Lima, San Miguel – Lima; Callao; Iquitos – Maynas, Loreto)
South Africa: Department Agriculture, Forestry and Fisheries (DAFF) (Port Elizabeth, Mthatha – Eastern Cape; Cape Town, Worcester – Western Cape; Durban, Ladysmith, Vulindlela – KwaZulu-Natal; Johannesburg, Pretoria, Mamelodi East, Soweto,

Tembisa – Gauteng; Rustenburg, Klerksdorp – North West; Bloemfontein, Free State; Middelburg, Mpumalanga; Dennilton, Limpopo), Pharma Ethics (Port Elizabeth, Eastern Cape; Durban, Ladysmith – KwaZulu-Natal; Cape Town, Western Cape; Pretoria, Mamelodi East, Johannesburg, Tembisa – Gauteng; Rustenburg, Klerksdorp – North West; Bloemfontein, Free State; Middelburg, Mpumalanga; Dennilton, Limpopo), SAHPRA - South African Health Products Regulatory Authority (Port Elizabeth, Mthatha – Eastern Cape; Cape Town, Worcester – Western Cape; Durban, Ladysmith, Vulindlela – KwaZulu-Natal; Johannesburg, Pretoria, Mamelodi East, Soweto, Tembisa – Gauteng; Rustenburg, Klerksdorp – North West; Bloemfontein, Free State; Middelburg, Mpumalanga; Dennilton, Limpopo), WIRB (Mamelodi East, Pretoria – Gauteng; Ladysmith, KwaZulu-Natal; Bloemfontein, Free State; Cape Town, Western Cape; Dennilton, Limpopo), Wits Health Consortium (Soweto, Johannesburg – Gauteng; Ladysmith, KwaZulu-Natal; Mthatha, Eastern Cape), Wits Institutional Biosafety Committee (Soweto, Pretoria, Johannesburg, Tembisa – Gauteng; Rustenburg, Klerksdorp – North West; Mthatha, Eastern Cape), University of Cape Town HREC (Cape Town, Worcester – Western Cape); University of Cape Town Institute of Infectious Disease & Molecular Medicine (Cape Town, Worcester – Western Cape), University of Cape Town Institutional Biosafety Committee (Cape Town, Worcester – Western Cape), SAMRC Human Research Ethics Committee Scientific Review (Durban, KwaZulu-Natal), Sefako Makgatho University Research Ethics Committee (SMUREC) (Pretoria, Gauteng), University of KwaZulu Natal Institutional Biosafety Committee (Durban, KwaZulu-Natal), University of KwaZulu-Natal Ethics (Durban, Vulindlela – KwaZulu-Natal), University of Stellenbosch Ethics Committee (Cape Town, Western Cape), University of KwaZulu Natal Institutional Biosafety Committee (Vulindlela, KwaZulu-Natal)
United States: Advarra IBC (Detroit, MI; Chapel Hill, NC; Boston, MA; Seattle, WA; Winston-Salem, NC; Austin, TX; Peoria, IL; Huntsville, AL; Long Beach, CA; Tucson, AZ), Biomedical Institute of New Mexico - IBC (Albuquerque, NM), Birmingham VA Medical Center - Alabama- IBC (Birmingham, AL), Clinical Biosafety Services (Hollywood, FL), Columbia University IBC (New York, NY), Copernicus Group IRB (Austin, Dallas, Houston, San Antonio – TX; Rochester, New York, Bronx, Binghamton – NY; Hillsborough, Hackensack, Newark, New Brunswick – NJ; West Palm Beach, Coral Gables, Hollywood, Miami, Orlando, Gainesville, Tampa, Hallandale Beach, Pinellas Park, The Villages, Jacksonville, Deland – FL; Fort Worth, Dallas, San Antonio – TX; Norfolk, Charlottesville – VA; Matairie, New Orleans – LA; Nashville, Knoxville, Memphis, Bristol – TN; Cincinnati, Cleveland, Columbus, Akron – OH; Detroit, Ann Arbor, Grand Rapids – MI; Philadelphia, Pittsburgh – PA; Stanford, San Diego, San Francisco, Oakland, Long Beach, Anaheim, Sacramento, West Hollywood – CA, Las Vegas, Reno – NV; Chicago, Peoria – IL; Omaha, NE; Mobile, Birmingham, Huntsville – AL; St Louis, Greer, Kansas City – MO; Boston, MA; Harrisburg, SD; Decatur, Atlanta, Savannah – GA; Baltimore, Rockville, Annapolis – MD; New Haven, Hartford – CT; Chapel Hill, Raleigh, Fayetteville, Charlotte, Durham, Winston-Salem – NC; Indianapolis, Valparaiso, Evansville – IN; Seattle, WA; Aurora, CO; Lexington, Louisville – KY; Murray, West Jordan, Salt Lake City – UT; Phoenix, Tucson, Glendale – AZ; Spartanburg, Columbia, North Charleston, Anderson, Charleston, Mount Pleasant – SC; Portland, Medford, Corvallis – OR; Albuquerque, Gallup – NM; Little Rock, AR; Jackson, MS; Newport News, VA, Minneapolis, MN; Lenexa, KS), WIRB (Hackensack, NJ; Dallas, TX; Baltimore, MD; Chicago, IL; Aurora, CO; Winston-Salem, NC; Minneapolis, MN; Orlando, Miami, Gainesville – FL; Philadelphia, Pittsburgh – PA; Boston, MA; St Louis, MO; Bronx, New York, NY; New Brunswick, NJ; Phoenix, AZ; Birmingham, AL; Louisville, KY; Albuquerque, NM; New Orleans, LA; Baltimore, MD; San Francisco, CA; Tampa, FL; Aurora, CO; Columbia, SC; Decatur, GA; Reno, NV; Raleigh, NC; Little Rock, AS), Clinical Biosafety Services (Dallas, San Antonio – TX; San Diego, CA; Lexington, KY; Murray, UT; Greer, Kansas City, St Louis – MO; Rockville, MD; Las Vegas, NV; Cincinnati, Columbus, Akron – OH; Phoenix, Tucson, Glendale – AZ; North Charleston, Anderson – SC; Orlando, Pinellas Park, The Villages, Miami – FL; Birmingham, AL; Valparaiso, Evansville – IN; Lenexa, KS), Columbia University IBC (Bronx, New York), Durham VA Medical Center-IBC (Raleigh, NC), Emory University IRB (Decatur, GA), Environmental Health and Safety Office (Atlanta, GA), Institutional Biosafety Committee (New Orleans, LA), James A. Haley Veterans Hospital_IBC (Tampa, FL), Jesse Brown VA Medical Center- IBC (Chicago, IL), Mass General Brigham IBC (Boston, MA), Mount Sinai- Icahn School of Medicine IBC (New York, NY), New York Blood Center IBC (New York, NY), OHSU IBC (Portland, OR), Partners Institutional Biosafety Committee (Boston, MA), Rocky Mountain Regional VA Medical Center-IBC (Aurora, CO), Rush University Medical Center (Chicago, IL), Rush University Medical Center IBC (Chicago, IL), Rutgers Institutional Biosafety Committee (New Brunswick, NJ), Saint Louis University IBC (St Louis, MO), Saint Michael's Medical Center IRB (Newark, NJ), Southeast Louisiana Veterans Health Care System IBC (New Orleans, LA), St. Jude Children's Research Hospital IBC Committee (Memphis, TN), St. Jude Children's Research Hospital IRB (Memphis, TN), Stanford University Administrative Panel on Human Subjects in Medical Research (Stanford, CA), Temple University – IBC (Philadelphia, PA), The University of Chicago Institutional Biosafety Committee (Chicago, IL), UAMS IBC (Little Rock, AS), UIC IBC (Chicago, IL), University of Alabama at Birmingham Institutional Biosafety Committee (Birmingham, AL), University of Arkansas IRB (Little Rock, AS), University of Kentucky Biological Safety (Lexington, KY), University of Kentucky IRB (Lexington, KY), University of Louisville IRB (Louisville, KY), University of Miami-IBC (Miami, FL), University of Mississippi Medical Center IRB (Jackson, MI), University of Pennsylvania Institutional Biosafety Committee (Philadelphia, PA), University of Pittsburgh IBC (Pittsburgh, Pennsylvania), University of South Florida IRB (Tampa, FL), University of Utah Institutional Biosafety Committee (Salt Lake City, UT), University of Utah IRB (Salt Lake City, UT), UTHealth – IBC (Houston, TX), VA Baltimore Research & Education Foundation (BREF)- IBC (Baltimore, MD), VA Central Arkansas Veterans Healthcare System-IBC (Little Rock, AS), VA James J. Peters Department of VA Medical Center-IBC (Bronx, NY), VA Medical Center - Atlanta-IBC (Decatur, GA), VA Medical Center San Francisco- IBC (San Francisco, CA), VA North Florida/South Georgia IBC (Gainesville, FL), VA North Texas Health Care System IBC (Dallas, TX), VA San Diego Healthcare System IBC (Phoenix, AZ), VA Sierra Nevada Health Care System-IBC (Reno, NV), Vanderbilt University Instituitional Review Board (Nashville, TN), Washington University IBC (St Louis, MO), WCG IBCS (Houston, TX; Orlando, FL), Western Institutional Review Board (San Diego, CA; Detroit, MI; New Orleans, LA; New York, NY), WIRB - IBCS Services (Chicago, IL; New Orleans, LA; Oakland, CA; Minneapolis, MN; Columbus, OH; Lexington, KY), WJB Dorne VA Medical Center IBC (Columbia, SC)

Note that full information on the approval of the study protocol must also be provided in the manuscript.

Field-specific reporting

Please select the one below that is the best fit for your research. If you are not sure, read the appropriate sections before making your selection.

☒ Life sciences ☐ Behavioural & social sciences ☐ Ecological, evolutionary & environmental sciences

For a reference copy of the document with all sections, see nature.com/documents/nr-reporting-summary-flat.pdf

Life sciences study design

All studies must disclose on these points even when the disclosure is negative.

Sample size	Using a two-step thought process, we considered rules for minimal numbers of endpoint cases to include a mark feature for sieve analysis (see the SAP, section "Filters/screens for sufficient variability of a mark feature for conducting sieve analysis"). In the first step, we applied the 'rule of 4', which is based off our knowledge that for binary marks, a minimum of 4 endpoint cases at each of the 2 mark levels is required to be able to achieve a Fisher's exact test p-value <0.05. Thus for any analysis we would set this as a minimal requirement, given that this amount of variability is necessary to possibly detect an association using an exact procedure. Secondly, we reasoned that the ENSEMBLE trial has a large number of COVID-19 endpoints, and it is of interest to find robust signals that do not depend on a few participants. Therefore, we made the rule considerably more stringent, up to a rule of 20. For the value of seeking robust signals, we were willing to give up the ability to detect less robust signals. Then for the severe-critical COVID-19 endpoint, because the number of endpoints was much less than for the COVID-19 endpoint, we relaxed the rule of 20 to a rule of 10, as the rule of 20 would permit very few analyses, and the rule of 10 still afforded an opportunity to detect significant associations. The rules of 10 or 20 were not chosen in order to meet a statistical power requirement for given alternative hypotheses; they were chosen under the knowledge that with these rules there is ample opportunity to detect many kinds of significant associations.
Data exclusions	For SARS-CoV-2 sequencing data: For samples that did not meet the following acceptance criteria, mutations in spike were not reported: >1 million raw reads, >750× mean genome coverage, >1000× mean spike gene coverage, 100% of spike gene with at least 200× coverage, and <10% missing data (Ns) in the final consensus sequence. A minimum of 6× depth per base was required to call consensus sequence. (Yu et al. 2023 PLOS Pathogens)<br>No data were excluded from the psVNA assay.
Replication	For SARS-CoV-2 next-generation sequencing: the sequencing assay has undergone full clinical validation, including determination of analytical sensitivity and specificity, limit of detection, accuracy, and assay precision (reproducibility and repeatability). Each sequence used in the analysis corresponds to the consensus sequence from the set of sequencing reads from a single run on a single clinical sample.<br><br>For the in-house developed psVNA assay, all attempts at replication were successful, based on meeting the following criteria: (1) The standard deviation of the high control (no serum control) over the assay plates was calculated for each SARS-CoV-2 variant and must have met the criterion of %CV <30%. (2) Furthermore, a panel of 4 anti-SARS-CoV-2 monoclonal antibodies and 8 heat-inactivated commercial human convalescent SARS-CoV-2 sera were tested in independent duplicates [2 different plates (independent duplicates) at different positions in the plate (duplicates per plate)]. The inter-assay variation, as well as the intra-assay variation of the titers, must have each met the same criterion of %CV < 30%. Each participant serum sample was only repeated within an assay (i.e. two technical replicates, with heat-inactivated serum samples undergoing two-fold serial dilution in duplicate over 10 columns).<br><br>Replication/reproducibility for binding data is characterized as follows: For Fig 8e: Biolayer interferometry: data from one biological replicate are shown with 1-2 technical replicates each; surface plasmon resonance: data from two biological replicates are shown with two technical replicates each; ELISA: data from two biological replicates are shown with two technical replicates each.
Randomization	In the ENSEMBLE trial, participants were randomized in parallel in a 1:1 ratio to receive intramuscular (IM) injections of Ad26.COV2.S or placebo (as described in Sadoff et al. 2022 NEJM). Randomization was done with the use of randomly permuted blocks in an interactive Web-response system.
Blinding	The ENSEMBLE trial was a double-blinded phase 3 efficacy trial. Laboratory investigators collecting the sequence data were blinded to treatment arm assignment during data collection.<br>For the data analysis: We performed unsupervised learning of the treatment-blinded trial sequence data to fully specify and down-select the set of AA sequence features that were studied for sieve effects. All statistical inferences were pre-specified in the SAP before treatment unblinding. Then, inferential statistical analysis (supervised learning that produces VE estimates, differential VE estimates, confidence intervals and p-values) was conducted in an automated/press-button fashion, with the inferences valid based on the pre-specification of inferences and the reproducibility of the computer code.

Reporting for specific materials, systems and methods

We require information from authors about some types of materials, experimental systems and methods used in many studies. Here, indicate whether each material, system or method listed is relevant to your study. If you are not sure if a list item applies to your research, read the appropriate section before selecting a response.

Materials & experimental systems

n/a	Involved in the study	
☐	☒	Antibodies
☐	☒	Eukaryotic cell lines
☒	☐	Palaeontology and archaeology
☒	☐	Animals and other organisms
☐	☒	Clinical data
☒	☐	Dual use research of concern
☒	☐	Plants

Methods

n/a	Involved in the study	
☒	☐	ChIP-seq
☒	☐	Flow cytometry
☒	☐	MRI-based neuroimaging

Antibodies

Antibodies used	None of the anti-nCoV monoclonal antibodies (mAbs) were available at suppliers at the time being. The anti-nCoV mAbs were described in publications (see below), and either produced in-house or ordered as a service from a supplier (GenScript- MamPilotTM guaranteed antibody expression service – COA delivered with the mAb batches). All listed below have neutralizing capabilities.<br>COVA2-15: manufactured in-house; reference: Philip J. M. Brouwer et al., Potent neutralizing antibodies from COVID-19 patients define multiple targets of vulnerability. Science369,643-650(2020), 10.1126/science.abc5902<br>C144: manufactured in-house; reference: Barnes, C.O., Jette, C.A., Abernathy, M.E. et al. SARS-CoV-2 neutralizing antibody structures inform therapeutic strategies. Nature 588, 682–687 (2020), 10.1038/s41586-020-2852-1<br>S2E12: manufactured in-house; reference: M. Alejandra Tortorici et al., Ultrapotent human antibodies protect against SARS-CoV-2 challenge via multiple mechanisms. Science370, 950-957(2020), 10.1126/science.abe3354<br>COV2-2130: manufactured by GenScript service; reference: Chen, R.E., Zhang, X., Case, J.B. et al. Resistance of SARS-CoV-2 variants to neutralization by monoclonal and serum-derived polyclonal antibodies. Nat Med 27, 717–726 (2021), 10.1038/s41591-021-01294-w<br>COV2-2381: manufactured by GenScript service; reference: Chen, R.E., Zhang, X., Case, J.B. et al. Resistance of SARS-CoV-2 variants to neutralization by monoclonal and serum-derived polyclonal antibodies. Nat Med 27, 717–726 (2021), 10.1038/s41591-021-01294-w<br>The NTD-targeted and RBD-targeted mAbs used in the ELISAs were purified previously (McCallum et al. Cell 2021 PMID 33761326; McCallum et al. Science 2021 PMID 34751595).
Validation	All antibody constructs were sequence-verified. Both the in-house produced antibodies as well as the GenScript produced batches were tested on purity (>90%) and tested in-house in binding experiments (ELISA, biolayer interferometry), neutralization (psVNA) against multiple SARS-CoV-2 Spike variants.

Eukaryotic cell lines

Policy information about cell lines and Sex and Gender in Research

Cell line source(s)	For the neutralizing antibody assay, a commercial cell line consisting of HEK293T target cells stably expressing the human ACE2 and human TMPRSS2 genes was used [CoronaAssay-293T(hACE2-hTMPRSS2), procured from VectorBuilder; Cat. CL0015].<br>While this cell line no longer appears to be sold by VectorBuilder, and an alternative source of the 293T cell line (ATCC) does not contain information on the sex of the fetus from which the cell line was derived, Lin et al. published in 2014 that "293 cells are of female provenance" based on genome sequencing of 293 cells and the lack of Y-chromosome-derived sequence in their data sets (doi: 10.1038/ncomms5767. PMID: 25182477). Therefore we infer that the commercial CoronaAssay-293T(hACE2-hTMPRSS2) cell line is most plausibly of female origin.<br>The Expi293F cells used were directly from ThermoFisher Scientific (product # A14527). See the response above regarding the likely sex of the fetus from which the cell line was derived.
Authentication	The commercial CoronaAssay-293T(hACE2-hTMPRSS2) cell line referred to above was not authenticated in-house. A Certificate of Analysis (COA) was provided by the vendor of the cell line (VectorBuilder). The authentication of the vendor was confirmed with an RT-qPCR test for hACE2 and hTMPRSS2 expression and compared to the unmodified 293T control cells.
Mycoplasma contamination	In the Certificate of Analysis (COA) provided by the vendor it was stated that the CoronaAssay-293T(hACE2-hTMPRSS2) cell line passed the performed mycoplasma test as negative.<br>ThermoFisher Scientific certified that the Expi293F cell line tested negative for mycoplasma.
Commonly misidentified lines (See ICLAC register)	None

Clinical data

Policy information about clinical studies
All manuscripts should comply with the ICMJE guidelines for publication of clinical research and a completed CONSORT checklist must be included with all submissions.

Clinical trial registration	ENSEMBLE ClinicalTrials.gov number, NCT04505722
Study protocol	Full trial protocol available with Sadoff et al. NEJM 2022: https://www.nejm.org/doi/suppl/10.1056/NEJMoa2117608/suppl_file/nejmoa2117608_protocol.pdf
Data collection	The trial began enrollment on September 21, 2020, and the data-cutoff date for the present analysis was January 22, 2021. Trial sites are listed in the Supplementary Appendix of Sadoff et al. 2021 (https://www.nejm.org/doi/suppl/10.1056/NEJMoa2101544/suppl_file/nejmoa2101544_appendix.pdf).<br>Molecular confirmation of SARS-CoV-2 infection by a central laboratory (University of Washington Virology laboratory) was used for case definition analysis. Next-generation sequencing was performed using at the Virology Laboratory of the University of Washington, Department of Laboratory Medicine and Pathology (UW Virology).<br>The reference strain (D614G) pseudotyped lentivirus neutralization assay (psVNA) was performed at Janssen.
Outcomes	The sieve analysis considers baseline SARS-CoV-2 seronegative per-protocol participants and the primary endpoint (first occurrence of molecularly confirmed, moderate to severe–critical COVID-19), as well as the severe–critical COVID-19 endpoint (secondary endpoint, first occurrence of molecularly confirmed severe-critical COVID-19), during the double-blinded period of follow-up.

In the sieve analysis, a primary endpoint case is defined as the moderate to severe-critical primary COVID-19 endpoint in the per-protocol baseline seronegative cohort, with disease onset starting 14 days post vaccination through to a participant's unblinding date. A severe endpoint case is the same, except with the severe-critical COVID-19 endpoint. Note that while the primary analyses (Sadoff et al. NEJM 2021, 2022) both counted endpoints starting 14 or 28 days post vaccination, all of the sieve analyses used endpoints starting 14 days post vaccination, given that similar results were expected and more COVID-19 endpoints could be included in the analysis.
Table S1 in Sadoff et al. NEJM 2021, 2022) describes the protocol-defined co-primary and secondary end points. In both the primary and secondary endpoints, molecularly confirmed COVID-19 was defined as a positive SARS-CoV-2 viral RNA result using a RT-PCR assay (Abbott) at the University of Washington. The case definitions of moderate and severe-critical COVID-19 were provided in Section 8.1.3.3 in the protocol provided with Sadoff et al. 2022 NEJM:

Case Definition for Moderate COVID-19
- A SARS-CoV-2 positive RT-PCR or molecular test result from any available respiratory tract sample (eg, nasal swab sample, sputum sample, throat swab sample, saliva sample) or other sample AND at any time during the course of observation:
- Any 1 of the following new or worsening signs AND any 1 of the following new or worsening symptoms:
Signs: Respiratory rate ≥20 breaths/minute, Abnormal saturation of oxygen (SpO2) but still >93% on room air at sea level, with SpO2 criteria adjusted according to altitude, Heart rate ≥90 beats/minute, Clinical or radiologic evidence of pneumonia,
Radiologic evidence of deep vein thrombosis (DVT)
Symptoms: Shortness of breath (difficulty breathing), Fever (≥38.0°C or ≥100.4°F), Cough, Sore throat; Malaise as evidenced by 1 or more of the following: - Loss of appetite, - Generally unwell, - Fatigue, - Physical weakness; Headache; Muscle pain (myalgia); Gastrointestinal symptoms (diarrhea, vomiting, nausea, abdominal pain)
OR
- Any 2 of the following new or worsening symptoms: Fever (≥38.0°C or ≥100.4°F), Shaking chills or rigors, Cough, Shortness of breath (difficulty breathing), Sore throat; Malaise as evidenced by 1 or more of the following: - Loss of appetite, - Generally unwell, - Fatigue, - Physical weakness; Headache, Muscle pain (myalgia); Gastrointestinal symptoms as evidenced by 1 or more of the following elements (diarrhea, vomiting, nausea, abdominal pain); Red or bruised looking feet or toes; New or changing olfactory or taste disorders.
* Having 2 or more elements of a symptom (eg, vomiting and diarrhea or fatigue and loss of appetite) is counted only as 1 symptom for the case definition. To meet the case definition, a participant would need to have at least 2 different symptoms.

Case Definition for Severe/Critical COVID-19
- A SARS-CoV-2 positive RT-PCR or molecular test result from any available respiratory tract sample (eg, nasal swab sample, sputum sample, throat swab sample, saliva sample) or other sample
AND any 1 of the following at any time during the course of observation:
- Clinical signs at rest indicative of severe systemic illness (respiratory rate ≥30 breaths/minute, heart rate ≥125 beats/minute, oxygen saturation (SpO2) ≤93% on room air at sea level*, or partial pressure of oxygen/fraction of inspired oxygen (PaO2/FiO2) <300 mmHg), with SpO2 criteria adjusted according to altitude,
- Respiratory failure (defined as needing high-flow oxygen, non-invasive ventilation, mechanical ventilation, or extracorporeal membrane oxygenation [ECMO])
-Evidence of shock (defined as systolic blood pressure <90 mmHg, diastolic blood pressure <60 mmHg, or requiring vasopressors)
- Significant acute renal, hepatic, or neurologic dysfunction
- Admission to the ICU
- Death
